# The Efficacy and Mechanism of Qinghua Jianpi Recipe in Inhibiting Canceration of Colorectal Adenoma Based on Inflammatory Cancer Transformation

**DOI:** 10.1155/2023/4319551

**Published:** 2023-02-15

**Authors:** Ting Liu, Juping You, Qingjian Gao, Yufeng Zhang, Wei Xu, Desong Kong, Li Chen, Bao Yuan, Haibing Hua

**Affiliations:** ^1^Department of Gastroenterology, Jiangyin Hospital of Traditional Chinese Medicine, Jiangyin Hospital Affiliated to Nanjing University of Chinese Medicine, Jiangyin, 214400 Jiangsu Province, China; ^2^Chinese Medicine Modernization and Big Data Research Center, Nanjing Hospital of Chinese Medicine Affiliated to Nanjing University of Chinese Medicine, Nanjing University of Chinese Medicine, Nanjing, 210022 Jiangsu Province, China; ^3^Department of Pharmacology, School of Pharmacy, Nanjing University of Chinese Medicine, Nanjing, 210023 Jiangsu Province, China

## Abstract

**Objective:**

This study is aimed at exploring the effect of Qinghua Jianpi Recipe on preventing colon polyp recurrence and inhibiting the progress of “inflammatory cancer transformation.” And another goal is to explore the changes of intestinal flora structure and intestinal inflammatory (immune) microenvironment of mice with colon polyps treated by Qinghua Jianpi Recipe and to clarify its mechanism.

**Methods:**

Clinical trials were conducted to confirm the therapeutic effect of Qinghua Jianpi Recipe on patients with inflammatory bowel disease. The inhibitory effect of Qinghua Jianpi Recipe on “inflammatory cancer transformation” of colon cancer was confirmed by an adenoma canceration mouse model. Histopathological examination was used to evaluate the effects of Qinghua Jianpi Recipe on intestinal inflammatory state, adenoma number, and pathological changes of adenoma model mice. The changes of inflammatory indexes in intestinal tissue were tested by ELISA. Intestinal flora was detected by 16S rRNA high-throughput sequencing. Short-chain fatty acid metabolism in the intestine was analyzed by targeted metabolomics. Network pharmacology analysis of possible mechanism of Qinghua Jianpi Recipe on colorectal cancer was performed. Western blot was used to detect the protein expression of the related signaling pathways.

**Results:**

Qinghua Jianpi Recipe can significantly improve intestinal inflammation status and function in patients with inflammatory bowel disease. Qinghua Jianpi Recipe could significantly improve the intestinal inflammatory activity and pathological damage of adenoma model mice and reduce the number of adenoma. Qinghua Jianpi Recipe significantly increased the levels of Peptostreptococcales_Tissierellales, NK4A214_group, Romboutsia, and other intestinal flora after intervention. Meanwhile, the treatment group of Qinghua Jianpi Recipe could reverse the changes of short-chain fatty acids. Network pharmacology analysis and experimental studies showed that Qinghua Jianpi Recipe inhibited the “inflammatory cancer transformation” of colon cancer by regulating intestinal barrier function-related proteins, inflammatory and immune-related signaling pathways, and free fatty acid receptor 2 (FFAR2).

**Conclusion:**

Qinghua Jianpi Recipe can improve the intestinal inflammatory activity and pathological damage of patient and adenoma cancer model mice. And its mechanism is related to the regulation of intestinal flora structure and abundance, short-chain fatty acid metabolism, intestinal barrier function, and inflammatory pathways.

## 1. Introduction

Colorectal cancer (CRC) is a malignant tumor originating in the colorectal mucosal epithelium. And it is one of the common malignant tumors in clinic. It is the third most common malignancy in the world. There are more than one million new cases of CRC in the world every year, and the number of deaths caused by CRC is as high as 500,000 [1, 2]. With the improvement of Chinese people's living standard, the change of life style, and diet structure, the incidence of CRC is increasing year by year, which seriously harms human health. Inflammatory bowel diseases (IBD) such as ulcerative colitis (UC) are considered major risk factors for CRC, and the degree of colon injury, lesion extent, duration, and activity of intestinal inflammation are closely related to prognosis and outcome [3, 4]. Studies have found that there is a certain correlation between the progression of UC to CRC and the lesion site. Compared with normal people, the risk of tumor development in total colitis, left colitis, and proctitis is 14.8, 2.8, and 1.7 times, respectively. Current studies suggest that IBD is related to genetic, environmental, immune, intestinal flora, and psychological factors, among which the role of intestinal flora in the occurrence and development of IBD cannot be ignored [5–7]. There is a very complex relationship between intestinal dysbiosis and IBD. The dysbiosis of intestinal flora may be the inducing factor of IBD, and IBD may aggravate the dysbiosis of intestinal flora, but the mechanism of interaction is still not very clear [8]. There is evidence that certain medications for UC may reduce the risk of CRC, while the risk of stationary disease is lower than that of chronic active disease. Therefore, it is of great value to seek therapeutic methods that can control intestinal inflammation, promote intestinal mucosal repair, and maintain intestinal homeostasis for the prevention and treatment of “inflammatory cancer transformation” of CRC.

There is no disease name of colonic polyps in traditional Chinese medicine (TCM) literature, but related clinical symptoms are described, such as diarrhea, hematochezia, and enterogastritis. Studies have shown that abnormal intestinal immune response is an important link in the occurrence and development of IBD and CRC, as well as one of the inducing factors for the persistence and recurrence of intestinal inflammation [5]. In patients with active UC or CRC, one of the most important changes of intestinal immune barrier dysfunction is the abnormal increase of inflammatory factors and the excessive activation of mucosal lamina propria immune system, especially the destruction of intestinal mucosal immune barrier by proinflammatory factors such as TNF-*α*, INF-*γ*, IL-13, IL-6, and IL-10, which leads to the increase of intestinal mucosal permeability [7, 8]. It is extremely important in the pathophysiology of CRC. A large number of studies have found that some effective components of TCM can significantly inhibit the expression of inflammatory factors in the intestinal mucosa of UC or CRC animal models or patients and reduce the intestinal inflammatory response, so as to promote the balance of intestinal mucosal immune regulation and improve the intestinal barrier function [9, 10]. Common therapeutic recipes include Shenling Baizhu powder, Lizhong decoction, Wumei pill, Sishen pill, and Pingwei powder [11]. According to the TCM, treatment based on syndrome differentiation can regulate the internal environment of the patient, enhance the immune function of the body, and adjust the anticancer ability of the body from a macro perspective. However, the adverse reactions of TCM are mild, because Chinese medicine is taken from natural animals, plants, and minerals.

Qinghua Jianpi Recipe is composed of Dangshen (Codonopsis pilosula (Franch.) Nannf), Huangqin (Scutellaria baicalensis Georgi), Baizhu (Atractylodes macrocephala Koidz), Fuling (Wolfiporia cocos (F.A. Wolf) Ryvarden & Gilb), Huangqi (Astragalus membranaceus (Fisch.) Bunge), Yiyiren (Semen Coicis), Wumei (Fructus Mume), Fangfeng (Saposhnikovia divaricata (Turcz.) Schischk), Chenpi (Citrus reticulata Blanco), Niuxi (Achyranthes bidentata Blume), and Gancao (Glycyrrhiza uralensis Fisch). It is a recipe created by Professor Zhu Bingyi, a famous Chinese doctor of TCM, for preventing recurrence after colon polyp removal. It is suitable for the pathogenesis of colorectal polyp and has achieved good clinical effect in clinical use. However, its therapeutic mechanism needs to be further clarified to provide a scientific basis for clinical medication and lay a theoretical foundation for the development of new drugs for the prevention and treatment of postoperative recurrence of colon polyps in colon cancer.

Therefore, based on the above points, this study is aimed at exploring the effect of Qinghua Jianpi Recipe on blocking the process of “inflammation-cancer transformation” and preventing the recurrence of colorectal polyps and CRC. And whether the above effects are caused by Qinghua Jianpi Recipe through regulating intestinal flora and improving intestinal the inflammatory (immune) microenvironment. Moreover, the effects of Qinghua Jianpi Recipe on s intestinal immune microenvironment and inflammatory process and the role of intestinal flora in the pathogenesis of colon polyps were systematically observed in the study.

## 2. Materials and Methods

### 2.1. Clinical Trial Scheme

All clinic participants in this study were screened and collected from the Department of Gastroenterology and Colorectal Surgery of Jiangyin Hospital of Chinese Medicine Affiliated to Nanjing University of Chinese Medicine from January 2021 to August 2022. Participants were diagnosed with ulcerative colitis (active stage) and randomly divided into 2 groups according to the order of treatment. There were 30 cases in the therapeutic group and 30 cases in the control group, including 35 male patients and 25 female patients. The study was approved by the Ethics Committee of Jiangyin Hospital of Chinese Medicine Affiliated to Nanjing University of Chinese Medicine (Approval No. 2020102) and carried out under the supervision and guidance of the committee. In the therapeutic group, Qinghua Jianpi Recipe granule was taken with warm water, one dose a day, three times a day. The Chinese medicine granule was dissolved in 300 mL boiling water after each meal, and the medicine was taken after the water temperature was moderate. Two weeks was the course of treatment, and two courses of treatment were taken continuously. The control group was given mesalazine enteric-coated tablet 1.0 g alone, three times a day, for 30 days. During the treatment, the enrolled patients did not use intestinal probiotics, prebiotics, broad-spectrum antibiotics, and other drugs, and other nutritional support treatment plans were consistent between the two groups. All participants were tested for the following indicators before and after treatment:

IBD activity was graded and converted to a quantitative score (2 endoscopist scores) according to the degree of grading by Baron endoscopic review: 0: grade 0, normal mucosa; 1: grade I, mucosal congestion and blurred blood vessels; 2 points: grade II, mucosal contact bleeding; 3: grade III, spontaneous hemorrhage of mucosa; and 4 points: grade IV. Ulcers of varying sizes were seen in the mucosa.

The standard modified Mayo index was used to rate the disease activity index. The score directly reflects the severity grade of the disease and the microscopic inflammatory activity grade for clinical treatment (Table 1).

Clinical efficacy was evaluated with the modified rating scale. Clinical efficacy: A decrease of ≥ 30% or ≥3 from baseline in the total Mayo score, accompanied with a decrease of ≥1 in the hematostoecium subscore or an absolute hematostoecium subscore of 0 or 1. Clinical remission: The total Mayo score ≤ 2 and no single item subscore > 1. Endoscopic response: Mayo score endoscopic subscore decreased by at least 1 from baseline. Mucosal healing: the absolute Mayo score of 0 or 1 on the endoscopic subscore.

### 2.2. Animal Grouping, Feeding, and Sampling

All animal experiments were performed according to the guidelines of Animal Experimentation at Nanjing University of Chinese Medicine (Nanjing, China). The research has been reviewed by the Ethics Committee of Animal Experiments at Nanjing University of Chinese Medicine (Approval No. 202111A041). All animals were kept in a pathogen-free environment and fed ad lib. The procedures for the care and use of animals were approved by the Ethics Committee, and all applicable institutional and governmental regulations concerning the ethical use of animals were followed.

According to the literature and previous experimental results, five cycles of AOM combined with DSS were selected to establish the mouse adenoma canceration model. Except for the blank group, all mice were intraperitoneally injected with AOM at a dose of 10 mg/kg on the first day. After a week's rest, they were given drinking water containing 2% DSS for 5 days and then, it was changed to ordinary drinking water for 14 days as a cycle. The experiment was completed at the 15th week. Based on the reported time point at which the adenoma appeared in this model mouse, three mice were randomly selected from the fifth week to be put to execution every week (except the blank group). At the time point of adenoma appearance, different concentrations of Qinghua Jianpi Recipe were given through intragastric administration. Sixty C57BL/6 mice were randomly divided into 4 groups: blank group, model group (AOM/DSS), Qinghua Jianpi Recipe low-dose group (15.6 g·kg^−1^), and Qinghua Jianpi Recipe high-dose group (31.2 g·kg^−1^).

After 16 weeks of feeding, blood was taken from the eyeballs, and the animals were sacrificed by neck removal. Tissue samples of the liver, spleen, colon, and intestinal contents were collected. The colon tissue was longitudinally cut to collect intestinal contents and then flattened to count the number of adenomas.

### 2.3. Intestinal Histopathology and Collagen Deposition Examination

Intestinal tissues were sampled in a fume cupboard. Tissue samples with appropriate target location and size were carefully separated with sampling instruments and fixed with 10% formalin solution. After fixation and washing, the specimen was dehydrated, which was completed by an automatic dehydrator. Paraffin is immersed in the tissue, and the wax-soaked tissue is placed in melted solid paraffin, which is solidified to form tissue wax blocks. Then, the paraffin slicer was used to cut the wax block into slices about 4 *μ*m thick. Use ophthalmic tweezers to gently spread the wax tape on the slide to fix the tissue, and put the slide into the slide baking machine at 60°C for 15-30 minutes to remove the paraffin wax that dissolved the tissue space. The slides were taken out, and the tissue sections were stained with hematoxylin-eosin (HE). And the pathological changes of intestinal tissues were observed under the microscope. The statistical analysis was performed using the analysis software ImageJ.

### 2.4. General Situation Assessment and Disease Activity Index (DAI) Scores

After administration, hair luster, mental activity, body weight change, food intake and water intake, stool characteristics, whether there is blood in the stool, and the degree of blood in the stool were observed and recorded at the same time every day (if there is no visible blood in stool, fecal occult blood test kit was used to determine whether there is blood in the stool). After weighing the body weight of mice at a regular time every day, the rate of change in body weight was calculated, and the changes in fecal traits, occult blood, and hematochezia of mice were comprehensively observed by naked eye. The DAI score of rats in each group was calculated according to the computational formula, DAI = (weight loss fraction + stool trait fraction + hematochezia fraction)/3, to evaluating the changes of inflammatory activity in mice.

### 2.5. ELISA Detection

MPO levels in intestinal tissue and IL-1*β*, IL-6, IL-18, and TNF-*α* levels in serum were detected by ELISA. After balancing at room temperature for 20 min, take out the lath, set the standard well and sample well, and add standard 50 *μ*L of different concentrations to the standard well. Add sample 10 *μ*L to the test sample well; then, add sample dilution 40 *μ*L; do not add to the blank well. In addition to the blank well, add 100 *μ*L horseradish peroxidase-labeled detection antibody to each well of standard and sample wells, seal the reaction well with seal plate membrane, and incubate at 37°C for 60 min. Discard liquid, dry with washing solution, add washing solution to each well, rest for 1 min, swing the washing solution, dry with washing solution, and repeat washing plate 5 times. Add substrates A and B 50 *μ*L to each well, and incubate at 37°C for 15 min away from light. Add the stop solution 50 *μ*L to each well, and measure the OD value of each well at 450 nm wavelength within 15 min.

### 2.6. High-Throughput Sequencing of 16S rRNA in Mouse Feces

E.Z.N.A.® Soil DNA Kit was used to extract microbial DNA from feces samples of mice. The specific steps were in accordance with the instructions. Primers for v4-V5 region of the 16S rRNA gene were used in this study. The upstream primers were 515F 5′-GTGCCAGCMGCCGCGG-3′, and the downstream primers were 907R 5′-CCGTCAattCMTTTRAGTTT-3′. PCR reaction system contains 5× FastPfu Buffer 4 *μ*L, (2.5 mM) dNTPs 2 *μ*L, (5 *μ*M) upstream and downstream primers 0.8 *μ*L, FastPfu Polymerase 0.4 *μ*L, DNA template 10 ng, and ddH_2_O to the total volume of 20 *μ*L. After the PCR reaction system was fully mixed, the amplification was carried out by the machine after heating up and the reaction procedure was as follows: predenaturation at 95°C for 2 min, denaturation at 95°C for 30 s, annealing at 55°C for 30 s, extension at 72°C for 30 s, cycle 25 times, and extension at 72°C for 10 min. All PCR products were detected by 2% agarose Gel electrophoresis and recovered and purified by AxyPrep DNA Gel Extraction Kit according to the instructions.

The purified PCR products were quantified using Qubit®3.0, and Illumina genomic DNA library was constructed based on Illumina genomic DNA library preparation program. The amplicon library was paired and sequenced on Illumina MiSeq platform according to the standard protocol (2 × 250).

### 2.7. Targeted Metabolomics Analysis of Short-Chain Fatty Acids

20 mg of feces samples was taken from each group; 120 *μ*L methanol was added, homogenized for 1 min, and centrifuged at 4°C for 14000 r/min for 10 min; 100 *μ*L was absorbed. The supernatant is extracted for 14000 r/min for 10 min at 4°C; 50 *μ*L supernatant was absorbed to prepare a liquid injection flask for UPLC-QTOF-MS analysis. For quality control sample (QC), weigh 10 mg of each stool sample and use it as QC sample after treatment in accordance with the above method. Before analysis, continuous sample injection 3 times is performed to ensure good stability of the instrument, and QC sample injection after every 8-needle sample during analysis. GC-MS instrument (2030/TQ-8040NX), Agilent DB-Wax capillary column (30 m × 0.25 mm, 0.5 *μ*m), was used for detection and analysis. The heating procedure is as follows: the initial temperature was 80°C and maintained for 2 min; then, the temperature was raised to 180°C at 10°C/min and maintained for 3 min; then, the temperature was raised to 230°C at 40°C/min and maintained for 2 min; injection temperature 230°C; split ratio 5 : 1; injection volume: 1 *μ*L; carrier gas: high purity helium (He); flow rate: 1 ml/min; electron bombardment source (EI); interface temperature: 240°C; ion source temperature: 230°C; select the ion monitoring (MRM) mode. For data processing and analysis, MarkView 1.3.1 software was used to preprocess the original data, such as peak identification, alignment, noise filtering, and peak area normalization, to obtain the data matrix file containing information, such as metabolite retention time, mass-to-charge ratio, and intensity. It was imported into SIMCA-P 14.0 software for principal component analysis (PCA) and orthogonal partial least squares discriminant analysis (OPLS-DA). Variables that meet variable importance projection value (VIP) > 1.0 and *P* < 0.05 is considered a differential variable. HMDB (http://www.hmdb.ca/) and KEGG (http://www.kegg.ca/) were used to identify the difference variables, and the error margin was set as 0.1 Da. MetaboAnalyst 5.0 database (http://www.metaboanalyst.ca/) was used for analysis of metabolic pathways. And if impact value which is greater than 0.10, metabolic pathway is considered to be a potential target path.

### 2.8. Network Pharmacology Analysis

Compound chemical composition and drug targets were collected and predicted. Dangshen (Codonopsis pilosula (Franch.) Nannf), Huangqin (Scutellaria baicalensis Georgi), Baizhu (Atractylodes macrocephala Koidz), Fuling (Wolfiporia cocos (F.A. Wolf) Ryvarden & Gilb), Huangqi (Astragalus membranaceus (Fisch.) Bunge), Yiyiren (Semen Coicis), Wumei (Fructus Mume), Fangfeng (Saposhnikovia divaricata (Turcz.) Schischk), Chenpi (Citrus reticulata Blanco), Niuxi (Achyranthes bidentata Blume), and Gancao (Glycyrrhiza uralensis Fisch) were inputted to the TCMSP database (https://old.tcmsp-e.com/tcmsp.php), to collect all compounds [12]. Then, according to the pharmacokinetic principle, the suitable compounds are screened based on oral bioavailability (OB) ≥ 30% and drug-like index (DL) ≥ 0.18. Drug target data were obtained from DrugBank (https://go.drugbank.com/) and standardized using the UniProt (https://www.Uniprot.org/) database [13]. Based on GeneCards (https://www.genecards.org/) and OMIM-NCBI databases (https://www.ncbi.nlm.nih.gov/omim), IBD as keyword was used to screen disease targets, while for species selection “Homosapiens” was used. Draw Venn Diagrams tool in R software is used to process drug targets and disease targets obtained in the above steps to obtain the intersection gene targets and output Venn Diagram for display.

PPI network interaction analysis was performed on the active chemical components and core targets of Qinghua Jianpi Recipe by using the STRING database and Cytoscape3.7.1 software. According to the requirements of Cytoscape, the “source-target” data table was constructed, and the NetworkAnalyzer plug-in was used to construct the regulation network of TCM, active component, and target. In the generated regulation network, nodes represent the interaction between TCM, active component, and target, and edges represent the interaction between active the component and target disease. Furthermore, MCC algorithm of CytoHubba plug-in was used to calculate and construct PPI network of each target. Based on Bioconductor (https://www.bioconductor.org/) in the R package (https://www.r-project.org/) and clusterProfiler 3.12.0 to GO (gene ontology) core target function and KEGG pathway enrichment analysis (KEGG pathway analysis), Homo sapiens was selected and a threshold *P* < 0.05 was set. According to the results, the core target-critical pathway network was conducted in Cytoscape 3.7.1 software.

### 2.9. Western Blot Analysis

After tissue sample fragmentation, precooled RIPA lysate was added; then, PMSF and phosphatase inhibitors were immediately added, incubated on ice for 30 minutes, then transferred into a tube, and centrifuged at 4°C, 15 000 r·min^−1^ for 15 minutes; and supernatant was obtained as the cell lysate. The protein concentration was determined by the BCA method. Electrophoresis buffer was added and incubated in boiling water for 10 minutes. Proteins (50 g per sample) were resolved on SDS-PAGE gel at 100 V and transferred to a blotting membrane for 1.5 h, using skim milk powder as a blocking agent. Then, the membranes were incubated with the first antibody. Membranes were washed, and then, the secondary antibody was incubated and the chemiluminescent agent ECL was used for visualization.

### 2.10. Statistical Methods

The experimental data were processed by SPASS 23.0, and the parameter values were expressed as mean + standard deviation, ANOVA, and *T*-test were used to compare the differences between groups, and the comparison between groups was analyzed by one-way ANOVA. *P* < 0.05 means the difference is statistically significant.

## 3. Results

### 3.1. Qinghua Jianpi Recipe Has a Significant Clinical Effect on Ulcerative Colitis

**Figure 1:**
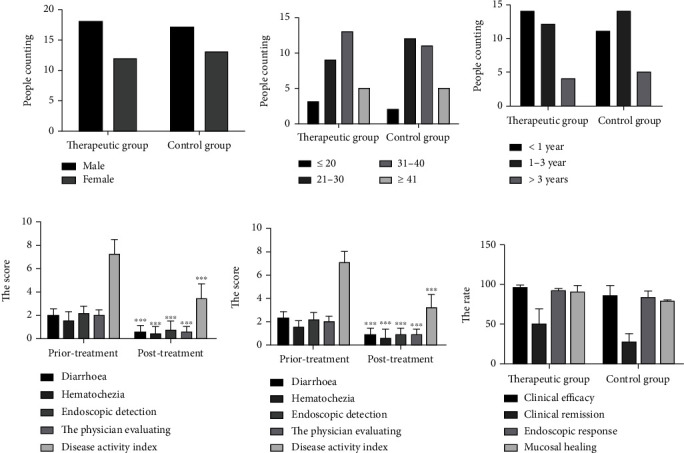
Qinghua Jianpi Recipe has a significant clinical effect on ulcerative colitis. The figure shows the gender (a), age (b), and disease duration (c) of the enrolled patients. The therapeutic group and control group patients, in the application of Qinghua Jianpi Recipe before (e) and after (d) treatment, get diarrhea, hematochezia, endoscopic detection, physician evaluation, and disease activity score. The clinical effective rate, clinical remission rate, endoscopic response rate, and experience healing rate of the therapeutic group and the control group were analyzed (f). ^∗∗∗^*P* < 0.001, the difference was statistically significant.

A total of 60 patients with UC were included, including 35 males and 25 females. Patients were randomly divided into 2 groups according to the order of visit. There were 30 cases in the therapeutic group and 30 cases in the control group. Pearson's chi-square test showed that there was no significant difference in age composition between the two groups, so the influence of gender could be excluded (Figure 1(a)). Among the 60 patients with UC, the age distribution was between 18 and 55 years old. The rank-sum test was used to analyze the age of the two groups, and the results were not statistically significant. Therefore, the influence of age factor was excluded, and the two groups could be compared (Figure 1(b)). In the therapeutic group, the shortest disease duration was 3 months and the longest disease duration was 41 months. The rank-sum test was also applied to rank variables of the two groups to exclude the influence of disease duration factors, indicating that the two groups could be compared (Figure 1(c)).

Paired sample *t*-test was used for pairwise comparison of individual items and overall mean scores before and after treatment in the two groups, and *P* values were much less than 0.05, indicating significant statistical differences. The results showed that the Qinghua Jianpi Recipe therapeutic group and mesalazine treatment control group were effective in treating the clinical manifestations of UC patients in terms of diarrhea, hematochezia, endoscopic manifestations (including Baron endoscopic grading score), physician evaluation, and activity index (Figures 1(d) and 1(e)). There was no significant difference in clinical efficacy, endoscopic response rate, and mucosal healing rate between the two groups after treatment (*P* > 0.05). There is no significant difference in the clinical effective rate, clinical remission rate, endoscopic response rate, and mucosal healing rate between the therapeutic group and the control group (Figure 1(f)). It indicates that the Qinghua Jianpi Recipe treatment group has achieved the same effect as the mesalazine treatment group in the single disease evaluation index.

No special discomfort or other serious adverse reactions were reported in the two groups. After 8 weeks of treatment, the electrocardiogram and liver and kidney function of all patients were reviewed and compared, and no obvious abnormalities were found, indicating that the Qinghua Jianpi Recipe group and mesalazine have greater safety in the treatment of ulcerative colitis patients.

### 3.2. Effect of Qinghua Jianpi Recipe on the Changes of Signs of Colorectal Adenoma Model Mice

**Figure 2:**
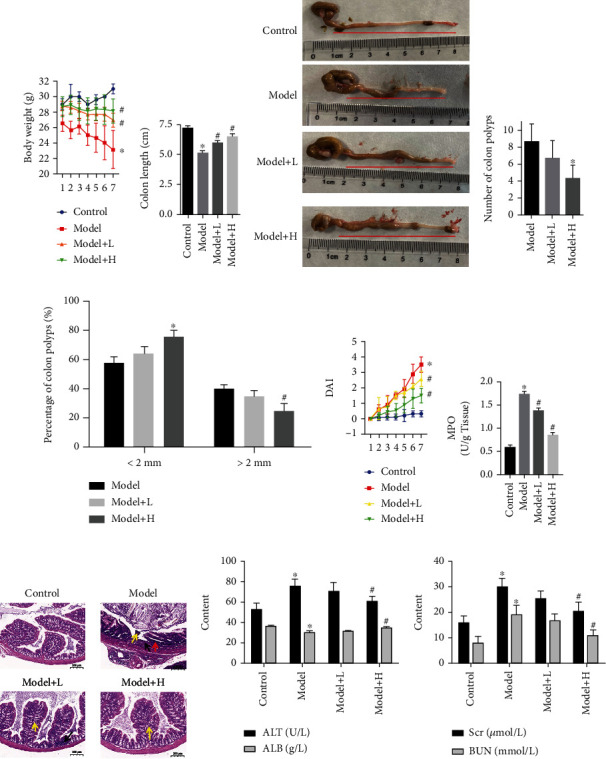
Effect of Qinghua Jianpi Recipe on intestinal inflammation, tumorigenesis, and pathological changes. (a) Body weight change in mice in seven weeks; (b, c) colon length of mice in each group and statistical results; (d) the number of intestinal tumor formation in adenoma model mice; (e) the size of intestinal tumor formation in adenoma model mice (<2 mm or >2 mm); (f) DAI score of rats in each group; (g) levels of MPO of intestinal tissues in each group; (h) HE staining of intestinal tissues. (i) Liver and kidney function indexes were analyzed in each group. ^∗^*P* < 0.05 and ^#^*P* < 0.05, the difference was statistically significant.

Before the experiment, mice in each group had normal development, bright hair color, good spirit, active activity, normal feeding, and granular stool. During the first DSS cycle, the general situation of blank group is normal activity. But mice activity of other groups decreased, a series of reactions occuring simultaneously including deformed feces, looking glazed, food intake reduction. However, change of ordinary drinking water can alleviate the symptoms. After 2 to 4 weeks' time DSS loop, the blank group is in good condition. And the other groups mice have the color dark and dry coat, depressed, and tired. They have diarrhea, bloody stools, and weight gain after replacement of ordinary water. In subsequent experiments, the general situation of mice in the blank group remained normal all time, while the situation of mice in the model group gradually intensified with the increase of subsequent DSS circulation, presenting dry hair, emaciation, perianal redness and swelling, anal prolapse, and large amount of bloody stools. Compared with the model group, the Qinghua Jianpi Recipe treatment group has improved hair gloss, mental state, and relatively increased physical activity. Similarly, diarrhea and blood stool symptoms were relieved. Experiments were made to start each group about the same weight. Giving DSS cycle period, except the blank group of mice, each weight was significantly decreased in mice. After the treatment with Qinghua Jianpi Recipe, compared with the model group, the low- and high-dose group mouse body weights have a rising trend (Figure 2(a)).

### 3.3. Qinghua Jianpi Recipe Significantly Increased Colon Length and Decreased DAI Score in Colorectal Adenoma Model Mice

Colon lengths reflect the degree of intestinal inflammatory response in mice with colitis to a certain extent [14]. Therefore, to clarify the inhibitory effect of Qinghua Jianpi Recipe on intestinal inflammatory activity in the mice with adenoma cancer model, the colon length of mice in each group was observed. In this study, we found that, compared with the model group, Qinghua Jianpi Recipe significantly increased the length of colon in mice, while reducing DAI score (Figures 2(b), 2(c), and 2(f)). This means that the intervention of Qinghua Jianpi Recipe can effectively inhibit the systemic inflammatory response, thus helping to improve the prognosis of patients with high levels of inflammation such as IBD.

### 3.4. Effect of Qinghua Jianpi Recipe on the Number and Size of Intestinal Tumor Formation in Adenoma Model Mice

The mice were dissected after death in each group, and the intestinal tubes were cut longitudinally and laid flat on the plate. The intestinal conditions of mice and the location, number, and size of tumors were observed by naked eye and recorded. The results showed that the colon of mice in the blank group was soft in texture, and the intestinal mucosa was smooth and flat, without hyperemia and edema or obvious hyperplasia. In the model group, the flexibility of the colon was decreased, the intestinal mucosa surface was rough, hyperemia and edema were obvious, hemorrhages were visible locally, and multiple bulges were observed in the mucosa of the middle and lower segment of the colon and near the anus, with the maximum diameter of 5 mm, and the number of tumors was 10.75 + 2.98. Compared with the model group, mice in the Qinghua Jianpi Recipe low-dose group had less colorectal hyperemia and edema, and mucosal neoplasm was mostly in the lower segment of the colorectum, with the number of tumors being 7.75 + 1.99, which was statistically different from that in the model group. The intestinal condition of the Qinghua Jianpi Recipe high-dose group was similar to that of the low-dose group, and the number of tumors was 5 + 1.91, which was significantly different from that of the model group. According to the tumor volume analysis, 41.9% of the mice in the model group had tumors larger than 2 mm in diameter, while 25.4% of the mice in the Qinghua Jianpi Recipe high-dose group had tumors larger than 2 mm in diameter, showing a significant difference compared with the model group. In summary, it indicates that Qinghua Jianpi Recipe can alleviate the intestinal injury induced by the molding agent and inhibit the number and size of intestinal adenoma formation (Figures 2(d) and 2(e)).

### 3.5. Qinghua Jianpi Recipe Significantly Improved the Colorectal Pathological Changes in Colorectal Adenoma Model Mice

After paraffin embedding, sectioning, and HE staining, the nucleus was purplish blue, and the cytoplasm was red. The pathological conditions of tissues and cells were determined by staining. HE staining results of intestinal tissues of mice in each group showed that the colon glands in the blank group were arranged regularly, with flat or cubic nuclei at the base and mild morphology, and a few lymphocytes infiltrated in the interstitium. In the model group, the epithelial cells showed no polarity and fused with each other, presenting nests and mesopores. Necrosis was observed in the center, the epithelial mucus layer disappeared, the nucleoplasma ratio increased, the nuclei were stained deeply, nucleoli were obvious, and mitosis was easy to see. In the Qinghua Jianpi Recipe low-dose group, goblet cells disappeared, proliferation, crowding, columnar or pseudostratified structure, thick nuclear chromatin, small nucleoli, and increased mitosis in colonic epithelial cells were noted. In the high-dose Qinghua Jianpi Recipe group, the gland arrangement was orderly, the number of goblet cells decreased, some crypts and epithelial mucus layer disappeared, epithelial nuclei slightly enlarged, small nucleoli were seen, and mitosis was occasionally seen. It was found that Qinghua Jianpi Recipe intervention could significantly reduce the incidence of adenoma in CRC and the state of colon inflammation, significantly improve the pathological damage of the colon in adenoma canceration model mice, and reduce inflammatory cell infiltration (Figure 2(h)). At the same time, we observed that the expression level of myeloperoxidase (MPO) in colon tissue could be significantly reduced by Qinghua Jianpi Recipe intervention compared with the adenoma cancer model mice (Figure 2(g)). We also found that the corresponding modeling reagents produced toxic effects on the liver and kidney of mice. However, Qinghua Jianpi Recipe plays the role of reducing the toxicity and increasing the effect (Figure 2(i)). It was not toxic to the liver or kidney at the doses in this study.

### 3.6. Qinghua Jianpi Recipe Significantly Improved the Intestinal Microflora Structure of Colorectal Adenoma Canceration Model Mice

**Figure 3:**
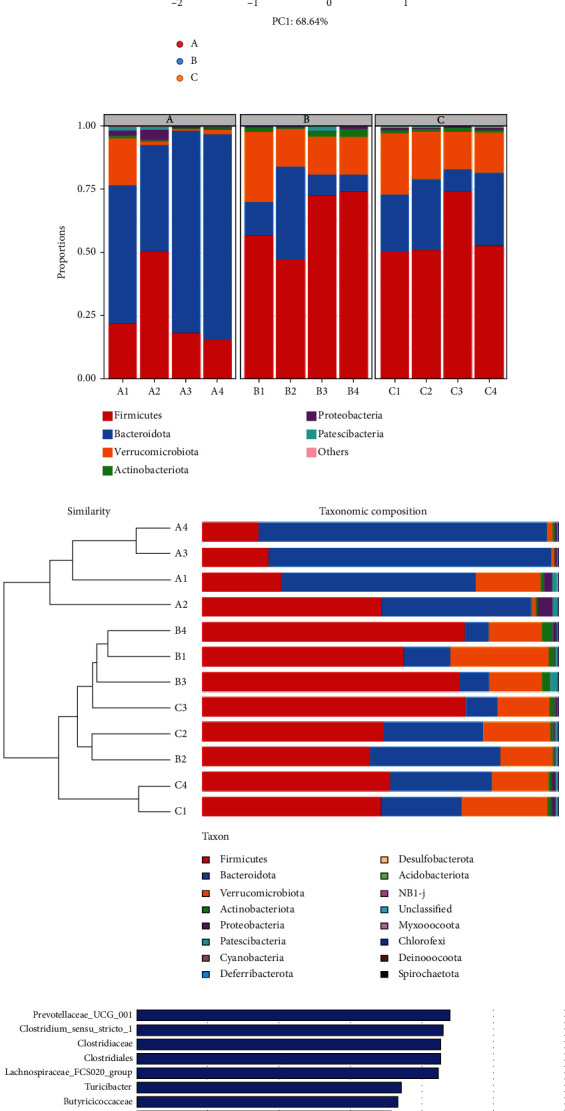
Effect of Qinghua Jianpi Recipe on the intestinal microflora structure of colorectal adenoma canceration model mice. (a) Rarefaction curve, the depth of sample sequencing can be obtained by making dilution curve; (b) alpha-diversity, the abundance and diversity of microbial communities can be reflected by single-sample diversity analysis (alpha diversity), including Simpson, Shannon, and Chao indexes; (c) beta diversity analysis, used to study similarities or differences in the composition of sample communities; (d, e) diversity analysis of intestinal microbiota of rats in each group; (F) LEfSe analysis, the figure shows the LDA scores obtained by LDA (linear regression analysis) for the microorganisms with significant effects in the two groups.

With the increase of intestinal flora 16S RAN sequencing depth, the trend of species increase slowed down, indicating that this sequencing has covered the dominant flora in the sample, suggesting that the sequencing data of this time is qualified (Figure 3(a)). Compared with the normal control group, the Simpson index of the model group has an increasing trend, while that in the Qinghua Jianpi Recipe intervention group had a similar trend. This suggests that the modeling of the colorectal adenoma canceration model reduced the diversity of intestinal flora, while the Qinghua Jianpi Recipe intervention restored the diversity of intestinal flora. Compared with the normal control group, the Shannon and Chaos indexes of the model group showed a decreasing trend. This suggests that the diversity of intestinal flora was reduced after modeling, while the Shannon and Chaos indexes of the Qinghua Jianpi Recipe intervention group showed a rising trend compared with the model group (Figure 3(b)). All the above results indicated that the intestinal microflora diversity of the Qinghua Jianpi Recipe intervention group was closer to that of the normal control group.

Beta diversity analysis (PCoA results) showed that the first principal component PCoA1 could explain 68.64% of the difference between groups, and the second principal component PCoA2 could explain 12.73% of the difference between groups. The distance between the normal control group, the model group, and Qinghua Jianpi Recipe intervention group was far (Figure 3(c)).

Analysis of the intestinal flora structure revealed that at the phylum level, the top 6 phyla in abundance were Firmicutes, Bacteroidota, Verrucomicrobiota, Actinobacteriota, Proteobacteria, and Patescibacteria. In terms of the composition and structure of bacteria, the model group and the Qinghua Jianpi Recipe intervention group were similar. However, on the whole, the dominant bacteria in the three groups were Firmicutes, Bacteroidota, and Verrucomicrobiota. At the genus level, the top 10 genera in relative abundance were Muribaculaceae_norank, Lactobacillus, Akkermansia, Clostridia UCG−014_norank, Dubosiella and Prevotellaceae UCG−001, Lachnospiraceae_uncultured, Clostridium sensu stricto 1, Lachnospiraceae NK4A136 group, and Ligilactobacillus. The three groups have significantly different structural changes at the genus level. The model group and the Qinghua Jianpi Recipe intervention group have relatively high Lactobacillus and Akkermansia, while the normal control group has relatively high Muribaculaceae_norank (Figures 3(d) and 3(e)).

LEfSe analysis was performed on fecal microbiome sequence data to explore the potential biomarker flora of related organisms. The results showed that after modeling, at the genus level, the levels of Coriobacteriia, Parvibacter, Limosilactobacillus, Dubosiella, Lactobacillus, Bacilli, and UCG_010 were significantly increased. The prognosis of Qinghua Jianpi Recipe was significantly increased levels of the genera Peptostreptococcales_Tissierellales, NK4A214_group, Romboutsia, Peptostreptococcaceae (Streptococcus mutans), Turicibacter (genus), Lachnospiraceae_FCS020_group, Clostridium_sensu_stricto_1, and Prevotellaceae_UCG_001. Compared with the model group and Qinghua Jianpi Recipe intervention group, Bacteroidota (Bacteroidetes) was specific in the phylum level in the normal control group. At the genus level, the specific bacterial communities include Peptococcales, Roseburia (Roche), Muribaculum (genus), Staphylococcus (Staphylococcus), Family_XIII_AD3011_group, ASF356, Alloprevotella, and Prevotellaceae_NK3B31_group (Figure 3(f)).

### 3.7. Qinghua Jianpi Recipe Can Regulate Short-Chain Fatty Acid Metabolism of Intestinal Bacteria in Mice with Colorectal Adenoma Cancer Model

**Figure 4:**
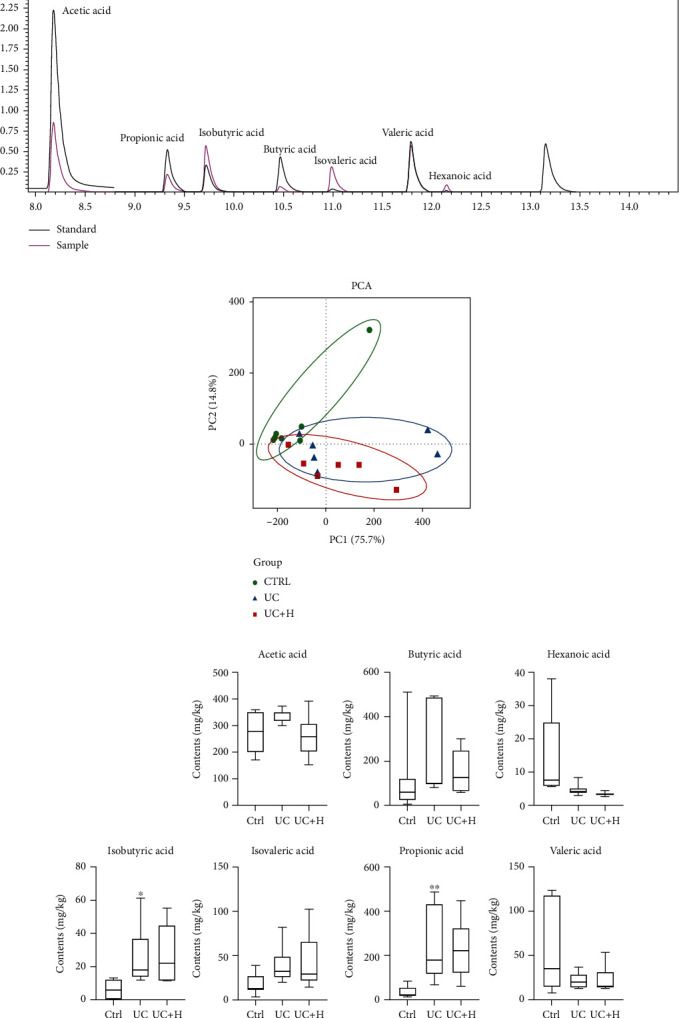
Qinghua Jianpi Recipe can regulate short-chain fatty acid metabolism of intestinal bacteria in mice with colorectal adenoma cancer model. The TIC chromatograms (a), PCA (b) and concentration (c) of the short-chain fatty acids in fecal samples. ^∗^*P* < 0.05 and ^∗∗^*P* < 0.05, the difference was statistically significant.

In this study, acetic acid, propionic acid, isobutyric acid, butyric acid, isovaleric acid, valeric acid, and caproic acid in feces were analyzed based on GC-MS. It was found that the content of short-chain fatty acids in the model group was significantly changed, especially isobutyric acid and propionic acid (Figures 4(a) and 4(c)). It was also found that the Qinghua Jianpi Recipe intervention group could reverse the changes of short-chain fatty acids (Figures 4(a) and 4(c)). PCA based on the determination results of 7 kinds of short-chain fatty acids showed that the normal group, the model group, and the drug treatment group could be clearly distinguished. This indicated that the content of short-chain fatty acids among the three groups had significant overall differences (Figure 4(b)).

### 3.8. Network Pharmacology Analysis Found That Qinghua Jianpi Recipe May Inhibit Inflammatory Cancer Transformation of the Station and the Occurrence and Development of Colorectal Tumor through Regulatory Pathways

**Figure 5:**
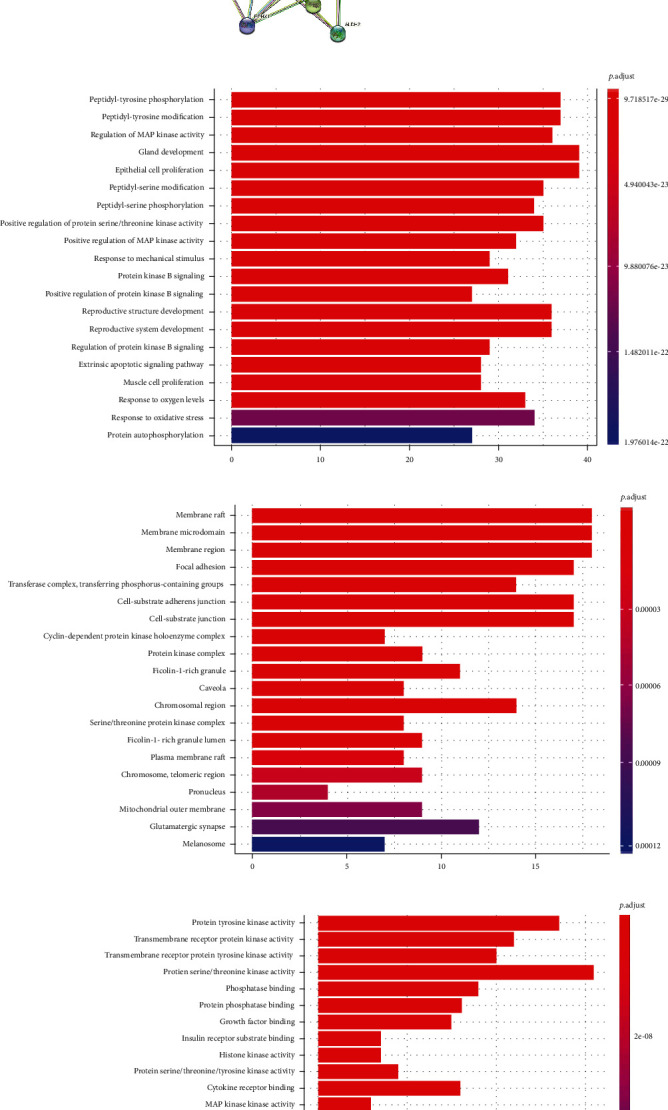
Network pharmacology analysis of the molecular mechanism of Qinghua Jianpi Recipe in preventing the transformation process of inflammatory cancer. Network diagram of drug-component-target-disease interactions (a); PPI network of protein interaction (b); the biological processes, cell components, and molecular functions of 137 common targets were selected by GO analysis after R language operation (c–e). The first 20 results formed a bar graph (f) of KEGG functional enrichment, with *P* value representing the significance of enrichment, and the redder the color, the higher the significance.

OB ≥ 30% and DL ≥ 0.18 were set in the TCMSP database to screen the effective components of Atractylodes macrocephala, Tangerine peel, Radix Paeoniae, Codonopsis pilosula, parsnip, Poria cocos, Glycyrrhiza, Astragalus membranaceus, Scutellaria baicalensis, Achyranthes bidentata, and coix seed. A total of 236 potential active components were obtained. 1036 drug targets were screened out using the Swiss Target Prediction database. Based on the keywords “colorectal carcinoma”, we searched OMIM, DisGeNet, and GeneCards databases and obtained 1011 disease targets. Venny2.1 online software mapping tool platform was used to input 1036 drug targets and 1011 disease targets to draw a Venn diagram, and 137 drug-disease common targets were obtained after the intersection of the two. The 236 potential active components and 137 drug-disease common targets in TCM compounds were input into Cytoscape software, and isolated components without intersection with targets were deleted to draw the network diagram of “drug-component-target-disease” interaction (Figures 5(a) and 5(b) and Supplementary materials 1-2).

The above 137 common targets were input into the STRING database for retrieval. The protein type was set as “Homo sapiens,” and the minimum interaction threshold was 0.4. The network relationship data of the target interaction was obtained and imported into Cytoscape software. Protein interaction network diagram was drawn. The biological process, cell components, and molecular functions of 137 common targets were selected by GO analysis after R language operation. GO results showed that the set of intersected genes was enriched into 2435 biological process pathways. It mainly includes regulation of MAP kinase activity, response to oxidative stress, and epithelial cell proliferation. The intersection gene sets were enriched with 59 cell components, which mainly involved cell-substrate adherens junctions, membrane region, and so on. The set of intersected genes was enriched with 129 genes related to molecular function. There are mainly protein serine/threonine kinase activity, protein tyrosine kinase activity, etc. (Figures 5(c)–5(e) and Supplementary materials 3-8).

151 KEGG pathways were obtained by running 137 common targets in R language, and the first 20 results formed a bar graph with KEGG enrichment. The results showed that Qinghua Jianpi Recipe may inhibit intestinal inflammation and the process of “inflammatory cancer transformation” through multiple pathways and multiple targets, mainly through regulating PI3K-Akt, JAK-Stat, nod-like receptor, and other signaling pathways (Figure 5(f) and Supplementary materials 9).

### 3.9. Qinghua Jianpi Recipe May Regulate the Expression of Pathway-Related Proteins and Inhibit the Expression of Inflammatory Factors in Intestinal Tissue

**Figure 6:**
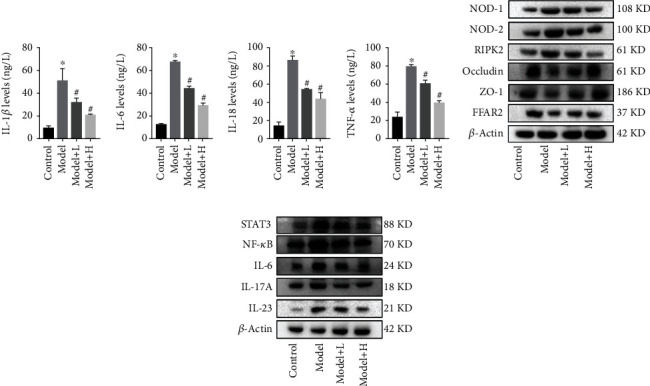
Qinghua Jianpi Recipe regulated the expression of pathway-related proteins and inhibited the expression of inflammatory factors in intestinal tissue. ELISA was used in testing the expression of inflammatory factors in intestinal tissue (a); Western blot analysis was used to detect related protein (*x* ± *s*, *n* ≥ 3) (b, c). *β*-Actin was used as an invariant control for equal loading. ^∗^*P* < 0.05 and ^#^*P* < 0.05, the difference was statistically significant.

Based on the network pharmacology analysis and related experimental results, we further confirmed the specific molecular mechanism of Qinghua Jianpi Recipe inhibiting intestinal inflammation and “inflammatory cancer transformation.” First, Qinghua Jianpi Recipe could inhibit the expression of inflammatory factors in colon tissues, including IL-1*β*, IL-6, IL-18, and TNF-*α* (Figure 6(a)). In the inflammatory state, intestinal barrier function was impaired, and the expression of intestinal barrier function-related proteins claudin-1, Occludin, and ZO-1 decreased. And the expression of the above proteins increased in the Qinghua Jianpi Recipe intervention group (Figure 6(b)). In the inflammatory state, the expression of pattern recognition receptor-related genes Nod1, Nod2, and Ripk2 increased, initiating the immune response; inflammatory factors NF-*κ*B and STAT3 and inflammatory factors IL-6, IL-23, and IL-17A increased. The expression of the above proteins decreased in the Qinghua Jianpi Recipe intervention group (Figures 6(b) and 6(c)). Intestinal flora is involved in many links of intestinal inflammation, among which metabolites play an important role. In the inflammatory state, the expression of free fatty acid receptor 2 (FFAR2) is decreased, and the protein expression is increased after the treatment of Qinghua Jianpi Recipe (Figure 6(b)).

## 4. Discussion

CRC is a disease with high morbidity and mortality due to the interaction of multiple factors, such as genes and environment, and its specific mechanism is unknown. CRC with genetic predisposition accounts for only a small proportion, including familial adenomatous polyposis (FAP), hereditary nonpolyposis colorectal cancer (HNPCC), and hamartomatous polyposis syndrome. Among them, chronic inflammation is the main environmental factor for the occurrence and development of CRC. Studies have shown that patients with Crohn's disease (CD) and UC have a higher risk of developing CRC [15, 16].

In this study, our goal was to confirm the inhibitory effect of Qinghua Jianpi Recipe on the “transformation” of inflammatory carcinoma. We first confirmed the efficacy of Qinghua Jianpi Recipe in improving colonic inflammatory status and function in patients with UC. And then, the influence of Qinghua Jianpi Recipe on adenoma canceration model mouse was observed, including intestinal inflammation state, number of adenoma, and pathological changes. Qinghua Jianpi Recipe can significantly improve the intestinal inflammatory state of adenoma cancer model mice, reduce the number of 1-3 mm adenoma, reduce intestinal inflammatory activity and pathological intestinal damage, and reduce the expression of proinflammatory cytokine MPO in colon tissue. Previous studies and network pharmacology studies have confirmed that the occurrence of colon polyps and colorectal tumors is closely related to intestinal barrier function, intestinal flora metabolism, and intestinal inflammation [17, 18]. Increased tumorigenesis was observed in mice lacking free fatty acid receptor 2 (FFAR2), which is considered due to reduced intestinal epithelial cell (IEC) integrity and an influx of bacteria [19]. We found that after modeling, at the genus level, the levels of Coriobacteriia, Parvibacter, Limosilactobacillus, Dubosiella, Lactobacillus, Bacilli, and UCG_010 were significantly increased. The prognosis of Qinghua Jianpi Recipe was significantly increased the genus level of Peptostreptococcales_Tissierellales, NK4A214_group, Romboutsia, Peptostreptococcaceae (Streptococcus mutans), Turicibacter, Lachnospiraceae_FCS020_group, Clostridium_sensu_stricto_1, and Prevotellaceae_UCG_001. The concentration of short-chain fatty acids in the feces of mice in each group was detected by GC-MS, and it was observed that the content of short-chain fatty acids in the model group was significantly changed, especially isobutyric acid and propionic acid, while the change of short-chain fatty acids in the Qinghua Jianpi Recipe group was reversed. Further experimental studies confirmed that intestinal barrier function-related proteins, inflammatory and immune-related signaling pathways (STAT3 and Nod pathways), and free fatty acid receptor 2 (FFAR2) inhibited the “inflammatory transformation” of colon cancer.

In this study, the effects of Qinghua Jianpi Recipe on preventing colon polyp recurrence and inhibiting the progress of “inflammatory cancer transformation” were observed. And the changes of the structure of intestinal flora and inflammatory (immune) microenvironment in mice with colon polyp were investigated by taking the intestinal flora as the core. However, there is still a lack of studies on the mutated genes in adenoma carcinoma sequence by Qinghua Jianpi Recipe, as well as the related signal pathways. It is believed that with the further development of Qinghua Jianpi Recipe, its mechanism of preventing adenoma canceration will be further clarified. That will provide a new strategy for preventing precancerous lesions of CRC. The research results of this project will further supplement the occurrence mechanism of colon polyps and CRC, provide an objective basis for TCM to prevent polyp recurrence and cancer, and lay a scientific foundation for the development of new TCM to prevent colorectal polyp recurrence and cancer.

## Figures and Tables

**Table 1:** The modified Mayo index.

Project	The score			
	0 points	1 points	2 points	3 points
Diarrhea	Normal	Over the normal period for 1 to 2 times/d	Over the normal period for 3 to 4 times/d	More than normal 5 times/d or more
Hematochezia	No bleeding	Less than half of the time for the blood mixed in the excrement	Most of the time for the blood mixed in the excrement	There has always been the blood mixed in the excrement
Endoscopic detection	Normal or no active lesions occurred	Mild lesions (reduced vascular texture of colon mucosa, visible mild erosion)	Moderate lesions (moderate mucosal erosion)	Severe lesions (spontaneous bleeding, scattered dotted ulcers)
The physician evaluation	Normal	Mild lesions	Moderate lesion	Severe lesions

During the quantification of the score, each subject was compared with himself to judge the grade of stool frequency. The hematochezia score represents the most severe hematochezia in one day. The overall evaluation included three criteria: the subjects' feeling of abdominal discomfort, the degree of impact of the disease on their life and work, and other manifestations.

## Data Availability

We can provide additional methods and data if needed.

## Supplementary Materials

Supplementary Materials The analysis data of the network pharmacology. Active ingredients in traditional Chinese medicine (1); 1011 targets in colorectal cancer (2); PPI topological analysis (3); topological analysis of 213 active components in the network diagram (4); MCODE analysis (5); biological processes (BP, GO enrichment analysis) (6); cell components (CC, GO enrichment analysis) (7); molecular function (MF, GO enrichment analysis) (8); KEGG analysis (9).
