# Supplementary material for: The Efficacy and Mechanism of Qinghua Jianpi Recipe in Inhibiting Canceration of Colorectal Adenoma Based on Inflammatory Cancer Transformation
Source: J Immunol Res. 2023 Feb 15;2023:4319551. doi: 10.1155/2023/4319551 (PMC9946765; doi:10.1155/2023/4319551)
Supplement: Supplementary Materials — The analysis data of the network pharmacology. Active ingredients in traditional Chinese medicine (1); 1011 targets in colorectal cancer (2); PPI topological analysis (3); topological analysis of 213 active components in the network diagram (4); MCODE analysis (5); biological processes (BP, GO enrichment analysis) (6); cell components (CC, GO enrichment analysis) (7); molecular function (MF, GO enrichment analysis) (8); KEGG analysis (9). [file 4319551.f1.zip › Active ingredients in traditional Chinese medicine.pdf]

编号	活性成分	OB（%）	DL值
MOL000020	12-senecioyl-2E,8E,10E-atractylentr	62.4	0.22
MOL000021	14-acetyl-12-senecioyl-2E,8E,10E-at	60.31	0.31
MOL000022	14-acetyl-12-senecioyl-2E,8Z,10E-at	63.37	0.3
MOL000028	alpha-Amyrin	39.51	0.76
MOL000033	(3S,8S,9S,10R,13R,14S,17R)-10,13-di	36.23	0.78
MOL000049	3beta-acetoxyatractylone	54.07	0.22
MOL000072	8beta-ethoxy atractylenolide III	35.95	0.21
MOL000359	sitosterol	36.91	0.75
MOL004328	naringenin	59.29	0.21
MOL005100	5,7-dihydroxy-2-(3-hydroxy-4-methox	47.74	0.27
MOL005815	Citromitin	86.9	0.51
MOL005828	nobiletin	61.67	0.52
MOL001002	ellagic acid	43.06	0.43
MOL001918	paeoniflorgenone	87.59	0.37
MOL001921	Lactiflorin	49.12	0.8
MOL001924	paeoniflorin	53.87	0.79
MOL001925	paeoniflorin_qt	68.18	0.4
MOL002714	baicalein	33.52	0.21
MOL002776	Baicalin	40.12	0.75
MOL000358	beta-sitosterol	36.91	0.75
MOL004355	Spinasterol	42.98	0.76
MOL000449	Stigmasterol	43.83	0.76
MOL000492	(+)-catechin	54.83	0.24
MOL006990	(1S,2S,4R)-trans-2-hydroxy-1,8-cine	30.25	0.27
MOL006992	(2R,3R)-4-methoxyl-distylin	59.98	0.3
MOL006994	1-o-beta-d-glucopyranosyl-8-o-benzo	36.01	0.3
MOL006996	1-o-beta-d-glucopyranosylpaeonisuff	65.08	0.35
MOL006999	stigmast-7-en-3-ol	37.42	0.75
MOL007003	benzoyl paeoniflorin	31.14	0.54
MOL007004	Albiflorin	30.25	0.77
MOL007005	Albiflorin_qt	48.7	0.33
MOL007008	4-ethyl-paeoniflorin_qt	56.87	0.44
MOL007012	4-o-methyl-paeoniflorin_qt	56.7	0.43
MOL007014	8-debenzoylpaeonidanin	31.74	0.45
MOL007016	Paeoniflorigenone	65.33	0.37
MOL007018	9-ethyl-neo-paeoniaflorin A_qt	64.42	0.3
MOL007022	evofolinB	64.74	0.22
MOL007025	isobenzoylpaeoniflorin	31.14	0.54
MOL002883	Ethyl oleate (NF)	32.4	0.19
MOL005043	campest-5-en-3beta-ol	37.58	0.71
MOL001006	poriferasta-7,22E-dien-3beta-ol	42.98	0.76
MOL002140	Perlolyrine	65.95	0.27
MOL002879	Diop	43.59	0.39
MOL003036	ZINC03978781	43.83	0.76
MOL003896	7-Methoxy-2-methyl isoflavone	42.56	0.2
MOL004492	Chrysanthemaxanthin	38.72	0.58

MOL005321	Frutinone A	65.9	0.34
MOL000006	luteolin	36.16	0.25
MOL006554	Taraxerol	38.4	0.77
MOL006774	stigmast-7-enol	37.42	0.75
MOL007059	3-beta-Hydroxymethyllenetanshiquino	32.16	0.41
MOL007514	methyl icosa-11,14-dienoate	39.67	0.23
MOL008391	5alpha-Stigmastan-3,6-dione	33.12	0.79
MOL008393	7-(beta-Xylosyl)cephalomannine_qt	38.33	0.29
MOL008397	Daturilin	50.37	0.77
MOL008400	glycitein	50.48	0.24
MOL008406	Spinoside A	39.97	0.4
MOL008407	(8S,9S,10R,13R,14S,17R)-17-[(E,2R,5	45.4	0.76
MOL008411	11-Hydroxyrankinidine	40	0.66
MOL000011	(2R,3R)-3-(4-hydroxy-3-methoxy-phen	68.83	0.66
MOL011730	11-hydroxy-sec-o-beta-d-glucosylham	50.24	0.27
MOL011732	anomalin	59.65	0.66
MOL011737	divaricatacid	87	0.32
MOL011740	divaricatol	31.65	0.38
MOL001941	Ammidin	34.55	0.22
MOL011747	ledebouriellol	32.05	0.51
MOL011749	phelloptorin	43.39	0.28
MOL011753	5-O-Methylvisamminol	37.99	0.25
MOL002644	Phellopterin	40.19	0.28
MOL000173	wogonin	30.68	0.23
MOL001494	Mandenol	42	0.19
MOL001942	isoimperatorin	45.46	0.23
MOL003588	Prangenidin	36.31	0.22
MOL013077	Decursin	39.27	0.38
MOL000273	(2R)-2-[(3S,5R,10S,13R,14R,16R,17R)-3,	30.93	0.81
MOL000275	trametenolic acid	38.71	0.8
MOL000276	7,9(11)-dehydropachymic acid	35.11	0.81
MOL000279	Cerevisterol	37.96	0.77
MOL000280	(2R)-2-[(3S,5R,10S,13R,14R,16R,17R)-3,	31.07	0.82
MOL000282	ergosta-7,22E-dien-3beta-ol	43.51	0.72
MOL000283	Ergosterol peroxide	40.36	0.81
MOL000285	(2R)-2-[(5R,10S,13R,14R,16R,17R)-16-hy	38.26	0.82
MOL000287	3beta-Hydroxy-24-methylene-8-lanostene-	38.7	0.81
MOL000289	pachymic acid	33.63	0.81
MOL000290	Poricoic acid A	30.61	0.76
MOL000291	Poricoic acid B	30.52	0.75
MOL000292	poricoic acid C	38.15	0.75
MOL000296	hederagenin	36.91	0.75
MOL000300	dehydroeburicoic acid	44.17	0.83
MOL001484	Inermine	75.18	0.54
MOL001792	DFV	32.76	0.18
MOL000211	Mairin	55.38	0.78
MOL002311	Glycyrol	90.78	0.67

MOL000239	Jaranol	50.83	0.29
MOL002565	Medicarpin	49.22	0.34
MOL000354	isorhamnetin	49.6	0.31
MOL003656	Lupiwighteone	51.64	0.37
MOL000392	formononetin	69.67	0.21
MOL000417	Calycosin	47.75	0.24
MOL000422	kaempferol	41.88	0.24
MOL004805	(2S)-2-[4-hydroxy-3-(3-methylbut-2-eny	31.79	0.72
MOL004806	euchrenone	30.29	0.57
MOL004808	glyasperin B	65.22	0.44
MOL004810	glyasperin F	75.84	0.54
MOL004811	Glyasperin C	45.56	0.4
MOL004814	Isotrifoliol	31.94	0.42
MOL004815	(E)-1-(2,4-dihydroxyphenyl)-3-(2,2-dim	39.62	0.35
MOL004820	kanzonols W	50.48	0.52
MOL004824	(2S)-6-(2,4-dihydroxyphenyl)-2-(2-hydr	60.25	0.63
MOL004827	Semilicoisoflavone B	48.78	0.55
MOL004828	Glepidotin A	44.72	0.35
MOL004829	Glepidotin B	64.46	0.34
MOL004833	Phaseolinisoflavan	32.01	0.45
MOL004835	Glypallichalcone	61.6	0.19
MOL004838	8-(6-hydroxy-2-benzofuranyl)-2,2-dimet	58.44	0.38
MOL004841	Licochalcone B	76.76	0.19
MOL004848	licochalcone G	49.25	0.32
MOL004849	3-(2,4-dihydroxyphenyl)-8-(1,1-dimethy	59.62	0.43
MOL004855	Licoricone	63.58	0.47
MOL004856	Gancaonin A	51.08	0.4
MOL004857	Gancaonin B	48.79	0.45
MOL004860	licorice glycoside E	32.89	0.27
MOL004863	3-(3,4-dihydroxyphenyl)-5,7-dihydroxy-	66.37	0.41
MOL004864	5,7-dihydroxy-3-(4-methoxyphenyl)-8-(3-	30.49	0.41
MOL004866	2-(3,4-dihydroxyphenyl)-5,7-dihydroxy-	44.15	0.41
MOL004879	Glycyrin	52.61	0.47
MOL004882	Licocoumarone	33.21	0.36
MOL004883	Licoisoflavone	41.61	0.42
MOL004884	Licoisoflavone B	38.93	0.55
MOL004885	licoisoflavanone	52.47	0.54
MOL004891	shinpterocarpin	80.3	0.73
MOL004898	(E)-3-[3,4-dihydroxy-5-(3-methylbut-2-	46.27	0.31
MOL004903	liquiritin	65.69	0.74
MOL004904	licopyranocoumarin	80.36	0.65
MOL004905	3,22-Dihydroxy-11-oxo-delta(12)-oleane	34.32	0.55
MOL004907	Glyzaglabrin	61.07	0.35
MOL004908	Glabridin	53.25	0.47
MOL004910	Glabranin	52.9	0.31
MOL004911	Glabrene	46.27	0.44
MOL004912	Glabrone	52.51	0.5

MOL004913	1,3-dihydroxy-9-methoxy-6-benzofurano[3	48.14	0.43
MOL004914	1,3-dihydroxy-8,9-dimethoxy-6-benzofura	62.9	0.53
MOL004915	Eurycarpin A	43.28	0.37
MOL004917	glycyroside	37.25	0.79
MOL004924	(-)-Medicocarpin	40.99	0.95
MOL004935	Sigmoidin-B	34.88	0.41
MOL004941	(2R)-7-hydroxy-2-(4-hydroxyphenyl)chro	71.12	0.18
MOL004945	(2S)-7-hydroxy-2-(4-hydroxyphenyl)-8-(3	36.57	0.32
MOL004948	Isoglycyrol	44.7	0.84
MOL004949	Isolicoflavonol	45.17	0.42
MOL004957	HMO	38.37	0.21
MOL004959	1-Methoxyphaseollidin	69.98	0.64
MOL004961	Quercetin der.	46.45	0.33
MOL004966	3'-Hydroxy-4'-O-Methylglabridin	43.71	0.57
MOL000497	licochalcone a	40.79	0.29
MOL004974	3'-Methoxyglabridin	46.16	0.57
MOL004978	2-[(3R)-8,8-dimethyl-3,4-dihydro-2H-py	36.21	0.52
MOL004980	Inflacoumarin A	39.71	0.33
MOL004985	icos-5-enoic acid	30.7	0.2
MOL004988	Kanzonol F	32.47	0.89
MOL004989	6-prenylated eriodictyol	39.22	0.41
MOL004990	7,2',4'-trihydroxy-5-methoxy-3-arylcou	83.71	0.27
MOL004991	7-Acetoxy-2-methylisoflavone	38.92	0.26
MOL004993	8-prenylated eriodictyol	53.79	0.4
MOL004996	gadelaidic acid	30.7	0.2
MOL000500	Vestitol	74.66	0.21
MOL005000	Gancaonin G	60.44	0.39
MOL005001	Gancaonin H	50.1	0.78
MOL005003	Licoagrocarpin	58.81	0.58
MOL005007	Glyasperins M	72.67	0.59
MOL005008	Glycyrrhiza flavonol A	41.28	0.6
MOL005012	Licoagroisoflavone	57.28	0.49
MOL005013	18alpha-hydroxyglycyrrhetic acid	41.16	0.71
MOL005016	Odoratin	49.95	0.3
MOL005017	Phaseol	78.77	0.58
MOL005018	Xambioona	54.85	0.87
MOL005020	dehydroglyasperins C	53.82	0.37
MOL000098	quercetin	46.43	0.28
MOL000371	3,9-di-O-methylnissolin	53.74	0.48
MOL000374	5'-hydroxyiso-muronulatol-2',5'-di-O-g	41.72	0.69
MOL000378	7-O-methylisomucronulatol	74.69	0.3
MOL000379	9,10-dimethoxypterocarpan-3-O-beta-D-g	36.74	0.92
MOL000380	(6aR,11aR)-9,10-dimethoxy-6a,11a-dihyd	64.26	0.42
MOL000387	Bifendate	31.1	0.67
MOL000398	isoflavanone	109.99	0.3
MOL000433	FA	68.96	0.71
MOL000438	(3R)-3-(2-hydroxy-3,4-dimethoxyphenyl)c	67.67	0.26

MOL000439	isomucronulatol-7,2'-di-O-glucosiole	49.28	0.62
MOL000442	1,7-Dihydroxy-3,9-dimethoxy pterocarpe	39.05	0.48
MOL001689	acacetin	34.97	0.24
MOL000228	(2R)-7-hydroxy-5-methoxy-2-phenylchroma	55.23	0.2
MOL002908	5,8,2'-Trihydroxy-7-methoxyflavone	37.01	0.27
MOL002909	5,7,2,5-tetrahydroxy-8,6-dimethoxyflav	33.82	0.45
MOL002910	Carthamidin	41.15	0.24
MOL002911	2,6,2',4'-tetrahydroxy-6'-methoxychalc	69.04	0.22
MOL002913	Dihydrobaicalin_qt	40.04	0.21
MOL002914	Eriodyctiol (flavanone)	41.35	0.24
MOL002915	Salvigenin	49.07	0.33
MOL002917	5,2',6'-Trihydroxy-7,8-dimethoxyflavon	45.05	0.33
MOL002925	5,7,2',6'-Tetrahydroxyflavone	37.01	0.24
MOL002926	dihydrooroxylin A	38.72	0.23
MOL002927	Skullcapflavone II	69.51	0.44
MOL002928	oroxylin a	41.37	0.23
MOL002932	Panicolin	76.26	0.29
MOL002933	5,7,4'-Trihydroxy-8-methoxyflavone	36.56	0.27
MOL002934	NEOBAICALEIN	104.34	0.44
MOL002937	DIHYDROOROXYLIN	66.06	0.23
MOL000525	Norwogonin	39.4	0.21
MOL000552	5,2'-Dihydroxy-6,7,8-trimethoxyflavone	31.71	0.35
MOL000073	ent-Epicatechin	48.96	0.24
MOL001458	coptisine	30.67	0.86
MOL001490	bis[(2S)-2-ethylhexyl] benzene-1,2-dic	43.59	0.35
MOL001506	Supraene	33.55	0.42
MOL002897	epiberberine	43.09	0.78
MOL008206	Moslosooflavone	44.09	0.25
MOL010415	11,13-Eicosadienoic acid, methyl ester	39.28	0.23
MOL012245	5,7,4'-trihydroxy-6-methoxyflavanone	36.63	0.27
MOL012246	5,7,4'-trihydroxy-8-methoxyflavanone	74.24	0.26
MOL012266	rivularin	37.94	0.37
MOL012461	28-norolean-17-en-3-ol	35.93	0.78
MOL012505	bidentatoside,ii_qt	31.76	0.59
MOL012537	Spinoside A	41.75	0.4
MOL012542	beta-ecdysterone	44.23	0.82
MOL001454	berberine	36.86	0.78
MOL002643	delta 7-stigmastenol	37.42	0.75
MOL003847	Inophyllum E	38.81	0.85
MOL000785	palmatine	64.6	0.65
MOL000085	beta-daucosterol_qt	36.91	0.75
MOL001040	(2R)-5,7-dihydroxy-2-(4-hydroxyphenyl)	42.36	0.21
MOL008601	Methyl arachidonate	46.9	0.23
MOL000953	CLR	37.87	0.68
MOL001323	Sitosterol alpha1	43.28	0.78
MOL002372	(6Z,10E,14E,18E)-2,6,10,15,19,23-hexam	33.55	0.42
MOL002882	[(2R)-2,3-dihydroxypropyl] (Z)-octadec	34.13	0.3

MOL008118	Coixenolide	32.4	0.43
MOL008121	2-Monoolein	34.23	0.29

Node1	Node2	Net
Colorectal carcinoma	CTSD	disease
Colorectal carcinoma	PGR	disease
Colorectal carcinoma	ESR2	disease
Colorectal carcinoma	CYP17A1	disease
Colorectal carcinoma	AR	disease
Colorectal carcinoma	NOS2	disease
Colorectal carcinoma	FLT1	disease
Colorectal carcinoma	RET	disease
Colorectal carcinoma	MDM2	disease
Colorectal carcinoma	PTGS2	disease
Colorectal carcinoma	GSK3B	disease
Colorectal carcinoma	KDR	disease
Colorectal carcinoma	ICAM1	disease
Colorectal carcinoma	ABCB1	disease
Colorectal carcinoma	EGFR	disease
Colorectal carcinoma	BCL2	disease
Colorectal carcinoma	MAPK1	disease
Colorectal carcinoma	PIK3CB	disease
Colorectal carcinoma	BUB1	disease
Colorectal carcinoma	CCNA2	disease
Colorectal carcinoma	MAPK14	disease
Colorectal carcinoma	AURKA	disease
Colorectal carcinoma	MMP1	disease
Colorectal carcinoma	MMP2	disease
Colorectal carcinoma	MET	disease
Colorectal carcinoma	ALK	disease
Colorectal carcinoma	TNF	disease
Colorectal carcinoma	MAP2K1	disease
Colorectal carcinoma	HSP90AA1	disease
Colorectal carcinoma	ERBB2	disease
Colorectal carcinoma	MMP9	disease
Colorectal carcinoma	MMP14	disease
Colorectal carcinoma	MMP7	disease
Colorectal carcinoma	NTRK1	disease
Colorectal carcinoma	STAT3	disease
Colorectal carcinoma	FGFR1	disease
Colorectal carcinoma	CDK2	disease
Colorectal carcinoma	ABCG2	disease
Colorectal carcinoma	CA7	disease
Colorectal carcinoma	ESR1	disease
Colorectal carcinoma	ALDH2	disease
Colorectal carcinoma	IL2	disease
Colorectal carcinoma	MCL1	disease
Colorectal carcinoma	RPS6KB1	disease
Colorectal carcinoma	JAK2	disease
Colorectal carcinoma	PRKCA	disease

Colorectal carcinoma	PPARG	disease
Colorectal carcinoma	TGFBR1	disease
Colorectal carcinoma	PIK3CA	disease
Colorectal carcinoma	PDGFRB	disease
Colorectal carcinoma	XIAP	disease
Colorectal carcinoma	MAPK8	disease
Colorectal carcinoma	CCND1	disease
Colorectal carcinoma	CDK4	disease
Colorectal carcinoma	SMO	disease
Colorectal carcinoma	CTSB	disease
Colorectal carcinoma	BCL2L1	disease
Colorectal carcinoma	EPHX1	disease
Colorectal carcinoma	NFKB1	disease
Colorectal carcinoma	CA9	disease
Colorectal carcinoma	NQO1	disease
Colorectal carcinoma	IGFBP3	disease
Colorectal carcinoma	PARP1	disease
Colorectal carcinoma	TOP1	disease
Colorectal carcinoma	ABCC1	disease
Colorectal carcinoma	CDK6	disease
Colorectal carcinoma	TERT	disease
Colorectal carcinoma	CDK1	disease
Colorectal carcinoma	DAPK1	disease
Colorectal carcinoma	IGF1R	disease
Colorectal carcinoma	PIK3R1	disease
Colorectal carcinoma	SRC	disease
Colorectal carcinoma	PTK2	disease
Colorectal carcinoma	MMP3	disease
Colorectal carcinoma	PLK1	disease
Colorectal carcinoma	AKT1	disease
Colorectal carcinoma	CCNB1	disease
Colorectal carcinoma	RAF1	disease
Colorectal carcinoma	IDH1	disease
Colorectal carcinoma	VDR	disease
Colorectal carcinoma	MAPK3	disease
Colorectal carcinoma	PTPN11	disease
Colorectal carcinoma	TOP2A	disease
Colorectal carcinoma	GLI1	disease
Colorectal carcinoma	JUN	disease
(8S,9S,10R,13R,14S,1	CTSD	target
Dangshen	(8S,9S,1C	mol
(8S,9S,10R,13R,14S,1	PGR	target
(8S,9S,10R,13R,14S,1	ESR2	target
(8S,9S,10R,13R,14S,1	CYP17A1	target
(8S,9S,10R,13R,14S,1	AR	target
(8S,9S,10R,13R,14S,1	NOS2	target
5alpha-Stigmastan-3,	AR	target

Dangshen	5alpha-St	mol
5alpha-Stigmastan-3,	CTSD	target
5alpha-Stigmastan-3,	NOS2	target
5alpha-Stigmastan-3,	FLT1	target
5alpha-Stigmastan-3,	RET	target
5alpha-Stigmastan-3,	MDM2	target
5alpha-Stigmastan-3,	PTGS2	target
5alpha-Stigmastan-3,	GSK3B	target
5alpha-Stigmastan-3,	KDR	target
5alpha-Stigmastan-3,	ICAM1	target
7-(beta-Xylosyl)ceph	ABCB1	target
Dangshen	7-(beta-X	mol
7-(beta-Xylosyl)ceph	EGFR	target
7-(beta-Xylosyl)ceph	BCL2	target
7-(beta-Xylosyl)ceph	MAPK1	target
7-(beta-Xylosyl)ceph	PIK3CB	target
7-(beta-Xylosyl)ceph	BUB1	target
7-(beta-Xylosyl)ceph	CCNA2	target
7-(beta-Xylosyl)ceph	MAPK14	target
7-(beta-Xylosyl)ceph	AURKA	target
7-(beta-Xylosyl)ceph	MMP1	target
7-(beta-Xylosyl)ceph	MMP2	target
7-(beta-Xylosyl)ceph	MET	target
7-(beta-Xylosyl)ceph	ALK	target
7-(beta-Xylosyl)ceph	TNF	target
7-(beta-Xylosyl)ceph	MAP2K1	target
7-(beta-Xylosyl)ceph	HSP90AA1	target
7-(beta-Xylosyl)ceph	ERBB2	target
7-(beta-Xylosyl)ceph	MMP9	target
7-(beta-Xylosyl)ceph	MMP14	target
7-(beta-Xylosyl)ceph	MMP7	target
7-(beta-Xylosyl)ceph	NTRK1	target
7-(beta-Xylosyl)ceph	STAT3	target
7-(beta-Xylosyl)ceph	KDR	target
7-(beta-Xylosyl)ceph	FGFR1	target
7-(beta-Xylosyl)ceph	CDK2	target
7-(beta-Xylosyl)ceph	CDK2	target
7-Methoxy-2-methyl i	ABCG2	target
Dangshen	7-Methoxy	mol
7-Methoxy-2-methyl i	CA7	target
7-Methoxy-2-methyl i	EGFR	target
7-Methoxy-2-methyl i	ABCB1	target
7-Methoxy-2-methyl i	ESR1	target
7-Methoxy-2-methyl i	ALDH2	target
7-Methoxy-2-methyl i	NTRK1	target
7-Methoxy-2-methyl i	ESR2	target
7-Methoxy-2-methyl i	IL2	target

7-Methoxy-2-methyl i	MCL1	target
7-Methoxy-2-methyl i	MMP1	target
7-Methoxy-2-methyl i	FLT1	target
7-Methoxy-2-methyl i	CYP17A1	target
7-Methoxy-2-methyl i	RPS6KB1	target
7-Methoxy-2-methyl i	AURKA	target
7-Methoxy-2-methyl i	JAK2	target
7-Methoxy-2-methyl i	PRKCA	target
7-Methoxy-2-methyl i	PPARG	target
7-Methoxy-2-methyl i	PGR	target
7-Methoxy-2-methyl i	TGFBR1	target
7-Methoxy-2-methyl i	PIK3CA	target
7-Methoxy-2-methyl i	PDGFRB	target
11-Hydroxyrankinidin	ERBB2	target
Dangshen	11-Hydrox	mol
11-Hydroxyrankinidin	EGFR	target
11-Hydroxyrankinidin	XIAP	target
11-Hydroxyrankinidin	NOS2	target
Chrysanthemaxanthin	MAPK8	target
Dangshen	Chrysanth	mol
Chrysanthemaxanthin	CCND1	target
Chrysanthemaxanthin	CDK4	target
Daturilin	PTGS2	target
Dangshen	Daturilin	mol
Daturilin	PGR	target
Daturilin	AR	target
Daturilin	SMO	target
Daturilin	MDM2	target
Daturilin	KDR	target
Daturilin	CDK2	target
Daturilin	CDK4	target
Daturilin	CYP17A1	target
Daturilin	CTSB	target
Daturilin	PDGFRB	target
Daturilin	BCL2L1	target
Daturilin	EPHX1	target
Daturilin	MAPK14	target
Diop	PRKCA	target
Dangshen	Diop	mol
Diop	AR	target
Frutinone A	NFKB1	target
Dangshen	Frutinone	mol
Frutinone A	CA7	target
Frutinone A	CA9	target
Frutinone A	ESR1	target
Frutinone A	ESR2	target
Frutinone A	NQO1	target

glycitein	EGFR	target
Dangshen	glyciteinmol	
glycitein	ESR1	target
glycitein	ESR2	target
glycitein	IL2	target
glycitein	CA7	target
glycitein	ABCB1	target
glycitein	ABCG2	target
glycitein	ALDH2	target
glycitein	MCL1	target
glycitein	IGFBP3	target
glycitein	HSP90AA1	target
luteolin	CA7	target
Dangshen	luteolin	mol
luteolin	GSK3B	target
luteolin	PARP1	target
luteolin	MMP9	target
luteolin	MMP2	target
luteolin	ABCG2	target
luteolin	TOP1	target
luteolin	ABCC1	target
luteolin	CDK6	target
luteolin	ABCB1	target
luteolin	ESR2	target
luteolin	ESR1	target
luteolin	PTGS2	target
luteolin	CDK2	target
luteolin	TERT	target
luteolin	CA9	target
luteolin	CDK1	target
luteolin	DAPK1	target
luteolin	IGF1R	target
luteolin	EGFR	target
luteolin	PIK3R1	target
luteolin	SRC	target
luteolin	PTK2	target
luteolin	KDR	target
luteolin	MMP3	target
luteolin	PLK1	target
luteolin	MET	target
luteolin	ALK	target
luteolin	AKT1	target
luteolin	AR	target
luteolin	CDK1	target
luteolin	CCNB1	target
methyl icosa-11,14-d	PPARG	target
Dangshen	methyl ic	mol

methyl icosa-11,14-d	PTGS2	target
methyl icosa-11,14-d	RAF1	target
methyl icosa-11,14-d	MCL1	target
methyl icosa-11,14-d	BCL2	target
methyl icosa-11,14-d	SMO	target
methyl icosa-11,14-d	IDH1	target
methyl icosa-11,14-d	EPHX1	target
poriferasta-7,22E-di	AR	target
Dangshen	poriferas	mol
poriferasta-7,22E-di	CYP17A1	target
poriferasta-7,22E-di	ESR1	target
poriferasta-7,22E-di	ESR2	target
poriferasta-7,22E-di	VDR	target
poriferasta-7,22E-di	NOS2	target
poriferasta-7,22E-di	PPARG	target
poriferasta-7,22E-di	TERT	target
poriferasta-7,22E-di	PGR	target
poriferasta-7,22E-di	MAPK3	target
poriferasta-7,22E-di	PTPN11	target
poriferasta-7,22E-di	TOP2A	target
Spinasterol	AR	target
Dangshen	Spinaster	mol
Spinasterol	CYP17A1	target
Spinasterol	ESR1	target
Spinasterol	ESR2	target
Spinasterol	VDR	target
Spinasterol	NOS2	target
Spinasterol	PPARG	target
Spinasterol	TERT	target
Spinasterol	MAPK3	target
Spinasterol	PTPN11	target
Spinasterol	TOP2A	target
Spinoside A	GLI1	target
Dangshen	Spinoside	mol
Spinoside A	JUN	target
stigmast-7-enol	AR	target
Dangshen	stigmast-	mol
stigmast-7-enol	CYP17A1	target
stigmast-7-enol	ESR1	target
stigmast-7-enol	ESR2	target
stigmast-7-enol	NOS2	target
stigmast-7-enol	VDR	target
stigmast-7-enol	PPARG	target
stigmast-7-enol	TERT	target
stigmast-7-enol	PGR	target
stigmast-7-enol	MAPK3	target
stigmast-7-enol	PTPN11	target

stigmast-7-enol	TOP2A	target
Stigmasterol	AR	target
Dangshen	Stigmaste	mol
Stigmasterol	CYP17A1	target
Stigmasterol	ESR1	target
Stigmasterol	ESR2	target
Stigmasterol	VDR	target
Stigmasterol	NOS2	target
Stigmasterol	PPARG	target
Taraxerol	AR	target
Dangshen	Taraxerol	mol
Taraxerol	ESR1	target
Taraxerol	ESR2	target
Taraxerol	CYP17A1	target
Taraxerol	TERT	target
Taraxerol	MAPK3	target
Taraxerol	PTPN11	target
Taraxerol	VDR	target
ZINC03978781	AR	target
Dangshen	ZINC03978	mol
ZINC03978781	CYP17A1	target
ZINC03978781	ESR1	target
ZINC03978781	ESR2	target
ZINC03978781	VDR	target
ZINC03978781	NOS2	target
ZINC03978781	MDM2	target

Node1	Node2	Net
Colorectal carcinoma	AR	disease
Colorectal carcinoma	ESR1	disease
Colorectal carcinoma	CYP17A1	disease
Colorectal carcinoma	ESR2	disease
Colorectal carcinoma	PPARG	disease
Colorectal carcinoma	TERT	disease
Colorectal carcinoma	MAPK3	disease
Colorectal carcinoma	PTPN11	disease
Colorectal carcinoma	CDK1	disease
Colorectal carcinoma	ABCB1	disease
Colorectal carcinoma	CA7	disease
Colorectal carcinoma	CDK6	disease
Colorectal carcinoma	CA9	disease
Colorectal carcinoma	ABCG2	disease
Colorectal carcinoma	PTGS2	disease
Colorectal carcinoma	GSK3B	disease
Colorectal carcinoma	ABCC1	disease
Colorectal carcinoma	PARP1	disease
Colorectal carcinoma	NOS2	disease
Colorectal carcinoma	MMP9	disease
Colorectal carcinoma	MMP2	disease
Colorectal carcinoma	TOP1	disease
Colorectal carcinoma	EGFR	disease
Colorectal carcinoma	SRC	disease
Colorectal carcinoma	CYP1A1	disease
Colorectal carcinoma	KIT	disease
Colorectal carcinoma	TOP2A	disease
Colorectal carcinoma	IGF1R	disease
Colorectal carcinoma	KDR	disease
Colorectal carcinoma	PLK1	disease
Colorectal carcinoma	MET	disease
Colorectal carcinoma	ALK	disease
Colorectal carcinoma	PLA2G2A	disease
Colorectal carcinoma	CCNB1	disease
Colorectal carcinoma	TNF	disease
Colorectal carcinoma	IL2	disease
Colorectal carcinoma	ALDH2	disease
Colorectal carcinoma	RPS6KB1	disease
Colorectal carcinoma	AURKA	disease
Colorectal carcinoma	RAC1	disease
Colorectal carcinoma	CHEK2	disease
Colorectal carcinoma	MAP2K1	disease
Colorectal carcinoma	PIK3CB	disease
Colorectal carcinoma	CHEK1	disease
Colorectal carcinoma	TYMS	disease
Colorectal carcinoma	JAK2	disease

Colorectal carcinoma	CDK2	disease
Colorectal carcinoma	CDK4	disease
Colorectal carcinoma	ICAM1	disease
Colorectal carcinoma	MAPK14	disease
Colorectal carcinoma	MAPK8	disease
Colorectal carcinoma	CCNA2	disease
Colorectal carcinoma	NTRK1	disease
Colorectal carcinoma	VDR	disease
Colorectal carcinoma	PGR	disease
Colorectal carcinoma	FLT1	disease
Colorectal carcinoma	EPHA2	disease
Colorectal carcinoma	MST1R	disease
Colorectal carcinoma	FGFR1	disease
Colorectal carcinoma	STAT3	disease
Colorectal carcinoma	TGFBR1	disease
Colorectal carcinoma	MDM2	disease
Colorectal carcinoma	CASP3	disease
Colorectal carcinoma	IDH1	disease
Colorectal carcinoma	EZH2	disease
Colorectal carcinoma	MMP3	disease
Colorectal carcinoma	MMP1	disease
Colorectal carcinoma	CTSB	disease
Colorectal carcinoma	BRAF	disease
Colorectal carcinoma	DAPK1	disease
Colorectal carcinoma	PIK3R1	disease
Colorectal carcinoma	PTK2	disease
Colorectal carcinoma	AKT1	disease
Colorectal carcinoma	RAF1	disease
Colorectal carcinoma	MTOR	disease
Colorectal carcinoma	TGFBR2	disease
Colorectal carcinoma	FGFR3	disease
Colorectal carcinoma	PDGFRB	disease
Colorectal carcinoma	FLT4	disease
Colorectal carcinoma	JAK1	disease
Colorectal carcinoma	GLI1	disease
Colorectal carcinoma	JUN	disease
Colorectal carcinoma	GRB2	disease
Colorectal carcinoma	MCL1	disease
Colorectal carcinoma	HSP90AA1	disease
Colorectal carcinoma	ODC1	disease
Colorectal carcinoma	PIK3CA	disease
Colorectal carcinoma	MAPK1	disease
Colorectal carcinoma	RELA	disease
28-norolean-17-en-3-o	AR	target
Niuxi	28-norole	mol
28-norolean-17-en-3-o	ESR1	target
28-norolean-17-en-3-o	CYP17A1	target

28-norolean-17-en-3-o	ESR2	target
28-norolean-17-en-3-o	PPARG	target
28-norolean-17-en-3-o	TERT	target
28-norolean-17-en-3-o	MAPK3	target
28-norolean-17-en-3-o	PTPN11	target
baicalein	CDK1	target
Niuxi	baicalein	mol
baicalein	ABCB1	target
baicalein	CA7	target
baicalein	CDK6	target
baicalein	CA9	target
baicalein	ABCG2	target
baicalein	ESR2	target
baicalein	ESR1	target
baicalein	PTGS2	target
baicalein	AR	target
baicalein	GSK3B	target
baicalein	ABCC1	target
baicalein	TERT	target
baicalein	PARP1	target
baicalein	NOS2	target
baicalein	MMP9	target
baicalein	MMP2	target
baicalein	TOP1	target
baicalein	EGFR	target
baicalein	SRC	target
baicalein	CYP1A1	target
baicalein	KIT	target
baicalein	TOP2A	target
baicalein	IGF1R	target
baicalein	KDR	target
baicalein	PLK1	target
baicalein	MET	target
baicalein	ALK	target
baicalein	MAPK3	target
baicalein	PLA2G2A	target
baicalein	CDK1	target
baicalein	CCNB1	target
Baicalin	TNF	target
Niuxi	Baicalin	mol
Baicalin	IL2	target
Baicalin	PTGS2	target
Baicalin	EGFR	target
Baicalin	ALDH2	target
berberine	RPS6KB1	target
Niuxi	berberine	mol
berberine	AURKA	target

berberine	RAC1	target
berberine	CHEK2	target
berberine	MAP2K1	target
berberine	PIK3CB	target
berberine	KIT	target
berberine	SRC	target
berberine	CHEK1	target
berberine	TYMS	target
berberine	PTGS2	target
berberine	JAK2	target
berberine	CDK2	target
berberine	CDK4	target
berberine	ICAM1	target
berberine	MAPK14	target
berberine	MET	target
berberine	MAPK8	target
berberine	CCNA2	target
berberine	NTRK1	target
berberine	CDK2	target
beta-daucosterol_qt	AR	target
Niuxi	beta-daucmol	
beta-daucosterol_qt	CYP17A1	target
beta-daucosterol_qt	ESR1	target
beta-daucosterol_qt	ESR2	target
beta-daucosterol_qt	VDR	target
beta-daucosterol_qt	NOS2	target
beta-daucosterol_qt	PPARG	target
beta-sitosterol	CYP17A1	target
Niuxi	beta-sitcmol	
beta-sitosterol	AR	target
beta-sitosterol	ESR1	target
beta-sitosterol	ESR2	target
beta-sitosterol	VDR	target
beta-sitosterol	NOS2	target
beta-sitosterol	PPARG	target
coptisine	RAC1	target
Niuxi	coptisinemol	
coptisine	CHEK2	target
delta 7-stigmastenol	AR	target
Niuxi	delta 7-smol	
delta 7-stigmastenol	CYP17A1	target
delta 7-stigmastenol	ESR1	target
delta 7-stigmastenol	ESR2	target
delta 7-stigmastenol	NOS2	target
delta 7-stigmastenol	VDR	target
delta 7-stigmastenol	PPARG	target
delta 7-stigmastenol	TERT	target

delta 7-stigmastenol	PGR	target
delta 7-stigmastenol	MAPK3	target
delta 7-stigmastenol	PTPN11	target
delta 7-stigmastenol	TOP2A	target
epiberberine	RAC1	target
Niuxi	epiberbermol	
epiberberine	RPS6KB1	target
epiberberine	AURKA	target
epiberberine	CHEK2	target
epiberberine	ICAM1	target
epiberberine	NTRK1	target
epiberberine	KIT	target
epiberberine	SRC	target
epiberberine	TYMS	target
epiberberine	PARP1	target
epiberberine	JAK2	target
epiberberine	PIK3CB	target
epiberberine	MAPK8	target
epiberberine	PTGS2	target
epiberberine	FLT1	target
epiberberine	EPHA2	target
epiberberine	MST1R	target
epiberberine	MET	target
epiberberine	FGFR1	target
epiberberine	STAT3	target
Inophyllum E	CA9	target
Niuxi	Inophyllumol	
Inophyllum E	CA7	target
Inophyllum E	GSK3B	target
Inophyllum E	MAPK14	target
Inophyllum E	TGFBR1	target
Inophyllum E	MDM2	target
Inophyllum E	PIK3CB	target
Inophyllum E	CASP3	target
Inophyllum E	EGFR	target
Inophyllum E	SRC	target
Inophyllum E	KDR	target
Inophyllum E	IDH1	target
Inophyllum E	EZH2	target
Inophyllum E	MAPK8	target
Inophyllum E	MET	target
Inophyllum E	MMP3	target
Inophyllum E	MMP9	target
Inophyllum E	MMP1	target
Inophyllum E	CTSB	target
Inophyllum E	BRAF	target
Inophyllum E	ALK	target

kaempferol	CA7	target
Niuxi	kaempfercmol	
kaempferol	ABCC1	target
kaempferol	ABCB1	target
kaempferol	ABCG2	target
kaempferol	GSK3B	target
kaempferol	MMP9	target
kaempferol	MMP2	target
kaempferol	ESR2	target
kaempferol	DAPK1	target
kaempferol	CDK6	target
kaempferol	CDK2	target
kaempferol	EGFR	target
kaempferol	IGF1R	target
kaempferol	PIK3R1	target
kaempferol	SRC	target
kaempferol	PTK2	target
kaempferol	KDR	target
kaempferol	MMP3	target
kaempferol	PLK1	target
kaempferol	CDK1	target
kaempferol	CA9	target
kaempferol	MET	target
kaempferol	ALK	target
kaempferol	AKT1	target
kaempferol	PARP1	target
kaempferol	TOP1	target
kaempferol	ESR1	target
kaempferol	PTGS2	target
kaempferol	TERT	target
kaempferol	CDK1	target
kaempferol	CCNB1	target
palmatine	RAC1	target
Niuxi	palmatinemol	
palmatine	AURKA	target
palmatine	MAP2K1	target
palmatine	CDK2	target
palmatine	PLK1	target
palmatine	MET	target
palmatine	MAPK8	target
palmatine	PIK3CB	target
palmatine	ALDH2	target
palmatine	ABCG2	target
palmatine	RAF1	target
palmatine	MST1R	target
palmatine	CYP1A1	target
palmatine	MDM2	target

palmatine	MTOR	target
palmatine	NTRK1	target
palmatine	CDK2	target
palmatine	CHEK2	target
palmatine	CDK4	target
palmatine	TGFBR2	target
palmatine	KIT	target
palmatine	FGFR3	target
palmatine	PDGFRB	target
palmatine	FLT4	target
palmatine	JAK1	target
palmatine	CCNA2	target
poriferasta-7,22E-die	AR	target
Niuxi	poriferas	mol
poriferasta-7,22E-die	CYP17A1	target
poriferasta-7,22E-die	ESR1	target
poriferasta-7,22E-die	ESR2	target
poriferasta-7,22E-die	VDR	target
poriferasta-7,22E-die	NOS2	target
poriferasta-7,22E-die	PPARG	target
poriferasta-7,22E-die	TERT	target
poriferasta-7,22E-die	PGR	target
poriferasta-7,22E-die	MAPK3	target
poriferasta-7,22E-die	PTPN11	target
poriferasta-7,22E-die	TOP2A	target
quercetin	IGF1R	target
Niuxi	quercetin	mol
quercetin	EGFR	target
quercetin	CA7	target
quercetin	PIK3R1	target
quercetin	DAPK1	target
quercetin	GSK3B	target
quercetin	SRC	target
quercetin	PTK2	target
quercetin	KDR	target
quercetin	MMP3	target
quercetin	ABCC1	target
quercetin	PLK1	target
quercetin	CDK1	target
quercetin	MMP9	target
quercetin	MMP2	target
quercetin	CA9	target
quercetin	MET	target
quercetin	ALK	target
quercetin	AKT1	target
quercetin	ABCB1	target
quercetin	ABCG2	target

quercetin	TOP2A	target
quercetin	ESR2	target
quercetin	CDK6	target
quercetin	CDK2	target
quercetin	PARP1	target
quercetin	TOP1	target
quercetin	TERT	target
quercetin	CDK1	target
quercetin	CCNB1	target
Spinasterol	AR	target
Niuxi	Spinastermol	
Spinasterol	CYP17A1	target
Spinasterol	ESR1	target
Spinasterol	ESR2	target
Spinasterol	VDR	target
Spinasterol	NOS2	target
Spinasterol	PPARG	target
Spinasterol	TERT	target
Spinasterol	MAPK3	target
Spinasterol	PTPN11	target
Spinasterol	TOP2A	target
Spinoside A	GLI1	target
Niuxi	Spinosidemol	
Spinoside A	JUN	target
Spinoside A	GRB2	target
Stigmasterol	AR	target
Niuxi	Stigmastemol	
Stigmasterol	CYP17A1	target
Stigmasterol	ESR1	target
Stigmasterol	ESR2	target
Stigmasterol	VDR	target
Stigmasterol	NOS2	target
Stigmasterol	PPARG	target
wogonin	PTGS2	target
Niuxi	wogonin	mol
wogonin	NOS2	target
wogonin	ABCB1	target
wogonin	KIT	target
wogonin	CDK1	target
wogonin	ESR2	target
wogonin	CYP1A1	target
wogonin	EGFR	target
wogonin	MCL1	target
wogonin	ABCG2	target
wogonin	ESR1	target
wogonin	TERT	target
wogonin	CA7	target

wogonin	CDK6	target
wogonin	CA9	target
wogonin	AR	target
wogonin	MET	target
wogonin	DAPK1	target
wogonin	HSP90AA1	target
wogonin	MMP9	target
wogonin	MMP2	target
wogonin	TOP1	target
wogonin	TOP2A	target
wogonin	PIK3R1	target
wogonin	MMP3	target
wogonin	ODC1	target
wogonin	SRC	target
wogonin	PLA2G2A	target
wogonin	GSK3B	target
wogonin	ABCC1	target
wogonin	CDK1	target
wogonin	CCNB1	target
beta-ecdysterone	TNF	target
Niuxi	beta-ecdymol	
beta-ecdysterone	NOS2	target
beta-ecdysterone	CYP17A1	target
beta-ecdysterone	AR	target
beta-ecdysterone	MAPK3	target
beta-ecdysterone	PGR	target
beta-ecdysterone	ESR1	target
beta-ecdysterone	ESR2	target
beta-ecdysterone	PTPN11	target
beta-ecdysterone	CDK2	target
beta-ecdysterone	MTOR	target
beta-ecdysterone	PIK3CA	target
beta-ecdysterone	NTRK1	target
beta-ecdysterone	CCNA2	target
beta-ecdysterone	MAPK1	target
beta-ecdysterone	MAPK8	target
beta-ecdysterone	MAP2K1	target
beta-ecdysterone	PPARG	target
beta-ecdysterone	KDR	target
beta-ecdysterone	FGFR1	target
beta-ecdysterone	MAPK14	target
beta-ecdysterone	RELA	target
beta-ecdysterone	FLT1	target
beta-ecdysterone	PDGFRB	target
beta-ecdysterone	KIT	target
beta-ecdysterone	FLT4	target
beta-ecdysterone	CDK2	target

beta-ecdysterone	CDK2	target
