# Supplementary material for: The Efficacy and Mechanism of Qinghua Jianpi Recipe in Inhibiting Canceration of Colorectal Adenoma Based on Inflammatory Cancer Transformation
Source: J Immunol Res. 2023 Feb 15;2023:4319551. doi: 10.1155/2023/4319551 (PMC9946765; doi:10.1155/2023/4319551)
Supplement: Supplementary Materials — The analysis data of the network pharmacology. Active ingredients in traditional Chinese medicine (1); 1011 targets in colorectal cancer (2); PPI topological analysis (3); topological analysis of 213 active components in the network diagram (4); MCODE analysis (5); biological processes (BP, GO enrichment analysis) (6); cell components (CC, GO enrichment analysis) (7); molecular function (MF, GO enrichment analysis) (8); KEGG analysis (9). [file 4319551.f1.zip › BP.pdf]

ID	Descriptic	GeneRatic	BgRatio	pvalue	p.adjust	qvalue	geneID	Count
GO:00181(	peptidyl-t	37/137	363/1867(	5.23E-32	9.72E-29	3.87E-29	MAP2K1/M/	37
GO:00182	peptidyl-t	37/137	366/1867(	7.09E-32	9.72E-29	3.87E-29	MAP2K1/M/	37
GO:00434(	regulatior	36/137	337/1867(	7.33E-32	9.72E-29	3.87E-29	MAPK14/T(	36
GO:00487	gland deve	39/137	434/1867(	1.16E-31	9.72E-29	3.87E-29	AR/ESR1/\	39
GO:00506	epithelial	39/137	434/1867(	1.16E-31	9.72E-29	3.87E-29	AR/ESR1/\	39
GO:00182(	peptidyl-s	35/137	322/1867(	3.16E-31	2.20E-28	8.75E-29	MAPK14/T(	35
GO:00181(	peptidyl-s	34/137	299/1867(	5.38E-31	3.21E-28	1.28E-28	MAPK14/T(	34
GO:00719(	positive r	35/137	334/1867(	1.14E-30	5.97E-28	2.38E-28	MAPK14/T(	35
GO:00434(	positive r	32/137	258/1867(	2.19E-30	1.02E-27	4.05E-28	MAPK14/T(	32
GO:00096	response t	29/137	210/1867(	6.29E-29	2.63E-26	1.05E-26	PPARG/MAI	29
GO:00434	protein ki	31/137	269/1867(	1.99E-28	7.55E-26	3.01E-26	ESR1/TGFI	31
GO:00518	positive r	27/137	176/1867(	3.12E-28	1.09E-25	4.33E-26	ESR1/TGFI	27
GO:00486(	reproducti	36/137	431/1867(	4.80E-28	1.54E-25	6.14E-26	AR/ESR1/\	36
GO:00614	reproducti	36/137	434/1867(	6.13E-28	1.83E-25	7.28E-26	AR/ESR1/\	36
GO:00518	regulatior	29/137	244/1867(	5.40E-27	1.50E-24	5.99E-25	ESR1/TGFI	29
GO:00971	extrinsic	28/137	224/1867(	1.07E-26	2.81E-24	1.12E-24	AR/TGFBR	28
GO:00330(	muscle ce	28/137	239/1867(	6.80E-26	1.67E-23	6.65E-24	PPARG/MAI	28
GO:00704	response t	33/137	394/1867(	1.02E-25	2.36E-23	9.39E-24	NOS2/PPAI	33
GO:00069	response t	34/137	451/1867(	5.39E-25	1.18E-22	4.72E-23	MAPK3/SR(	34
GO:00467	protein au	27/137	235/1867(	9.46E-25	1.98E-22	7.87E-23	MAPK3/SR(	27
GO:00712	cellular r	30/137	331/1867(	2.17E-24	4.12E-22	1.64E-22	MAPK14/M/	30
GO:01040(	cellular r	30/137	331/1867(	2.17E-24	4.12E-22	1.64E-22	MAPK14/M/	30
GO:00011(	response t	30/137	343/1867(	6.20E-24	1.13E-21	4.48E-22	PPARG/SR(	30
GO:00506	regulatior	31/137	378/1867(	7.00E-24	1.22E-21	4.85E-22	AR/VDR/PI	31
GO:00703	ERK1 and I	29/137	317/1867(	1.07E-23	1.78E-21	7.09E-22	MAP2K1/M/	29
GO:19038	positive r	29/137	324/1867(	1.99E-23	3.19E-21	1.27E-21	MAPK14/TI	29
GO:00016(	response t	30/137	359/1867(	2.36E-23	3.65E-21	1.45E-21	NOS2/CA9/	30
GO:00362	response t	30/137	370/1867(	5.70E-23	8.50E-21	3.39E-21	NOS2/CA9/	30
GO:00075(	aging	28/137	321/1867(	2.53E-22	3.65E-20	1.45E-20	MAPK14/M/	28
GO:00003(	response t	25/137	232/1867(	2.98E-22	4.15E-20	1.65E-20	MAPK3/SR(	25
GO:00466	response t	28/137	327/1867(	4.20E-22	5.66E-20	2.26E-20	SRC/MET/S	28
GO:00703	regulatior	27/137	300/1867(	6.72E-22	8.74E-20	3.48E-20	MAP2K1/M/	27
GO:00321	activatior	28/137	333/1867(	6.91E-22	8.74E-20	3.48E-20	MAPK14/T(	28
GO:00345	cellular r	27/137	302/1867(	8.01E-22	9.84E-20	3.92E-20	MAPK3/SR(	27
GO:00093	response t	31/137	448/1867(	1.12E-21	1.34E-19	5.32E-20	MAPK14/K	31
GO:00140(	phosphatic	21/137	148/1867(	2.35E-21	2.73E-19	1.09E-19	MAPK3/SR(	21
GO:00434	response t	30/137	436/1867(	6.50E-21	7.34E-19	2.92E-19	PPARG/PTI	30
GO:00480	phosphatic	22/137	181/1867(	8.03E-21	8.83E-19	3.52E-19	MAPK3/SR(	22
GO:00480	inositol l	22/137	184/1867(	1.16E-20	1.24E-18	4.94E-19	MAPK3/SR(	22
GO:00316(	response t	31/137	499/1867(	2.65E-20	2.77E-18	1.10E-18	VDR/PPAR(	31
GO:00485	response t	28/137	385/1867(	3.50E-20	3.57E-18	1.42E-18	AR/ESR1/I	28
GO:00140(	regulatior	19/137	124/1867(	4.62E-20	4.60E-18	1.83E-18	MAPK3/SR(	19
GO:00713	cellular r	26/137	321/1867(	6.05E-20	5.88E-18	2.34E-18	PPARG/PTI	26
GO:00611	mammary gl	16/137	72/18670	8.35E-20	7.93E-18	3.16E-18	AR/ESR1/\	16
GO:00973(	response t	23/137	233/1867(	1.19E-19	1.10E-17	4.39E-18	PPARG/TGI	23
GO:00506	positive r	22/137	206/1867(	1.41E-19	1.28E-17	5.10E-18	AR/TGFBR	22

GO:20012:	regulation	28/137	406/1867(	1.45E-19	1.29E-17	5.12E-18	AR/TGFBR1	28
GO:00356(	cellular r	27/137	369/1867(	1.52E-19	1.32E-17	5.27E-18	NOS2/MAPI	27
GO:00714(	cellular r	26/137	339/1867(	2.38E-19	2.03E-17	8.09E-18	VDR/PPAR(	26
GO:00703'	positive r	22/137	215/1867(	3.60E-19	3.01E-17	1.20E-17	MAP2K1/M/	22
GO:00712(	cellular r	16/137	79/18670	4.19E-19	3.43E-17	1.37E-17	MAPK3/PTI	16
GO:19016!	cellular r	27/137	385/1867(	4.54E-19	3.65E-17	1.45E-17	PPARG/PTI	27
GO:00510!	regulation	28/137	429/1867(	6.25E-19	4.93E-17	1.96E-17	PPARG/MAI	28
GO:00486(	positive r	17/137	101/1867(	8.89E-19	6.87E-17	2.74E-17	TERT/MMP2	17
GO:00486(	regulation	20/137	169/1867(	9.07E-19	6.88E-17	2.74E-17	PPARG/TEI	20
GO:00420(	gliogenesi	24/137	290/1867(	1.13E-18	8.42E-17	3.35E-17	PPARG/MAI	24
GO:00486!	smooth mus	20/137	171/1867(	1.15E-18	8.42E-17	3.35E-17	PPARG/TEI	20
GO:00510!	positive r	22/137	228/1867(	1.29E-18	9.32E-17	3.71E-17	MAPK3/SR(	22
GO:00352(	organ grov	21/137	204/1867(	2.17E-18	1.53E-16	6.08E-17	AR/ESR1/M	21
GO:00140(	positive r	16/137	87/18670	2.19E-18	1.53E-16	6.08E-17	SRC/KDR/1	16
GO:20012:	regulation	19/137	155/1867(	3.60E-18	2.47E-16	9.82E-17	AR/TGFBR1	19
GO:00486:	regulation	25/137	347/1867(	5.63E-18	3.79E-16	1.51E-16	AR/MAPK14	25
GO:19054'	regulation	20/137	187/1867(	6.92E-18	4.59E-16	1.83E-16	AR/MMP14/	20
GO:00457(	positive r	26/137	389/1867(	7.25E-18	4.73E-16	1.88E-16	TERT/PTPN	26
GO:00100(	glial cell	21/137	218/1867(	8.63E-18	5.55E-16	2.21E-16	PPARG/MAI	21
GO:00725(	reactive c	23/137	284/1867(	1.03E-17	6.49E-16	2.58E-16	NOS2/MAPI	23
GO:20003'	regulation	20/137	195/1867(	1.59E-17	9.94E-16	3.96E-16	MAPK14/PI	20
GO:00346:	cellular r	19/137	168/1867(	1.69E-17	1.04E-15	4.13E-16	MAPK3/SR(	19
GO:00100:	response t	25/137	364/1867(	1.76E-17	1.06E-15	4.23E-16	TERT/MAPI	25
GO:00308'	mammary gl	18/137	143/1867(	1.79E-17	1.07E-15	4.24E-16	AR/ESR1/V	18
GO:00310(	regenerati	20/137	198/1867(	2.16E-17	1.27E-15	5.06E-16	PPARG/MAI	20
GO:19054'	positive r	17/137	122/1867(	2.51E-17	1.46E-15	5.80E-16	BCL2/LGAI	17
GO:00448	cell cycle	23/137	298/1867(	2.98E-17	1.71E-15	6.80E-16	TERT/BCL2	23
GO:00300(	myeloid ce	26/137	416/1867(	3.75E-17	2.12E-15	8.42E-16	PPARG/MAI	26
GO:20012:	negative r	16/137	104/1867(	4.43E-17	2.46E-15	9.81E-16	AR/TGFBR1	16
GO:00459:	positive r	22/137	270/1867(	4.91E-17	2.70E-15	1.07E-15	MAPK14/T(	22
GO:00074(	axonogenes	27/137	468/1867(	6.46E-17	3.51E-15	1.40E-15	MAP2K1/M/	27
GO:00709(	neuron dea	24/137	348/1867(	7.53E-17	4.03E-15	1.60E-15	TERT/GSK3	24
GO:00611:	morphogene	19/137	182/1867(	7.71E-17	4.08E-15	1.62E-15	AR/ESR1/V	19
GO:00106:	epithelial	24/137	351/1867(	9.15E-17	4.78E-15	1.90E-15	PPARG/TGI	24
GO:00510(	regulation	26/137	432/1867(	9.39E-17	4.84E-15	1.93E-15	AR/ESR1/I	26
GO:00901:	epithelium	24/137	354/1867(	1.11E-16	5.66E-15	2.25E-15	PPARG/TGI	24
GO:00901:	tissue mig	24/137	360/1867(	1.63E-16	8.18E-15	3.26E-15	PPARG/TGI	24
GO:00311(	animal org	14/137	73/18670	1.70E-16	8.47E-15	3.37E-15	PPARG/CDI	14
GO:00019:	endothelia	19/137	191/1867(	1.92E-16	9.39E-15	3.74E-15	PPARG/TGI	19
GO:00457(	positive r	25/137	403/1867(	1.93E-16	9.39E-15	3.74E-15	PTPN11/SI	25
GO:00447(	multi-mult	20/137	222/1867(	2.06E-16	9.87E-15	3.93E-15	AR/ESR1/V	20
GO:00075(	female pre	19/137	192/1867(	2.11E-16	1.00E-14	4.00E-15	AR/ESR1/V	19
GO:00324(	response t	23/137	330/1867(	2.81E-16	1.32E-14	5.25E-15	NOS2/MAPI	23
GO:00017(	morphogene	19/137	196/1867(	3.11E-16	1.44E-14	5.75E-15	AR/ESR1/V	19
GO:00485:	rhythmic p	22/137	295/1867(	3.20E-16	1.47E-14	5.85E-15	ESR1/NOS2	22
GO:20012:	negative r	20/137	230/1867(	4.10E-16	1.86E-14	7.42E-15	AR/TGFBR1	20
GO:00016(	ameboidal-	26/137	461/1867(	4.51E-16	2.02E-14	8.06E-15	PPARG/TGI	26

GO:19028(	regulatior	19/137	202/1867(	5.47E-16	2.43E-14	9.68E-15	TERT/BCL2	19
GO:00022:	response t	23/137	343/1867(	6.52E-16	2.86E-14	1.14E-14	NOS2/MAPI	23
GO:00075	sex differ	21/137	270/1867(	6.87E-16	2.99E-14	1.19E-14	AR/CYP17/	21
GO:00328(	response t	21/137	272/1867(	7.97E-16	3.43E-14	1.37E-14	PPARG/PTI	21
GO:00020(	epithelial	19/137	207/1867(	8.63E-16	3.68E-14	1.47E-14	AR/ESR1/I	19
GO:20003	positive r	15/137	102/1867(	8.93E-16	3.77E-14	1.50E-14	MAPK14/PI	15
GO:00712	cellular r	19/137	209/1867(	1.03E-15	4.31E-14	1.72E-14	PPARG/SR(	19
GO:19012	regulatior	22/137	313/1867(	1.11E-15	4.56E-14	1.82E-14	TERT/GSK:	22
GO:00001	activatior	17/137	152/1867(	1.11E-15	4.56E-14	1.82E-14	MAPK14/M/	17
GO:00165	histone pl	11/137	38/18670	2.24E-15	9.10E-14	3.62E-14	MAPK3/CHI	11
GO:00075	response t	19/137	219/1867(	2.46E-15	9.87E-14	3.93E-14	VDR/PPAR(	19
GO:19049	positive r	25/137	456/1867(	3.40E-15	1.35E-13	5.38E-14	MAPK14/M/	25
GO:00459	positive r	17/137	163/1867(	3.65E-15	1.44E-13	5.72E-14	TERT/PTPI	17
GO:00380	signal tra	13/137	72/18670	4.91E-15	1.90E-13	7.56E-14	TERT/GSK:	13
GO:00971	extrinsic	13/137	72/18670	4.91E-15	1.90E-13	7.56E-14	TERT/GSK:	13
GO:00459	negative r	22/137	338/1867(	5.49E-15	2.10E-13	8.37E-14	BCL2/CHEI	22
GO:19043	regulatior	15/137	115/1867(	5.66E-15	2.15E-13	8.56E-14	AR/MMP14/	15
GO:00480	vascular (	14/137	96/18670	9.56E-15	3.60E-13	1.43E-13	MAPK14/SI	14
GO:00025	myeloid le	18/137	204/1867(	1.04E-14	3.88E-13	1.54E-13	PPARG/MAI	18
GO:00226	gland morp	15/137	120/1867(	1.08E-14	4.01E-13	1.60E-13	AR/ESR1/V	15
GO:00102	response t	16/137	147/1867(	1.25E-14	4.57E-13	1.82E-13	MAPK14/B(	16
GO:00074	axon guida	20/137	276/1867(	1.38E-14	5.00E-13	1.99E-13	MAPK3/PTI	20
GO:00974	neuron pro	20/137	277/1867(	1.48E-14	5.32E-13	2.12E-13	MAPK3/PTI	20
GO:00604	mesenchyme	20/137	278/1867(	1.58E-14	5.64E-13	2.25E-13	TGFBR1/M/	20
GO:00000	G1/S trans	20/137	279/1867(	1.69E-14	5.99E-13	2.39E-13	TERT/BCL2	20
GO:00487	branching	16/137	150/1867(	1.72E-14	6.05E-13	2.41E-13	AR/ESR1/V	16
GO:00224(	regulatior	23/137	403/1867(	2.08E-14	7.25E-13	2.89E-13	MAPK14/P1	23
GO:00018	placenta (	16/137	152/1867(	2.13E-14	7.34E-13	2.92E-13	VDR/PPAR(	16
GO:00605(	epithelial	21/137	322/1867(	2.32E-14	7.93E-13	3.16E-13	AR/ESR1/V	21
GO:00321(	positive r	21/137	323/1867(	2.46E-14	8.36E-13	3.33E-13	MAPK14/M/	21
GO:20000	regulatior	17/137	184/1867(	2.79E-14	9.37E-13	3.73E-13	TERT/BCL2	17
GO:00000	cell cycle	18/137	216/1867(	2.83E-14	9.37E-13	3.73E-13	MAPK14/P1	18
GO:00328(	cellular r	18/137	216/1867(	2.83E-14	9.37E-13	3.73E-13	PPARG/PTI	18
GO:00084(	gonad deve	18/137	217/1867(	3.06E-14	1.01E-12	4.01E-13	AR/ESR1/1	18
GO:00971	intrinsic	20/137	289/1867(	3.30E-14	1.08E-12	4.29E-13	SRC/MMP9/	20
GO:00601	maternal p	12/137	64/18670	3.54E-14	1.15E-12	4.57E-13	AR/ESR1/V	12
GO:00487(	mesenchyma	18/137	219/1867(	3.59E-14	1.16E-12	4.60E-13	TGFBR1/M/	18
GO:00620	regulatior	24/137	459/1867(	3.66E-14	1.17E-12	4.64E-13	VDR/NOS2/	24
GO:00451	developmer	18/137	223/1867(	4.92E-14	1.56E-12	6.20E-13	AR/ESR1/1	18
GO:00323	response t	15/137	134/1867(	5.75E-14	1.79E-12	7.13E-13	ESR1/PDGI	15
GO:00485(	digestive	15/137	134/1867(	5.75E-14	1.79E-12	7.13E-13	RET/KIT/I	15
GO:00900(	positive r	20/137	298/1867(	5.90E-14	1.82E-12	7.27E-13	TERT/FGFI	20
GO:00510	positive r	19/137	261/1867(	6.09E-14	1.87E-12	7.45E-13	AR/ESR1/I	19
GO:19016	response t	17/137	193/1867(	6.16E-14	1.88E-12	7.48E-13	AR/PPARG/	17
GO:19019	regulatior	24/137	480/1867(	9.71E-14	2.94E-12	1.17E-12	TERT/BCL2	24
GO:00466	regulatior	14/137	113/1867(	9.87E-14	2.97E-12	1.18E-12	MAPK14/T(	14
GO:00714	cellular r	18/137	234/1867(	1.13E-13	3.38E-12	1.35E-12	PPARG/CA	18

GO:00106:	positive r	16/137	171/1867(	1.37E-13	4.05E-12	1.61E-12	SRC/KDR/M	16
GO:00015(	ossificati	22/137	398/1867(	1.55E-13	4.56E-12	1.82E-12	MAPK14/MA	22
GO:00332:	response t	13/137	93/18670	1.58E-13	4.61E-12	1.84E-12	VDR/PPARG	13
GO:00514(	neuron apo	18/137	239/1867(	1.63E-13	4.73E-12	1.88E-12	TERT/BCL2	18
GO:00714	cellular r	17/137	207/1867(	1.96E-13	5.63E-12	2.24E-12	CA9/TERT/	17
GO:00019	regulation	14/137	119/1867(	2.05E-13	5.84E-12	2.33E-12	SRC/KDR/(	14
GO:00458:	positive r	15/137	146/1867(	2.07E-13	5.84E-12	2.33E-12	PPARG/SRC	15
GO:00551	digestive	15/137	146/1867(	2.07E-13	5.84E-12	2.33E-12	RET/KIT/I	15
GO:19030	regulation	13/137	95/18670	2.10E-13	5.88E-12	2.34E-12	AR/MMP14/	13
GO:00076	circadian	17/137	208/1867(	2.12E-13	5.89E-12	2.35E-12	NOS2/PPAI	17
GO:00019	regulation	16/137	176/1867(	2.15E-13	5.95E-12	2.37E-12	PPARG/TGI	16
GO:19001	positive r	12/137	74/18670	2.23E-13	6.11E-12	2.43E-12	MAPK14/TI	12
GO:00517(	interactio	17/137	209/1867(	2.29E-13	6.25E-12	2.49E-12	CTSB/SRC/	17
GO:00085	female gor	13/137	96/18670	2.41E-13	6.53E-12	2.60E-12	ESR1/PGR/	13
GO:00192	regulation	22/137	410/1867(	2.82E-13	7.61E-12	3.03E-12	VDR/PPARG	22
GO:00514(	stress-act	19/137	286/1867(	3.16E-13	8.46E-12	3.37E-12	MAPK14/MA	19
GO:00105(	regulation	20/137	327/1867(	3.36E-13	8.95E-12	3.56E-12	MAPK3/KDI	20
GO:00510	regulation	21/137	373/1867(	4.10E-13	1.08E-11	4.31E-12	PPARG/TGI	21
GO:00512	positive r	22/137	418/1867(	4.16E-13	1.09E-11	4.36E-12	MAPK14/MA	22
GO:00362	cellular r	17/137	217/1867(	4.23E-13	1.10E-11	4.37E-12	CA9/TERT/	17
GO:00712	cellular r	17/137	217/1867(	4.23E-13	1.10E-11	4.37E-12	MAPK3/MMI	17
GO:00486:	positive r	16/137	184/1867(	4.30E-13	1.10E-11	4.39E-12	MAPK14/MA	16
GO:00106:	regulation	19/137	291/1867(	4.31E-13	1.10E-11	4.39E-12	PPARG/SRC	19
GO:00181(	peptidyl-t	14/137	126/1867(	4.58E-13	1.16E-11	4.62E-12	TGFBR1/MA	14
GO:00105	positive r	11/137	59/18670	4.58E-13	1.16E-11	4.62E-12	ESR1/FGFI	11
GO:00465	developmer	13/137	101/1867(	4.72E-13	1.19E-11	4.73E-12	ESR1/PGR/	13
GO:19013	regulation	22/137	422/1867(	5.04E-13	1.26E-11	5.02E-12	PPARG/TEI	22
GO:00507(	positive r	23/137	474/1867(	6.27E-13	1.56E-11	6.21E-12	PPARG/MAI	23
GO:00485(	embryonic	22/137	428/1867(	6.69E-13	1.65E-11	6.58E-12	TGFBR1/MA	22
GO:00148	striated m	12/137	81/18670	6.87E-13	1.69E-11	6.72E-12	MAPK14/TG	12
GO:00714	cellular r	16/137	191/1867(	7.68E-13	1.88E-11	7.47E-12	MAPK14/CI	16
GO:19028(	negative r	14/137	131/1867(	7.88E-13	1.90E-11	7.58E-12	BCL2/CDK	14
GO:00331	positive r	13/137	105/1867(	7.88E-13	1.90E-11	7.58E-12	RET/BCL2/	13
GO:00481	regulation	12/137	83/18670	9.29E-13	2.23E-11	8.88E-12	ESR1/PPAI	12
GO:00160	cell growt	23/137	484/1867(	9.66E-13	2.31E-11	9.18E-12	ESR2/PPAI	23
GO:00466	positive r	11/137	63/18670	9.84E-13	2.33E-11	9.29E-12	MAPK14/MA	11
GO:00481	fibroblast	12/137	84/18670	1.08E-12	2.53E-11	1.01E-11	ESR1/PPAI	12
GO:00182	peptidyl-t	14/137	134/1867(	1.08E-12	2.53E-11	1.01E-11	TGFBR1/MA	14
GO:00018	liver deve	14/137	135/1867(	1.20E-12	2.79E-11	1.11E-11	MET/PIK3(	14
GO:00070	mitotic ce	15/137	165/1867(	1.26E-12	2.92E-11	1.16E-11	BCL2L1/CI	15
GO:00480	ephrin rec	12/137	86/18670	1.44E-12	3.32E-11	1.32E-11	PTPN11/SI	12
GO:00610(	hepaticobi	14/137	138/1867(	1.62E-12	3.73E-11	1.48E-11	MET/PIK3(	14
GO:00712	cellular r	17/137	236/1867(	1.66E-12	3.77E-11	1.50E-11	NOS2/MAPI	17
GO:00094	response t	19/137	314/1867(	1.66E-12	3.77E-11	1.50E-11	KIT/BCL2/	19
GO:00310	stress-act	19/137	315/1867(	1.76E-12	3.97E-11	1.58E-11	MAPK14/MA	19
GO:00070	cell cycle	17/137	237/1867(	1.77E-12	3.98E-11	1.58E-11	TGFBR1/MA	17
GO:00509(	leukocyte	23/137	499/1867(	1.81E-12	4.05E-11	1.61E-11	MAPK14/MA	23

GO:00432	anoikis	9/137	34/18670	2.12E-12	4.72E-11	1.88E-11	SRC/BCL2/	9
GO:00094	response t	14/137	141/1867(	2.19E-12	4.83E-11	1.92E-11	BCL2/CHEI	14
GO:00712	cellular r	16/137	205/1867(	2.29E-12	5.02E-11	2.00E-11	NOS2/MAPI	16
GO:00303	lung devel	15/137	172/1867(	2.31E-12	5.06E-11	2.01E-11	MAP2K1/P(	15
GO:00381	ERBB signa	14/137	142/1867(	2.41E-12	5.25E-11	2.09E-11	PTPN11/SI	14
GO:00329	collagen m	13/137	115/1867(	2.59E-12	5.58E-11	2.22E-11	PPARG/CTS	13
GO:00466	female se	13/137	115/1867(	2.59E-12	5.58E-11	2.22E-11	ESR1/PGR/	13
GO:19001	regulatior	13/137	116/1867(	2.90E-12	6.21E-11	2.47E-11	MAPK14/TI	13
GO:00488	homeostasi	17/137	246/1867(	3.23E-12	6.86E-11	2.73E-11	MAPK14/P7	17
GO:00303	respirator	15/137	176/1867(	3.23E-12	6.86E-11	2.73E-11	MAP2K1/P(	15
GO:00435	regulatior	16/137	210/1867(	3.31E-12	6.94E-11	2.76E-11	TERT/BCL2	16
GO:00105	regulatior	11/137	70/18670	3.31E-12	6.94E-11	2.76E-11	ESR1/FGFI	11
GO:00421	T cell act	22/137	464/1867(	3.32E-12	6.94E-11	2.76E-11	PTPN11/SI	22
GO:00457	positive r	9/137	36/18670	3.76E-12	7.82E-11	3.11E-11	FGFR1/CDI	9
GO:00712	cellular r	16/137	212/1867(	3.82E-12	7.91E-11	3.15E-11	NOS2/MAPI	16
GO:19012	positive r	12/137	94/18670	4.28E-12	8.80E-11	3.50E-11	GSK3B/MT(	12
GO:00601	positive r	11/137	72/18670	4.57E-12	9.35E-11	3.72E-11	ESR1/FGFI	11
GO:00108	regulatior	16/137	215/1867(	4.74E-12	9.61E-11	3.83E-11	SRC/KDR/(	16
GO:00432	regulatior	16/137	215/1867(	4.74E-12	9.61E-11	3.83E-11	PPARG/SR(	16
GO:19034	reactive (	13/137	122/1867(	5.58E-12	1.13E-10	4.48E-11	NOS2/MTOI	13
GO:00071	leukocyte	19/137	337/1867(	5.75E-12	1.16E-10	4.60E-11	PTPN11/SI	19
GO:00457	regulatior	20/137	383/1867(	6.17E-12	1.23E-10	4.91E-11	PPARG/TEI	20
GO:00454	response t	13/137	125/1867(	7.63E-12	1.51E-10	6.02E-11	STAT3/CDI	13
GO:20001	negative r	13/137	125/1867(	7.63E-12	1.51E-10	6.02E-11	BCL2/CDK1	13
GO:00550	regulatior	12/137	100/1867(	9.06E-12	1.79E-10	7.11E-11	MAPK14/T(	12
GO:00352	endocrine	13/137	127/1867(	9.37E-12	1.84E-10	7.31E-11	TGFBR1/M/	13
GO:00315	DNA integr	14/137	157/1867(	9.57E-12	1.87E-10	7.43E-11	MAPK14/P7	14
GO:19030	regulatior	18/137	304/1867(	9.61E-12	1.87E-10	7.43E-11	PTPN11/SI	18
GO:00068	nitric oxi	11/137	77/18670	9.78E-12	1.89E-10	7.53E-11	NOS2/MTOI	11
GO:00600	regulatior	10/137	57/18670	1.05E-11	2.01E-10	8.01E-11	MAPK14/T(	10
GO:00550	regulatior	11/137	78/18670	1.13E-11	2.17E-10	8.63E-11	MAPK14/T(	11
GO:19019	regulatior	21/137	444/1867(	1.14E-11	2.17E-10	8.63E-11	TERT/BCL2	21
GO:19019	negative r	17/137	267/1867(	1.20E-11	2.27E-10	9.04E-11	BCL2/CHEI	17
GO:00069	autophagy	22/137	496/1867(	1.23E-11	2.31E-10	9.20E-11	MAPK3/SR(	22
GO:00619	process ut	22/137	496/1867(	1.23E-11	2.31E-10	9.20E-11	MAPK3/SR(	22
GO:00313	positive r	17/137	268/1867(	1.27E-11	2.38E-10	9.47E-11	ESR1/SRC/	17
GO:00097	response t	16/137	230/1867(	1.32E-11	2.45E-10	9.76E-11	IGF1R/PII	16
GO:19040	positive r	16/137	230/1867(	1.32E-11	2.45E-10	9.76E-11	TERT/KDR/	16
GO:00319	response t	14/137	162/1867(	1.47E-11	2.71E-10	1.08E-10	SRC/BCL2/	14
GO:00487	cardiac mu	16/137	233/1867(	1.61E-11	2.96E-10	1.18E-10	MAPK14/T(	16
GO:00550	cardiac mu	12/137	105/1867(	1.63E-11	2.99E-10	1.19E-10	MAPK14/T(	12
GO:00435	endothelia	17/137	273/1867(	1.70E-11	3.10E-10	1.24E-10	PPARG/TGI	17
GO:00487	astrocyte	11/137	81/18670	1.73E-11	3.15E-10	1.25E-10	MAP2K1/M/	11
GO:00605	respirator	15/137	198/1867(	1.77E-11	3.20E-10	1.28E-10	MAP2K1/P(	15
GO:19019	positive r	12/137	106/1867(	1.83E-11	3.29E-10	1.31E-10	TERT/CDK1	12
GO:00462	nitric oxi	11/137	82/18670	1.99E-11	3.56E-10	1.42E-10	NOS2/MTOI	11
GO:00328	regulatior	16/137	237/1867(	2.08E-11	3.71E-10	1.48E-10	MAP2K1/M/	16

GO:00458(	positive r	19/137	363/1867(	2.09E-11	3.72E-10	1.48E-10	PPARG/MAI	19
GO:00108(	positive r	9/137	43/18670	2.16E-11	3.82E-10	1.52E-10	ESR1/FGFI	9
GO:00711!	regulation	12/137	108/1867(	2.29E-11	4.04E-10	1.61E-10	CDK1/AURI	12
GO:00703(	regulation	16/137	239/1867(	2.36E-11	4.12E-10	1.64E-10	MAP2K1/MA	16
GO:20001:	regulation	16/137	239/1867(	2.36E-11	4.12E-10	1.64E-10	PPARG/SR(	16
GO:00550:	positive r	10/137	62/18670	2.52E-11	4.39E-10	1.75E-10	MAPK14/MA	10
GO:00018:	positive r	21/137	464/1867(	2.59E-11	4.49E-10	1.79E-10	MAPK14/MA	21
GO:00604:	regulation	11/137	84/18670	2.60E-11	4.49E-10	1.79E-10	MAPK14/T(	11
GO:00380!	Fc recepto	16/137	241/1867(	2.67E-11	4.59E-10	1.83E-10	MAPK3/SR(	16
GO:00457(	positive r	15/137	204/1867(	2.71E-11	4.65E-10	1.85E-10	TERT/KDR/	15
GO:19047(	regulation	11/137	85/18670	2.97E-11	5.00E-10	1.99E-10	PPARG/TEI	11
GO:19908:	vascular s	11/137	85/18670	2.97E-11	5.00E-10	1.99E-10	PPARG/TEI	11
GO:20010!	reactive r	11/137	85/18670	2.97E-11	5.00E-10	1.99E-10	NOS2/MTOI	11
GO:00331:	regulation	13/137	139/1867(	2.98E-11	5.00E-10	1.99E-10	RET/BCL2/	13
GO:00466!	response t	10/137	63/18670	2.98E-11	5.00E-10	1.99E-10	TERT/MAPI	10
GO:19002:	regulation	9/137	45/18670	3.35E-11	5.60E-10	2.23E-10	ESR1/FGFI	9
GO:00086:	extrinsic	11/137	86/18670	3.39E-11	5.64E-10	2.24E-10	BCL2/BCL:	11
GO:00019:	positive r	12/137	112/1867(	3.54E-11	5.84E-10	2.33E-10	TGFBR1/KI	12
GO:00604:	heart grow	12/137	112/1867(	3.54E-11	5.84E-10	2.33E-10	MAPK14/T(	12
GO:00082!	insulin re	13/137	141/1867(	3.57E-11	5.87E-10	2.34E-10	PTPN11/SI	13
GO:00016!	urogenital	18/137	330/1867(	3.74E-11	6.13E-10	2.44E-10	AR/ESR1/I	18
GO:00458:	positive r	11/137	87/18670	3.85E-11	6.26E-10	2.49E-10	MAPK14/MA	11
GO:00486:	positive r	11/137	87/18670	3.85E-11	6.26E-10	2.49E-10	MAPK14/MA	11
GO:00604:	mammary gl	9/137	46/18670	4.14E-11	6.70E-10	2.67E-10	AR/ESR1/V	9
GO:19018(	positive r	11/137	88/18670	4.38E-11	7.06E-10	2.81E-10	MAPK14/MA	11
GO:00713!	cellular r	16/137	250/1867(	4.63E-11	7.44E-10	2.96E-10	AR/ESR1/I	16
GO:00305:	collagen c	9/137	47/18670	5.09E-11	8.08E-10	3.22E-10	CTSB/MMP:	9
GO:20012:	regulation	9/137	47/18670	5.09E-11	8.08E-10	3.22E-10	TERT/RET/	9
GO:00000:	DNA damage	13/137	145/1867(	5.09E-11	8.08E-10	3.22E-10	MAPK14/P:	13
GO:00075(	cell aging	12/137	116/1867(	5.37E-11	8.50E-10	3.38E-10	MAPK14/MA	12
GO:20000:	regulation	16/137	253/1867(	5.53E-11	8.72E-10	3.47E-10	AR/ESR1/V	16
GO:00425:	response t	13/137	146/1867(	5.55E-11	8.72E-10	3.47E-10	SRC/MET/S	13
GO:00600:	cardiac mu	10/137	67/18670	5.64E-11	8.80E-10	3.50E-10	MAPK14/T(	10
GO:00487:	tissue rem	14/137	179/1867(	5.64E-11	8.80E-10	3.50E-10	VDR/SRC/I	14
GO:00381:	ERBB2 sign	8/137	32/18670	6.20E-11	9.60E-10	3.82E-10	SRC/PIK3(	8
GO:00435!	positive r	8/137	32/18670	6.20E-11	9.60E-10	3.82E-10	SRC/PDGFI	8
GO:00550:	positive r	9/137	48/18670	6.23E-11	9.60E-10	3.82E-10	MAPK14/MA	9
GO:00433!	regulation	15/137	217/1867(	6.54E-11	1.00E-09	4.00E-10	TGFBR1/TI	15
GO:19030:	positive r	15/137	218/1867(	6.98E-11	1.07E-09	4.25E-10	PTPN11/SI	15
GO:00147(	striated m	19/137	390/1867(	7.17E-11	1.09E-09	4.35E-10	MAPK14/T(	19
GO:19047(	positive r	9/137	49/18670	7.58E-11	1.15E-09	4.58E-10	TERT/MMP:	9
GO:00606(	mammary gl	8/137	33/18670	8.14E-11	1.23E-09	4.90E-10	AR/ESR1/V	8
GO:00603:	cell chemo	17/137	304/1867(	9.23E-11	1.39E-09	5.53E-10	MAPK14/MA	17
GO:00162(	regulation	13/137	152/1867(	9.24E-11	1.39E-09	5.53E-10	MAPK14/T(	13
GO:00620:	positive r	13/137	153/1867(	1.00E-10	1.50E-09	5.98E-10	NOS2/PPAI	13
GO:00714:	cellular r	10/137	71/18670	1.03E-10	1.53E-09	6.09E-10	MAPK14/B(	10
GO:00071(	cell-matri	15/137	225/1867(	1.09E-10	1.62E-09	6.45E-10	SRC/KDR/(	15

GO:00481⁴	positive r	9/137	51/18670	1.11E-10	1.64E-09	6.54E-10	ESR1/PDGI	9
GO:19018(	regulatior	13/137	155/1867(	1.18E-10	1.74E-09	6.94E-10	MAPK14/T(	13
GO:00525⁴	regulatior	20/137	452/1867(	1.21E-10	1.77E-09	7.06E-10	PPARG/MAI	20
GO:00486:	regulatior	13/137	156/1867(	1.28E-10	1.88E-09	7.47E-10	MAPK14/T(	13
GO:00604:	positive r	9/137	52/18670	1.33E-10	1.95E-09	7.75E-10	MAPK14/M/	9
GO:00436:	response t	10/137	73/18670	1.36E-10	1.97E-09	7.84E-10	ESR1/PPAI	10
GO:00724(	signal tra	10/137	73/18670	1.36E-10	1.97E-09	7.84E-10	CHEK1/CDI	10
GO:00724:	signal tra	10/137	73/18670	1.36E-10	1.97E-09	7.84E-10	CHEK1/CDI	10
GO:00601!	regulatior	11/137	98/18670	1.44E-10	2.07E-09	8.26E-10	ESR1/FGFI	11
GO:00605:	muscle tis	19/137	408/1867(	1.54E-10	2.22E-09	8.83E-10	MAPK14/T(	19
GO:00723!	signal tra	10/137	74/18670	1.57E-10	2.24E-09	8.92E-10	CHEK1/CDI	10
GO:00902:	positive r	8/137	36/18670	1.74E-10	2.48E-09	9.88E-10	SRC/PDGFI	8
GO:01500	neuroinfla	10/137	75/18670	1.80E-10	2.55E-09	1.02E-09	MMP9/MMP:	10
GO:00432!	regulatior	20/137	467/1867(	2.14E-10	3.03E-09	1.21E-09	ESR1/PTPI	20
GO:00380{	peptidyl-t	8/137	37/18670	2.21E-10	3.12E-09	1.24E-09	MAPK3/SR(	8
GO:00015⁴	ovarian fc	9/137	55/18670	2.26E-10	3.17E-09	1.26E-09	ESR1/SRC/	9
GO:00435!	regulatior	9/137	55/18670	2.26E-10	3.17E-09	1.26E-09	SRC/PDGFI	9
GO:00305:	intracellu	16/137	280/1867(	2.49E-10	3.48E-09	1.38E-09	AR/ESR1/I	16
GO:00108:	regulatior	10/137	78/18670	2.67E-10	3.70E-09	1.47E-09	MAPK14/TI	10
GO:00606{	regulatior	9/137	56/18670	2.68E-10	3.70E-09	1.47E-09	AR/ESR1/I	9
GO:19030	positive r	9/137	56/18670	2.68E-10	3.70E-09	1.47E-09	LGALS3/E(	9
GO:00427	signal tra	12/137	133/1867(	2.70E-10	3.72E-09	1.48E-09	MAPK14/CI	12
GO:00600⁴	positive r	8/137	38/18670	2.78E-10	3.82E-09	1.52E-09	MAPK14/M/	8
GO:19037(	regulatior	20/137	475/1867(	2.89E-10	3.95E-09	1.57E-09	MAPK14/S1	20
GO:20002(	regulatior	7/137	24/18670	3.06E-10	4.18E-09	1.66E-09	SRC/BCL2/	7
GO:00509:	positive r	12/137	135/1867(	3.21E-10	4.37E-09	1.74E-09	MAPK14/M/	12
GO:00421:	neurotrans	11/137	106/1867(	3.40E-10	4.61E-09	1.84E-09	NOS2/MTOI	11
GO:00342{	response t	14/137	207/1867(	3.90E-10	5.27E-09	2.10E-09	IGF1R/PII	14
GO:00972:	cellular r	15/137	247/1867(	4.03E-10	5.43E-09	2.16E-09	ABCG2/SR(	15
GO:00085{	male gonac	12/137	138/1867(	4.15E-10	5.57E-09	2.22E-09	AR/ESR1/1	12
GO:00062	regulatior	11/137	108/1867(	4.17E-10	5.58E-09	2.22E-09	FGFR1/CDI	11
GO:00313⁴	positive r	18/137	383/1867(	4.22E-10	5.62E-09	2.24E-09	TGFBR1/M/	18
GO:00604⁴	branching	7/137	25/18670	4.23E-10	5.62E-09	2.24E-09	AR/ESR1/\	7
GO:19019!	negative r	15/137	248/1867(	4.26E-10	5.65E-09	2.25E-09	BCL2/CDK1	15
GO:00465⁴	developmer	12/137	139/1867(	4.51E-10	5.96E-09	2.37E-09	AR/ESR1/1	12
GO:00426!	muscle cel	18/137	385/1867(	4.59E-10	6.04E-09	2.41E-09	MAPK14/DI	18
GO:00456:	regulatior	15/137	251/1867(	5.03E-10	6.61E-09	2.63E-09	MAPK14/S1	15
GO:00224(	positive r	15/137	255/1867(	6.27E-10	8.21E-09	3.27E-09	PTPN11/SI	15
GO:00432(	response t	11/137	113/1867(	6.81E-10	8.89E-09	3.54E-09	MMP2/DNM1	11
GO:00468:	positive r	9/137	62/18670	6.91E-10	8.99E-09	3.58E-09	MAPK14/G!	9
GO:00513{	response t	12/137	146/1867(	7.98E-10	1.03E-08	4.08E-09	BCL2/PTG!	12
GO:00454:	positive r	8/137	43/18670	7.99E-10	1.03E-08	4.08E-09	MTOR/PTG!	8
GO:00315	mitotic G1	9/137	63/18670	8.01E-10	1.03E-08	4.08E-09	CDK1/AURI	9
GO:00448	mitotic G1	9/137	63/18670	8.01E-10	1.03E-08	4.08E-09	CDK1/AURI	9
GO:19043	positive r	9/137	63/18670	8.01E-10	1.03E-08	4.08E-09	LGALS3/E(	9
GO:19018{	regulatior	10/137	88/18670	9.02E-10	1.15E-08	4.57E-09	SRC/KDR/I	10
GO:19037:	regulatior	10/137	88/18670	9.02E-10	1.15E-08	4.57E-09	SRC/PDGFI	10

GO:00345(	protein lo	15/137	262/1867(	9.12E-10	1.16E-08	4.61E-09	MAPK14/TI	15
GO:00300(	lymphocyte	17/137	353/1867(	9.24E-10	1.16E-08	4.62E-09	MMP14/KI1	17
GO:00327(	regulation	9/137	64/18670	9.26E-10	1.16E-08	4.62E-09	VDR/TERT/	9
GO:00435!	regulation	9/137	64/18670	9.26E-10	1.16E-08	4.62E-09	SRC/PDGFI	9
GO:00447{	G1 DNA dan	9/137	64/18670	9.26E-10	1.16E-08	4.62E-09	CDK1/AURI	9
GO:00315{	cell-subst	17/137	354/1867(	9.65E-10	1.21E-08	4.80E-09	SRC/KDR/(	17
GO:19044(	positive r	8/137	44/18670	9.71E-10	1.21E-08	4.82E-09	MTOR/PTGS	8
GO:19037(	positive r	13/137	185/1867(	1.07E-09	1.32E-08	5.28E-09	MAPK14/S1	13
GO:19907	protein lo	16/137	311/1867(	1.15E-09	1.43E-08	5.69E-09	AR/MMP14/	16
GO:00454	regulation	9/137	66/18670	1.23E-09	1.52E-08	6.05E-09	MTOR/PTGS	9
GO:19019(	positive r	10/137	91/18670	1.26E-09	1.55E-08	6.18E-09	TERT/CDK1	10
GO:00109	negative r	17/137	361/1867(	1.30E-09	1.59E-08	6.33E-09	BCL2/CHEI	17
GO:00313	positive r	17/137	361/1867(	1.30E-09	1.59E-08	6.33E-09	MAPK3/KDI	17
GO:00903	positive r	12/137	153/1867(	1.37E-09	1.67E-08	6.66E-09	MAPK14/GS	12
GO:00451(	cell fate	15/137	270/1867(	1.38E-09	1.68E-08	6.69E-09	AR/PPARG/	15
GO:00713	cellular r	8/137	46/18670	1.41E-09	1.71E-08	6.82E-09	PTPN11/S1	8
GO:00105(	regulation	14/137	229/1867(	1.46E-09	1.77E-08	7.05E-09	PPARG/KDI	14
GO:00712	cellular r	13/137	190/1867(	1.48E-09	1.78E-08	7.11E-09	MAPK3/MMI	13
GO:00015!	regulation	18/137	416/1867(	1.58E-09	1.90E-08	7.55E-09	ESR2/PPAI	18
GO:20008	negative r	6/137	17/18670	1.62E-09	1.94E-08	7.74E-09	SRC/BCL2/	6
GO:00974	liver rege	7/137	30/18670	1.74E-09	2.08E-08	8.27E-09	EGFR/AURI	7
GO:00507(	regulation	19/137	472/1867(	1.76E-09	2.11E-08	8.39E-09	NOS2/MAPI	19
GO:00140	regulation	11/137	124/1867(	1.85E-09	2.20E-08	8.76E-09	PPARG/TEI	11
GO:00603	face devel	8/137	48/18670	2.02E-09	2.39E-08	9.50E-09	MAP2K1/M/	8
GO:19037	positive r	8/137	48/18670	2.02E-09	2.39E-08	9.50E-09	SRC/PDGFI	8
GO:00017(	in utero e	17/137	373/1867(	2.13E-09	2.51E-08	1.00E-08	AR/TGFBR1	17
GO:00525	regulation	18/137	425/1867(	2.21E-09	2.60E-08	1.04E-08	PPARG/SR(	18
GO:00466(	male sex c	12/137	160/1867(	2.29E-09	2.69E-08	1.07E-08	AR/ESR1/1	12
GO:00350(	response t	8/137	49/18670	2.40E-09	2.80E-08	1.12E-08	MAPK1/BCI	8
GO:00105(	positive r	11/137	128/1867(	2.60E-09	3.03E-08	1.21E-08	KDR/MET/I	11
GO:00316	regulation	15/137	284/1867(	2.75E-09	3.19E-08	1.27E-08	TERT/SRC/	15
GO:00507	regulation	19/137	485/1867(	2.76E-09	3.19E-08	1.27E-08	ESR1/NOS2	19
GO:00512	regulation	19/137	485/1867(	2.76E-09	3.19E-08	1.27E-08	PTPN11/SI	19
GO:00707	response t	8/137	50/18670	2.83E-09	3.26E-08	1.30E-08	PTPN11/S1	8
GO:00425(	glucose ho	14/137	241/1867(	2.83E-09	3.26E-08	1.30E-08	PPARG/PTI	14
GO:00335(	carbohydra	14/137	242/1867(	2.99E-09	3.43E-08	1.37E-08	PPARG/PTI	14
GO:00097	response t	13/137	202/1867(	3.12E-09	3.57E-08	1.42E-08	IGF1R/PII	13
GO:00506{	negative r	12/137	165/1867(	3.25E-09	3.71E-08	1.48E-08	AR/VDR/PI	12
GO:00302	regulation	13/137	203/1867(	3.31E-09	3.77E-08	1.50E-08	MAPK14/M/	13
GO:19034	regulation	10/137	101/1867(	3.54E-09	4.02E-08	1.60E-08	MTOR/PTGS	10
GO:00480	neurotroph	7/137	33/18670	3.58E-09	4.05E-08	1.61E-08	PTPN11/SI	7
GO:00000	regulation	10/137	102/1867(	3.90E-09	4.40E-08	1.75E-08	SRC/EGFR/	10
GO:00094	response t	15/137	292/1867(	4.02E-09	4.52E-08	1.80E-08	PPARG/MT(	15
GO:00300	insulin se	13/137	207/1867(	4.20E-09	4.71E-08	1.88E-08	NOS2/PTPI	13
GO:00511	striated m	15/137	293/1867(	4.21E-09	4.71E-08	1.88E-08	MAPK14/PI	15
GO:00303(	positive r	12/137	169/1867(	4.27E-09	4.76E-08	1.89E-08	TGFBR1/MI	12
GO:00380(	Fc-epsilor	12/137	169/1867(	4.27E-09	4.76E-08	1.89E-08	MAPK3/MAI	12

GO:00109	regulatior	19/137	499/1867(	4.39E-09	4.88E-08	1.94E-08	MAP2K1/GS	19
GO:00902	regulatior	13/137	208/1867(	4.45E-09	4.92E-08	1.96E-08	NOS2/PTPN	13
GO:19012	negative r	13/137	208/1867(	4.45E-09	4.92E-08	1.96E-08	TERT/GSK3	13
GO:00027	regulatior	19/137	500/1867(	4.54E-09	4.99E-08	1.99E-08	NOS2/MAPK	19
GO:00300	peptide hc	14/137	250/1867(	4.54E-09	4.99E-08	1.99E-08	NOS2/PTPN	14
GO:00060	glucose me	13/137	209/1867(	4.72E-09	5.15E-08	2.05E-08	MAPK14/SE	13
GO:00086	intrinsic	10/137	104/1867(	4.72E-09	5.15E-08	2.05E-08	BCL2/BCL2	10
GO:00468	regulatior	10/137	104/1867(	4.72E-09	5.15E-08	2.05E-08	MAPK14/PT	10
GO:00018	maternal p	7/137	35/18670	5.56E-09	6.05E-08	2.41E-08	VDR/CTSB/	7
GO:00447	mitotic DN	10/137	106/1867(	5.69E-09	6.16E-08	2.45E-08	CDK1/AURK	10
GO:19040	regulatior	10/137	106/1867(	5.69E-09	6.16E-08	2.45E-08	SRC/EGFR/	10
GO:00507	regulatior	14/137	256/1867(	6.15E-09	6.64E-08	2.64E-08	PTPN11/SE	14
GO:00165	histone mc	18/137	454/1867(	6.21E-09	6.69E-08	2.66E-08	MAPK3/DNM	18
GO:00435	positive r	9/137	79/18670	6.31E-09	6.77E-08	2.70E-08	KDR/FGFR1	9
GO:00018	epithelial	11/137	140/1867(	6.71E-09	7.19E-08	2.86E-08	TGFBR1/GS	11
GO:00507	regulatior	12/137	176/1867(	6.76E-09	7.23E-08	2.88E-08	NOS2/PTPN	12
GO:19046	glucose tr	10/137	108/1867(	6.83E-09	7.28E-08	2.90E-08	MAPK14/TE	10
GO:19010	negative r	7/137	36/18670	6.86E-09	7.28E-08	2.90E-08	TERT/BCL2	7
GO:20012	negative r	7/137	36/18670	6.86E-09	7.28E-08	2.90E-08	TERT/BCL2	7
GO:00709	regulatior	6/137	21/18670	6.93E-09	7.33E-08	2.92E-08	ESR1/MAP2	6
GO:00069	DNA damage	8/137	56/18670	7.23E-09	7.60E-08	3.03E-08	CDK1/AURK	8
GO:00103	response t	8/137	56/18670	7.23E-09	7.60E-08	3.03E-08	BCL2/BCL2	8
GO:00509	regulatior	13/137	217/1867(	7.42E-09	7.79E-08	3.10E-08	MAPK14/MA	13
GO:00726	protein lc	14/137	260/1867(	7.50E-09	7.85E-08	3.13E-08	AR/MMP14/	14
GO:00070	cell-subst	9/137	81/18670	7.90E-09	8.20E-08	3.27E-08	SRC/KDR/M	9
GO:00480	focal adhe	9/137	81/18670	7.90E-09	8.20E-08	3.27E-08	SRC/KDR/M	9
GO:01101	positive r	9/137	81/18670	7.90E-09	8.20E-08	3.27E-08	AR/VDR/TC	9
GO:20012	positive r	12/137	179/1867(	8.19E-09	8.48E-08	3.38E-08	TGFBR1/GS	12
GO:00020	columnar/c	8/137	57/18670	8.35E-09	8.57E-08	3.41E-08	GSK3B/FGF	8
GO:00724	signal tra	8/137	57/18670	8.35E-09	8.57E-08	3.41E-08	CDK1/AURK	8
GO:19024	intracellu	8/137	57/18670	8.35E-09	8.57E-08	3.41E-08	CDK1/AURK	8
GO:19034	positive r	8/137	57/18670	8.35E-09	8.57E-08	3.41E-08	MTOR/PTGS	8
GO:00712	cellular r	7/137	37/18670	8.41E-09	8.61E-08	3.43E-08	MAPK3/MMF	7
GO:00435	blood vess	12/137	180/1867(	8.72E-09	8.90E-08	3.54E-08	PPARG/KDF	12
GO:00711	positive r	9/137	82/18670	8.81E-09	8.98E-08	3.58E-08	CDK1/AURK	9
GO:00423	vitamin D	6/137	22/18670	9.47E-09	9.63E-08	3.83E-08	VDR/FGFR1	6
GO:00316	lipopolysa	8/137	58/18670	9.63E-09	9.76E-08	3.89E-08	MAPK14/MA	8
GO:00020	columnar/c	10/137	112/1867(	9.74E-09	9.83E-08	3.91E-08	GSK3B/FGF	10
GO:00086	hexose tra	10/137	112/1867(	9.74E-09	9.83E-08	3.91E-08	MAPK14/TE	10
GO:00346	response t	15/137	312/1867(	9.82E-09	9.86E-08	3.93E-08	MAPK14/MA	15
GO:00468	hormone se	15/137	312/1867(	9.82E-09	9.86E-08	3.93E-08	NOS2/PTPN	15
GO:00468	regulatior	14/137	266/1867(	1.00E-08	1.00E-07	4.00E-08	NOS2/PTPN	14
GO:00016	eye develc	16/137	362/1867(	1.01E-08	1.01E-07	4.02E-08	TGFBR1/RE	16
GO:00508	regulatior	15/137	314/1867(	1.07E-08	1.07E-07	4.25E-08	PTPN11/SE	15
GO:00316	cellular r	14/137	268/1867(	1.10E-08	1.09E-07	4.35E-08	VDR/PPARC	14
GO:00724	signal tra	8/137	59/18670	1.11E-08	1.09E-07	4.35E-08	CDK1/AURK	8
GO:19024	signal tra	8/137	59/18670	1.11E-08	1.09E-07	4.35E-08	CDK1/AURK	8

GO:19024(	signal tra	8/137	59/18670	1.11E-08	1.09E-07	4.35E-08	CDK1/AURI	8
GO:00434(	steroid hc	12/137	184/1867(	1.12E-08	1.10E-07	4.38E-08	AR/ESR1/I	12
GO:00331	regulatior	13/137	225/1867(	1.15E-08	1.13E-07	4.48E-08	MAPK14/PT	13
GO:00157	monosacche	10/137	114/1867(	1.16E-08	1.13E-07	4.52E-08	MAPK14/TI	10
GO:01500(	visual sys	16/137	366/1867(	1.18E-08	1.15E-07	4.59E-08	TGFBR1/RI	16
GO:00165(	covalent c	18/137	474/1867(	1.21E-08	1.18E-07	4.70E-08	MAPK3/DNI	18
GO:00381	neurotropl	7/137	39/18670	1.24E-08	1.21E-07	4.81E-08	PTPN11/SI	7
GO:00518	regulatior	8/137	60/18670	1.27E-08	1.23E-07	4.89E-08	SRC/KDR/I	8
GO:00901(	regulatior	8/137	60/18670	1.27E-08	1.23E-07	4.89E-08	SRC/KDR/I	8
GO:19028(	positive r	8/137	60/18670	1.27E-08	1.23E-07	4.89E-08	TERT/EGFI	8
GO:00069(	phagocytos	16/137	369/1867(	1.32E-08	1.28E-07	5.08E-08	PPARG/MAI	16
GO:19021(	regulatior	14/137	272/1867(	1.33E-08	1.28E-07	5.10E-08	MMP14/MT(	14
GO:00217	glial cell	10/137	116/1867(	1.37E-08	1.31E-07	5.22E-08	EGFR/CDK(	10
GO:00342	carbohydra	10/137	116/1867(	1.37E-08	1.31E-07	5.22E-08	MAPK14/TI	10
GO:00098	positive r	17/137	423/1867(	1.39E-08	1.32E-07	5.27E-08	MAPK3/KDI	17
GO:00323	regulatior	17/137	423/1867(	1.39E-08	1.32E-07	5.27E-08	MAPK14/MA	17
GO:00614	connective	14/137	273/1867(	1.39E-08	1.33E-07	5.28E-08	MAPK14/T(	14
GO:00488	sensory sy	16/137	371/1867(	1.43E-08	1.36E-07	5.40E-08	TGFBR1/RI	16
GO:00062(	DNA replic	14/137	274/1867(	1.46E-08	1.38E-07	5.51E-08	TOP1/FGFI	14
GO:00099	hormone tr	15/137	322/1867(	1.50E-08	1.42E-07	5.63E-08	NOS2/PTPI	15
GO:00466	decidualiz	6/137	24/18670	1.69E-08	1.59E-07	6.34E-08	VDR/CTSB/	6
GO:00421	neurotrans	11/137	153/1867(	1.70E-08	1.60E-07	6.38E-08	NOS2/MTOI	11
GO:00018	kidney dev	14/137	278/1867(	1.75E-08	1.64E-07	6.54E-08	TGFBR1/RI	14
GO:00071	epidermal	10/137	119/1867(	1.75E-08	1.64E-07	6.54E-08	PTPN11/SI	10
GO:00507	positive r	12/137	192/1867(	1.80E-08	1.68E-07	6.69E-08	PTPN11/SI	12
GO:00097	hormone-me	13/137	235/1867(	1.92E-08	1.79E-07	7.14E-08	AR/ESR1/I	13
GO:00343	adherens j	9/137	90/18670	2.02E-08	1.88E-07	7.49E-08	SRC/KDR/I	9
GO:00309(	forebrain	16/137	381/1867(	2.07E-08	1.92E-07	7.66E-08	SRC/GSK3I	16
GO:00308	regulatior	11/137	156/1867(	2.08E-08	1.93E-07	7.67E-08	VDR/GSK3I	11
GO:00435	regulatior	11/137	156/1867(	2.08E-08	1.93E-07	7.67E-08	PPARG/KDI	11
GO:00316(	cellular r	13/137	237/1867(	2.13E-08	1.96E-07	7.81E-08	VDR/PPAR(	13
GO:00020	positive r	6/137	25/18670	2.21E-08	2.03E-07	8.07E-08	STAT1/FGI	6
GO:00033(	type B par	6/137	25/18670	2.21E-08	2.03E-07	8.07E-08	GSK3B/HNI	6
GO:00456	positive r	9/137	91/18670	2.23E-08	2.04E-07	8.13E-08	MAPK14/ST	9
GO:00097	response t	12/137	197/1867(	2.39E-08	2.19E-07	8.71E-08	IGF1R/PII	12
GO:19016	cellular r	9/137	93/18670	2.70E-08	2.46E-07	9.81E-08	AR/PPARG/	9
GO:00071(	negative r	14/137	289/1867(	2.86E-08	2.60E-07	1.03E-07	PTPN11/SI	14
GO:00092(	response t	13/137	243/1867(	2.86E-08	2.60E-07	1.03E-07	PPARG/MAI	13
GO:00343	cell junct	14/137	290/1867(	2.98E-08	2.70E-07	1.08E-07	TGFBR1/SI	14
GO:00108	positive r	7/137	44/18670	3.00E-08	2.71E-07	1.08E-07	MAPK14/TI	7
GO:19033	regulatior	8/137	67/18670	3.10E-08	2.80E-07	1.11E-07	SRC/KDR/I	8
GO:00508	positive r	12/137	202/1867(	3.16E-08	2.85E-07	1.13E-07	PTPN11/SI	12
GO:00059	monosaccha	14/137	292/1867(	3.25E-08	2.91E-07	1.16E-07	MAPK14/SI	14
GO:00902	regulatior	14/137	292/1867(	3.25E-08	2.91E-07	1.16E-07	TGFBR1/F(	14
GO:00508(	positive r	16/137	394/1867(	3.31E-08	2.96E-07	1.18E-07	PTPN11/SI	16
GO:00330	regulatior	15/137	342/1867(	3.33E-08	2.97E-07	1.18E-07	MAPK3/SR(	15
GO:00720(	renal syst	14/137	293/1867(	3.39E-08	3.02E-07	1.20E-07	TGFBR1/RI	14

GO:00359:	cellular r	8/137	68/18670	3.49E-08	3.10E-07	1.24E-07	MAPK14/KI	8
GO:00310	endocrine	7/137	45/18670	3.53E-08	3.13E-07	1.24E-07	GSK3B/HNI	7
GO:00485:	thymus dev	7/137	45/18670	3.53E-08	3.13E-07	1.24E-07	TGFBR1/MA	7
GO:00358	enteroendo	6/137	27/18670	3.65E-08	3.22E-07	1.28E-07	GSK3B/HNI	6
GO:20012	regulation	11/137	165/1867(	3.73E-08	3.29E-07	1.31E-07	SRC/MMP9/	11
GO:00483(	organelle	5/137	14/18670	3.75E-08	3.29E-07	1.31E-07	MAP2K1/MA	5
GO:00483	Golgi inhe	5/137	14/18670	3.75E-08	3.29E-07	1.31E-07	MAP2K1/MA	5
GO:00063	DNA-templa	13/137	249/1867(	3.81E-08	3.33E-07	1.33E-07	AR/ESR1/I	13
GO:00193	hexose met	13/137	249/1867(	3.81E-08	3.33E-07	1.33E-07	MAPK14/SI	13
GO:00016	ureteric b	9/137	97/18670	3.92E-08	3.40E-07	1.35E-07	RET/FGFR	9
GO:00070	cell-subst	9/137	97/18670	3.92E-08	3.40E-07	1.35E-07	SRC/KDR/M	9
GO:00447	mitotic DN	9/137	97/18670	3.92E-08	3.40E-07	1.35E-07	CDK1/AURI	9
GO:00061(	regulation	12/137	206/1867(	3.93E-08	3.40E-07	1.36E-07	SRC/GSK3I	12
GO:00020(	glandular	7/137	46/18670	4.14E-08	3.57E-07	1.42E-07	GSK3B/HNI	7
GO:00440(	modificati	7/137	46/18670	4.14E-08	3.57E-07	1.42E-07	BCL2L1/GA	7
GO:00350	cardiocyte	11/137	167/1867(	4.22E-08	3.64E-07	1.45E-07	MAPK3/MAI	11
GO:00721(	mesonephri	9/137	98/18670	4.29E-08	3.67E-07	1.46E-07	RET/FGFR	9
GO:00721(	mesonephri	9/137	98/18670	4.29E-08	3.67E-07	1.46E-07	RET/FGFR	9
GO:20012	negative r	9/137	98/18670	4.29E-08	3.67E-07	1.46E-07	SRC/MMP9/	9
GO:00301	B cell dif	10/137	131/1867(	4.41E-08	3.77E-07	1.50E-07	MMP14/KIT	10
GO:00616	pri-miRNA	7/137	47/18670	4.83E-08	4.11E-07	1.64E-07	PPARG/TEI	7
GO:00708	response t	7/137	47/18670	4.83E-08	4.11E-07	1.64E-07	MAPK3/PTI	7
GO:00302(	entry into	10/137	134/1867(	5.48E-08	4.62E-07	1.84E-07	CTSB/SRC/	10
GO:00444(	entry into	10/137	134/1867(	5.48E-08	4.62E-07	1.84E-07	CTSB/SRC/	10
GO:00518(	entry into	10/137	134/1867(	5.48E-08	4.62E-07	1.84E-07	CTSB/SRC/	10
GO:00518	entry into	10/137	134/1867(	5.48E-08	4.62E-07	1.84E-07	CTSB/SRC/	10
GO:00518	regulation	8/137	72/18670	5.52E-08	4.65E-07	1.85E-07	SRC/KDR/I	8
GO:20010	positive r	5/137	15/18670	5.60E-08	4.70E-07	1.87E-07	KDR/MET/I	5
GO:00224	cellular p	15/137	357/1867(	5.86E-08	4.91E-07	1.96E-07	TGFBR1/SI	15
GO:00072	JNK cascac	12/137	214/1867(	5.98E-08	5.00E-07	1.99E-07	IGF1R/HRA	12
GO:00423	negative r	17/137	468/1867(	6.05E-08	5.06E-07	2.01E-07	IGF1R/MT(	17
GO:00018	mesonephro	9/137	102/1867(	6.09E-08	5.08E-07	2.02E-07	RET/FGFR	9
GO:00323	positive r	12/137	215/1867(	6.29E-08	5.24E-07	2.08E-07	MAPK14/GS	12
GO:00098	negative r	14/137	308/1867(	6.31E-08	5.24E-07	2.09E-07	NOS2/MAPI	14
GO:00308	negative r	7/137	49/18670	6.52E-08	5.41E-07	2.15E-07	GSK3B/MMI	7
GO:00421	B cell act	14/137	310/1867(	6.84E-08	5.66E-07	2.25E-07	MMP14/KIT	14
GO:00024	immune res	17/137	473/1867(	7.05E-08	5.82E-07	2.32E-07	MAPK3/SR(	17
GO:00148	muscle cel	9/137	104/1867(	7.21E-08	5.94E-07	2.37E-07	TERT/SRC/	9
GO:00484(	cell matur	11/137	177/1867(	7.67E-08	6.31E-07	2.51E-07	PPARG/PGI	11
GO:00024	immune res	10/137	139/1867(	7.76E-08	6.35E-07	2.53E-07	MAPK3/SR(	10
GO:00380	Fc-gamma r	10/137	139/1867(	7.76E-08	6.35E-07	2.53E-07	MAPK3/SR(	10
GO:00430	camera-typ	14/137	314/1867(	8.02E-08	6.55E-07	2.61E-07	TGFBR1/RI	14
GO:00448	cell cycle	13/137	266/1867(	8.27E-08	6.74E-07	2.69E-07	ABCB1/CHI	13
GO:00720	kidney epi	10/137	140/1867(	8.30E-08	6.76E-07	2.69E-07	RET/STAT	10
GO:00510	positive r	11/137	179/1867(	8.60E-08	6.99E-07	2.78E-07	PPARG/TEI	11
GO:00509	regulation	7/137	51/18670	8.69E-08	7.04E-07	2.80E-07	TERT/HIF	7
GO:19028	positive r	6/137	31/18670	8.86E-08	7.17E-07	2.85E-07	TERT/HIF	6

GO:00507	positive r	13/137	268/18670	9.02E-08	7.29E-07	2.90E-07	MAPK14/MA	13
GO:190533	regulation	11/137	180/18670	9.10E-08	7.34E-07	2.92E-07	AR/ESR1/S	11
GO:005134	regulation	9/137	107/18670	9.24E-08	7.44E-07	2.96E-07	VDR/TERT/	9
GO:003809	Fc-gamma r	10/137	142/18670	9.49E-08	7.63E-07	3.04E-07	MAPK3/SRC	10
GO:200027	regulation	9/137	108/18670	1.00E-07	8.03E-07	3.20E-07	PPARG/MAP	9
GO:004887	multicellu	17/137	485/18670	1.01E-07	8.08E-07	3.22E-07	PTPN11/SH	17
GO:00310	pancreas d	8/137	78/18670	1.04E-07	8.32E-07	3.31E-07	MET/GSK3B	8
GO:000193	negative r	16/137	429/18670	1.07E-07	8.49E-07	3.38E-07	IGF1R/MTO	16
GO:000204	sprouting	11/137	183/18670	1.08E-07	8.57E-07	3.41E-07	KDR/VEGFA	11
GO:006064	mammary gl	5/137	17/18670	1.14E-07	9.03E-07	3.60E-07	HIF1A/AKT	5
GO:200064	regulation	5/137	17/18670	1.14E-07	9.03E-07	3.60E-07	MAP2K1/MA	5
GO:000018	activation	7/137	53/18670	1.14E-07	9.04E-07	3.60E-07	TGFBR1/MA	7
GO:000157	vasculogen	8/137	79/18670	1.15E-07	9.10E-07	3.62E-07	KDR/PDGFB	8
GO:000243	Fc recepto	10/137	145/18670	1.16E-07	9.11E-07	3.63E-07	MAPK3/SRC	10
GO:004632	regulation	11/137	185/18670	1.20E-07	9.47E-07	3.77E-07	IGF1R/HRA	11
GO:003520	multicellu	10/137	146/18670	1.23E-07	9.69E-07	3.86E-07	AR/PTPN11	10
GO:19040	epithelial	9/137	111/18670	1.27E-07	9.95E-07	3.96E-07	TERT/KDR/	9
GO:00096	response t	14/137	326/18670	1.27E-07	9.95E-07	3.96E-07	MAPK14/SH	14
GO:005099	regulation	7/137	54/18670	1.30E-07	1.02E-06	4.05E-07	MTOR/TNF/	7
GO:190464	response t	7/137	54/18670	1.30E-07	1.02E-06	4.05E-07	IGF1R/GSK	7
GO:000220	myeloid ce	10/137	147/18670	1.32E-07	1.02E-06	4.05E-07	MAPK14/ST	10
GO:004352	negative r	10/137	147/18670	1.32E-07	1.02E-06	4.05E-07	TERT/BCL2	10
GO:001003	response t	6/137	33/18670	1.32E-07	1.02E-06	4.05E-07	BCL2/HIF1	6
GO:001040	regulation	6/137	33/18670	1.32E-07	1.02E-06	4.05E-07	STAT1/FGF	6
GO:005080	modulation	16/137	436/18670	1.33E-07	1.03E-06	4.08E-07	CA7/SRC/C	16
GO:006024	anatomical	16/137	437/18670	1.37E-07	1.05E-06	4.19E-07	TERT/MAPK	16
GO:009917	regulation	16/137	437/18670	1.37E-07	1.05E-06	4.19E-07	CA7/SRC/C	16
GO:004327	response t	9/137	112/18670	1.37E-07	1.05E-06	4.19E-07	PPARG/MTO	9
GO:00518	modificati	9/137	112/18670	1.37E-07	1.05E-06	4.19E-07	BCL2L1/GA	9
GO:000864	carbohydra	10/137	148/18670	1.40E-07	1.07E-06	4.27E-07	MAPK14/TH	10
GO:000630	transcript	11/137	188/18670	1.42E-07	1.08E-06	4.31E-07	AR/ESR1/H	11
GO:005109	positive r	10/137	149/18670	1.49E-07	1.14E-06	4.53E-07	AR/STAT3/	10
GO:001097	positive r	13/137	281/18670	1.56E-07	1.19E-06	4.73E-07	MAP2K1/RH	13
GO:003599	response t	5/137	18/18670	1.57E-07	1.19E-06	4.73E-07	MAPK14/JU	5
GO:006074	mammary gl	5/137	18/18670	1.57E-07	1.19E-06	4.73E-07	AR/ESR1/V	5
GO:006137	mammary gl	5/137	18/18670	1.57E-07	1.19E-06	4.73E-07	AR/ESR1/V	5
GO:00302	erythrocyt	9/137	114/18670	1.60E-07	1.21E-06	4.81E-07	MAPK14/ST	9
GO:006050	developmen	12/137	235/18670	1.66E-07	1.25E-06	4.98E-07	ESR1/GSK3	12
GO:003129	T cell cos	7/137	56/18670	1.69E-07	1.27E-06	5.05E-07	PTPN11/SH	7
GO:009018	regulation	7/137	56/18670	1.69E-07	1.27E-06	5.05E-07	RET/MMP9/	7
GO:005125	positive r	14/137	334/18670	1.71E-07	1.28E-06	5.11E-07	PTPN11/SH	14
GO:002170	developmen	13/137	284/18670	1.77E-07	1.32E-06	5.26E-07	PPARG/PGF	13
GO:001050	negative r	8/137	84/18670	1.87E-07	1.39E-06	5.53E-07	MET/BCL2/	8
GO:00149	regulation	8/137	84/18670	1.87E-07	1.39E-06	5.53E-07	TERT/SRC/	8
GO:004315	negative r	8/137	84/18670	1.87E-07	1.39E-06	5.53E-07	SRC/MMP9/	8
GO:003129	lymphocyte	7/137	57/18670	1.91E-07	1.42E-06	5.65E-07	PTPN11/SH	7
GO:004352	positive r	7/137	57/18670	1.91E-07	1.42E-06	5.65E-07	TNF/CASP3	7

GO:00072(	Ras protei	16/137	448/1867(	1.92E-07	1.42E-06	5.66E-07	MAPK14/MI	16
GO:00027(	regulatior	9/137	117/1867(	2.00E-07	1.48E-06	5.89E-07	MTOR/PRK(	9
GO:00027!	positive ı	13/137	288/1867(	2.07E-07	1.53E-06	6.09E-07	MAPK14/MA	13
GO:19037!	regulatior	5/137	19/18670	2.12E-07	1.56E-06	6.21E-07	ESR1/MAP2	5
GO:00718!	DNA biosyr	11/137	196/1867(	2.16E-07	1.58E-06	6.31E-07	PPARG/TEI	11
GO:00619(	glial cell	7/137	58/18670	2.16E-07	1.58E-06	6.31E-07	EGFR/TNF/	7
GO:00609(	regulatior	10/137	155/1867(	2.16E-07	1.58E-06	6.31E-07	ESR1/PPAI	10
GO:00343:	cell junct	12/137	241/1867(	2.18E-07	1.59E-06	6.35E-07	SRC/KDR/N	12
GO:00705	response t	8/137	86/18670	2.24E-07	1.64E-06	6.51E-07	PPARG/SR(	8
GO:00905!	regulatior	8/137	86/18670	2.24E-07	1.64E-06	6.51E-07	GSK3B/BCI	8
GO:00107	macrophage	6/137	36/18670	2.27E-07	1.65E-06	6.57E-07	PPARG/PLA	6
GO:00900	foam cell	6/137	36/18670	2.27E-07	1.65E-06	6.57E-07	PPARG/PLA	6
GO:00713!	cellular ı	13/137	291/1867(	2.34E-07	1.69E-06	6.74E-07	MAPK14/MA	13
GO:00018!	embryonic	8/137	87/18670	2.46E-07	1.78E-06	7.08E-07	MAP2K1/MA	8
GO:19035:	positive ı	15/137	399/1867(	2.47E-07	1.79E-06	7.11E-07	MAPK14/MA	15
GO:00720	stem cell	9/137	120/1867(	2.49E-07	1.79E-06	7.14E-07	TERT/ABCI	9
GO:00434(	regulatior	10/137	158/1867(	2.59E-07	1.86E-06	7.41E-07	NOS2/GSK:	10
GO:00341(	regulatior	8/137	88/18670	2.69E-07	1.93E-06	7.69E-07	VDR/SRC/I	8
GO:00328	regulatior	6/137	37/18670	2.70E-07	1.93E-06	7.69E-07	GSK3B/MT(	6
GO:00423(	positive ı	6/137	37/18670	2.70E-07	1.93E-06	7.69E-07	MAPK14/MA	6
GO:00463:	regulatior	7/137	60/18670	2.74E-07	1.96E-06	7.81E-07	MAPK14/TI	7
GO:00033:	type B par	5/137	20/18670	2.81E-07	2.00E-06	7.95E-07	GSK3B/HNI	5
GO:00517(	nitric-oxi	5/137	20/18670	2.81E-07	2.00E-06	7.95E-07	KDR/STAT	5
GO:00517(	regulatior	5/137	20/18670	2.81E-07	2.00E-06	7.95E-07	KDR/STAT	5
GO:00508!	cognition	13/137	296/1867(	2.84E-07	2.01E-06	8.02E-07	MAPK1/KIT	13
GO:00000	G2/M trans	12/137	247/1867(	2.84E-07	2.01E-06	8.02E-07	ABCB1/HSI	12
GO:00341(	erythrocyt	9/137	122/1867(	2.86E-07	2.03E-06	8.08E-07	MAPK14/ST	9
GO:00230(	signal rel	16/137	462/1867(	2.90E-07	2.05E-06	8.17E-07	NOS2/PTPN	16
GO:00071	transmembı	14/137	349/1867(	2.92E-07	2.06E-06	8.20E-07	MAPK14/T(	14
GO:00486	animal or	7/137	61/18670	3.08E-07	2.17E-06	8.64E-07	AR/MAP2K	7
GO:00463:	positive ı	6/137	38/18670	3.18E-07	2.24E-06	8.91E-07	MAPK14/TI	6
GO:00107	regulatior	8/137	90/18670	3.20E-07	2.25E-06	8.94E-07	TGFBR1/M	8
GO:00086:	apoptotic	9/137	124/1867(	3.29E-07	2.30E-06	9.17E-07	GSK3B/MMI	9
GO:00511(	regulatior	9/137	124/1867(	3.29E-07	2.30E-06	9.17E-07	PPARG/MMI	9
GO:00215:	telencepha	12/137	251/1867(	3.37E-07	2.36E-06	9.39E-07	GSK3B/HII	12
GO:00149(	smooth mus	8/137	91/18670	3.49E-07	2.43E-06	9.68E-07	TERT/SRC/	8
GO:00075	muscle org	15/137	410/1867(	3.50E-07	2.43E-06	9.69E-07	MAPK14/T(	15
GO:00358:	modificati	10/137	164/1867(	3.66E-07	2.54E-06	1.01E-06	NOS2/BCL:	10
GO:00351(	embryonic	5/137	21/18670	3.66E-07	2.54E-06	1.01E-06	KDR/KIT/V	5
GO:00190	modulatior	6/137	39/18670	3.74E-07	2.59E-06	1.03E-06	BCL2L1/CA	6
GO:19045!	positive ı	6/137	39/18670	3.74E-07	2.59E-06	1.03E-06	MAPK14/MA	6
GO:20001	negative ı	8/137	92/18670	3.79E-07	2.62E-06	1.04E-06	SRC/MMP9/	8
GO:00716!	anatomical	10/137	165/1867(	3.87E-07	2.67E-06	1.06E-06	PGR/MMP2/	10
GO:00512	cartilage	11/137	209/1867(	4.11E-07	2.83E-06	1.13E-06	MAPK14/T(	11
GO:00364	cell deatl	8/137	93/18670	4.13E-07	2.83E-06	1.13E-06	MET/BCL2/	8
GO:00076	learning (	12/137	256/1867(	4.17E-07	2.86E-06	1.14E-06	MAPK1/KIT	12
GO:00026	positive ı	9/137	128/1867(	4.31E-07	2.95E-06	1.18E-06	MAPK14/MA	9

GO:00430(	extracellu	15/137	422/1867(	5.04E-07	3.45E-06	1.37E-06	TGFBR1/KI	15
GO:01500'	regulatior	6/137	41/18670	5.09E-07	3.47E-06	1.38E-06	MMP9/MMP:	6
GO:19028!	regulatior	6/137	41/18670	5.09E-07	3.47E-06	1.38E-06	TERT/HIF:	6
GO:00466:	regulatior	7/137	66/18670	5.33E-07	3.63E-06	1.44E-06	PTPN11/SI	7
GO:00301!	extracellu	14/137	368/1867(	5.52E-07	3.75E-06	1.49E-06	TGFBR1/KI	14
GO:00305:	intracellu	9/137	132/1867(	5.60E-07	3.80E-06	1.51E-06	AR/ESR1/I	9
GO:19038(	positive r	4/137	10/18670	5.63E-07	3.81E-06	1.52E-06	MAP2K1/E(	4
GO:00215	pallium de	10/137	172/1867(	5.68E-07	3.84E-06	1.53E-06	GSK3B/HII	10
GO:00303:	osteoclast	8/137	97/18670	5.71E-07	3.86E-06	1.54E-06	MAPK14/SI	8
GO:20005'	positive r	7/137	67/18670	5.92E-07	3.99E-06	1.59E-06	MAPK3/SR(	7
GO:20006:	positive r	5/137	23/18670	5.98E-07	4.02E-06	1.60E-06	MAP2K1/S'	5
GO:00510	positive r	15/137	428/1867(	6.02E-07	4.04E-06	1.61E-06	MAPK14/M/	15
GO:00456(	positive r	14/137	371/1867(	6.09E-07	4.08E-06	1.62E-06	MAP2K1/RI	14
GO:00723:	signal tra	12/137	267/1867(	6.52E-07	4.36E-06	1.74E-06	MAPK14/B(	12
GO:00435	ear develc	11/137	219/1867(	6.54E-07	4.37E-06	1.74E-06	MAPK3/PTI	11
GO:00336:	cell adhes	7/137	68/18670	6.56E-07	4.37E-06	1.74E-06	PTPN11/RI	7
GO:00463:	glucose in	7/137	68/18670	6.56E-07	4.37E-06	1.74E-06	MAPK14/TI	7
GO:00067'	fat-solubl	6/137	43/18670	6.82E-07	4.52E-06	1.80E-06	VDR/FGFR:	6
GO:00086:	intrinsic	6/137	43/18670	6.82E-07	4.52E-06	1.80E-06	BCL2/HIF:	6
GO:00328	regulatior	6/137	43/18670	6.82E-07	4.52E-06	1.80E-06	GSK3B/MT(	6
GO:00072!	female gan	9/137	136/1867(	7.21E-07	4.77E-06	1.90E-06	PGR/SRC/I	9
GO:00019!	regulatior	10/137	177/1867(	7.38E-07	4.88E-06	1.94E-06	PPARG/PTI	10
GO:00601	positive r	5/137	24/18670	7.51E-07	4.94E-06	1.97E-06	MAP2K1/S'	5
GO:19036	regulatior	5/137	24/18670	7.51E-07	4.94E-06	1.97E-06	MAP2K1/M/	5
GO:20010:	regulatior	5/137	24/18670	7.51E-07	4.94E-06	1.97E-06	KDR/MET/I	5
GO:00454	fat cell c	11/137	223/1867(	7.82E-07	5.14E-06	2.04E-06	PPARG/MAI	11
GO:00104(	mesenchyma	6/137	44/18670	7.85E-07	5.14E-06	2.05E-06	STAT1/FGI	6
GO:00302:	macrophage	6/137	44/18670	7.85E-07	5.14E-06	2.05E-06	MMP9/VEGI	6
GO:00026!	positive r	14/137	380/1867(	8.10E-07	5.29E-06	2.11E-06	PTPN11/SI	14
GO:00421'	regulatior	14/137	381/1867(	8.35E-07	5.45E-06	2.17E-06	NOS2/GSK:	14
GO:00016	osteoblast	11/137	225/1867(	8.54E-07	5.56E-06	2.22E-06	IGFBP3/EI	11
GO:00714(	cellular r	10/137	180/1867(	8.61E-07	5.60E-06	2.23E-06	HNF4A/EGI	10
GO:19017!	regulatior	10/137	180/1867(	8.61E-07	5.60E-06	2.23E-06	MAPK14/B(	10
GO:00604:	trachea mc	4/137	11/18670	8.80E-07	5.69E-06	2.27E-06	MAP2K1/M/	4
GO:00701(	interleuki	4/137	11/18670	8.80E-07	5.69E-06	2.27E-06	STAT1/ST/	4
GO:00707!	interleuki	4/137	11/18670	8.80E-07	5.69E-06	2.27E-06	STAT1/ST/	4
GO:00510!	positive r	7/137	71/18670	8.83E-07	5.70E-06	2.27E-06	SRC/PDGFI	7
GO:00020(	glandular	5/137	25/18670	9.34E-07	6.02E-06	2.40E-06	GSK3B/HNI	5
GO:00108:	regulatior	10/137	182/1867(	9.53E-07	6.13E-06	2.44E-06	KDR/GSK3I	10
GO:00607:	labyrinthi	6/137	46/18670	1.03E-06	6.61E-06	2.63E-06	MAP2K1/M/	6
GO:00106'	regulatior	9/137	142/1867(	1.04E-06	6.64E-06	2.64E-06	SRC/GSK3I	9
GO:00343:	adherens	9/137	142/1867(	1.04E-06	6.64E-06	2.64E-06	SRC/KDR/I	9
GO:00456	regulatior	7/137	73/18670	1.07E-06	6.84E-06	2.72E-06	PPARG/MT(	7
GO:00310!	regulatior	9/137	143/1867(	1.10E-06	7.01E-06	2.79E-06	MAPK3/DNI	9
GO:00443	cellular r	9/137	143/1867(	1.10E-06	7.01E-06	2.79E-06	MAPK3/PTI	9
GO:00073(	gastrulati	10/137	185/1867(	1.11E-06	7.04E-06	2.80E-06	MMP2/MMP!	10
GO:00713:	cellular r	9/137	144/1867(	1.17E-06	7.40E-06	2.95E-06	IGF1R/PII	9

GO:19021(	positive r	9/137	144/1867(	1.17E-06	7.40E-06	2.95E-06	MMP14/PRI	9
GO:00140	positive r	7/137	74/18670	1.17E-06	7.43E-06	2.96E-06	PPARG/MT(	7
GO:19000	regulatior	7/137	74/18670	1.17E-06	7.43E-06	2.96E-06	PTPN11/SI	7
GO:00075	blood coag	13/137	336/1867(	1.19E-06	7.50E-06	2.99E-06	MAPK3/PTI	13
GO:00450	regulatior	15/137	452/1867(	1.19E-06	7.52E-06	2.99E-06	ESR1/PPAI	15
GO:00303	DNA damage	8/137	107/1867(	1.21E-06	7.63E-06	3.04E-06	CDK1/AURI	8
GO:00513	negative r	12/137	285/1867(	1.30E-06	8.15E-06	3.25E-06	PPARG/SR(	12
GO:00070	mitotic nu	4/137	12/18670	1.31E-06	8.22E-06	3.27E-06	CDK1/PRK(	4
GO:00106	programmec	4/137	12/18670	1.31E-06	8.22E-06	3.27E-06	KIT/BCL2/	4
GO:00423	vitamin D	4/137	12/18670	1.31E-06	8.22E-06	3.27E-06	VDR/TNF/N	4
GO:00519	regulatior	6/137	48/18670	1.33E-06	8.33E-06	3.32E-06	PPARG/MAI	6
GO:00442	cellular c	12/137	286/1867(	1.34E-06	8.39E-06	3.34E-06	SRC/GSK3I	12
GO:00217	limbic sys	8/137	109/1867(	1.40E-06	8.70E-06	3.46E-06	GSK3B/FGI	8
GO:00075	hemostasis	13/137	341/1867(	1.40E-06	8.71E-06	3.47E-06	MAPK3/PTI	13
GO:00468	regulatior	5/137	27/18670	1.40E-06	8.71E-06	3.47E-06	VDR/HIF1/	5
GO:00469	regulatior	7/137	76/18670	1.41E-06	8.73E-06	3.48E-06	GSK3B/BCI	7
GO:00607	regulatior	10/137	190/1867(	1.41E-06	8.73E-06	3.48E-06	PPARG/PTI	10
GO:00485	embryonic	12/137	288/1867(	1.45E-06	8.94E-06	3.56E-06	TGFBR1/M/	12
GO:00508	coagulatic	13/137	342/1867(	1.45E-06	8.94E-06	3.56E-06	MAPK3/PTI	13
GO:00329	regulatior	13/137	343/1867(	1.49E-06	9.22E-06	3.67E-06	TGFBR1/MI	13
GO:00485	digestive	6/137	49/18670	1.51E-06	9.30E-06	3.70E-06	BCL2/HIF1	6
GO:00486	response t	7/137	77/18670	1.54E-06	9.47E-06	3.77E-06	MAP2K1/B(	7
GO:00072	Notch sigr	10/137	193/1867(	1.62E-06	9.98E-06	3.97E-06	STAT1/MMI	10
GO:00717	response t	9/137	150/1867(	1.64E-06	1.01E-05	4.00E-06	MAPK3/PTI	9
GO:00720	nephron mc	7/137	78/18670	1.68E-06	1.03E-05	4.10E-06	STAT1/PD(	7
GO:00327	positive r	5/137	28/18670	1.70E-06	1.04E-05	4.13E-06	TERT/HIF1	5
GO:00107	positive r	6/137	50/18670	1.70E-06	1.04E-05	4.13E-06	TGFBR1/MT	6
GO:00604	lung morpł	6/137	50/18670	1.70E-06	1.04E-05	4.13E-06	MAP2K1/M/	6
GO:19000	positive r	6/137	50/18670	1.70E-06	1.04E-05	4.13E-06	TERT/EGFI	6
GO:00423	molting cy	8/137	112/1867(	1.71E-06	1.04E-05	4.14E-06	TERT/BCL2	8
GO:00426	hair cycle	8/137	112/1867(	1.71E-06	1.04E-05	4.14E-06	TERT/BCL2	8
GO:00439	regulatior	14/137	405/1867(	1.72E-06	1.04E-05	4.15E-06	NOS2/MAPI	14
GO:00016	cellular g	9/137	152/1867(	1.83E-06	1.11E-05	4.41E-06	IGF1R/PII	9
GO:19000	regulatior	7/137	79/18670	1.83E-06	1.11E-05	4.41E-06	MAPK3/GSI	7
GO:00026	regulatior	10/137	196/1867(	1.87E-06	1.13E-05	4.49E-06	MAPK14/M/	10
GO:00306	regulatior	4/137	13/18670	1.88E-06	1.13E-05	4.51E-06	VDR/TNF/N	4
GO:00331	regulatior	4/137	13/18670	1.88E-06	1.13E-05	4.51E-06	MAPK3/CCN	4
GO:00903	replicativ	4/137	13/18670	1.88E-06	1.13E-05	4.51E-06	TERT/CHEI	4
GO:00019	positive r	6/137	51/18670	1.92E-06	1.15E-05	4.58E-06	KDR/GSK3I	6
GO:00310	dsRNA proc	6/137	51/18670	1.92E-06	1.15E-05	4.58E-06	ESR1/MAP2	6
GO:00709	productior	6/137	51/18670	1.92E-06	1.15E-05	4.58E-06	ESR1/MAP2	6
GO:00301	platelet a	9/137	153/1867(	1.93E-06	1.15E-05	4.60E-06	MAPK3/PTI	9
GO:00109	positive r	10/137	197/1867(	1.95E-06	1.17E-05	4.64E-06	PPARG/MAI	10
GO:00085	fibroblast	8/137	114/1867(	1.96E-06	1.17E-05	4.64E-06	MAPK3/PTI	8
GO:00427	regulatior	8/137	114/1867(	1.96E-06	1.17E-05	4.64E-06	PPARG/GSI	8
GO:00226	extracellu	7/137	80/18670	2.00E-06	1.19E-05	4.72E-06	MMP2/MMP9	7
GO:00105	positive r	5/137	29/18670	2.04E-06	1.21E-05	4.80E-06	PTGS2/HII	5

GO:00440(	modulatior	5/137	29/18670	2.04E-06	1.21E-05	4.80E-06	BCL2L1/G/	5
GO:00701(	interleuki	5/137	29/18670	2.04E-06	1.21E-05	4.80E-06	PTPN11/S7	5
GO:00725	JAK-STAT c	9/137	156/1867(	2.27E-06	1.34E-05	5.34E-06	RET/STAT1	9
GO:00715(	cellular r	11/137	249/1867(	2.30E-06	1.36E-05	5.40E-06	TGFBR1/SI	11
GO:00108:	telomere m	7/137	82/18670	2.36E-06	1.39E-05	5.53E-06	TERT/MAPI	7
GO:00302'	negative r	7/137	82/18670	2.36E-06	1.39E-05	5.53E-06	BCL2/HIF1	7
GO:00108	positive r	8/137	117/1867(	2.38E-06	1.39E-05	5.55E-06	KDR/GSK3I	8
GO:00109(	regulatior	8/137	117/1867(	2.38E-06	1.39E-05	5.55E-06	SRC/GSK3I	8
GO:00601	regulatior	8/137	117/1867(	2.38E-06	1.39E-05	5.55E-06	ESR1/PPAI	8
GO:00609(	regulatior	8/137	117/1867(	2.38E-06	1.39E-05	5.55E-06	ESR1/PPAI	8
GO:00432'	negative r	9/137	157/1867(	2.39E-06	1.40E-05	5.57E-06	MMP9/BCL2	9
GO:00714	cellular r	5/137	30/18670	2.43E-06	1.42E-05	5.65E-06	BCL2L1/HI	5
GO:00430	regulatior	15/137	479/1867(	2.44E-06	1.42E-05	5.66E-06	GSK3B/MT(	15
GO:00069'	response t	7/137	83/18670	2.56E-06	1.49E-05	5.94E-06	ABCB1/PT(	7
GO:00513	positive r	6/137	54/18670	2.70E-06	1.57E-05	6.26E-06	VDR/TERT/	6
GO:00357(	endotheli	5/137	31/18670	2.88E-06	1.67E-05	6.64E-06	KDR/MET/I	5
GO:00434	regulatior	5/137	31/18670	2.88E-06	1.67E-05	6.64E-06	NOS2/PIK3	5
GO:00457	positive r	5/137	31/18670	2.88E-06	1.67E-05	6.64E-06	SRC/EGFR/	5
GO:00715	response t	11/137	255/1867(	2.89E-06	1.67E-05	6.65E-06	TGFBR1/SI	11
GO:00423(	regulatior	6/137	55/18670	3.02E-06	1.74E-05	6.93E-06	MAPK14/M/	6
GO:00488(	stem cell	11/137	257/1867(	3.12E-06	1.80E-05	7.15E-06	ESR1/MAPI	11
GO:00017'	leukocyte	7/137	86/18670	3.26E-06	1.87E-05	7.46E-06	BCL2/PIK3	7
GO:00030(	heart mor	11/137	259/1867(	3.36E-06	1.93E-05	7.69E-06	TGFBR1/M1	11
GO:00070	regulatior	9/137	164/1867(	3.43E-06	1.97E-05	7.83E-06	PDGFRB/CI	9
GO:00066	phospholi	14/137	430/1867(	3.45E-06	1.97E-05	7.86E-06	SRC/PLA2(	14
GO:00423(	fat-solubl	4/137	15/18670	3.56E-06	2.03E-05	8.08E-06	VDR/TNF/I	4
GO:00517'	positive r	4/137	15/18670	3.56E-06	2.03E-05	8.08E-06	KDR/STAT1	4
GO:19033	regulatior	4/137	15/18670	3.56E-06	2.03E-05	8.08E-06	MAP2K1/M/	4
GO:00713	cellular r	6/137	57/18670	3.73E-06	2.13E-05	8.47E-06	PPARG/SR(	6
GO:00976	STAT casca	9/137	166/1867(	3.79E-06	2.15E-05	8.58E-06	RET/STAT1	9
GO:19004(	regulatior	7/137	88/18670	3.80E-06	2.16E-05	8.59E-06	MET/MMP3/	7
GO:19040:	regulatior	7/137	88/18670	3.80E-06	2.16E-05	8.59E-06	TERT/KDR/	7
GO:19027	regulatior	10/137	213/1867(	3.93E-06	2.23E-05	8.88E-06	CHEK1/HSI	10
GO:19018	positive r	5/137	33/18670	3.98E-06	2.25E-05	8.96E-06	KDR/VEGF/	5
GO:20010:	regulatior	10/137	214/1867(	4.10E-06	2.32E-05	9.23E-06	BCL2/CHEI	10
GO:19020	regulatior	6/137	58/18670	4.14E-06	2.33E-05	9.29E-06	BCL2L1/L(	6
GO:19045	regulatior	6/137	58/18670	4.14E-06	2.33E-05	9.29E-06	MAPK14/M/	6
GO:00511(	negative r	9/137	169/1867(	4.38E-06	2.47E-05	9.83E-06	MAPK3/ME1	9
GO:00436	regulatior	8/137	127/1867(	4.41E-06	2.48E-05	9.86E-06	CA9/CHEK1	8
GO:00310	positive r	7/137	90/18670	4.42E-06	2.48E-05	9.87E-06	MAPK3/DNI	7
GO:00468	bone remo(	7/137	90/18670	4.42E-06	2.48E-05	9.87E-06	SRC/PRKC/	7
GO:00018:	release of	6/137	59/18670	4.58E-06	2.56E-05	1.02E-05	MMP9/BCL2	6
GO:00336	membrane p	6/137	59/18670	4.58E-06	2.56E-05	1.02E-05	RET/TNF/I	6
GO:00433	positive r	6/137	59/18670	4.58E-06	2.56E-05	1.02E-05	PPARG/MMI	6
GO:00074:	salivary g	5/137	34/18670	4.64E-06	2.58E-05	1.03E-05	FGFR1/EGI	5
GO:00105'	regulatior	5/137	34/18670	4.64E-06	2.58E-05	1.03E-05	PTGS2/HII	5
GO:00347(	negative r	8/137	128/1867(	4.67E-06	2.60E-05	1.03E-05	MMP9/MTOI	8

GO:00303(	membrane c	4/137	16/18670	4.72E-06	2.62E-05	1.04E-05	CDK1/PRK(	4
GO:00510(	nuclear er	4/137	16/18670	4.72E-06	2.62E-05	1.04E-05	CDK1/PRK(	4
GO:00162	regulation	9/137	171/1867(	4.83E-06	2.67E-05	1.06E-05	MAPK3/KDI	9
GO:00550(	striated m	9/137	173/1867(	5.31E-06	2.94E-05	1.17E-05	PDGFRB/B(	9
GO:00713	cellular r	5/137	35/18670	5.38E-06	2.96E-05	1.18E-05	BCL2L1/C(	5
GO:19010	positive r	5/137	35/18670	5.38E-06	2.96E-05	1.18E-05	GSK3B/BCI	5
GO:19040	positive r	5/137	35/18670	5.38E-06	2.96E-05	1.18E-05	SRC/EGFR/	5
GO:19053	positive r	5/137	35/18670	5.38E-06	2.96E-05	1.18E-05	AR/MTOR/\	5
GO:00074(	neuroblast	6/137	61/18670	5.58E-06	3.06E-05	1.22E-05	FGFR1/VE(	6
GO:19059	negative r	6/137	61/18670	5.58E-06	3.06E-05	1.22E-05	PPARG/PTI	6
GO:00706(	regulation	10/137	222/1867(	5.68E-06	3.11E-05	1.24E-05	BCL2/LGAI	10
GO:00329	regulation	13/137	388/1867(	5.76E-06	3.15E-05	1.26E-05	TGFBR1/MI	13
GO:00455	regulation	8/137	132/1867(	5.87E-06	3.21E-05	1.28E-05	PPARG/MAI	8
GO:00550(	cardiac mu	8/137	132/1867(	5.87E-06	3.21E-05	1.28E-05	PDGFRB/M	8
GO:00609	kidney mor	7/137	94/18670	5.91E-06	3.22E-05	1.28E-05	STAT1/PD(	7
GO:00094(	response t	9/137	176/1867(	6.10E-06	3.32E-05	1.32E-05	MAPK3/GSI	9
GO:00022(	lymphocyte	6/137	62/18670	6.14E-06	3.34E-05	1.33E-05	BCL2/HIF	6
GO:00105	vascular e	5/137	36/18670	6.21E-06	3.37E-05	1.34E-05	PTGS2/HII	5
GO:00713	cellular r	8/137	133/1867(	6.21E-06	3.37E-05	1.34E-05	IGF1R/PII	8
GO:00440	regulation	7/137	95/18670	6.34E-06	3.44E-05	1.37E-05	CA7/ABCB	7
GO:00067(	vitamin me	8/137	134/1867(	6.56E-06	3.55E-05	1.41E-05	VDR/ABCC	8
GO:00070(	mitochondr	8/137	134/1867(	6.56E-06	3.55E-05	1.41E-05	GSK3B/BCI	8
GO:00603	response t	8/137	135/1867(	6.93E-06	3.74E-05	1.49E-05	MAPK3/MAI	8
GO:00713	cellular r	8/137	135/1867(	6.93E-06	3.74E-05	1.49E-05	IGF1R/PII	8
GO:19030	regulation	9/137	179/1867(	7.00E-06	3.77E-05	1.50E-05	MAP2K1/M	9
GO:00074	salivary g	5/137	37/18670	7.14E-06	3.82E-05	1.52E-05	FGFR1/EGI	5
GO:00140	Schwann ce	5/137	37/18670	7.14E-06	3.82E-05	1.52E-05	CDK1/AKT	5
GO:00302	hyaluronan	5/137	37/18670	7.14E-06	3.82E-05	1.52E-05	FGF2/AKT	5
GO:00344(	response t	5/137	37/18670	7.14E-06	3.82E-05	1.52E-05	SRC/PDGFI	5
GO:00430	T cell hom	5/137	37/18670	7.14E-06	3.82E-05	1.52E-05	BCL2/IL2/	5
GO:00159(	fatty acid	7/137	97/18670	7.28E-06	3.88E-05	1.54E-05	NOS2/PPAI	7
GO:00432	regulation	7/137	97/18670	7.28E-06	3.88E-05	1.54E-05	GSK3B/MT(	7
GO:19028	regulation	7/137	97/18670	7.28E-06	3.88E-05	1.54E-05	MET/MMP3/	7
GO:00713	cellular r	8/137	136/1867(	7.32E-06	3.90E-05	1.55E-05	IGF1R/PII	8
GO:00465	positive r	6/137	64/18670	7.39E-06	3.92E-05	1.56E-05	PDGFRB/HI	6
GO:19036	positive r	6/137	64/18670	7.39E-06	3.92E-05	1.56E-05	KDR/VEGF/	6
GO:00224(	negative r	9/137	181/1867(	7.66E-06	4.06E-05	1.61E-05	VEGFA/LG/	9
GO:00511	regulation	9/137	181/1867(	7.66E-06	4.06E-05	1.61E-05	MAPK14/DI	9
GO:00346(	cellular r	8/137	137/1867(	7.73E-06	4.08E-05	1.62E-05	MAPK3/GSI	8
GO:00464	regulation	8/137	137/1867(	7.73E-06	4.08E-05	1.62E-05	RET/KIT/S	8
GO:00300	contractil	7/137	98/18670	7.79E-06	4.11E-05	1.63E-05	TGFBR1/SI	7
GO:00431	stress fib	7/137	98/18670	7.79E-06	4.11E-05	1.63E-05	TGFBR1/SI	7
GO:00323	regulation	5/137	38/18670	8.17E-06	4.28E-05	1.70E-05	VDR/HIF1/	5
GO:00450	early endo	5/137	38/18670	8.17E-06	4.28E-05	1.70E-05	MAP2K1/M/	5
GO:00480(	insulin-li	5/137	38/18670	8.17E-06	4.28E-05	1.70E-05	AR/IGF1R/	5
GO:00604	response t	5/137	38/18670	8.17E-06	4.28E-05	1.70E-05	STAT3/PTI	5
GO:00703(	cellular r	7/137	99/18670	8.34E-06	4.36E-05	1.74E-05	SRC/MET/(	7

GO:00424	hormone me	10/137	232/1867(	8.37E-06	4.37E-05	1.74E-05	CYP17A1/I	10
GO:00353(	regulatior	8/137	139/1867(	8.60E-06	4.49E-05	1.79E-05	GSK3B/PD(	8
GO:00069	nucleocyto	12/137	343/1867(	8.69E-06	4.53E-05	1.80E-05	MAPK14/P7	12
GO:00107	negative r	12/137	344/1867(	8.94E-06	4.66E-05	1.85E-05	TERT/GSK3	12
GO:00485	developmer	10/137	234/1867(	9.02E-06	4.69E-05	1.87E-05	GSK3B/MT(	10
GO:19022	regulatior	9/137	185/1867(	9.14E-06	4.75E-05	1.89E-05	MAPK3/DNM	9
GO:00511(	nuclear tr	12/137	346/1867(	9.48E-06	4.92E-05	1.96E-05	MAPK14/P7	12
GO:00550(	muscle cel	9/137	186/1867(	9.54E-06	4.95E-05	1.97E-05	PDGFRB/B(	9
GO:00309	positive r	4/137	19/18670	9.87E-06	5.09E-05	2.03E-05	VEGFA/HII	4
GO:00601	negative r	4/137	19/18670	9.87E-06	5.09E-05	2.03E-05	ESR1/PPAI	4
GO:00604	trachea de	4/137	19/18670	9.87E-06	5.09E-05	2.03E-05	MAP2K1/MA	4
GO:00609(	negative r	4/137	19/18670	9.87E-06	5.09E-05	2.03E-05	ESR1/PPAI	4
GO:00720(	nephron de	8/137	142/1867(	1.01E-05	5.18E-05	2.06E-05	RET/STAT	8
GO:00439(	positive r	9/137	188/1867(	1.04E-05	5.34E-05	2.13E-05	NOS2/MAPI	9
GO:00517	regulatior	9/137	188/1867(	1.04E-05	5.34E-05	2.13E-05	PDGFRB/CI	9
GO:00465	regulatior	10/137	238/1867(	1.05E-05	5.37E-05	2.14E-05	MET/PDGFI	10
GO:00509	positive c	6/137	68/18670	1.05E-05	5.39E-05	2.15E-05	KDR/MET/V	6
GO:00712	cellular r	6/137	68/18670	1.05E-05	5.39E-05	2.15E-05	MMP2/DNM7	6
GO:00901	positive r	5/137	40/18670	1.06E-05	5.41E-05	2.15E-05	RET/PDGFI	5
GO:00066(	protein in	8/137	143/1867(	1.06E-05	5.41E-05	2.15E-05	MAPK14/MA	8
GO:19052(	positive r	7/137	103/1867(	1.08E-05	5.52E-05	2.20E-05	MAPK3/DNM	7
GO:00487(	skeletal s	10/137	239/1867(	1.08E-05	5.53E-05	2.20E-05	MAPK14/T(	10
GO:00302	T cell dif	10/137	240/1867(	1.12E-05	5.72E-05	2.28E-05	KIT/BCL2/	10
GO:00426	ovulation	6/137	69/18670	1.15E-05	5.82E-05	2.32E-05	ESR1/PGR/	6
GO:00713(	cellular r	6/137	69/18670	1.15E-05	5.82E-05	2.32E-05	PPARG/RE7	6
GO:00425	response t	9/137	191/1867(	1.18E-05	5.99E-05	2.38E-05	PPARG/MAI	9
GO:00015(	regulatior	12/137	354/1867(	1.19E-05	6.03E-05	2.40E-05	NOS2/GSK3	12
GO:00989	vesicle-me	5/137	41/18670	1.20E-05	6.06E-05	2.41E-05	MAP2K1/MA	5
GO:19048	regulatior	8/137	146/1867(	1.23E-05	6.22E-05	2.48E-05	RET/KIT/S	8
GO:20012	negative r	8/137	146/1867(	1.23E-05	6.22E-05	2.48E-05	SRC/DNMT	8
GO:00070(	telomere m	6/137	70/18670	1.25E-05	6.28E-05	2.50E-05	TERT/MAPI	6
GO:00706(	leukocyte	11/137	298/1867(	1.27E-05	6.39E-05	2.54E-05	KIT/BCL2/	11
GO:00342	regulatior	14/137	483/1867(	1.29E-05	6.49E-05	2.59E-05	MAPK3/MAI	14
GO:00425	positive r	6/137	71/18670	1.35E-05	6.80E-05	2.71E-05	KIT/STAT3	6
GO:00092(	cellular r	8/137	149/1867(	1.43E-05	7.17E-05	2.85E-05	MAPK3/MAI	8
GO:00103	regulatior	9/137	196/1867(	1.45E-05	7.27E-05	2.90E-05	HSP90AA1/	9
GO:00325	response t	7/137	108/1867(	1.48E-05	7.40E-05	2.94E-05	PPARG/RE7	7
GO:00064	regulatior	13/137	424/1867(	1.49E-05	7.42E-05	2.95E-05	MAPK3/MAI	13
GO:00519(	regulatior	4/137	21/18670	1.51E-05	7.51E-05	2.99E-05	SRC/KDR/I	4
GO:00550	response t	4/137	21/18670	1.51E-05	7.51E-05	2.99E-05	PPARG/PD(	4
GO:00456	positive r	5/137	43/18670	1.52E-05	7.56E-05	3.01E-05	PPARG/MT(	5
GO:00713(	cellular r	5/137	43/18670	1.52E-05	7.56E-05	3.01E-05	PTPN11/E(	5
GO:00459	negative r	10/137	249/1867(	1.55E-05	7.69E-05	3.06E-05	ESR2/PPAI	10
GO:00468	regulatior	9/137	198/1867(	1.57E-05	7.81E-05	3.11E-05	VDR/MTOR/	9
GO:00068(	superoxide	6/137	73/18670	1.59E-05	7.88E-05	3.14E-05	NOS2/EGFI	6
GO:00071	transformi	9/137	199/1867(	1.64E-05	8.09E-05	3.22E-05	TGFBR1/SI	9
GO:00170	protein in	9/137	199/1867(	1.64E-05	8.09E-05	3.22E-05	MAPK14/MA	9

GO:00310:	actomyosir	9/137	199/1867(	1.64E-05	8.09E-05	3.22E-05	TGFBR1/SI	9
GO:00140(	astrocyte	5/137	44/18670	1.71E-05	8.39E-05	3.34E-05	EGFR/CDK6	5
GO:00380{	vascular e	5/137	44/18670	1.71E-05	8.39E-05	3.34E-05	KDR/PDGFI	5
GO:19010:	regulatior	5/137	44/18670	1.71E-05	8.39E-05	3.34E-05	GSK3B/BCI	5
GO:00342!	positive r	8/137	153/1867(	1.73E-05	8.51E-05	3.39E-05	MAPK3/MAI	8
GO:00507:	positive r	8/137	153/1867(	1.73E-05	8.51E-05	3.39E-05	ABCC1/PLA	8
GO:00308;	thyroid gl	4/137	22/18670	1.83E-05	8.94E-05	3.56E-05	MAP2K1/MA	4
GO:00312{	positive r	4/137	22/18670	1.83E-05	8.94E-05	3.56E-05	NOS2/MAPI	4
GO:00443/	type B par	4/137	22/18670	1.83E-05	8.94E-05	3.56E-05	IGFBP3/BA	4
GO:00510(	positive r	4/137	22/18670	1.83E-05	8.94E-05	3.56E-05	TERT/HIF1	4
GO:19015:	positive r	4/137	22/18670	1.83E-05	8.94E-05	3.56E-05	VEGFA/HII	4
GO:00074:	peripheral	6/137	75/18670	1.86E-05	9.05E-05	3.60E-05	CDK1/ERBI	6
GO:19032(	regulatior	6/137	75/18670	1.86E-05	9.05E-05	3.60E-05	MET/MMP3/	6
GO:00325;	response t	5/137	45/18670	1.91E-05	9.28E-05	3.69E-05	SRC/TYMS/	5
GO:00905{	sensory or	10/137	256/1867(	1.97E-05	9.55E-05	3.80E-05	MAPK3/MAI	10
GO:00609(	regulatior	7/137	113/1867(	1.99E-05	9.64E-05	3.84E-05	ESR1/PPAI	7
GO:00347(	positive r	9/137	204/1867(	2.00E-05	9.67E-05	3.85E-05	MAPK14/TI	9
GO:00720{	nephron ep	6/137	76/18670	2.01E-05	9.71E-05	3.87E-05	STAT1/BCI	6
GO:00454/	endothelia	7/137	114/1867(	2.10E-05	1.02E-04	4.05E-05	KDR/MET/V	7
GO:00336:	regulatior	5/137	46/18670	2.13E-05	1.03E-04	4.08E-05	PTPN11/RI	5
GO:00351(	productior	5/137	46/18670	2.13E-05	1.03E-04	4.08E-05	ESR1/MAP2	5
GO:00716;	regulatior	5/137	46/18670	2.13E-05	1.03E-04	4.08E-05	MAPK14/MA	5
GO:00062;	RNA-depenc	6/137	77/18670	2.16E-05	1.04E-04	4.14E-05	TERT/MAPI	6
GO:00518{	mitochondr	4/137	23/18670	2.20E-05	1.06E-04	4.21E-05	SRC/KDR/I	4
GO:00711!	negative r	4/137	23/18670	2.20E-05	1.06E-04	4.21E-05	CCND1/CDI	4
GO:19034:	regulatior	4/137	23/18670	2.20E-05	1.06E-04	4.21E-05	RET/BCL2/	4
GO:00705!	response t	9/137	207/1867(	2.24E-05	1.07E-04	4.27E-05	MAPK3/SR(	9
GO:00506;	regulatior	9/137	208/1867(	2.33E-05	1.11E-04	4.42E-05	BCL2/LGAI	9
GO:00002;	polysacchæ	6/137	78/18670	2.33E-05	1.11E-04	4.42E-05	GSK3B/MT(	6
GO:00331/	regulatior	6/137	78/18670	2.33E-05	1.11E-04	4.42E-05	AR/ESR1/S	6
GO:00706(	negative r	6/137	78/18670	2.33E-05	1.11E-04	4.42E-05	IL2/ERBB2	6
GO:00149:	positive r	5/137	47/18670	2.37E-05	1.13E-04	4.48E-05	TERT/SRC/	5
GO:00216(	cerebellar	5/137	47/18670	2.37E-05	1.13E-04	4.48E-05	MAP2K1/PT	5
GO:00456/	regulatior	5/137	47/18670	2.37E-05	1.13E-04	4.48E-05	MAPK14/ST	5
GO:00329/	regulatior	9/137	209/1867(	2.42E-05	1.15E-04	4.56E-05	BCL2/LGAI	9
GO:00353(	regulatior	9/137	209/1867(	2.42E-05	1.15E-04	4.56E-05	SRC/GSK3I	9
GO:00105(	positive r	7/137	117/1867(	2.49E-05	1.18E-04	4.69E-05	MAPK3/KDI	7
GO:00436(	regulatior	7/137	117/1867(	2.49E-05	1.18E-04	4.69E-05	GSK3B/PD(	7
GO:00975:	myeloid le	9/137	210/1867(	2.51E-05	1.19E-04	4.72E-05	MAPK14/MA	9
GO:01400:	mitotic nu	10/137	264/1867(	2.56E-05	1.21E-04	4.81E-05	PDGFRB/CI	10
GO:00308!	prostate g	5/137	48/18670	2.63E-05	1.23E-04	4.91E-05	AR/ESR1/(	5
GO:00352;	exocrine s	5/137	48/18670	2.63E-05	1.23E-04	4.91E-05	FGFR1/EGI	5
GO:01010:	vascular e	5/137	48/18670	2.63E-05	1.23E-04	4.91E-05	PPARG/FGI	5
GO:19055(	regulatior	5/137	48/18670	2.63E-05	1.23E-04	4.91E-05	PPARG/FGI	5
GO:00091:	vitamin bi	4/137	24/18670	2.63E-05	1.23E-04	4.91E-05	VDR/TNF/I	4
GO:00603(	growth hor	4/137	24/18670	2.63E-05	1.23E-04	4.91E-05	STAT3/PTI	4
GO:00020{	lens devel	6/137	80/18670	2.69E-05	1.26E-04	5.02E-05	TGFBR1/EI	6

GO:00511[illegible]	import int	8/137	163/1867(	2.74E-05	1.28E-04	5.10E-05	MAPK14/MA	8
GO:00217(	hippocampu	6/137	81/18670	2.89E-05	1.35E-04	5.38E-05	GSK3B/CDI	6
GO:00512(	protein ir	5/137	49/18670	2.91E-05	1.36E-04	5.40E-05	BCL2/EGFI	5
GO:00160[illegible]	carbohydra	9/137	214/1867(	2.91E-05	1.36E-04	5.40E-05	GSK3B/MT(	9
GO:00457[illegible]	positive r	9/137	214/1867(	2.91E-05	1.36E-04	5.40E-05	GSK3B/HSI	9
GO:00017(	formation	7/137	121/1867(	3.09E-05	0.000143	5.70E-05	MMP2/MMP[illegible]	7
GO:00108[illegible]	positive r	7/137	121/1867(	3.09E-05	0.000143	5.70E-05	KDR/GSK3I	7
GO:00436[illegible]	regulation	7/137	121/1867(	3.09E-05	0.000143	5.70E-05	CA9/CHEK[illegible]	7
GO:00467[illegible]	viral entr	7/137	121/1867(	3.09E-05	0.000143	5.70E-05	CTSB/CDK[illegible]	7
GO:00346[illegible]	cellular r	6/137	82/18670	3.10E-05	0.000143	5.70E-05	CHEK1/PT(	6
GO:00975[illegible]	lamellipoc	6/137	82/18670	3.10E-05	0.000143	5.70E-05	SRC/KIT/M	6
GO:00190[illegible]	modulation	4/137	25/18670	3.11E-05	0.000143	5.70E-05	BCL2L1/CA	4
GO:00518[illegible]	positive r	4/137	25/18670	3.11E-05	0.000143	5.70E-05	KDR/VEGFA	4
GO:00603[illegible]	regulation	4/137	25/18670	3.11E-05	0.000143	5.70E-05	PPARG/STA	4
GO:00603[illegible]	regulation	4/137	25/18670	3.11E-05	0.000143	5.70E-05	PPARG/STA	4
GO:00605[illegible]	morphogene	4/137	25/18670	3.11E-05	0.000143	5.70E-05	AR/HIF1A/	4
GO:00713[illegible]	cellular r	4/137	25/18670	3.11E-05	0.000143	5.70E-05	STAT3/PTI	4
GO:00603[illegible]	bone devel	9/137	217/1867(	3.25E-05	0.000149	5.94E-05	PTPN11/SI	9
GO:00425(	regulation	6/137	83/18670	3.32E-05	0.000152	6.06E-05	KIT/STAT[illegible]	6
GO:00459[illegible]	positive r	6/137	83/18670	3.32E-05	0.000152	6.06E-05	SRC/HIF1A	6
GO:00508(	positive r	8/137	168/1867(	3.40E-05	0.000155	6.18E-05	CA7/GSK3I	8
GO:00513(	regulation	8/137	168/1867(	3.40E-05	0.000155	6.18E-05	BCL2L1/VI	8
GO:00020(	chondrocyt	7/137	123/1867(	3.44E-05	0.000157	6.25E-05	MAPK14/T(	7
GO:00106[illegible]	negative r	12/137	395/1867(	3.51E-05	0.00016	6.37E-05	SRC/MET/I	12
GO:00075(	embryo imp	5/137	51/18670	3.54E-05	0.000161	6.42E-05	MMP2/MMP[illegible]	5
GO:00311(	axon reger	5/137	51/18670	3.54E-05	0.000161	6.42E-05	MAP2K1/B(	5
GO:00514[illegible]	regulation	6/137	84/18670	3.56E-05	0.000162	6.43E-05	TGFBR1/MI	6
GO:00328(	receptor k	4/137	26/18670	3.66E-05	0.000165	6.59E-05	PPARG/HII	4
GO:00607[illegible]	prostate g	4/137	26/18670	3.66E-05	0.000165	6.59E-05	AR/ESR1/I	4
GO:19007[illegible]	regulation	4/137	26/18670	3.66E-05	0.000165	6.59E-05	BCL2/CASI	4
GO:19007[illegible]	positive r	4/137	26/18670	3.66E-05	0.000165	6.59E-05	BCL2/CASI	4
GO:19039[illegible]	regulation	4/137	26/18670	3.66E-05	0.000165	6.59E-05	ABCB1/MT(	4
GO:00085[illegible]	epidermis	13/137	464/1867(	3.80E-05	0.000172	6.83E-05	VDR/MAP2I	13
GO:00140[illegible]	mesenchyma	6/137	85/18670	3.80E-05	0.000172	6.83E-05	MAPK3/RE[illegible]	6
GO:00439(	regulation	9/137	222/1867(	3.88E-05	0.000175	6.96E-05	STAT1/BCI	9
GO:00458[illegible]	negative r	5/137	52/18670	3.90E-05	0.000175	6.98E-05	CHEK1/PLI	5
GO:00108[illegible]	lipid loca	12/137	400/1867(	3.97E-05	0.000178	7.10E-05	NOS2/PPAI	12
GO:00513[illegible]	negative r	13/137	466/1867(	3.97E-05	0.000178	7.10E-05	SRC/GSK3I	13
GO:00456(	regulation	7/137	126/1867(	4.01E-05	0.00018	7.17E-05	CDK6/TNF/	7
GO:00510[illegible]	regulation	11/137	338/1867(	4.05E-05	0.000181	7.22E-05	SRC/MET/I	11
GO:00019[illegible]	hair folli	6/137	86/18670	4.06E-05	0.000182	7.23E-05	BCL2/EGFI	6
GO:00072(	tyrosine p	6/137	86/18670	4.06E-05	0.000182	7.23E-05	KIT/STAT[illegible]	6
GO:00424[illegible]	hormone bi	6/137	86/18670	4.06E-05	0.000182	7.23E-05	CYP17A1/V	6
GO:00305[illegible]	leukocyte	9/137	224/1867(	4.16E-05	0.000186	7.39E-05	MAPK14/MA	9
GO:00016[illegible]	temperatur	8/137	173/1867(	4.19E-05	0.000187	7.43E-05	IGF1R/VE(	8
GO:00457[illegible]	positive r	7/137	127/1867(	4.22E-05	0.000188	7.48E-05	MAPK3/MAI	7
GO:00109[illegible]	positive r	4/137	27/18670	4.27E-05	0.000189	7.54E-05	CDK1/CCNI	4

GO:00359(	response t	4/137	27/18670	4.27E-05	0.000189	7.54E-05	PPARG/CYI	4
GO:00481	astrocyte	4/137	27/18670	4.27E-05	0.000189	7.54E-05	EGFR/TNF/	4
GO:19021	regulation	4/137	27/18670	4.27E-05	0.000189	7.54E-05	HIF1A/PAI	4
GO:00301(	regulation	10/137	281/1867(	4.35E-05	0.000192	7.66E-05	PPARG/SR(	10
GO:20012	positive r	8/137	174/1867(	4.36E-05	0.000193	7.69E-05	MAPK3/DNM	8
GO:00514	positive r	9/137	226/1867(	4.46E-05	0.000196	7.82E-05	TGFBR1/MI	9
GO:00016(	fever gene	3/137	10/18670	4.47E-05	0.000196	7.82E-05	PTGS2/TNI	3
GO:00109	positive r	3/137	10/18670	4.47E-05	0.000196	7.82E-05	AKT1/AKT2	3
GO:00140	regulation	3/137	10/18670	4.47E-05	0.000196	7.82E-05	RET/BCL2/	3
GO:00711(	response t	3/137	10/18670	4.47E-05	0.000196	7.82E-05	STAT1/STA	3
GO:20006	regulation	3/137	10/18670	4.47E-05	0.000196	7.82E-05	HRAS/NFKI	3
GO:00435	positive r	12/137	405/1867(	4.47E-05	0.000196	7.82E-05	GSK3B/HRA	12
GO:00097	post-embry	6/137	88/18670	4.63E-05	0.000202	8.06E-05	TGFBR1/B(	6
GO:00224(	molting cy	6/137	88/18670	4.63E-05	0.000202	8.06E-05	BCL2/EGFI	6
GO:00224(	hair cycle	6/137	88/18670	4.63E-05	0.000202	8.06E-05	BCL2/EGFI	6
GO:00987	skin epide	6/137	88/18670	4.63E-05	0.000202	8.06E-05	BCL2/EGFI	6
GO:00032(	cardiac ch	7/137	129/1867(	4.66E-05	0.000204	8.11E-05	TGFBR1/HI	7
GO:00714	cellular r	7/137	129/1867(	4.66E-05	0.000204	8.11E-05	CHEK1/PT(	7
GO:00027(	positive r	5/137	54/18670	4.68E-05	0.000204	8.12E-05	PRKCA/TNI	5
GO:00973	mitochondr	5/137	54/18670	4.68E-05	0.000204	8.12E-05	GSK3B/BCI	5
GO:00159	energy der	10/137	285/1867(	4.89E-05	0.000213	8.48E-05	NOS2/GSK3	10
GO:00604	regulation	8/137	177/1867(	4.92E-05	0.000214	8.52E-05	TGFBR1/SI	8
GO:00464	positive r	6/137	89/18670	4.93E-05	0.000214	8.52E-05	KIT/STAT3	6
GO:00550	cardiac mu	6/137	89/18670	4.93E-05	0.000214	8.52E-05	PDGFRB/MI	6
GO:00335	mammary gl	4/137	28/18670	4.95E-05	0.000214	8.52E-05	ESR1/MAPI	4
GO:00362	response t	4/137	28/18670	4.95E-05	0.000214	8.52E-05	PPARG/PD(	4
GO:00605	prostate g	4/137	28/18670	4.95E-05	0.000214	8.52E-05	AR/ESR1/I	4
GO:19907	response t	4/137	28/18670	4.95E-05	0.000214	8.52E-05	SRC/PTGS2	4
GO:00109	positive r	8/137	178/1867(	5.12E-05	0.000221	8.80E-05	PPARG/STA	8
GO:00468	positive r	7/137	131/1867(	5.15E-05	0.000222	8.83E-05	PTPN11/F(	7
GO:00140	neural cre	6/137	90/18670	5.25E-05	0.000226	9.00E-05	MAPK3/RET	6
GO:00716	mononuclea	6/137	90/18670	5.25E-05	0.000226	9.00E-05	MAPK14/MA	6
GO:00083(	regulation	8/137	179/1867(	5.33E-05	0.000229	9.12E-05	GSK3B/RET	8
GO:00031	endotheliu	7/137	132/1867(	5.40E-05	0.000231	9.21E-05	KDR/MET/V	7
GO:00425	myelinatio	7/137	132/1867(	5.40E-05	0.000231	9.21E-05	MTOR/ERBI	7
GO:00432	positive r	7/137	132/1867(	5.40E-05	0.000231	9.21E-05	PPARG/TNI	7
GO:00713	cellular r	8/137	180/1867(	5.55E-05	0.000237	9.45E-05	NOS2/PPAI	8
GO:00059	regulation	4/137	29/18670	5.72E-05	0.000244	9.71E-05	GSK3B/MT(	4
GO:00109(	regulation	4/137	29/18670	5.72E-05	0.000244	9.71E-05	GSK3B/MT(	4
GO:00707	response t	4/137	29/18670	5.72E-05	0.000244	9.71E-05	TGFBR1/SM	4
GO:00105(	regulation	8/137	181/1867(	5.77E-05	0.000246	9.79E-05	PPARG/OD(	8
GO:00226(	regulation	13/137	484/1867(	5.85E-05	0.000249	9.91E-05	MAP2K1/SI	13
GO:00072	ensheathme	7/137	134/1867(	5.94E-05	0.000252	0.0001	MTOR/ERBI	7
GO:00083(	axon enshe	7/137	134/1867(	5.94E-05	0.000252	0.0001	MTOR/ERBI	7
GO:19048	positive r	6/137	92/18670	5.95E-05	0.000252	0.0001	KIT/STAT3	6
GO:00433	neutrophil	13/137	485/1867(	5.97E-05	2.53E-04	1.01E-04	MAPK14/CT	13
GO:00064(	negative r	9/137	235/1867(	6.03E-05	2.56E-04	1.02E-04	IGF1R/PLI	9

GO:00427	hydrogen p	5/137	57/18670	6.09E-05	2.56E-04	1.02E-04	MMP3/STA	5
GO:00459	positive r	5/137	57/18670	6.09E-05	2.56E-04	1.02E-04	NOS2/HIF	5
GO:00480	platelet-c	5/137	57/18670	6.09E-05	2.56E-04	1.02E-04	PTPN11/SI	5
GO:19005	positive r	5/137	57/18670	6.09E-05	2.56E-04	1.02E-04	NOS2/HIF	5
GO:00316	positive r	3/137	11/18670	6.11E-05	2.56E-04	1.02E-04	PTGS2/TNI	3
GO:00468	positive r	3/137	11/18670	6.11E-05	2.56E-04	1.02E-04	HIF1A/TNI	3
GO:00519	negative r	3/137	11/18670	6.11E-05	2.56E-04	1.02E-04	PPARG/SR(	3
GO:00605	prostate g	3/137	11/18670	6.11E-05	2.56E-04	1.02E-04	ESR1/FGFI	3
GO:00607	prostate g	3/137	11/18670	6.11E-05	2.56E-04	1.02E-04	AR/ESR1/I	3
GO:00725	caveolin-m	3/137	11/18670	6.11E-05	2.56E-04	1.02E-04	MAPK3/SR(	3
GO:00749	mesoderm c	7/137	135/1867(	6.23E-05	2.61E-04	1.04E-04	FGFR1/VE(	7
GO:00320	positive r	6/137	93/18670	6.32E-05	2.64E-04	1.05E-04	TERT/GSK3	6
GO:00720	nephron tu	6/137	93/18670	6.32E-05	2.64E-04	1.05E-04	STAT1/BCI	6
GO:00022	neutrophil	13/137	488/1867(	6.36E-05	2.66E-04	1.06E-04	MAPK14/CT	13
GO:00508	regulation	8/137	184/1867(	6.47E-05	2.70E-04	1.08E-04	MMP14/BCI	8
GO:00162	macroautop	10/137	295/1867(	6.53E-05	2.72E-04	1.08E-04	MAPK3/SR(	10
GO:00507	negative r	10/137	295/1867(	6.53E-05	2.72E-04	1.08E-04	TERT/GSK3	10
GO:00018	protein in	4/137	30/18670	6.56E-05	2.72E-04	1.08E-04	BCL2/CASI	4
GO:00216	cerebellar	4/137	30/18670	6.56E-05	2.72E-04	1.08E-04	MAP2K1/PT	4
GO:00463	regulation	4/137	30/18670	6.56E-05	2.72E-04	1.08E-04	PPARG/MT(	4
GO:19027	positive r	4/137	30/18670	6.56E-05	2.72E-04	1.08E-04	CDK1/CCNI	4
GO:00305	intracellu	5/137	58/18670	6.63E-05	2.74E-04	1.09E-04	AR/ESR1/I	5
GO:00353	positive r	5/137	58/18670	6.63E-05	2.74E-04	1.09E-04	SRC/PDGFI	5
GO:00302	lipid modi	9/137	238/1867(	6.65E-05	2.75E-04	1.10E-04	PPARG/MAI	9
GO:00027	innate imm	10/137	298/1867(	7.10E-05	2.93E-04	1.17E-04	ESR1/CTSI	10
GO:00550	cardiac ce	6/137	95/18670	7.12E-05	2.93E-04	1.17E-04	PDGFRB/MT	6
GO:00613	renal tubu	6/137	95/18670	7.12E-05	2.93E-04	1.17E-04	STAT1/BCI	6
GO:01100	regulation	6/137	95/18670	7.12E-05	2.93E-04	1.17E-04	TGFBR1/MI	6
GO:20000	positive r	6/137	95/18670	7.12E-05	2.93E-04	1.17E-04	GSK3B/AUI	6
GO:00016	branching	5/137	59/18670	7.20E-05	2.95E-04	1.18E-04	BCL2/VEGI	5
GO:00018	endothelia	5/137	59/18670	7.20E-05	2.95E-04	1.18E-04	MET/VEGF/	5
GO:00311	neuron pro	5/137	59/18670	7.20E-05	2.95E-04	1.18E-04	MAP2K1/B(	5
GO:00034	axis elong	4/137	31/18670	7.49E-05	3.05E-04	1.22E-04	ESR1/FGFI	4
GO:00071	female mei	4/137	31/18670	7.49E-05	3.05E-04	1.22E-04	AURKA/TOI	4
GO:00101	response t	4/137	31/18670	7.49E-05	3.05E-04	1.22E-04	CCND1/CAS	4
GO:00315	G2 DNA dam	4/137	31/18670	7.49E-05	3.05E-04	1.22E-04	CHEK1/CDI	4
GO:00456	positive r	4/137	31/18670	7.49E-05	3.05E-04	1.22E-04	MAPK14/ST	4
GO:19033	positive r	4/137	31/18670	7.49E-05	3.05E-04	1.22E-04	KDR/VEGF/	4
GO:00192	regulation	6/137	96/18670	7.55E-05	3.08E-04	1.22E-04	PPARG/MT(	6
GO:00301	regulation	11/137	363/1867(	7.69E-05	3.13E-04	1.25E-04	ESR1/MAPI	11
GO:00421	neutrophil	13/137	498/1867(	7.80E-05	3.17E-04	1.26E-04	MAPK14/CT	13
GO:00517	negative r	5/137	60/18670	7.81E-05	3.17E-04	1.26E-04	CHEK1/PLI	5
GO:19021	positive r	5/137	60/18670	7.81E-05	3.17E-04	1.26E-04	GSK3B/BCI	5
GO:19033	positive r	7/137	140/1867(	7.84E-05	3.18E-04	1.27E-04	GSK3B/HSI	7
GO:00024	neutrophil	13/137	499/1867(	7.96E-05	3.22E-04	1.28E-04	MAPK14/CT	13
GO:00322	regulation	6/137	97/18670	8.00E-05	3.23E-04	1.29E-04	TGFBR1/MI	6
GO:00487	oligodendr	6/137	97/18670	8.00E-05	3.23E-04	1.29E-04	PPARG/MT(	6

GO:00068(	lipid trar	11/137	365/1867(	8.07E-05	3.25E-04	1.30E-04	NOS2/PPAI	11
GO:00025!	mast cell	3/137	12/18670	8.10E-05	3.25E-04	1.30E-04	KIT/VEGF/	3
GO:00426!	regulatior	3/137	12/18670	8.10E-05	3.25E-04	1.30E-04	AR/FGFR1/	3
GO:00603!	positive r	3/137	12/18670	8.10E-05	3.25E-04	1.30E-04	TGFBR1/P/	3
GO:00604	branching	3/137	12/18670	8.10E-05	3.25E-04	1.30E-04	ESR1/FGFI	3
GO:00716	commissura	3/137	12/18670	8.10E-05	3.25E-04	1.30E-04	VEGFA/SM(	3
GO:00975	mast cell	3/137	12/18670	8.10E-05	3.25E-04	1.30E-04	KIT/VEGF/	3
GO:00020	cell migra	6/137	98/18670	8.47E-05	3.40E-04	1.35E-04	KDR/VEGF/	6
GO:00459(	positive r	4/137	32/18670	8.51E-05	3.40E-04	1.35E-04	PTGS2/EGI	4
GO:00513	response t	4/137	32/18670	8.51E-05	3.40E-04	1.35E-04	SRC/PARP1	4
GO:00603	face morpl	4/137	32/18670	8.51E-05	0.00034	1.35E-04	PTPN11/MI	4
GO:00712!	cellular r	4/137	32/18670	8.51E-05	0.00034	1.35E-04	VDR/PPAR(	4
GO:00431	receptor m	8/137	192/1867(	8.72E-05	0.000348	1.39E-04	PPARG/VE(	8
GO:00488	inner ear	8/137	192/1867(	8.72E-05	0.000348	1.39E-04	PTPN11/F(	8
GO:00017(	cell fate	6/137	99/18670	8.96E-05	0.000357	1.42E-04	AR/FGFR1/	6
GO:00421	cellular k	9/137	248/1867(	9.12E-05	0.000363	1.45E-04	CYP17A1/I	9
GO:19026	mitochondı	5/137	62/18670	9.15E-05	0.000363	1.45E-04	GSK3B/BCI	5
GO:19043	regulatior	5/137	62/18670	9.15E-05	0.000363	1.45E-04	MAPK3/SR(	5
GO:00102	endomembra	12/137	438/1867(	9.44E-05	0.000375	1.49E-04	AR/MAP2K1	12
GO:00140	response t	4/137	33/18670	9.62E-05	0.00038	1.51E-04	MTOR/MDM2	4
GO:00363	response t	4/137	33/18670	9.62E-05	0.00038	1.51E-04	TGFBR1/SI	4
GO:00432	response t	4/137	33/18670	9.62E-05	0.00038	0.000151	MTOR/MDM2	4
GO:00512(	protein ir	4/137	33/18670	9.62E-05	0.00038	0.000151	BCL2/CASI	4
GO:19026!	regulatior	4/137	33/18670	9.62E-05	0.00038	0.000151	VEGFA/HII	4
GO:00109!	negative r	9/137	250/1867(	9.69E-05	0.000383	0.000152	SRC/MMP9/	9
GO:00076	learning	7/137	145/1867(	9.78E-05	0.000385	0.000153	KIT/MTOR/	7
GO:00719(	negative r	7/137	145/1867(	9.78E-05	0.000385	0.000153	PLK1/AKT1	7
GO:00512!	negative r	7/137	146/1867(	1.02E-04	0.000402	0.00016	LGALS3/II	7
GO:00074!	midgut dev	3/137	13/18670	1.05E-04	0.000409	0.000163	RET/EGFR/	3
GO:00148	regulatior	3/137	13/18670	1.05E-04	0.000409	0.000163	FGF2/STA1	3
GO:00316!	regulatior	3/137	13/18670	1.05E-04	0.000409	0.000163	PTGS2/TNI	3
GO:00357	interleuki	3/137	13/18670	1.05E-04	0.000409	0.000163	STAT3/JAI	3
GO:00474	regulatior	3/137	13/18670	1.05E-04	0.000409	0.000163	ABCB1/PT(	3
GO:00713!	cellular r	3/137	13/18670	0.000105	0.000409	0.000163	STAT3/JAI	3
GO:19015!	regulatior	3/137	13/18670	0.000105	0.000409	0.000163	VEGFA/TNI	3
GO:19031	regulatior	3/137	13/18670	0.000105	0.000409	0.000163	VEGFA/TNI	3
GO:19039(	negative r	3/137	13/18670	0.000105	0.000409	0.000163	MTOR/AKT1	3
GO:00023(	cytokine p	6/137	102/1867(	0.000106	0.000412	0.000164	MAPK14/M/	6
GO:00300	lamellipoc	5/137	64/18670	0.000107	0.000414	0.000165	KIT/MTOR/	5
GO:00357!	positive r	5/137	64/18670	0.000107	0.000414	0.000165	GSK3B/BCI	5
GO:00456(	positive r	5/137	64/18670	0.000107	0.000414	0.000165	PPARG/MAI	5
GO:00712	cellular r	7/137	147/1867(	0.000107	0.000414	0.000165	SRC/MET/(	7
GO:00070!	mitotic sp	4/137	34/18670	0.000108	0.000419	0.000167	PLK1/CCNI	4
GO:00315	spindle cl	4/137	34/18670	0.000108	0.000419	0.000167	PLK1/CCNI	4
GO:00321	activatior	4/137	34/18670	0.000108	0.000419	0.000167	SRC/MTOR/	4
GO:00711	spindle as	4/137	34/18670	0.000108	0.000419	0.000167	PLK1/CCNI	4
GO:00711	mitotic sp	4/137	34/18670	0.000108	0.000419	0.000167	PLK1/CCNI	4

GO:003514	tube forma	7/137	148/18670	0.000111	0.000429	0.000171	RET/VEGFA	7
GO:006104	regulation	7/137	148/18670	0.000111	0.000429	0.000171	MTOR/FGF2	7
GO:003209	negative r	6/137	103/18670	0.000112	0.00043	0.000171	MAPK3/MET	6
GO:003470	negative r	6/137	103/18670	0.000112	0.00043	0.000171	MMP9/MTOR	6
GO:003434	response t	8/137	199/18670	0.000112	0.00043	0.000171	NOS2/PPAR	8
GO:000738	pattern sp	12/137	446/18670	0.000112	0.00043	0.000171	AR/TGFBR1	12
GO:005190	negative r	10/137	315/18670	0.000112	0.000432	0.000172	TERT/GSK3	10
GO:006067	ureteric b	5/137	65/18670	0.000115	0.00044	0.000175	BCL2/VEGF	5
GO:200105	positive r	7/137	149/18670	0.000116	0.000445	0.000177	PPARG/TNF	7
GO:001923	sensory pe	6/137	104/18670	0.000118	0.000451	0.000179	MAPK3/MAP	6
GO:007188	leukocyte	6/137	104/18670	0.000118	0.000451	0.000179	PIK3CB/HI	6
GO:004828	organelle	12/137	449/18670	0.000119	0.000456	0.000182	KDR/PDGFR	12
GO:003367	negative r	9/137	257/18670	0.000119	0.000457	0.000182	IGF1R/PLK	9
GO:001633	morphogene	7/137	150/18670	0.000121	0.000461	0.000184	AR/RET/HI	7
GO:006025	regulation	4/137	35/18670	0.000122	0.000464	0.000185	TERT/MTOR	4
GO:007087	regulation	4/137	35/18670	0.000122	0.000464	0.000185	GSK3B/MTO	4
GO:009875	bone cell	4/137	35/18670	0.000122	0.000464	0.000185	PTPN11/SR	4
GO:004001	regulation	5/137	66/18670	0.000123	0.000468	0.000186	BCL2/PIK3	5
GO:007217	mesonephri	5/137	66/18670	0.000123	0.000468	0.000186	BCL2/VEGF	5
GO:190210	regulation	5/137	66/18670	0.000123	0.000468	0.000186	GSK3B/BCL	5
GO:190488	cranial sk	5/137	66/18670	0.000123	0.000468	0.000186	TGFBR1/MM	5
GO:190571	positive r	5/137	66/18670	0.000123	0.000468	0.000186	GSK3B/BCL	5
GO:005085	T cell rec	8/137	202/18670	0.000124	0.00047	0.000187	MAPK1/PIK	8
GO:000859	regulation	6/137	105/18670	0.000124	0.00047	0.000187	MMP14/KIT	6
GO:000221	activation	10/137	319/18670	0.000125	0.000471	0.000187	ESR1/CTSK	10
GO:001699	antibiotic	7/137	151/18670	0.000126	0.000476	0.000189	MMP3/STAT	7
GO:000330	cardiac mu	6/137	106/18670	0.000131	0.000494	0.000197	MTOR/PRKC	6
GO:000647	protein de	10/137	321/18670	0.000131	0.000494	0.000197	PTPN11/GS	10
GO:003090	hindbrain	7/137	152/18670	0.000131	0.000494	0.000197	MAP2K1/PT	7
GO:004210	positive r	5/137	67/18670	0.000133	0.000495	0.000197	STAT3/TNF	5
GO:001484	skeletal m	3/137	14/18670	0.000133	0.000495	0.000197	FGF2/STAT	3
GO:001485	regulation	3/137	14/18670	0.000133	0.000495	0.000197	FGF2/STAT	3
GO:003021	hyaluronan	3/137	14/18670	0.000133	0.000495	0.000197	NFKB1/IL1	3
GO:003609	phosphatid	3/137	14/18670	0.000133	0.000495	0.000197	PIK3CA/PI	3
GO:004565	positive r	3/137	14/18670	0.000133	0.000495	0.000197	PRKCA/CAS	3
GO:005171	positive r	3/137	14/18670	0.000133	0.000495	0.000197	NOS2/GAPD	3
GO:007067	response t	3/137	14/18670	0.000133	0.000495	0.000197	STAT3/JAK	3
GO:007139	cellular r	3/137	14/18670	0.000133	0.000495	0.000197	ESR1/MDM2	3
GO:007242	signal tra	3/137	14/18670	0.000133	0.000495	0.000197	CHEK1/PLK	3
GO:190505	positive r	3/137	14/18670	0.000133	0.000495	0.000197	MAPK14/MA	3
GO:003022	monocyte c	4/137	36/18670	0.000136	0.000505	0.000201	PPARG/VEG	4
GO:003094	regulation	4/137	36/18670	0.000136	0.000505	0.000201	VEGFA/HIF	4
GO:004584	negative r	4/137	36/18670	0.000136	0.000505	0.000201	PLK1/CCNB	4
GO:007139	cellular r	4/137	36/18670	0.000136	0.000505	0.000201	ESR1/EGFR	4
GO:009032	regulation	4/137	36/18670	0.000136	0.000505	0.000201	EGFR/TNFA	4
GO:190313	mononuclea	4/137	36/18670	0.000136	0.000505	0.000201	PPARG/VEG	4
GO:005101	actin fila	7/137	153/18670	0.000137	0.000507	0.000202	TGFBR1/SR	7

GO:00620	negative r	6/137	107/1867(	0.000138	0.00051	0.000203	STAT3/PAI	6
GO:00072	germ cell	9/137	262/1867(	0.000138	0.000511	0.000203	SRC/KIT/I	9
GO:00104	negative r	9/137	262/1867(	0.000138	0.000511	0.000203	SRC/MMP9/	9
GO:00313	negative r	9/137	264/1867(	0.000146	0.00054	0.000215	MAPK14/MI	9
GO:00215	cerebellum	4/137	37/18670	0.000152	0.000557	0.000222	MAP2K1/PT	4
GO:00332	response t	4/137	37/18670	0.000152	0.000557	0.000222	VDR/PTGS2	4
GO:00459	positive r	4/137	37/18670	0.000152	0.000557	0.000222	PPARG/PTC	4
GO:00603	head morph	4/137	37/18670	0.000152	0.000557	0.000222	PTPN11/MM	4
GO:00715	dopaminerg	4/137	37/18670	0.000152	0.000557	0.000222	GSK3B/VEC	4
GO:00900	positive r	4/137	37/18670	0.000152	0.000557	0.000222	KDR/VEGFA	4
GO:19021	negative r	4/137	37/18670	0.000152	0.000557	0.000222	PLK1/CCNI	4
GO:00159	long-chain	5/137	69/18670	0.000152	0.000558	0.000222	PPARG/PLA	5
GO:00148	striated m	6/137	109/1867(	0.000153	0.000558	0.000222	MTOR/PRKC	6
GO:00511	positive r	6/137	109/1867(	0.000153	0.000558	0.000222	MAPK14/MM	6
GO:00720	nephron ep	6/137	109/1867(	0.000153	0.000558	0.000222	STAT1/BCI	6
GO:00421	regulation	7/137	156/1867(	0.000154	0.000563	0.000224	LGALS3/II	7
GO:00164	protein pr	10/137	328/1867(	0.000156	0.00057	0.000227	SRC/MMP14	10
GO:00190	viral life	10/137	328/1867(	0.000156	0.00057	0.000227	CTSB/BCL2	10
GO:00026	negative r	12/137	463/1867(	0.000159	0.000578	0.00023	PPARG/LGA	12
GO:00017	neuron mig	7/137	157/1867(	0.00016	0.000583	0.000232	FGFR1/VEC	7
GO:00615	actin fila	7/137	157/1867(	0.00016	0.000583	0.000232	TGFBR1/SI	7
GO:19059	regulation	7/137	157/1867(	0.00016	0.000583	0.000232	PPARG/PTI	7
GO:00603	SMAD prote	5/137	70/18670	0.000163	0.000592	0.000236	TGFBR1/HN	5
GO:00720	regulation	5/137	70/18670	0.000163	0.000592	0.000236	TERT/VEGI	5
GO:00148	skeletal m	3/137	15/18670	0.000165	0.000595	0.000237	FGF2/STAT	3
GO:00307	sequesteri	3/137	15/18670	0.000165	0.000595	0.000237	PPARG/TNI	3
GO:00323	positive r	3/137	15/18670	0.000165	0.000595	0.000237	HIF1A/TNI	3
GO:00356	entry of h	3/137	15/18670	0.000165	0.000595	0.000237	SRC/MET/(	3
GO:00454	positive r	3/137	15/18670	0.000165	0.000595	0.000237	STAT3/TNI	3
GO:00458	positive r	3/137	15/18670	0.000165	0.000595	0.000237	AKT1/AKT2	3
GO:00148	muscle hyp	6/137	111/1867(	0.000168	0.000607	0.000242	MTOR/PRKC	6
GO:00220	metencepha	6/137	111/1867(	0.000168	0.000607	0.000242	MAP2K1/PT	6
GO:00108	positive r	4/137	38/18670	0.000169	0.000607	0.000242	MAPK14/MM	4
GO:00463	negative r	4/137	38/18670	1.69E-04	6.07E-04	2.42E-04	AKT1/GSTI	4
GO:00468	regulation	4/137	38/18670	1.69E-04	6.07E-04	2.42E-04	PTPN11/GS	4
GO:00516	protein ma	11/137	397/1867(	1.69E-04	6.07E-04	2.42E-04	SRC/MMP14	11
GO:00901	establishm	10/137	332/1867(	1.72E-04	6.18E-04	2.46E-04	BCL2/HSP9	10
GO:00987	maintenanc	7/137	159/1867(	1.73E-04	6.22E-04	2.48E-04	KIT/FGF2/	7
GO:00070	actin fila	11/137	400/1867(	1.80E-04	6.46E-04	2.57E-04	TGFBR1/SI	11
GO:00466	lymphocyte	9/137	272/1867(	1.83E-04	6.55E-04	2.61E-04	BCL2/LGAI	9
GO:00022	stimulator	6/137	113/1867(	1.86E-04	6.63E-04	2.64E-04	SRC/HRAS/	6
GO:00063	DNA alkyla	5/137	72/18670	1.86E-04	6.63E-04	2.64E-04	DNMT1/PII	5
GO:00063	DNA methyl	5/137	72/18670	1.86E-04	6.63E-04	2.64E-04	DNMT1/PII	5
GO:00350	somatic st	5/137	72/18670	1.86E-04	6.63E-04	2.64E-04	KIT/FGF2/	5
GO:00511	positive r	5/137	72/18670	1.86E-04	6.63E-04	2.64E-04	MAPK14/MM	5
GO:00331	regulation	4/137	39/18670	1.87E-04	6.63E-04	2.64E-04	AR/ESR1/S	4
GO:00335	response t	4/137	39/18670	1.87E-04	6.63E-04	2.64E-04	AR/CA9/CI	4

GO:00609(	negative r	4/137	39/18670	1.87E-04	6.63E-04	2.64E-04	ESR1/PPAI	4
GO:19020	negative r	4/137	39/18670	1.87E-04	6.63E-04	2.64E-04	BCL2L1/C/	4
GO:20006	positive r	4/137	39/18670	1.87E-04	6.63E-04	2.64E-04	TERT/VEGI	4
GO:20008	negative r	4/137	39/18670	1.87E-04	6.63E-04	2.64E-04	PLK1/CCNI	4
GO:00329	mononuclea	9/137	274/1867(	1.93E-04	6.85E-04	2.73E-04	BCL2/LGAI	9
GO:00007	telomere m	7/137	162/1867(	1.95E-04	6.88E-04	2.74E-04	TERT/MAPI	7
GO:00157	monocarbox	7/137	162/1867(	1.95E-04	6.88E-04	2.74E-04	NOS2/PPAI	7
GO:00059	polysaccha	6/137	114/1867(	1.95E-04	6.88E-04	2.74E-04	GSK3B/MT(	6
GO:00420	regulation	6/137	114/1867(	1.95E-04	6.88E-04	2.74E-04	STAT3/TNI	6
GO:00032(	cardiac ve	5/137	73/18670	1.99E-04	7.00E-04	2.79E-04	TGFBR1/HI	5
GO:00329	negative r	5/137	73/18670	1.99E-04	7.00E-04	2.79E-04	IL2/ERBB2	5
GO:00506	negative r	5/137	73/18670	1.99E-04	7.00E-04	2.79E-04	IL2/ERBB2	5
GO:19019	regulation	5/137	73/18670	1.99E-04	7.00E-04	2.79E-04	MAPK3/GSI	5
GO:00020	epithelial	3/137	16/18670	2.02E-04	7.05E-04	2.81E-04	PGR/HIF1/	3
GO:00027	regulation	3/137	16/18670	2.02E-04	7.05E-04	2.81E-04	MAPK14/M/	3
GO:00062(	pyrimidine	3/137	16/18670	2.02E-04	7.05E-04	2.81E-04	MAPK1/MT(	3
GO:00432	myelin mai	3/137	16/18670	2.02E-04	7.05E-04	2.81E-04	AKT1/AKT2	3
GO:00480	hepatocyte	3/137	16/18670	2.02E-04	7.05E-04	2.81E-04	MET/RAC1/	3
GO:00609(	negative r	3/137	16/18670	2.02E-04	7.05E-04	2.81E-04	ESR1/PPAI	3
GO:00713	cellular r	3/137	16/18670	2.02E-04	7.05E-04	2.81E-04	PPARG/AKT	3
GO:00717	cellular r	3/137	16/18670	2.02E-04	7.05E-04	2.81E-04	MMP3/CDK2	3
GO:00903	regulation	3/137	16/18670	2.02E-04	7.05E-04	2.81E-04	MAPK14/MT	3
GO:00714	cellular r	4/137	40/18670	2.07E-04	7.20E-04	2.87E-04	PTGS2/CAS	4
GO:00722	metanephri	4/137	40/18670	2.07E-04	7.20E-04	2.87E-04	RET/STAT	4
GO:19058	negative r	4/137	40/18670	2.07E-04	7.20E-04	2.87E-04	PLK1/CCNI	4
GO:00328	regulation	8/137	218/1867(	2.09E-04	7.28E-04	2.90E-04	MET/GSK3I	8
GO:00002	nuclear di	11/137	407/1867(	2.09E-04	7.28E-04	2.90E-04	PDGFRB/CI	11
GO:00316	cellular r	5/137	74/18670	2.12E-04	7.36E-04	2.93E-04	VDR/PPAR(	5
GO:00511	smooth mus	5/137	74/18670	2.12E-04	7.36E-04	2.93E-04	DNMT1/KIT	5
GO:00720	nephron tu	5/137	74/18670	2.12E-04	7.36E-04	2.93E-04	BCL2/VEGI	5
GO:19030	positive r	5/137	74/18670	2.12E-04	7.36E-04	2.93E-04	MTOR/HRAS	5
GO:00022	innate imm	6/137	116/1867(	2.14E-04	7.42E-04	2.95E-04	SRC/HRAS/	6
GO:00219	cerebral c	6/137	116/1867(	2.14E-04	7.42E-04	2.95E-04	GSK3B/HII	6
GO:00511	regulation	6/137	117/1867(	2.25E-04	0.000776	3.09E-04	MAPK14/MM	6
GO:00022	hematopoie	7/137	166/1867(	2.26E-04	0.000781	3.11E-04	PDGFRB/KI	7
GO:00215	hindbrain	4/137	41/18670	2.28E-04	0.000785	3.13E-04	MAP2K1/PT	4
GO:00610	establishm	4/137	41/18670	2.28E-04	0.000785	3.13E-04	VEGFA/TNI	4
GO:00157	organic ar	12/137	482/1867(	2.30E-04	0.000792	3.15E-04	NOS2/PPAI	12
GO:00019	negative r	5/137	76/18670	2.41E-04	0.000828	3.30E-04	PPARG/TGI	5
GO:00604	cardiac se	5/137	76/18670	2.41E-04	0.000828	3.30E-04	TGFBR1/MI	5
GO:19901	neuron pro	7/137	168/1867(	2.43E-04	0.000835	3.32E-04	GSK3B/VE(	7
GO:00316	heat gener	3/137	17/18670	2.44E-04	0.000835	3.32E-04	PTGS2/TNI	3
GO:00358	megakaryoc	3/137	17/18670	2.44E-04	0.000835	3.32E-04	PTPN11/KI	3
GO:00517(	regulation	3/137	17/18670	2.44E-04	0.000835	3.32E-04	NOS2/GAPI	3
GO:19049	positive r	3/137	17/18670	2.44E-04	0.000835	3.32E-04	TNF/ICAM	3
GO:20012(	regulation	3/137	17/18670	2.44E-04	0.000835	0.000332	MMP9/CASI	3
GO:00106	positive r	4/137	42/18670	2.50E-04	0.000855	0.00034	MTOR/PRK(	4

GO:00330	negative r	4/137	42/18670	2.50E-04	0.000855	0.00034	PLK1/CCNI	4
GO:00456	regulation	7/137	169/1867(	2.52E-04	0.000862	0.000343	MMP14/IL2	7
GO:00324	positive r	5/137	77/18670	2.56E-04	0.000871	0.000347	PTPN11/ST	5
GO:00614	regulation	5/137	77/18670	2.56E-04	0.000871	0.000347	CA9/VEGFA	5
GO:19012	positive r	5/137	77/18670	2.56E-04	0.000871	0.000347	EGFR/TNF/	5
GO:19030	positive r	6/137	120/1867(	2.58E-04	0.000877	0.000349	GSK3B/AUI	6
GO:00608	regulation	9/137	286/1867(	2.65E-04	0.000903	0.00036	MAPK14/SI	9
GO:00435	skin devel	11/137	419/1867(	2.69E-04	0.000913	0.000364	VDR/MAP2I	11
GO:00030	regionaliz	10/137	351/1867(	0.000269	0.000915	0.000364	AR/TGFBRI	10
GO:00032	cardiac ch	7/137	171/1867(	0.000271	0.00092	0.000366	TGFBR1/HI	7
GO:00613	renal tubu	5/137	78/18670	0.000272	0.00092	0.000366	BCL2/VEGI	5
GO:00723	response t	5/137	78/18670	0.000272	0.00092	0.000366	MTOR/CCNA	5
GO:00903	cellular s	5/137	78/18670	0.000272	0.00092	0.000366	MAPK14/MA	5
GO:00147	positive r	4/137	43/18670	0.000274	0.000925	0.000368	MTOR/PRKC	4
GO:00312	regulation	4/137	43/18670	0.000274	0.000925	0.000368	NOS2/MAPI	4
GO:00324	regulation	4/137	43/18670	0.000274	0.000925	0.000368	SRC/MMP3/	4
GO:00341	cellular r	4/137	43/18670	0.000274	0.000925	0.000368	MAPK3/MAI	4
GO:19046	cellular r	4/137	43/18670	0.000274	0.000925	0.000368	IGF1R/GSI	4
GO:00018	tissue hom	8/137	227/1867(	0.000275	0.000927	0.000369	PTPN11/SI	8
GO:00435	muscle ada	6/137	122/1867(	0.000282	0.000948	0.000377	MTOR/PRKC	6
GO:00031	outflow tr	5/137	79/18670	0.000288	0.00097	0.000386	VEGFA/HII	5
GO:00030	vascular p	7/137	173/1867(	0.000291	0.000975	0.000388	SRC/VEGFA	7
GO:00312	membrane p	3/137	18/18670	0.000291	0.000975	0.000388	NFKB1/REI	3
GO:00319	regulation	3/137	18/18670	0.000291	0.000975	0.000388	MTOR/AKTI	3
GO:00609	coronary v	3/137	18/18670	0.000291	0.000975	0.000388	TGFBR1/PI	3
GO:00901	positive r	3/137	18/18670	0.000291	0.000975	0.000388	VEGFA/SMC	3
GO:00420	cytokine b	6/137	123/1867(	0.000294	0.000985	0.000392	STAT3/TNI	6
GO:00330	negative r	4/137	44/18670	0.0003	0.001	0.000398	PLK1/CCNI	4
GO:00341	positive r	4/137	44/18670	0.0003	0.001	0.000398	VDR/PRKCA	4
GO:00353	positive r	4/137	44/18670	0.0003	0.001	0.000398	PDGFRB/MT	4
GO:00617	leukocyte	4/137	44/18670	0.0003	0.001	0.000398	TNF/ICAMI	4
GO:00903	regulation	4/137	44/18670	0.0003	0.001	0.000398	VEGFA/MAI	4
GO:00464	phosphatic	7/137	174/1867(	0.000301	0.001003	0.0004	PLA2G2A/I	7
GO:00421	cytokine m	6/137	124/1867(	0.000307	0.001023	0.000407	STAT3/TNI	6
GO:00026	negative r	7/137	175/1867(	0.000312	0.001035	0.000412	LGALS3/II	7
GO:00109	regulation	7/137	175/1867(	0.000312	0.001035	0.000412	GSK3B/PDC	7
GO:00322	telomere c	7/137	175/1867(	0.000312	0.001035	0.000412	TERT/MAPI	7
GO:01200	regulation	7/137	175/1867(	0.000312	0.001035	0.000412	TGFBR1/KI	7
GO:00319	TOR signal	6/137	125/1867(	0.000321	0.001064	0.000424	MTOR/PIK3	6
GO:00140	neural cre	5/137	81/18670	0.000324	0.001071	0.000426	MAPK3/RET	5
GO:00311	anaphase-p	5/137	81/18670	0.000324	0.001071	0.000426	CDK1/AURI	5
GO:00322	regulation	5/137	81/18670	0.000324	0.001071	0.000426	MAPK3/SRC	5
GO:00324	positive r	5/137	81/18670	0.000324	0.001071	0.000426	GSK3B/AUI	5
GO:00487	embryonic	4/137	45/18670	0.000327	0.001079	0.00043	TGFBR1/MI	4
GO:00519	negative r	4/137	45/18670	0.000327	0.001079	0.00043	PLK1/CCNI	4
GO:19027	regulation	4/137	45/18670	0.000327	0.001079	0.00043	SRC/MTOR/	4
GO:00326	regulation	5/137	82/18670	0.000343	0.001124	0.000448	HIF1A/EPI	5

GO:00345(	protein l(	5/137	82/18670	0.000343	0.001124	0.000448	ESR1/TER[illegible]	5
GO:00236(	alpha-beta	3/137	19/18670	0.000344	0.001124	0.000448	BCL2/MTO[illegible]	3
GO:00329(	positive r	3/137	19/18670	0.000344	0.001124	0.000448	EGFR/TGF[illegible]	3
GO:00331(	response t	3/137	19/18670	0.000344	0.001124	0.000448	PPARG/CY[illegible]	3
GO:00363(	lymph vess	3/137	19/18670	0.000344	0.001124	0.000448	VEGFA/EP[illegible]	3
GO:00602(	positive r	3/137	19/18670	0.000344	0.001124	0.000448	MTOR/TNF/	3
GO:00717(	response t	3/137	19/18670	0.000344	0.001124	0.000448	MMP3/CDK[illegible]	3
GO:19004(	positive r	3/137	19/18670	0.000344	0.001124	0.000448	MMP3/TNF/	3
GO:19021(	cellular r	3/137	19/18670	0.000344	0.001124	0.000448	MMP3/CDK[illegible]	3
GO:00091(	nucleoside	10/137	362/1867(	0.000344	0.001124	0.000448	HIF1A/ST[illegible]	10
GO:00508(	protein st	7/137	178/1867(	0.000346	0.001129	0.00045	HSP90AA1/	7
GO:00323(	regulation	6/137	127/1867(	0.00035	0.001141	0.000454	PPARG/PT[illegible]	6
GO:00059(	glycogen b	4/137	46/18670	0.000357	0.001156	0.00046	GSK3B/MT[illegible]	4
GO:00092(	glucan bio	4/137	46/18670	0.000357	0.001156	0.00046	GSK3B/MT[illegible]	4
GO:00226(	ovulation	4/137	46/18670	0.000357	0.001156	0.00046	ESR1/PGR/	4
GO:00316(	protein de	4/137	46/18670	0.000357	0.001156	0.00046	SRC/PLK1/	4
GO:00323(	negative r	4/137	46/18670	0.000357	0.001156	0.00046	PTPN11/A[illegible]	4
GO:00485(	oocyte dev	4/137	46/18670	0.000357	0.001156	0.00046	BCL2/AUR[illegible]	4
GO:19040(	negative r	4/137	46/18670	0.000357	0.001156	0.00046	TERT/KDR/	4
GO:19909(	response t	4/137	46/18670	0.000357	0.001156	0.00046	MAPK3/MA[illegible]	4
GO:00301(	positive r	7/137	179/1867(	0.000358	0.001158	0.000461	TERT/SRC/	7
GO:00713(	cellular r	7/137	179/1867(	0.000358	0.001158	0.000461	MAPK3/HI[illegible]	7
GO:00423(	regulation	11/137	434/1867(	0.000362	0.001172	0.000467	SRC/KDR/[illegible]	11
GO:00431(	regulation	8/137	237/1867(	0.000367	0.001188	0.000473	ESR1/STA[illegible]	8
GO:19030(	negative r	6/137	129/1867(	0.00038	0.001228	0.000489	LGALS3/I[illegible]	6
GO:00027(	regulation	5/137	84/18670	0.000384	0.001237	0.000493	MAPK14/M[illegible]	5
GO:00468(	positive r	5/137	84/18670	0.000384	0.001237	0.000493	MTOR/PTG[illegible]	5
GO:00069(	acute-phas	4/137	47/18670	0.000387	0.001248	0.000497	PTGS2/ST[illegible]	4
GO:19035(	positive r	4/137	47/18670	0.000387	0.001248	0.000497	HIF1A/ST[illegible]	4
GO:00032(	cardiac ve	6/137	130/1867(	0.000396	0.001275	0.000508	TGFBR1/H[illegible]	6
GO:00023(	cytokine s	3/137	20/18670	0.000402	0.001289	0.000513	MAPK14/M[illegible]	3
GO:00302(	platelet f	3/137	20/18670	0.000402	0.001289	0.000513	PTPN11/C[illegible]	3
GO:00433(	CD4-positi	3/137	20/18670	0.000402	0.001289	0.000513	BCL2/MTO[illegible]	3
GO:00606(	branch el(	3/137	20/18670	0.000402	0.001289	0.000513	ESR1/FGF[illegible]	3
GO:19055(	positive r	3/137	20/18670	0.000402	0.001289	0.000513	MAPK14/M[illegible]	3
GO:00488(	stem cell	5/137	85/18670	0.000405	0.001298	0.000517	MAPK3/RE[illegible]	5
GO:00325(	regulation	10/137	370/1867(	0.000408	0.001306	0.00052	GSK3B/RE[illegible]	10
GO:00380(	NIK/NF-kap	7/137	183/1867(	0.000409	0.001306	0.00052	EGFR/ALK/	7
GO:00507(	regulation	7/137	183/1867(	0.000409	0.001306	0.00052	MAP2K1/G[illegible]	7
GO:00900(	regulation	8/137	241/1867(	0.000411	0.001311	0.000522	TGFBR1/P[illegible]	8
GO:00017(	microglial	4/137	48/18670	0.00042	0.001333	0.000531	TNF/JUN/[illegible]	4
GO:00022(	leukocyte	4/137	48/18670	0.00042	0.001333	0.000531	TNF/JUN/[illegible]	4
GO:00075(	lactation	4/137	48/18670	0.00042	0.001333	0.000531	VDR/VEGF[illegible]	4
GO:00080(	neuron rec	4/137	48/18670	0.00042	0.001333	0.000531	CASP3/EP[illegible]	4
GO:00085(	visual lea	4/137	48/18670	0.00042	0.001333	0.000531	KIT/MTOR/	4
GO:00459(	negative r	4/137	48/18670	0.00042	0.001333	0.000531	GSK3B/ST[illegible]	4
GO:00468(	regulation	4/137	48/18670	0.00042	0.001333	0.000531	SRC/PRKC[illegible]	4

GO:0051159	regulation	4/137	48/18670	0.00042	0.001333	0.000531	DNMT1/KIT	4
GO:0090199	regulation	4/137	48/18670	0.00042	0.001333	0.000531	MMP9/BCL2	4
GO:0042098	T cell pro	7/137	184/18670	0.000422	0.001337	0.000532	LGALS3/IL	7
GO:0043409	negative r	7/137	184/18670	4.22E-04	1.34E-03	5.32E-04	IGF1R/AKT	7
GO:0046034	ATP metabo	9/137	305/18670	4.25E-04	1.34E-03	5.35E-04	HIF1A/STA	9
GO:0006919	activation	5/137	86/18670	4.28E-04	1.35E-03	5.38E-04	PPARG/TNF	5
GO:0034614	cellular r	5/137	86/18670	4.28E-04	1.35E-03	5.38E-04	GSK3B/MTO	5
GO:0072175	epithelial	6/137	132/18670	4.30E-04	1.36E-03	5.40E-04	RET/HIF1A	6
GO:0044282	small mole	11/137	445/18670	4.47E-04	1.41E-03	5.61E-04	NOS2/MTOR	11
GO:0070507	regulation	7/137	186/18670	4.50E-04	1.42E-03	5.65E-04	MET/GSK3B	7
GO:0048477	oogenesis	5/137	87/18670	4.51E-04	1.42E-03	5.65E-04	SRC/BCL2/	5
GO:0051781	positive r	5/137	87/18670	4.51E-04	1.42E-03	5.65E-04	VEGFA/FGF	5
GO:0009123	nucleoside	10/137	375/18670	4.54E-04	1.43E-03	5.68E-04	HIF1A/STA	10
GO:0032350	regulation	4/137	49/18670	4.55E-04	1.43E-03	5.68E-04	SRC/KDR/F	4
GO:0010715	regulation	4/137	49/18670	4.55E-04	1.43E-03	5.68E-04	PPARG/PDG	4
GO:0043124	negative r	4/137	49/18670	4.55E-04	1.43E-03	5.68E-04	ESR1/STAT	4
GO:0071715	icosanoid	4/137	49/18670	4.55E-04	1.43E-03	5.68E-04	NOS2/ABCC	4
GO:1901571	fatty acid	4/137	49/18670	4.55E-04	1.43E-03	5.68E-04	NOS2/ABCC	4
GO:0032270	positive r	6/137	134/18670	4.66E-04	1.45E-03	5.78E-04	MET/MTOR/	6
GO:0046683	response t	6/137	134/18670	4.66E-04	0.001451	5.78E-04	STAT1/PTG	6
GO:0010310	regulation	3/137	21/18670	4.67E-04	0.001451	5.78E-04	MMP3/STAT	3
GO:0032511	positive r	3/137	21/18670	4.67E-04	0.001451	5.78E-04	PDGFRB/MT	3
GO:0036120	cellular r	3/137	21/18670	4.67E-04	0.001451	5.78E-04	SRC/PDGFR	3
GO:0036344	platelet m	3/137	21/18670	4.67E-04	0.001451	5.78E-04	PTPN11/CA	3
GO:0046826	positive r	3/137	21/18670	4.67E-04	0.001451	5.78E-04	GSK3B/MDM	3
GO:0061213	positive r	3/137	21/18670	4.67E-04	0.001451	5.78E-04	VEGFA/SMO	3
GO:1902895	positive r	3/137	21/18670	4.67E-04	0.001451	5.78E-04	MMP3/TNF/	3
GO:1903393	negative r	3/137	21/18670	4.67E-04	0.001451	5.78E-04	SRC/MMP14	3
GO:0097194	execution	5/137	88/18670	4.76E-04	0.001477	5.88E-04	BCL2L1/TG	5
GO:2000177	regulation	5/137	88/18670	4.76E-04	0.001477	5.88E-04	VEGFA/HIF	5
GO:0009141	nucleoside	7/137	188/18670	4.80E-04	0.00149	5.93E-04	HIF1A/STA	7
GO:0010171	body morph	4/137	50/18670	4.92E-04	0.001522	6.06E-04	PTPN11/MM	4
GO:0014002	glial cell	4/137	50/18670	4.92E-04	0.001522	6.06E-04	TERT/MTOR	4
GO:0032873	negative r	4/137	50/18670	4.92E-04	0.001522	6.06E-04	AKT1/GSTP	4
GO:0070303	negative r	4/137	50/18670	4.92E-04	0.001522	6.06E-04	AKT1/GSTP	4
GO:0032602	chemokine	5/137	89/18670	5.01E-04	0.001547	6.16E-04	HIF1A/EPH	5
GO:0097306	cellular r	5/137	89/18670	5.01E-04	0.001547	6.16E-04	PPARG/AKT	5
GO:0045088	positive r	10/137	381/18670	5.13E-04	0.001585	6.31E-04	ESR1/CTSB	10
GO:0072655	establishm	6/137	137/18670	5.24E-04	0.001614	6.43E-04	BCL2/HSP9	6
GO:1903672	regulation	6/137	137/18670	5.24E-04	0.001614	6.43E-04	KDR/VEGFA	6
GO:0001656	metanephro	5/137	90/18670	5.27E-04	0.001623	6.46E-04	RET/STAT1	5
GO:0051304	chromosome	5/137	90/18670	5.27E-04	0.001623	0.000646	TOP2A/PLK	5
GO:0009994	oocyte dif	4/137	51/18670	5.31E-04	0.001628	0.000648	BCL2/AURK	4
GO:0030071	regulation	4/137	51/18670	5.31E-04	0.001628	0.000648	PLK1/CCNB	4
GO:0045606	negative r	4/137	51/18670	5.31E-04	0.001628	0.000648	CDK6/TNF/	4
GO:0051495	positive r	4/137	51/18670	5.31E-04	0.001628	0.000648	TGFBR1/MT	4
GO:0097366	response t	4/137	51/18670	5.31E-04	0.001628	0.000648	MMP3/CDK2	4

GO:00066:	fatty acid	10/137	383/1867(	5.35E-04	0.001636	0.000651	PPARG/MAI	10
GO:00020!	positive r	3/137	22/18670	5.37E-04	0.001636	0.000651	VEGFA/HII	3
GO:00108(	regulatior	3/137	22/18670	5.37E-04	0.001636	0.000651	PPARG/HII	3
GO:00329:	regulatior	3/137	22/18670	5.37E-04	0.001636	0.000651	EGFR/TGFI	3
GO:00361:	response t	3/137	22/18670	5.37E-04	0.001636	0.000651	SRC/PDGFI	3
GO:00443:	response t	3/137	22/18670	5.37E-04	0.001636	0.000651	STAT3/CCI	3
GO:00456	regulatior	3/137	22/18670	5.37E-04	0.001636	0.000651	PRKCA/CAS	3
GO:00603!	regulatior	3/137	22/18670	5.37E-04	0.001636	0.000651	TGFBR1/PA	3
GO:00713	cellular r	3/137	22/18670	5.37E-04	0.001636	0.000651	PPARG/AKT	3
GO:00901	regulatior	3/137	22/18670	5.37E-04	0.001636	0.000651	VEGFA/SM(	3
GO:00508!	antigen re	9/137	316/1867(	5.48E-04	0.001668	0.000664	MAPK1/BCI	9
GO:00518!	membrane c	5/137	91/18670	5.55E-04	0.001685	0.000671	SRC/KDR/I	5
GO:00603:	interferor	5/137	91/18670	5.55E-04	0.001685	0.000671	PPARG/STA	5
GO:00027(	regulatior	6/137	139/1867(	0.000565	0.001715	0.000683	MAPK14/MA	6
GO:00507:	positive r	6/137	139/1867(	0.000565	0.001715	0.000683	MAPK14/MA	6
GO:00091(	nucleotide	10/137	386/1867(	0.000568	0.001722	0.000685	NOS2/MAPI	10
GO:00031	heart valv	4/137	52/18670	0.000572	0.001727	0.000688	MTOR/MDM:	4
GO:00069!	nuclear er	4/137	52/18670	0.000572	0.001727	0.000688	CDK1/PRK(	4
GO:00315:	ruffle org	4/137	52/18670	0.000572	0.001727	0.000688	MTOR/HRAS	4
GO:00721:	mesenchyme	4/137	52/18670	0.000572	0.001727	0.000688	TGFBR1/F(	4
GO:20001	positive r	4/137	52/18670	0.000572	0.001727	0.000688	VEGFA/HII	4
GO:00705	protein lc	6/137	141/1867(	0.00061	0.001832	0.00073	BCL2/HSP!	6
GO:00068:	mitochondr	8/137	256/1867(	0.000612	0.001832	0.00073	GSK3B/BCI	8
GO:19011	regulatior	5/137	93/18670	0.000613	0.001832	0.00073	MMP9/EGFI	5
GO:00074:	axonal fas	3/137	23/18670	0.000615	0.001832	0.00073	CASP3/EPI	3
GO:00346!	response t	3/137	23/18670	0.000615	0.001832	0.00073	PPARG/AKT	3
GO:00487:	positive r	3/137	23/18670	0.000615	0.001832	0.00073	PPARG/MT(	3
GO:00509!	negative r	3/137	23/18670	0.000615	0.001832	0.00073	TNF/AKT1/	3
GO:00604	branching	3/137	23/18670	0.000615	0.001832	0.00073	FGFR1/TNI	3
GO:00722:	regulatior	3/137	23/18670	0.000615	0.001832	0.00073	RET/STAT:	3
GO:01060:	neuron prc	3/137	23/18670	0.000615	0.001832	0.00073	CASP3/EPI	3
GO:19018	negative r	3/137	23/18670	0.000615	0.001832	0.00073	SRC/MMP1	3
GO:00076:	visual bel	4/137	53/18670	0.000615	0.001832	0.00073	KIT/MTOR/	4
GO:00316:	zymogen ac	4/137	53/18670	0.000615	0.001832	0.00073	MMP14/CAS	4
GO:00350(	regulatior	4/137	53/18670	0.000615	0.001832	0.00073	MAPK3/CHI	4
GO:00456(	regulatior	4/137	53/18670	0.000615	0.001832	0.00073	MAPK14/I(	4
GO:00458	positive r	4/137	53/18670	0.000615	0.001832	0.00073	PDGFRB/AI	4
GO:19020!	regulatior	4/137	53/18670	0.000615	0.001832	0.00073	PLK1/CCNI	4
GO:19055:	macrophage	4/137	53/18670	0.000615	0.001832	0.00073	MAPK14/MA	4
GO:19012!	nucleoside	10/137	390/1867(	0.000615	0.001832	0.00073	NOS2/MAPI	10
GO:19005	regulatior	6/137	142/1867(	0.000633	0.001884	0.00075	NOS2/HIF:	6
GO:00447:	DNA methyl	5/137	94/18670	0.000643	0.001912	0.000761	DNMT1/PII	5
GO:00456:	positive r	5/137	94/18670	0.000643	0.001912	0.000761	MMP14/IL:	5
GO:00030:	muscle sys	11/137	465/1867(	0.000644	0.001913	0.000762	KIT/MTOR/	11
GO:00070!	metaphase/	4/137	54/18670	0.00066	0.001959	0.00078	PLK1/CCNI	4
GO:00322:	regulatior	4/137	54/18670	0.00066	0.001959	0.00078	MAPK3/SR(	4
GO:00508(	negative r	7/137	199/1867(	0.000673	0.001996	0.000795	LGALS3/II	7

GO:00027(	positive r	5/137	95/18670	0.000675	0.002	0.000796	MAPK14/MA	5
GO:01100E	regulation	8/137	261/1867(	0.000694	0.002055	0.000818	TGFBR1/MI	8
GO:00716	positive r	3/137	24/18670	0.000699	0.002066	0.000823	LGALS3/TN	3
GO:19055(	positive r	3/137	24/18670	0.000699	0.002066	0.000823	FGFR1/FGI	3
GO:00182(	peptidyl-I	10/137	397/1867(	0.000705	0.002083	0.000829	MAPK3/DNM	10
GO:00902	positive r	5/137	96/18670	0.000708	0.00209	0.000832	HIF1A/EGI	5
GO:00614	regulation	6/137	146/1867(	0.000732	0.002159	0.00086	NOS2/HIF1	6
GO:00447E	metaphase/	4/137	56/18670	0.000758	0.002233	0.000889	PLK1/CCNI	4
GO:00613E	neural pre	6/137	147/1867(	0.000759	0.002233	0.000889	FGFR1/VE(	6
GO:00902(	positive r	6/137	147/1867(	0.000759	0.002233	0.000889	SRC/EGFR/	6
GO:00082(	steroid me	9/137	331/1867(	0.000763	0.002245	0.000894	CYP17A1/I	9
GO:19029(	positive r	7/137	204/1867(	0.000779	0.00229	0.000912	TGFBR1/MI	7
GO:00019	lymph vess	3/137	25/18670	0.00079	0.002309	0.000919	VEGFA/EPI	3
GO:00310(	hair folli	3/137	25/18670	0.00079	0.002309	0.000919	BCL2/SMO/	3
GO:00481(	somatic st	3/137	25/18670	0.00079	0.002309	0.000919	FGFR1/KIT	3
GO:00509	positive r	3/137	25/18670	0.00079	0.002309	0.000919	KDR/VEGFA	3
GO:00612	regulation	3/137	25/18670	0.00079	0.002309	0.000919	VEGFA/SM(	3
GO:00722	metanephri	3/137	25/18670	0.00079	0.002309	0.000919	STAT1/PD(	3
GO:19043E	cellular r	3/137	25/18670	0.00079	0.002309	0.000919	SRC/NFKB1	3
GO:20006	positive r	3/137	25/18670	0.00079	0.002309	0.000919	PARP1/EP3	3
GO:00163	dephosphor	11/137	478/1867(	0.000807	0.002358	0.000939	PTPN11/SI	11
GO:00109(	regulation	4/137	57/18670	0.000811	0.002367	0.000942	PLK1/CCNI	4
GO:00303	negative r	9/137	334/1867(	0.000814	0.002368	0.000943	PPARG/BCI	9
GO:00193	fatty acid	5/137	99/18670	0.000814	0.002368	0.000943	PPARG/MAI	5
GO:00140	response t	6/137	149/1867(	0.000814	0.002368	0.000943	PPARG/STA	6
GO:00485	eye morpho	6/137	149/1867(	0.000814	0.002368	0.000943	BCL2/VEGI	6
GO:20000E	regulation	6/137	149/1867(	0.000814	0.002368	0.000943	GSK3B/AUI	6
GO:00092(	purine rik	9/137	335/1867(	0.000831	0.002413	0.000961	HIF1A/STA	9
GO:00600	canonical	9/137	335/1867(	0.000831	0.002413	0.000961	MAPK14/SI	9
GO:00072	I-kappaB k	8/137	269/1867(	0.000844	0.002451	0.000976	ESR1/STAT	8
GO:01200	positive r	5/137	100/1867(	0.000852	0.002471	0.000984	TGFBR1/KI	5
GO:00192	regulation	4/137	58/18670	0.000866	0.002507	0.000998	PTGS2/EGI	4
GO:00713E	cellular r	4/137	58/18670	0.000866	0.002507	0.000998	EGFR/ICAM	4
GO:19037	positive r	4/137	58/18670	0.000866	0.002507	0.000998	BCL2/CASI	4
GO:00091	nucleoside	7/137	208/1867(	0.000873	0.002524	0.001005	HIF1A/STA	7
GO:00507	regulation	7/137	208/1867(	0.000873	0.002524	0.001005	STAT1/BCI	7
GO:00023(	T cell lin	3/137	26/18670	0.000888	0.002553	0.001017	BCL2/MTOI	3
GO:00220	myelinatic	3/137	26/18670	0.000888	0.002553	0.001017	AKT1/NTRI	3
GO:00322	peripheral	3/137	26/18670	0.000888	0.002553	0.001017	AKT1/NTRI	3
GO:00481(	regulation	3/137	26/18670	0.000888	0.002553	0.001017	KIT/HRAS/	3
GO:00509	regulation	3/137	26/18670	0.000888	0.002553	0.001017	KDR/VEGFA	3
GO:19032(	regulation	3/137	26/18670	0.000888	0.002553	0.001017	HIF1A/PAI	3
GO:19043E	regulation	3/137	26/18670	0.000888	0.002553	0.001017	MAPK3/MAI	3
GO:19050	regulation	3/137	26/18670	0.000888	0.002553	0.001017	MAPK14/MA	3
GO:00344	lipid oxic	5/137	101/1867(	0.000891	0.002558	0.001019	PPARG/MAI	5
GO:00466	alpha-beta	5/137	101/1867(	0.000891	0.002558	0.001019	BCL2/MTOI	5
GO:00091(	purine rik	9/137	340/1867(	0.000922	0.002645	0.001053	HIF1A/STA	9

GO:20003!	regulatior	4/137	59/18670	0.000924	0.002647	0.001054	TERT/KDR/	4
GO:20007!	regulatior	4/137	59/18670	0.000924	0.002647	0.001054	MAPK3/CHI	4
GO:00215	cerebellum	5/137	102/1867(	0.000932	0.002667	0.001062	MAP2K1/PT	5
GO:00091	purine nuc	9/137	341/1867(	0.000941	0.002691	0.001072	HIF1A/STA	9
GO:00091	ribonuclec	9/137	341/1867(	0.000941	0.002691	0.001072	HIF1A/STA	9
GO:00091	purine nuc	9/137	342/1867(	0.000961	0.002745	0.001093	HIF1A/STA	9
GO:19021(	negative r	5/137	103/1867(	0.000973	0.002779	0.001107	CDK6/IL2/	5
GO:00513(	mitotic si	4/137	60/18670	0.000984	0.002808	0.001118	PLK1/CCNI	4
GO:00364	neuron dea	3/137	27/18670	0.000994	0.002832	0.001127	HIF1A/PAI	3
GO:20001!	regulatior	3/137	27/18670	0.000994	0.002832	0.001127	AKT1/AKT2	3
GO:00901(	positive r	5/137	104/1867(	0.001017	0.002893	0.001152	TGFBR1/PA	5
GO:19018(	positive r	5/137	104/1867(	0.001017	0.002893	0.001152	GSK3B/AUI	5
GO:00510!	negative r	6/137	156/1867(	0.001033	0.002938	0.00117	PPARG/SR(	6
GO:00031	heart valv	4/137	61/18670	0.001047	0.002966	0.001181	MTOR/MDM2	4
GO:00308	regulatior	4/137	61/18670	0.001047	0.002966	0.001181	BCL2/IL2/	4
GO:00322	positive r	4/137	61/18670	0.001047	0.002966	0.001181	TGFBR1/MT	4
GO:00713	cellular r	4/137	61/18670	0.001047	0.002966	0.001181	EGFR/ICAM	4
GO:00903(	positive r	4/137	61/18670	0.001047	0.002966	0.001181	MTOR/HRAS	4
GO:20012	positive r	4/137	61/18670	0.001047	0.002966	0.001181	BCL2/BCL2	4
GO:19027!	negative r	5/137	105/1867(	0.001061	0.003003	0.001196	CHEK1/CDI	5
GO:00198	stem cell	6/137	157/1867(	0.001068	0.00302	0.001203	KIT/FGF2/	6
GO:20001	negative r	9/137	349/1867(	0.001107	0.003108	0.001238	PPARG/BCI	9
GO:00336	regulatior	3/137	28/18670	0.001107	0.003108	0.001238	BCL2/FGFI	3
GO:00426	regulatior	3/137	28/18670	0.001107	0.003108	0.001238	TERT/TNF/	3
GO:00454(	regulatior	3/137	28/18670	0.001107	0.003108	0.001238	STAT3/TNI	3
GO:00455	regulatior	3/137	28/18670	0.001107	0.003108	0.001238	MMP14/BAI	3
GO:00523	modulatior	3/137	28/18670	0.001107	0.003108	0.001238	JUN/NTRK3	3
GO:00711(	protein lo	3/137	28/18670	0.001107	0.003108	0.001238	ESR1/PLK1	3
GO:19027	positive r	3/137	28/18670	0.001107	0.003108	0.001238	SRC/MTOR/	3
GO:19037!	negative r	3/137	28/18670	0.001107	0.003108	0.001238	MTOR/AKT1	3
GO:19049!	regulatior	3/137	28/18670	0.001107	0.003108	0.001238	TNF/ICAM1	3
GO:00022	T cell act	5/137	106/1867(	0.001107	0.003108	0.001238	MTOR/LGAI	5
GO:00106	positive r	4/137	62/18670	0.001113	0.003117	0.001241	SRC/AKT1/	4
GO:00108	regulatior	4/137	62/18670	0.001113	0.003117	0.001241	MAPK14/MN	4
GO:00454	bone resor	4/137	62/18670	0.001113	0.003117	0.001241	SRC/PRKCA	4
GO:19058	regulatior	4/137	62/18670	0.001113	0.003117	0.001241	PLK1/CCNI	4
GO:00165	protein de	8/137	283/1867(	0.00117	0.003272	0.001303	AR/ESR1/T	8
GO:00067!	ATP biosyr	6/137	160/1867(	0.001177	0.003288	0.001309	HIF1A/STA	6
GO:00219	neural tub	6/137	160/1867(	0.001177	0.003288	0.001309	HIF1A/EPI	6
GO:00302(	glycosamir	6/137	160/1867(	0.001177	0.003288	0.001309	PDGFRB/F(	6
GO:00025	acute infl	7/137	220/1867(	0.001209	0.003374	0.001344	PPARG/PT(	7
GO:00091(	ribonuclec	9/137	354/1867(	0.001222	0.003407	0.001356	HIF1A/STA	9
GO:00072(	nitric oxi	3/137	29/18670	0.001228	0.003407	0.001356	NOS2/VEGI	3
GO:00108(	positive r	3/137	29/18670	0.001228	0.003407	0.001356	MAPK1/PLI	3
GO:00109!	positive r	3/137	29/18670	0.001228	0.003407	0.001356	SRC/MMP14	3
GO:00140	Schwann ce	3/137	29/18670	0.001228	0.003407	0.001356	AKT1/NTRI	3
GO:00310(	regulatior	3/137	29/18670	0.001228	0.003407	0.001356	VEGFA/MAI	3

GO:00422[illegible]	interleuki	3/137	29/18670	0.001228	0.003407	0.001356	STAT3/TNI	3
GO:00487[illegible]	epidermis	3/137	29/18670	0.001228	0.003407	0.001356	BCL2/SMO/	3
GO:00509[illegible]	detection	3/137	29/18670	0.001228	0.003407	0.001356	KIT/NTRK[illegible]	3
GO:00024[illegible]	productior	8/137	286/1867[illegible]	0.001251	0.003467	0.00138	MAPK14/MA	8
GO:00357[illegible]	insulin se	4/137	64/18670	0.001254	0.003468	0.001381	HIF1A/RAI	4
GO:00456[illegible]	regulatior	4/137	64/18670	0.001254	0.003468	0.001381	MTOR/TNF/	4
GO:20003[illegible]	negative r	4/137	64/18670	0.001254	0.003468	0.001381	BCL2/MMP[illegible]	4
GO:00335[illegible]	unsaturate	5/137	110/1867[illegible]	0.001307	0.003612	0.001438	MAPK3/PT[illegible]	5
GO:00148[illegible]	response t	4/137	65/18670	0.001328	0.003665	0.001459	MTOR/HIF[illegible]	4
GO:00327[illegible]	positive r	4/137	65/18670	0.001328	0.003665	0.001459	HRAS/TNF/	4
GO:00725[illegible]	endothelia	4/137	65/18670	0.001328	0.003665	0.001459	TERT/KDR/	4
GO:00017[illegible]	B cell hom	3/137	30/18670	0.001357	0.003718	0.00148	BCL2/HIF[illegible]	3
GO:00105[illegible]	miRNA meta	3/137	30/18670	0.001357	0.003718	0.00148	HRAS/NFKI	3
GO:00107[illegible]	regulatior	3/137	30/18670	0.001357	0.003718	0.00148	PPARG/PLA	3
GO:00350[illegible]	positive r	3/137	30/18670	0.001357	0.003718	0.00148	MAPK3/IL[illegible]	3
GO:00459[illegible]	positive r	3/137	30/18670	0.001357	0.003718	0.00148	MTOR/TNF/	3
GO:00481[illegible]	negative r	3/137	30/18670	0.001357	0.003718	0.00148	PPARG/GS[illegible]	3
GO:00488[illegible]	homeostasi	3/137	30/18670	0.001357	0.003718	0.00148	PTPN11/B[illegible]	3
GO:00508[illegible]	negative r	3/137	30/18670	0.001357	0.003718	0.00148	CASP3/ATM	3
GO:00715[illegible]	cellular r	3/137	30/18670	0.001357	0.003718	0.00148	EGFR/ICAM	3
GO:00903[illegible]	positive r	3/137	30/18670	0.001357	0.003718	0.00148	HRAS/ERBI	3
GO:19048[illegible]	beta-cater	3/137	30/18670	0.001357	0.003718	0.00148	TERT/EP3[illegible]	3
GO:00509[illegible]	sensory pe	6/137	165/1867[illegible]	0.001379	0.003774	0.001503	FGFR1/KI[illegible]	6
GO:00517[illegible]	positive r	4/137	66/18670	0.001406	0.003844	0.001531	PDGFRB/AI	4
GO:00519[illegible]	negative r	4/137	66/18670	0.001406	0.003844	0.001531	BCL2/PTG[illegible]	4
GO:00032[illegible]	cardiac se	5/137	112/1867[illegible]	0.001416	0.003857	0.001536	TGFBR1/MI	5
GO:00149[illegible]	myotube di	5/137	112/1867[illegible]	0.001416	0.003857	0.001536	MAPK14/MM	5
GO:00508[illegible]	negative r	5/137	112/1867[illegible]	0.001416	0.003857	0.001536	LGALS3/II	5
GO:19035[illegible]	mucopolysa	5/137	112/1867[illegible]	0.001416	0.003857	0.001536	FGF2/AKT[illegible]	5
GO:19907[illegible]	cellular c	5/137	112/1867[illegible]	0.001416	0.003857	0.001536	ABCG2/PT[illegible]	5
GO:00458[illegible]	negative r	9/137	363/1867[illegible]	0.001453	0.003957	0.001576	SRC/MMP9/	9
GO:00332[illegible]	tumor necr	6/137	167/1867[illegible]	0.001466	0.00399	0.001589	STAT1/TNI	6
GO:00026[illegible]	positive r	3/137	31/18670	0.001495	0.004049	0.001612	PTGS2/TNI	3
GO:00107[illegible]	regulatior	3/137	31/18670	0.001495	0.004049	0.001612	FGF2/AKT[illegible]	3
GO:00336[illegible]	osteoblast	3/137	31/18670	0.001495	0.004049	0.001612	BCL2/FGFI	3
GO:00346[illegible]	response t	3/137	31/18670	0.001495	0.004049	0.001612	PPARG/AK[illegible]	3
GO:00458[illegible]	positive r	3/137	31/18670	0.001495	0.004049	0.001612	SMO/GLI1/	3
GO:00703[illegible]	lens fiber	3/137	31/18670	0.001495	0.004049	0.001612	EPHA2/NTI	3
GO:19033[illegible]	positive r	3/137	31/18670	0.001495	0.004049	0.001612	SRC/MMP1[illegible]	3
GO:00066[illegible]	icosanoid	5/137	114/1867[illegible]	0.001531	0.004139	0.001648	MAPK3/AB[illegible]	5
GO:00302[illegible]	bone miner	5/137	114/1867[illegible]	1.53E-03	4.14E-03	1.65E-03	PTGS2/HII	5
GO:00603[illegible]	bone morph	5/137	114/1867[illegible]	1.53E-03	4.14E-03	1.65E-03	MMP14/FGI	5
GO:00108[illegible]	negative r	4/137	68/18670	1.57E-03	4.23E-03	1.69E-03	SRC/MMP1[illegible]	4
GO:00199[illegible]	lipid stor	4/137	68/18670	1.57E-03	4.23E-03	1.69E-03	PPARG/TNI	4
GO:00330[illegible]	regulatior	4/137	68/18670	1.57E-03	4.23E-03	1.69E-03	PLK1/CCNI	4
GO:00458[illegible]	negative r	4/137	68/18670	1.57E-03	4.23E-03	1.69E-03	LGALS3/EI	4
GO:00712[illegible]	cellular r	4/137	68/18670	1.57E-03	4.23E-03	1.69E-03	MAPK3/MAI	4

GO:19033:	regulatior	7/137	231/1867(	1.60E-03	4.31E-03	1.72E-03	TGFBR1/M7	7
GO:00060:	aminoglyca	6/137	170/1867(	1.61E-03	0.00432	1.72E-03	PDGFRB/F(	6
GO:00033:	metanephro	3/137	32/18670	1.64E-03	0.004404	1.75E-03	STAT1/PD(	3
GO:00519(	positive r	3/137	32/18670	1.64E-03	0.004404	1.75E-03	PTGS2/EGI	3
GO:00550(	response t	3/137	32/18670	1.64E-03	0.004404	1.75E-03	PPARG/AK7	3
GO:00606'	placenta k	3/137	32/18670	1.64E-03	0.004404	1.75E-03	MAP2K1/MA	3
GO:00063(	DNA modifi	5/137	116/1867(	1.65E-03	0.004431	1.76E-03	DNMT1/PII	5
GO:00066(	phosphatic	5/137	116/1867(	1.65E-03	0.004431	1.76E-03	PIK3CA/PI	5
GO:00092(	purine rit	6/137	171/1867(	1.65E-03	0.004431	1.76E-03	HIF1A/STA	6
GO:00706	protein mc	8/137	299/1867(	1.66E-03	0.004435	1.77E-03	AR/ESR1/7	8
GO:00702:	lymphocyte	4/137	69/18670	1.66E-03	0.004438	1.77E-03	HIF1A/LGA	4
GO:00163	dendrite c	7/137	233/1867(	1.68E-03	0.004494	1.79E-03	GSK3B/MT(	7
GO:00091	purine nuc	6/137	172/1867(	1.70E-03	0.004549	1.81E-03	HIF1A/STA	6
GO:00328'	positive r	6/137	172/1867(	1.70E-03	0.004549	1.81E-03	VEGFA/HRA	6
GO:19012:	regulatior	5/137	117/1867(	1.72E-03	0.004583	1.82E-03	EGFR/TNF/	5
GO:00107(	regulatior	8/137	301/1867(	1.73E-03	0.004607	1.83E-03	MAP2K1/GS	8
GO:00519(	regulatior	4/137	70/18670	1.75E-03	0.004659	0.001855	PTGS2/EGI	4
GO:00615	myeloid ce	4/137	70/18670	1.75E-03	0.004659	0.001855	PTPN11/SI	4
GO:00104(	regulatior	6/137	173/1867(	1.75E-03	0.004667	0.001858	ESR2/SRC/	6
GO:00703(	positive r	6/137	173/1867(	1.75E-03	0.004667	0.001858	VEGFA/HRA	6
GO:00424'	ear morpho	5/137	118/1867(	1.78E-03	0.00474	0.001887	MAPK3/MAI	5
GO:00100(	specificat	3/137	33/18670	1.80E-03	0.004752	0.001892	AR/FGFR1/	3
GO:00105(	regulatior	3/137	33/18670	1.80E-03	0.004752	0.001892	MTOR/EPHA	3
GO:00162	negative r	3/137	33/18670	1.80E-03	0.004752	0.001892	MTOR/PIK3	3
GO:00357'	positive r	3/137	33/18670	1.80E-03	0.004752	0.001892	HIF1A/BAI	3
GO:00457:	negative r	3/137	33/18670	1.80E-03	0.004752	0.001892	PLK1/CASI	3
GO:20003	negative r	3/137	33/18670	1.80E-03	0.004752	0.001892	TERT/KDR/	3
GO:20007	positive r	3/137	33/18670	0.001795	0.004752	0.001892	MAPK3/IL1	3
GO:00336(	cellular p	4/137	71/18670	0.001843	0.004876	0.001941	GSK3B/MT(	4
GO:00170	regulatior	5/137	120/1867(	0.001919	0.005074	0.00202	TGFBR1/EI	5
GO:00320:	positive r	4/137	72/18670	0.00194	0.005124	0.00204	HIF1A/JAI	4
GO:19037	regulatior	4/137	72/18670	0.00194	0.005124	0.00204	BCL2/CASI	4
GO:00109:	positive r	3/137	34/18670	0.001958	0.005148	0.00205	PDGFRB/M7	3
GO:00322	positive r	3/137	34/18670	0.001958	0.005148	0.00205	MAPK3/MAI	3
GO:00433(	positive l	3/137	34/18670	0.001958	0.005148	0.00205	BCL2/MTOI	3
GO:00708	regulatior	3/137	34/18670	0.001958	0.005148	0.00205	GSK3B/MT(	3
GO:00714(	cellular r	3/137	34/18670	0.001958	0.005148	0.00205	PPARG/AK7	3
GO:01060	regulatior	3/137	34/18670	0.001958	0.005148	0.00205	GSK3B/MT(	3
GO:19040:	negative r	3/137	34/18670	0.001958	0.005148	0.00205	PLK1/CASI	3
GO:00092(	ribonucle(	6/137	177/1867(	0.001969	0.005172	0.002059	HIF1A/STA	6
GO:00506(	cytokine s	7/137	240/1867(	0.001987	0.005217	0.002077	NOS2/MAPI	7
GO:00486'	axon exter	5/137	121/1867(	0.001991	0.005223	0.002079	GSK3B/VE(	5
GO:19035:	positive r	4/137	73/18670	0.002041	0.005353	0.002131	PTGS2/EGI	4
GO:00324:	regulatior	5/137	122/1867(	0.002064	0.005401	0.00215	GSK3B/AUI	5
GO:19035'	regulatior	5/137	122/1867(	0.002064	0.005401	0.00215	HIF1A/STA	5
GO:19038	regulatior	5/137	122/1867(	0.002064	0.005401	0.00215	TGFBR1/EI	5
GO:00032(	endocardia	3/137	35/18670	0.002131	0.005558	0.002213	TGFBR1/MI	3

GO:0021953	central ne	3/137	35/18670	0.002131	0.005558	0.002213	HSP90AA1/	3
GO:0048333	mesodermal	3/137	35/18670	0.002131	0.005558	0.002213	FGFR1/ITG	3
GO:0070423	nucleotide	3/137	35/18670	0.002131	0.005558	0.002213	CASP8/XIA	3
GO:0110111	negative r	3/137	35/18670	0.002131	0.005558	0.002213	STAT1/BCL	3
GO:0051271	negative r	9/137	384/18670	0.002132	0.005558	0.002213	PPARG/BCL	9
GO:0071622	granulocyt	5/137	123/18670	0.002139	0.005572	0.002219	MAPK14/MA	5
GO:0005977	glycogen m	4/137	74/18670	0.002146	0.005585	0.002224	GSK3B/MTO	4
GO:1900006	positive r	4/137	74/18670	0.002146	0.005585	0.002224	MTOR/ALK/	4
GO:0002285	lymphocyte	6/137	181/18670	0.002203	0.005729	0.002281	MTOR/LGAL	6
GO:0001832	embryonic	5/137	124/18670	0.002215	0.005751	0.00229	RET/HIF1A	5
GO:0007098	centrosome	5/137	124/18670	0.002215	0.005751	0.00229	CHEK1/CDK	5
GO:0019221	regulation	5/137	124/18670	0.002215	0.005751	0.00229	VDR/KIT/T	5
GO:0001707	mesoderm f	4/137	75/18670	0.002254	0.005834	0.002323	FGFR1/EPH	4
GO:0003281	ventricula	4/137	75/18670	0.002254	0.005834	0.002323	TGFBR1/MD	4
GO:0006073	cellular g	4/137	75/18670	0.002254	0.005834	0.002323	GSK3B/MTO	4
GO:0010611	regulation	4/137	75/18670	0.002254	0.005834	0.002323	MTOR/PRKC	4
GO:0044042	glucan met	4/137	75/18670	0.002254	0.005834	0.002323	GSK3B/MTO	4
GO:0006805	xenobiotic	5/137	125/18670	0.002294	0.005856	0.002332	HNF4A/CYP	5
GO:0006006	glucose ca	3/137	36/18670	0.002312	0.005856	0.002332	GAPDH/PKM	3
GO:0009111	nucleobase	3/137	36/18670	0.002312	0.005856	0.002332	MAPK1/MTO	3
GO:0010455	regulation	3/137	36/18670	0.002312	0.005856	0.002332	AR/FGFR1/	3
GO:0032200	negative r	3/137	36/18670	0.002312	0.005856	0.002332	SRC/PARP1	3
GO:0034439	smooth mus	3/137	36/18670	0.002312	0.005856	0.002332	PPARG/DNM	3
GO:0034439	regulation	3/137	36/18670	0.002312	0.005856	0.002332	PPARG/DNM	3
GO:0035587	nucleotide	3/137	36/18670	0.002312	0.005856	0.002332	CASP8/XIA	3
GO:0042555	superoxide	3/137	36/18670	0.002312	0.005856	0.002332	EGFR/TGFB	3
GO:0046622	negative r	3/137	36/18670	0.002312	0.005856	0.002332	RPS6KB1/I	3
GO:0051197	positive r	3/137	36/18670	0.002312	0.005856	0.002332	MAPK3/MAP	3
GO:0090311	regulation	3/137	36/18670	0.002312	0.005856	0.002332	HRAS/ERBB	3
GO:0002299	T cell act	2/137	10/18670	0.002314	0.005856	0.002332	LGALS3/IG	2
GO:0003314	membranous	2/137	10/18670	0.002314	0.005856	0.002332	FGFR2/TGF	2
GO:0010744	regulation	2/137	10/18670	0.002314	0.005856	0.002332	VEGFA/EGF	2
GO:0021933	regulation	2/137	10/18670	0.002314	0.005856	0.002332	SMO/GLI1	2
GO:0032023	response t	2/137	10/18670	0.002314	0.005856	0.002332	CASP3/CAS	2
GO:0033483	gas homeos	2/137	10/18670	0.002314	0.005856	0.002332	HIF1A/GST	2
GO:0035676	endothelia	2/137	10/18670	0.002314	0.005856	0.002332	FGFR1/FGF	2
GO:0045799	negative r	2/137	10/18670	0.002314	0.005856	0.002332	MTOR/AKT1	2
GO:0045944	positive r	2/137	10/18670	0.002314	0.005856	0.002332	AR/MTOR	2
GO:0048577	notochord	2/137	10/18670	0.002314	0.005856	0.002332	EPHA2/GLI	2
GO:0051159	response t	2/137	10/18670	0.002314	0.005856	0.002332	MGMT/TYMS	2
GO:0051900	positive r	2/137	10/18670	0.002314	0.005856	0.002332	KDR/PARP1	2
GO:0060034	bone trabe	2/137	10/18670	0.002314	0.005856	0.002332	MMP2/SFRP	2
GO:0070399	response t	2/137	10/18670	0.002314	0.005856	0.002332	MAPK14/RP	2
GO:0071222	cellular r	2/137	10/18670	0.002314	0.005856	0.002332	MAPK14/RP	2
GO:0071677	smooth mus	2/137	10/18670	0.002314	0.005856	0.002332	PDGFRB/GS	2
GO:0072200	cell proli	2/137	10/18670	0.002314	0.005856	0.002332	STAT1/PDG	2
GO:0090003	positive r	2/137	10/18670	0.002314	0.005856	0.002332	VEGFA/FLT	2

GO:00903	positive r	2/137	10/18670	0.002314	0.005856	0.002332	MAPK14/PT	2
GO:01060	regulation	2/137	10/18670	0.002314	0.005856	0.002332	PTGS2/BAI	2
GO:19037	negative r	2/137	10/18670	0.002314	0.005856	0.002332	ESR1/TGFI	2
GO:19038	positive r	2/137	10/18670	0.002314	0.005856	0.002332	CDK1/CCNI	2
GO:19043	regulation	2/137	10/18670	0.002314	0.005856	0.002332	GSK3B/SFI	2
GO:20005	regulation	2/137	10/18670	0.002314	0.005856	0.002332	FGFR1/FGI	2
GO:20012	regulation	2/137	10/18670	0.002314	0.005856	0.002332	CA7/ABCB1	2
GO:00091	purine nuc	6/137	183/1867	0.002327	0.005877	0.00234	HIF1A/STA	6
GO:00091	purine rib	6/137	183/1867	0.002327	0.005877	0.00234	HIF1A/STA	6
GO:00219	central ne	6/137	183/1867	0.002327	0.005877	0.00234	HSP90AA1/	6
GO:19033	regulation	7/137	247/1867	0.002336	0.005895	0.002347	GSK3B/HSI	7
GO:00074	endoderm c	4/137	76/18670	0.002366	0.005964	0.002375	MMP2/MMP9	4
GO:00423	vasoconstr	4/137	76/18670	0.002366	0.005964	0.002375	PTGS2/EGI	4
GO:00324	regulation	5/137	126/1867	0.002375	0.005981	0.002381	PTPN11/ST	5
GO:00460	ADP metabo	5/137	126/1867	0.002375	0.005981	0.002381	HIF1A/STA	5
GO:00483	mesoderm m	4/137	77/18670	0.002482	0.006244	0.002486	FGFR1/EPI	4
GO:00027	positive r	3/137	37/18670	0.002502	0.006278	0.0025	TNF/IL2/T	3
GO:00028	positive r	3/137	37/18670	0.002502	0.006278	0.0025	TNF/IL2/T	3
GO:00431	regulation	3/137	37/18670	0.002502	0.006278	0.0025	SRC/VEGFA	3
GO:19043	positive r	3/137	37/18670	0.002502	0.006278	0.0025	MAPK3/MAI	3
GO:20002	regulation	3/137	37/18670	0.002502	0.006278	0.0025	HRAS/NTRI	3
GO:00303	negative r	6/137	186/1867	0.002524	0.006328	0.00252	ESR2/PPAI	6
GO:00326	type I int	5/137	128/1867	0.002543	0.006373	0.002538	PTPN11/ST	5
GO:00481	regulation	6/137	187/1867	0.002592	0.00649	0.002584	GSK3B/MAI	6
GO:00083	associativ	4/137	78/18670	0.002601	0.00649	0.002584	KIT/MTOR/	4
GO:00085	regulation	4/137	78/18670	0.002601	0.00649	0.002584	SMO/GLI1/	4
GO:00147	regulation	4/137	78/18670	0.002601	0.00649	0.002584	MTOR/PRKC	4
GO:00219	central ne	4/137	78/18670	0.002601	0.00649	0.002584	HSP90AA1/	4
GO:00422	tissue reg	4/137	78/18670	0.002601	0.00649	0.002584	CCNB1/PKM	4
GO:00723	intrinsic	4/137	78/18670	0.002601	0.00649	0.002584	BCL2/MDM2	4
GO:00400	negative r	9/137	396/1867	0.00262	0.006534	0.002602	PPARG/BCI	9
GO:00320	response t	3/137	38/18670	0.002702	0.006723	0.002677	MTOR/CYP1	3
GO:00482	macrophage	3/137	38/18670	0.002702	0.006723	0.002677	MAPK14/MA	3
GO:19000	negative r	3/137	38/18670	0.002702	0.006723	0.002677	RPS6KB1/I	3
GO:20002	negative r	3/137	38/18670	0.002702	0.006723	0.002677	PPARG/SRC	3
GO:00519	regulation	7/137	254/1867	0.00273	0.006786	0.002702	PDGFRB/BC	7
GO:00193	pyridine r	6/137	189/1867	0.002733	0.006786	0.002702	PTGS2/HII	6
GO:00464	nicotinami	6/137	189/1867	0.002733	0.006786	0.002702	PTGS2/HII	6
GO:00987	detoxifica	5/137	131/1867	0.002812	0.006929	0.002759	ABCG2/PTC	5
GO:00059	disacchari	2/137	11/18670	0.002815	0.006929	0.002759	BRAF/SLC2	2
GO:00319	positive r	2/137	11/18670	0.002815	0.006929	0.002759	PTGS2/EPI	2
GO:00331	positive r	2/137	11/18670	0.002815	0.006929	0.002759	AR/PARP1	2
GO:00333	Leydig cel	2/137	11/18670	0.002815	0.006929	0.002759	AR/CCND1	2
GO:00354	histone-se	2/137	11/18670	0.002815	0.006929	0.002759	AURKA/CCN	2
GO:00357	cell chemo	2/137	11/18670	0.002815	0.006929	0.002759	FGFR1/FGI	2
GO:00431	positive r	2/137	11/18670	0.002815	0.006929	0.002759	VEGFA/TGI	2
GO:00457	positive r	2/137	11/18670	0.002815	0.006929	0.002759	RET/CDK4	2

GO:00550	ventricula	2/137	11/18670	0.002815	0.006929	0.002759	CDK1/CCNI	2
GO:00613	cardiac ne	2/137	11/18670	0.002815	0.006929	0.002759	MAPK3/MAI	2
GO:00613	cardiac ne	2/137	11/18670	0.002815	0.006929	0.002759	MAPK3/MAI	2
GO:00718	positive r	2/137	11/18670	0.002815	0.006929	0.002759	SRC/TNF	2
GO:19048	regulation	2/137	11/18670	0.002815	0.006929	0.002759	FGFR1/FGI	2
GO:20012	positive r	2/137	11/18670	0.002815	0.006929	0.002759	CASP8/JAI	2
GO:00330	regulation	4/137	80/18670	0.002852	0.007015	0.002793	PLK1/CCNI	4
GO:00459	regulation	5/137	132/18670	0.002905	0.007134	0.002841	AR/FGFR1/	5
GO:00107	fibroblast	3/137	39/18670	0.002912	0.007134	0.002841	FGF2/AKT1	3
GO:00358	vascular s	3/137	39/18670	0.002912	0.007134	0.002841	DNMT1/KI1	3
GO:00459	positive r	3/137	39/18670	0.002912	0.007134	0.002841	PARP1/IL2	3
GO:00487	regulation	3/137	39/18670	0.002912	0.007134	0.002841	PPARG/MT(	3
GO:19055	regulation	3/137	39/18670	0.002912	0.007134	0.002841	MAPK14/MA	3
GO:20008	regulation	3/137	39/18670	0.002912	0.007134	0.002841	TGFBR1/SI	3
GO:19029	regulation	4/137	81/18670	0.002983	0.007299	0.002906	VDR/TNF/I	4
GO:20010	negative r	4/137	81/18670	0.002983	0.007299	0.002906	BCL2/BCL2	4
GO:00072	smoothened	5/137	133/18670	0.003001	0.007336	0.002921	SMO/GLI1/	5
GO:00310	microtubul	5/137	133/18670	0.003001	0.007336	0.002921	CHEK1/CDI	5
GO:00453	cellular r	6/137	193/18670	0.003031	0.007404	0.002948	NOS2/PIK3	6
GO:00086	phospholip	7/137	260/18670	0.003107	0.007585	0.00302	PLA2G2A/I	7
GO:00900	regulation	4/137	82/18670	0.003118	0.007608	0.003029	KDR/VEGFA	4
GO:00451	regulation	3/137	40/18670	0.003131	0.007631	0.003039	SRC/PRKCA	3
GO:00715	response t	3/137	40/18670	0.003131	0.007631	0.003039	EGFR/ICAI	3
GO:00725	pyridine-c	6/137	195/18670	0.003189	0.007767	0.003093	PTGS2/HII	6
GO:00158	organic ac	8/137	333/18670	0.00322	0.007828	0.003117	NOS2/PPAI	8
GO:00160	lipid cata	8/137	333/18670	0.00322	0.007828	0.003117	PLA2G2A/I	8
GO:00469	carboxylic	8/137	333/18670	0.00322	0.007828	0.003117	NOS2/PPAI	8
GO:00455	positive r	4/137	83/18670	0.003258	0.007912	0.00315	IL2/BAD/1	4
GO:20001	regulation	4/137	83/18670	0.003258	0.007912	0.00315	PIK3CB/HI	4
GO:00091	ribonucleo	6/137	197/18670	0.003353	0.008066	0.003212	HIF1A/STA	6
GO:00434	skeletal m	3/137	41/18670	0.003361	0.008066	0.003212	PKM/EZH2/	3
GO:00482	lung alveo	3/137	41/18670	0.003361	0.008066	0.003212	PGR/FLT4/	3
GO:00508	brown fat	3/137	41/18670	0.003361	0.008066	0.003212	MAPK14/MI	3
GO:00971	ruffle ass	3/137	41/18670	0.003361	0.008066	0.003212	HRAS/ICAI	3
GO:00026	negative r	2/137	12/18670	0.003362	0.008066	0.003212	PPARG/GS1	2
GO:00033	mesenchyma	2/137	12/18670	0.003362	0.008066	0.003212	STAT1/SM(	2
GO:00313	regulation	2/137	12/18670	0.003362	0.008066	0.003212	PTGS2/IL1	2
GO:00331	response t	2/137	12/18670	0.003362	0.008066	0.003212	PPARG/CCI	2
GO:00381	interleuki	2/137	12/18670	0.003362	0.008066	0.003212	IL2/JAK1	2
GO:00450	positive r	2/137	12/18670	0.003362	0.008066	0.003212	TNF/IL1B	2
GO:00454	response t	2/137	12/18670	0.003362	0.008066	0.003212	CDK4/MDM2	2
GO:00455	positive r	2/137	12/18670	0.003362	0.008066	0.003212	MMP14/BAI	2
GO:00515	positive r	2/137	12/18670	0.003362	0.008066	0.003212	MMP9/MTOI	2
GO:00606	branching	2/137	12/18670	0.003362	0.008066	0.003212	FGFR2/GRI	2
GO:00607	epithelial	2/137	12/18670	0.003362	0.008066	0.003212	AR/FGFR2	2
GO:00714	cellular r	2/137	12/18670	0.003362	0.008066	0.003212	ATM/SFRP1	2
GO:00972	negative r	2/137	12/18670	0.003362	0.008066	0.003212	VHL/JUN	2

GO:01500(	regulatior	2/137	12/18670	0.003362	0.008066	0.003212	VEGFA/MAI	2
GO:19041{	positive r	2/137	12/18670	0.003362	0.008066	0.003212	KDR/PARP1	2
GO:20001(	positive r	2/137	12/18670	0.003362	0.008066	0.003212	FGFR1/CDI	2
GO:00454	myoblast c	4/137	84/18670	0.003402	0.008152	0.003246	MAPK14/I(	4
GO:00977	negative r	4/137	84/18670	0.003402	0.008152	0.003246	PTGS2/EGI	4
GO:00091	purine nuc	5/137	138/1867(	0.003516	0.008412	0.003349	HIF1A/STA	5
GO:00091	purine rit	5/137	138/1867(	0.003516	0.008412	0.003349	HIF1A/STA	5
GO:00466	alpha-beta	5/137	138/1867(	0.003516	0.008412	0.003349	BCL2/MTOI	5
GO:00456{	regulatior	4/137	85/18670	0.00355	0.008487	0.003379	VDR/TNF/S	4
GO:00106	negative r	3/137	42/18670	0.0036	0.008593	0.003421	GSK3B/STA	3
GO:00109(	positive r	3/137	42/18670	0.0036	0.008593	0.003421	SRC/AKT1/	3
GO:00171	stem cell	3/137	42/18670	0.0036	0.008593	0.003421	FGFR1/KIT	3
GO:00455{	regulatior	5/137	139/1867(	0.003626	0.008651	0.003444	IL2/ERBB2	5
GO:00420	regulatior	4/137	86/18670	0.003702	0.008826	0.003514	MMP9/EGFI	4
GO:00091{	ribonucle	5/137	140/1867(	0.003739	0.008899	0.003543	HIF1A/STA	5
GO:00350	regulatior	5/137	140/1867(	0.003739	0.008899	0.003543	MET/PDGFI	5
GO:00463	positive r	5/137	140/1867(	0.003739	0.008899	0.003543	HRAS/TNF/	5
GO:00019	negative r	3/137	43/18670	0.003849	0.009131	0.003636	SRC/MMP14	3
GO:00331	calcineuri	3/137	43/18670	0.003849	0.009131	0.003636	GSK3B/MT(	3
GO:00420{	T-helper 1	3/137	43/18670	0.003849	0.009131	0.003636	MTOR/HRAS	3
GO:00457	negative r	3/137	43/18670	0.003849	0.009131	0.003636	MMP14/EGI	3
GO:00516(	response t	3/137	43/18670	0.003849	0.009131	0.003636	SRC/NQO1/	3
GO:19019{	positive r	3/137	43/18670	0.003849	0.009131	0.003636	MAPK3/IL1	3
GO:00975	granulocyt	5/137	141/1867(	0.003854	0.009136	0.003638	MAPK14/MA	5
GO:00026	positive r	4/137	87/18670	0.003859	0.009137	0.003638	MAPK14/MA	4
GO:00061	energy res	4/137	87/18670	0.003859	0.009137	0.003638	GSK3B/MT(	4
GO:00071{	SMAD prote	2/137	13/18670	0.003954	0.009279	0.003695	PARP1/TGI	2
GO:00107(	positive r	2/137	13/18670	0.003954	0.009279	0.003695	AKT1/TGFI	2
GO:00108	positive r	2/137	13/18670	0.003954	0.009279	0.003695	HIF1A/JAI	2
GO:00421	xenobiotic	2/137	13/18670	0.003954	0.009279	0.003695	CYP1A1/GS	2
GO:00426	positive r	2/137	13/18670	0.003954	0.009279	0.003695	TERT/TNF	2
GO:00429(	xenobiotic	2/137	13/18670	0.003954	0.009279	0.003695	ABCC1/SL(	2
GO:00435(	positive r	2/137	13/18670	0.003954	0.009279	0.003695	AR/IGFBP3	2
GO:00509(	detection	2/137	13/18670	0.003954	0.009279	0.003695	NTRK1/CX(	2
GO:00603	regulatior	2/137	13/18670	0.003954	0.009279	0.003695	FLT4/TGFI	2
GO:00605	intestinal	2/137	13/18670	0.003954	0.009279	0.003695	HIF1A/TYM	2
GO:00606(	lateral sp	2/137	13/18670	0.003954	0.009279	0.003695	AR/FGFR2	2
GO:00610	eyelid dev	2/137	13/18670	0.003954	0.009279	0.003695	EGFR/JUN	2
GO:00614	bone trabe	2/137	13/18670	0.003954	0.009279	0.003695	MMP2/SFRI	2
GO:00614(	regulatior	2/137	13/18670	0.003954	0.009279	0.003695	SFRP1/MEI	2
GO:00713	cellular r	2/137	13/18670	0.003954	0.009279	0.003695	IL2/JAK1	2
GO:00905	inflammato	2/137	13/18670	0.003954	0.009279	0.003695	HIF1A/TGI	2
GO:00070	Golgi orga	5/137	142/1867(	0.003971	0.009309	0.003707	MAP2K1/MA	5
GO:00508	positive r	5/137	142/1867(	0.003971	0.009309	0.003707	MMP14/BCI	5
GO:00064	protein ac	6/137	204/1867(	0.003977	0.009313	0.003708	MAPK3/GSI	6
GO:00313	regulatior	6/137	204/1867(	0.003977	0.009313	0.003708	TGFBR1/MT	6
GO:00435(	regulatior	4/137	88/18670	0.00402	0.009406	0.003745	TNF/GSTP1	4

GO:00031	endocardia	3/137	44/18670	0.004109	0.009562	0.003807	TGFBR1/MI	3
GO:00034	endochondı	3/137	44/18670	0.004109	0.009562	0.003807	FGFR2/FGI	3
GO:00329	regulation	3/137	44/18670	0.004109	0.009562	0.003807	PPARG/PD(	3
GO:00358	epithelial	3/137	44/18670	0.004109	0.009562	0.003807	MMP9/STA	3
GO:00440	regulation	3/137	44/18670	0.004109	0.009562	0.003807	PTPN11/F(	3
GO:00456	regulation	3/137	44/18670	0.004109	0.009562	0.003807	VEGFA/TNI	3
GO:00466	response t	3/137	44/18670	0.004109	0.009562	0.003807	CDK1/CYP	3
GO:00509	detection	3/137	44/18670	0.004109	0.009562	0.003807	KIT/NTRK	3
GO:00604	ventricula	3/137	44/18670	0.004109	0.009562	0.003807	TGFBR1/F(	3
GO:00716	regulation	3/137	44/18670	0.004109	0.009562	0.003807	MAPK14/MA	3
GO:00610	somite dev	4/137	89/18670	0.004185	0.009734	0.003876	SMO/ATM/I	4
GO:00067	oxidoreduc	6/137	207/1867(	0.00427	0.009925	0.003952	PTGS2/HII	6
GO:00306	regulation	4/137	90/18670	0.004355	0.010113	0.004027	MAPK3/CA	4
GO:00451	meiotic ch	4/137	90/18670	0.004355	0.010113	0.004027	TOP2A/PLI	4
GO:00019	blood vess	3/137	45/18670	0.004379	0.010145	0.00404	FLT4/MDM	3
GO:00323	icosanoid	3/137	45/18670	0.004379	0.010145	0.00404	NOS2/PLA	3
GO:00359	endodermal	3/137	45/18670	0.004379	0.010145	0.00404	MMP2/MMP	3
GO:00427	intrinsic	3/137	45/18670	0.004379	0.010145	0.00404	BCL2/CHEI	3
GO:20002	regulation	5/137	146/1867(	0.004468	0.010344	0.004119	AR/ESR1/S	5
GO:19029	regulation	8/137	352/1867(	0.004493	0.010398	0.00414	TGFBR1/MI	8
GO:00507	regulation	6/137	210/1867(	0.004578	0.010537	0.004196	MAPK14/MA	6
GO:00071	mitotic ce	2/137	14/18670	0.004591	0.010537	0.004196	CHEK1/AUI	2
GO:00323	prostaglar	2/137	14/18670	0.004591	0.010537	0.004196	NOS2/IL1I	2
GO:00357	platelet-c	2/137	14/18670	0.004591	0.010537	0.004196	SRC/PDGFI	2
GO:00455	positive r	2/137	14/18670	0.004591	0.010537	0.004196	IL2/TGFB	2
GO:00515	regulation	2/137	14/18670	0.004591	0.010537	0.004196	MMP9/MTOI	2
GO:00600	Sertoli ce	2/137	14/18670	0.004591	0.010537	0.004196	ICAM1/NTI	2
GO:00603	JAK-STAT c	2/137	14/18670	0.004591	0.010537	0.004196	STAT3/JAI	2
GO:00706	response t	2/137	14/18670	0.004591	0.010537	0.004196	IL2/JAK1	2
GO:00708	negative r	2/137	14/18670	0.004591	0.010537	0.004196	GSK3B/MT(	2
GO:00722	positive r	2/137	14/18670	0.004591	0.010537	0.004196	RET/PDGFI	2
GO:00972	activation	2/137	14/18670	0.004591	0.010537	0.004196	CASP8/BAI	2
GO:01060	negative r	2/137	14/18670	0.004591	0.010537	0.004196	GSK3B/MT(	2
GO:19033	cellular r	2/137	14/18670	0.004591	0.010537	0.004196	MAPK3/MAI	2
GO:20012	regulation	2/137	14/18670	0.004591	0.010537	0.004196	PTGS2/IL	2
GO:00193	nicotinami	5/137	147/1867(	0.004598	0.010541	0.004197	PTGS2/HII	5
GO:00193	pyridine r	5/137	147/1867(	0.004598	0.010541	0.004197	PTGS2/HII	5
GO:00108	regulation	3/137	46/18670	0.00466	0.01066	0.004244	PPARG/TNI	3
GO:00308	positive r	3/137	46/18670	0.00466	0.01066	0.004244	NOS2/HIF	3
GO:00977	calcineuri	3/137	46/18670	0.00466	0.01066	0.004244	GSK3B/MT(	3
GO:19003	positive r	3/137	46/18670	0.00466	0.01066	0.004244	NOS2/HIF	3
GO:00357	CD4-positi	4/137	92/18670	0.00471	0.010762	0.004285	MTOR/STA	4
GO:00458	negative r	4/137	92/18670	0.00471	0.010762	0.004285	TNF/AKT1/	4
GO:00515	response t	5/137	148/1867(	0.004731	0.010805	0.004302	EGFR/CCNI	5
GO:00068	calcium ic	9/137	434/1867(	0.004776	0.010901	0.004341	VDR/PDGFI	9
GO:00507	regulation	5/137	149/1867(	0.004867	0.011104	0.004421	GSK3B/MT(	5
GO:00064	protein de	4/137	93/18670	0.004894	0.01114	0.004435	VEGFA/MAI	4

GO:00075{	body fluic	4/137	93/18670	0.004894	0.01114	0.004435	VDR/VEGF/	4
GO:00352<	synaptic t	4/137	93/18670	0.004894	0.01114	0.004435	PTGS2/EGI	4
GO:00487(	embryonic	4/137	93/18670	0.004894	0.01114	0.004435	TGFBR1/MN	4
GO:00512{	protein pc	7/137	283/1867(	0.004931	0.011203	0.004461	MET/MTOR/	7
GO:00107{	regulatior	3/137	47/18670	0.004951	0.011203	0.004461	MAPK1/PLI	3
GO:00302(	chromosome	3/137	47/18670	0.004951	0.011203	0.004461	CDK1/TOP2	3
GO:00319{	regulatior	3/137	47/18670	0.004951	0.011203	0.004461	SRC/VEGF/	3
GO:00425{	neuron mat	3/137	47/18670	0.004951	0.011203	0.004461	RET/BCL2/	3
GO:00450{	T cell sel	3/137	47/18670	0.004951	0.011203	0.004461	BCL2/MTOI	3
GO:00488(	genitalia	3/137	47/18670	0.004951	0.011203	0.004461	AR/ESR1/I	3
GO:00609{	endocrine	3/137	47/18670	0.004951	0.011203	0.004461	PTPN11/F(	3
GO:00613{	trabecula	3/137	47/18670	0.004951	0.011203	0.004461	TGFBR1/MN	3
GO:00702:	T cell apc	3/137	47/18670	0.004951	0.011203	0.004461	HIF1A/LGA	3
GO:00988(	bone growt	3/137	47/18670	0.004951	0.011203	0.004461	FGFR2/FGI	3
GO:00083(	regulatior	5/137	150/1867(	0.005006	0.011309	0.004503	KDR/KIT/V	5
GO:00507:	negative r	5/137	150/1867(	0.005006	0.011309	0.004503	PPARG/LGA	5
GO:00725:	pyridine-c	5/137	150/1867(	0.005006	0.011309	0.004503	PTGS2/HII	5
GO:19030{	regulatior	6/137	214/1867(	0.005013	0.01132	0.004507	GSK3B/AUI	6
GO:00349:	response t	7/137	285/1867(	0.005121	0.011556	0.004601	GSK3B/BCI	7
GO:00199:	second-mes	9/137	439/1867(	0.00514	0.011594	0.004616	NOS2/KDR/	9
GO:00024(	adaptive i	8/137	361/1867(	0.005217	0.011704	0.00466	MTOR/STAT	8
GO:00026{	positive r	6/137	216/1867(	0.005242	0.011704	0.00466	MAPK14/MA	6
GO:00027(	negative r	3/137	48/18670	0.005253	0.011704	0.00466	CDK6/PIK3	3
GO:00550]	ventricula	3/137	48/18670	0.005253	0.011704	0.00466	TGFBR1/F(	3
GO:00726(	interleuki	3/137	48/18670	0.005253	0.011704	0.00466	NOS2/TNF/	3
GO:00107{	positive r	2/137	15/18670	0.005272	0.011704	0.00466	MAPK14/MA	2
GO:00170]	protein ni	2/137	15/18670	0.005272	0.011704	0.00466	NOS2/GAPI	2
GO:00181]	peptidyl-c	2/137	15/18670	0.005272	0.011704	0.00466	NOS2/GAPI	2
GO:00336{	negative r	2/137	15/18670	0.005272	0.011704	0.00466	BCL2/SFRI	2
GO:00343<	glial cell	2/137	15/18670	0.005272	0.011704	0.00466	PRKCA/CAS	2
GO:00364<	neuronal s	2/137	15/18670	0.005272	0.011704	0.00466	FGFR1/FGI	2
GO:00421{	lipoprotei	2/137	15/18670	0.005272	0.011704	0.00466	CTSD/ATM	2
GO:00435(	negative r	2/137	15/18670	0.005272	0.011704	0.00466	GSTP1/SFI	2
GO:00450:	regulatior	2/137	15/18670	0.005272	0.011704	0.00466	TNF/IL1B	2
GO:00463:	positive r	2/137	15/18670	0.005272	0.011704	0.00466	PPARG/AKT	2
GO:00483(	lateral me	2/137	15/18670	0.005272	0.011704	0.00466	FGFR1/SM(	2
GO:00509(	detection	2/137	15/18670	0.005272	0.011704	0.00466	NTRK1/CX(	2
GO:00509(	detection	2/137	15/18670	0.005272	0.011704	0.00466	NTRK1/CX(	2
GO:00510<	positive r	2/137	15/18670	0.005272	0.011704	0.00466	TNF/IL1B	2
GO:00512{	centrosome	2/137	15/18670	0.005272	0.011704	0.00466	CHEK1/AUI	2
GO:00518{	killing of	2/137	15/18670	0.005272	0.011704	0.00466	GAPDH/BAI	2
GO:00550{	neuroblast	2/137	15/18670	0.005272	0.011704	0.00466	FGFR1/FGI	2
GO:00703(	hepatocyte	2/137	15/18670	0.005272	0.011704	0.00466	HNF4A/CYI	2
GO:00718(	regulatior	2/137	15/18670	0.005272	0.011704	0.00466	SRC/TNF	2
GO:00720:	metanephri	2/137	15/18670	0.005272	0.011704	0.00466	STAT1/PD(	2
GO:00722{	metanephri	2/137	15/18670	0.005272	0.011704	0.00466	STAT1/SM(	2
GO:19029(	mitotic DN	2/137	15/18670	0.005272	0.011704	0.00466	FGFR1/CHI	2

GO:19033!	response t	2/137	15/18670	0.005272	0.011704	0.00466	MAPK3/MAI	2
GO:00421(	B cell pr(	4/137	95/18670	0.005276	0.011704	0.00466	BCL2/IL2/	4
GO:00421]	macrophage	4/137	95/18670	0.005276	0.011704	0.00466	TNF/JUN/J	4
GO:00508]	regulatior	4/137	95/18670	0.005276	0.011704	0.00466	VDR/TNF/N	4
GO:00464?	glyceropho	6/137	217/1867(	0.00536	0.011883	0.004731	PLA2G2A/I	6
GO:00106(	regulatior	4/137	96/18670	0.005475	0.012102	0.004819	PPARG/DNM	4
GO:00327!	positive r	4/137	96/18670	0.005475	0.012102	0.004819	PTPN11/ST	4
GO:00973?	response t	4/137	96/18670	0.005475	0.012102	0.004819	EGFR/CDK4	4
GO:00060(	alcohol me	8/137	364/1867(	0.005478	0.012102	0.004819	VDR/FGFR]	8
GO:00197?	calcium-me	6/137	218/1867(	0.005479	0.012102	0.004819	KDR/GSK3I	6
GO:00322?	regulatior	6/137	218/1867(	0.005479	0.012102	0.004819	MET/MTOR/	6
GO:00508(	regulatior	6/137	218/1867(	0.005479	0.012102	0.004819	MAPK14/TN	6
GO:19900!	cellular r	3/137	49/18670	0.005566	0.012289	0.004893	AKT1/NTRI	3
GO:00099!	anterior/p	6/137	219/1867(	0.0056	0.012357	0.00492	TGFBR1/AU	6
GO:00030]	heart proc	7/137	290/1867(	0.005621	0.012396	0.004936	SRC/MTOR/	7
GO:00140?	primary ne	4/137	97/18670	0.005679	0.012504	0.004979	HIF1A/CAS	4
GO:01201(	positive r	4/137	97/18670	0.005679	0.012504	0.004979	IGF1R/VE(	4
GO:20010?	positive r	4/137	97/18670	0.005679	0.012504	0.004979	EGFR/PARI	4
GO:19037(	negative r	5/137	155/1867(	0.005742	0.012638	0.005032	CDK6/IL2/	5
GO:00068!	regulatior	4/137	98/18670	0.005887	0.012915	0.005142	MAPK3/CA?	4
GO:00313?	regulatior	4/137	98/18670	0.005887	0.012915	0.005142	NOS2/GAPI	4
GO:00507(	regulatior	4/137	98/18670	0.005887	0.012915	0.005142	PPARG/TNI	4
GO:00017(	endoderm f	3/137	50/18670	0.00589	0.012915	0.005142	MMP2/MMP!	3
GO:00094(	response t	3/137	50/18670	0.00589	0.012915	0.005142	PPARG/HSI	3
GO:00468!	phosphatic	3/137	50/18670	0.00589	0.012915	0.005142	PIK3CA/PI	3
GO:00609?	coronary v	3/137	50/18670	0.00589	0.012915	0.005142	TGFBR1/PI	3
GO:00165?	histone ac	5/137	156/1867(	0.005898	0.012919	0.005144	MAPK3/CHI	5
GO:00452]	cell-cell	5/137	156/1867(	0.005898	0.012919	0.005144	TGFBR1/PI	5
GO:00019?	lymphangic	2/137	16/18670	0.005997	0.013025	0.005186	EPHA2/FL?	2
GO:00022!	T-helper c	2/137	16/18670	0.005997	0.013025	0.005186	MTOR/STA?	2
GO:00215?	dentate gy	2/137	16/18670	0.005997	0.013025	0.005186	CDK6/SMO	2
GO:00302]	hyaluronar	2/137	16/18670	0.005997	0.013025	0.005186	FGF2/TGFI	2
GO:00308!	negative r	2/137	16/18670	0.005997	0.013025	0.005186	CASP3/ATN	2
GO:00341]	positive r	2/137	16/18670	0.005997	0.013025	0.005186	TNF/IL1B	2
GO:00420?	chemokine	2/137	16/18670	0.005997	0.013025	0.005186	TNF/IL1B	2
GO:00507!	chemokine	2/137	16/18670	0.005997	0.013025	0.005186	TNF/IL1B	2
GO:00550]	ventricula	2/137	16/18670	0.005997	0.013025	0.005186	CDK1/CCNI	2
GO:00605?	morphogene	2/137	16/18670	0.005997	0.013025	0.005186	AR/FGFR2	2
GO:00722?	metanephri	2/137	16/18670	0.005997	0.013025	0.005186	RET/PDGFI	2
GO:00900?	regulatior	2/137	16/18670	0.005997	0.013025	0.005186	VEGFA/FL?	2
GO:00970(	cellular r	2/137	16/18670	0.005997	0.013025	0.005186	CTSB/KIT	2
GO:19020(	fatty acic	2/137	16/18670	0.005997	0.013025	0.005186	AKT1/AKT2	2
GO:19020(	positive r	2/137	16/18670	0.005997	0.013025	0.005186	TNF/CASP3	2
GO:20011?	negative r	2/137	16/18670	0.005997	0.013025	0.005186	STAT3/PAI	2
GO:00091?	nucleoside	5/137	158/1867(	0.006219	0.01348	0.005367	HIF1A/STA	5
GO:00164!	cytosolic	5/137	158/1867(	0.006219	0.01348	0.005367	MAP2K1/MA	5
GO:00027]	regulatior	3/137	51/18670	0.006225	0.01348	0.005367	TNF/IL2/?	3

GO:00028{	regulatior	3/137	51/18670	0.006225	0.01348	0.005367	TNF/IL2/7	3
GO:00193(	arachidoni	3/137	51/18670	0.006225	0.01348	0.005367	MAPK3/PT(	3
GO:00322(	positive r	3/137	51/18670	0.006225	0.01348	0.005367	MAPK3/MAI	3
GO:00018:	negative r	7/137	296/1867(	0.006267	0.013564	0.005401	FGFR1/EPI	7
GO:00028:	positive r	4/137	100/1867(	0.006319	0.013656	0.005437	TNF/IL2/1	4
GO:00106!	muscle cel	4/137	100/1867(	0.006319	0.013656	0.005437	PPARG/DNI	4
GO:00704!	interleuki	4/137	100/1867(	0.006319	0.013656	0.005437	MAPK3/NFI	4
GO:19035:	regulatior	7/137	297/1867(	0.00638	0.01378	0.005487	PTGS2/EGI	7
GO:00026:	regulatior	5/137	159/1867(	0.006384	0.013781	0.005487	PPARG/PT(	5
GO:00000:	regulatior	4/137	101/1867(	0.006543	0.014096	0.005612	CHEK1/PAI	4
GO:00315:	actin cyto	4/137	101/1867(	0.006543	0.014096	0.005612	KIT/HRAS/	4
GO:00326:	interleuki	4/137	101/1867(	0.006543	0.014096	0.005612	CASP8/JAI	4
GO:00326	regulatior	4/137	101/1867(	0.006543	0.014096	0.005612	HRAS/TNF/	4
GO:00075:	skeletal m	5/137	160/1867(	0.006552	0.014107	0.005617	MAPK14/B(	5
GO:00465{	negative r	3/137	52/18670	0.006571	0.014119	0.005622	MET/ITGB:	3
GO:00481(	regulatior	3/137	52/18670	0.006571	0.014119	0.005622	KIT/HRAS/	3
GO:19900{	response t	3/137	52/18670	0.006571	0.014119	0.005622	AKT1/NTRI	3
GO:20006:	regulatior	3/137	52/18670	0.006571	0.014119	0.005622	PARP1/EP:	3
GO:00508(	regulatior	6/137	227/1867(	0.006641	0.014262	0.005679	MAPK14/TI	6
GO:00183!	internal p	5/137	161/1867(	0.006723	0.014341	0.00571	MAPK3/CHI	5
GO:00326:	interleuki	5/137	161/1867(	0.006723	0.014341	0.00571	NOS2/PTPI	5
GO:00068:	cellular c	9/137	458/1867(	0.006726	0.014341	0.00571	ESR1/VDR/	9
GO:00061(	purine nuc	7/137	300/1867(	0.006728	0.014341	0.00571	NOS2/HIF:	7
GO:00031!	epithelial	2/137	17/18670	0.006764	0.014341	0.00571	TGFBR1/T(	2
GO:00062(	DNA ligati	2/137	17/18670	0.006764	0.014341	0.00571	TOP2A/MGI	2
GO:00102:	response t	2/137	17/18670	0.006764	0.014341	0.00571	BCL2/REL/	2
GO:00107	positive r	2/137	17/18670	0.006764	0.014341	0.00571	PLA2G2A/I	2
GO:00157:	prostaglar	2/137	17/18670	0.006764	0.014341	0.00571	NOS2/IL1I	2
GO:00170{	response t	2/137	17/18670	0.006764	0.014341	0.00571	CYP1A1/C(	2
GO:00324(	negative r	2/137	17/18670	0.006764	0.014341	0.00571	SRC/EP30(	2
GO:00327	positive r	2/137	17/18670	0.006764	0.014341	0.00571	IL2/TGFB:	2
GO:00331	positive r	2/137	17/18670	0.006764	0.014341	0.00571	AR/PARP1	2
GO:00332(	regulatior	2/137	17/18670	0.006764	0.014341	0.00571	FGFR1/CHI	2
GO:00359{	chondrocyt	2/137	17/18670	0.006764	0.014341	0.00571	MMP14/FGI	2
GO:00433:	CD4-positi	2/137	17/18670	0.006764	0.014341	0.00571	MTOR/STA:	2
GO:00439:	positive r	2/137	17/18670	0.006764	0.014341	0.00571	JUN/EP30(	2
GO:00457:	positive r	2/137	17/18670	0.006764	0.014341	0.00571	AKT1/AKT:	2
GO:00506(	hydrogen p	2/137	17/18670	0.006764	0.014341	0.00571	STAT3/CYI	2
GO:00518:	disruptior	2/137	17/18670	0.006764	0.014341	0.00571	GAPDH/BAI	2
GO:00612!	retina vas	2/137	17/18670	0.006764	0.014341	0.00571	PDGFRB/H:	2
GO:00901{	negative r	2/137	17/18670	0.006764	0.014341	0.00571	MMP9/STA:	2
GO:19032(	positive r	2/137	17/18670	0.006764	0.014341	0.00571	MMP3/MCL:	2
GO:19043!	positive r	2/137	17/18670	0.006764	0.014341	0.00571	MAPK3/MAI	2
GO:19053:	negative r	2/137	17/18670	0.006764	0.014341	0.00571	STAT1/TNI	2
GO:00988(	cellular c	4/137	102/1867(	0.006772	0.014352	0.005715	PTGS2/TNI	4
GO:00329(	collagen b	3/137	53/18670	0.006929	0.014646	0.005832	PPARG/PD(	3
GO:00380(	p38MAPK ca	3/137	53/18670	0.006929	0.014646	0.005832	MAPK14/VI	3

GO:00433:	response t	3/137	53/18670	0.006929	0.014646	0.005832	MAPK3/MAI	3
GO:00702:	regulation	3/137	53/18670	0.006929	0.014646	0.005832	HIF1A/LGA	3
GO:19011	negative r	3/137	53/18670	0.006929	0.014646	0.005832	EGFR/ERBI	3
GO:00722:	integrin-m	4/137	103/1867(	0.007007	0.014781	0.005885	PTPN11/SI	4
GO:00356(	protein de	4/137	103/1867(	0.007007	0.014781	0.005885	VEGFA/MAI	4
GO:00442(	cellular p	4/137	103/1867(	0.007007	0.014781	0.005885	GSK3B/MT(	4
GO:00519	regulation	4/137	103/1867(	0.007007	0.014781	0.005885	PLK1/CCNI	4
GO:00312	biomineral	5/137	163/1867(	0.007074	0.014917	0.005939	PTGS2/HII	5
GO:00463	carboxylic	9/137	462/1867(	0.007103	0.014969	0.00596	PTGS2/HII	9
GO:00160	organic ac	9/137	463/1867(	0.0072	0.015165	0.006038	PTGS2/HII	9
GO:00987	macromolec	4/137	104/1867(	0.007246	0.015256	0.006074	VEGFA/MAI	4
GO:00027	positive r	3/137	54/18670	0.007297	0.015317	0.006099	MAPK14/MA	3
GO:00326	regulation	3/137	54/18670	0.007297	0.015317	0.006099	MAPK14/NI	3
GO:00433	negative r	3/137	54/18670	0.007297	0.015317	0.006099	JUN/JAK2/	3
GO:00457	positive r	3/137	54/18670	0.007297	0.015317	0.006099	KIT/STAT:	3
GO:00610(	cell diffe	3/137	54/18670	0.007297	0.015317	0.006099	MMP9/STA	3
GO:00903	regulation	3/137	54/18670	0.007297	0.015317	0.006099	FGFR1/CDI	3
GO:00018	neural tub	4/137	105/1867(	0.007491	0.015692	0.006248	HIF1A/CAS	4
GO:00027(	positive r	4/137	105/1867(	0.007491	0.015692	0.006248	TNF/IL2/I	4
GO:00028	positive r	4/137	105/1867(	0.007491	0.015692	0.006248	TNF/IL2/I	4
GO:00070(	plasma mem	4/137	105/1867(	0.007491	0.015692	0.006248	AR/AKT1/A	4
GO:00072	positive r	2/137	18/18670	0.007573	0.015729	0.006263	STAT1/EP:	2
GO:00308(	negative r	2/137	18/18670	0.007573	0.015729	0.006263	STAT3/PAI	2
GO:00310(	positive r	2/137	18/18670	0.007573	0.015729	0.006263	VEGFA/TGI	2
GO:00430	myeloid de	2/137	18/18670	0.007573	0.015729	0.006263	TGFB1/TGI	2
GO:00443	cellular r	2/137	18/18670	0.007573	0.015729	0.006263	STAT3/CCI	2
GO:00456	negative r	2/137	18/18670	0.007573	0.015729	0.006263	SMO/EZH2	2
GO:00457	positive r	2/137	18/18670	0.007573	0.015729	0.006263	PRKCA/EGI	2
GO:00468	positive r	2/137	18/18670	0.007573	0.015729	0.006263	PRKCA/EGI	2
GO:00510	regulation	2/137	18/18670	0.007573	0.015729	0.006263	TNF/IL2	2
GO:00518	negative r	2/137	18/18670	0.007573	0.015729	0.006263	SRC/MMP14	2
GO:00708	positive r	2/137	18/18670	0.007573	0.015729	0.006263	AKT1/AKT:	2
GO:00716(	monocyte c	2/137	18/18670	0.007573	0.015729	0.006263	IL1B/GSTI	2
GO:00716	regulation	2/137	18/18670	0.007573	0.015729	0.006263	IL1B/GSTI	2
GO:00720	renal vesi	2/137	18/18670	0.007573	0.015729	0.006263	STAT1/SM(	2
GO:00970	craniofaci	2/137	18/18670	0.007573	0.015729	0.006263	MMP14/TGI	2
GO:01500	positive r	2/137	18/18670	0.007573	0.015729	0.006263	TNF/IL1B	2
GO:19003	negative r	2/137	18/18670	0.007573	0.015729	0.006263	STAT3/PAI	2
GO:00064	internal p	5/137	166/1867(	0.007625	0.01583	0.006303	MAPK3/CHI	5
GO:00032	ventricula	3/137	55/18670	0.007677	0.015899	0.006331	TGFBR1/F(	3
GO:00162	telomere c	3/137	55/18670	0.007677	0.015899	0.006331	MAPK3/MAI	3
GO:00422	response t	3/137	55/18670	0.007677	0.015899	0.006331	MTOR/CCNA	3
GO:00456	negative r	3/137	55/18670	0.007677	0.015899	0.006331	IL2/ERBB:	3
GO:00480	inositol p	3/137	55/18670	0.007677	0.015899	0.006331	GSK3B/MT(	3
GO:00070	mitotic sp	4/137	106/1867(	0.007742	0.016016	0.006377	AURKA/PLI	4
GO:00519(	regulation	4/137	106/1867(	0.007742	0.016016	0.006377	PTK2/NTRI	4
GO:19015(	fatty acid	5/137	167/1867(	0.007815	0.016161	0.006435	MAPK3/AB(	5

GO:00435(	regulatior	4/137	107/1867(	0.007997	0.016529	0.006581	MTOR/PRK(	4
GO:00550'	calcium ic	9/137	471/1867(	0.008011	0.016548	0.006589	ESR1/VDR/	9
GO:00094:	NAD biosyr	3/137	56/18670	0.008069	0.016636	0.006624	PTGS2/GAI	3
GO:00326:	interleuki	3/137	56/18670	0.008069	0.016636	0.006624	MAPK14/NI	3
GO:00985	cellular r	3/137	56/18670	0.008069	0.016636	0.006624	MAPK14/B(	3
GO:19032(	negative r	3/137	56/18670	0.008069	0.016636	0.006624	MET/HIF1/	3
GO:00183(	peptidyl-l	5/137	169/1867(	0.008205	0.0169	0.006729	MAPK3/CHI	5
GO:00605:	skeletal n	5/137	169/1867(	0.008205	0.0169	0.006729	MAPK14/B(	5
GO:00300(	cellular n	4/137	108/1867(	0.008259	0.017002	0.00677	MAPK3/CA'	4
GO:00725:	purine-cor	7/137	313/1867(	0.008401	0.017171	0.006837	NOS2/HIF:	7
GO:00029:	positive r	2/137	19/18670	0.008423	0.017171	0.006837	TNF/IL1B	2
GO:00105:	negative r	2/137	19/18670	0.008423	0.017171	0.006837	BCL2/TGFI	2
GO:00108	negative r	2/137	19/18670	0.008423	0.017171	0.006837	PPARG/TNI	2
GO:00160	detection	2/137	19/18670	0.008423	0.017171	0.006837	NTRK1/CX(	2
GO:00320:	response t	2/137	19/18670	0.008423	0.017171	0.006837	CCND1/MDN	2
GO:00427	mRNA trans	2/137	19/18670	0.008423	0.017171	0.006837	HIF1A/ST/	2
GO:00515	keratinocy	2/137	19/18670	0.008423	0.017171	0.006837	MMP9/MTOI	2
GO:00602:	mesenchyma	2/137	19/18670	0.008423	0.017171	0.006837	STAT1/SM(	2
GO:00607:	labyrinthi	2/137	19/18670	0.008423	0.017171	0.006837	MAPK1/AK'	2
GO:00718(	podosome a	2/137	19/18670	0.008423	0.017171	0.006837	SRC/TNF	2
GO:00720'	kidney mes	2/137	19/18670	0.008423	0.017171	0.006837	STAT1/PD(	2
GO:00720	renal vesi	2/137	19/18670	0.008423	0.017171	0.006837	STAT1/SM(	2
GO:00902(	negative r	2/137	19/18670	0.008423	0.017171	0.006837	BCL2L1/AI	2
GO:19021'	negative r	2/137	19/18670	0.008423	0.017171	0.006837	HIF1A/AK'	2
GO:19052	vascular a	2/137	19/18670	0.008423	0.017171	0.006837	PPARG/DNN	2
GO:19054	regulatior	2/137	19/18670	0.008423	0.017171	0.006837	PPARG/DNN	2
GO:20000:	positive r	2/137	19/18670	0.008423	0.017171	0.006837	TNF/AKT1	2
GO:20002	positive r	2/137	19/18670	0.008423	0.017171	0.006837	HRAS/NTRI	2
GO:20006(	regulatior	2/137	19/18670	0.008423	0.017171	0.006837	MMP9/STA'	2
GO:00017	neural cre	3/137	57/18670	0.008472	0.017238	0.006864	RET/HIF1/	3
GO:00193:	hexose cat	3/137	57/18670	0.008472	0.017238	0.006864	GAPDH/PKN	3
GO:00611'	regulatior	3/137	57/18670	0.008472	0.017238	0.006864	HIF1A/BAI	3
GO:19004(	negative r	3/137	57/18670	0.008472	0.017238	0.006864	MET/HIF1/	3
GO:00016'	long-chair	4/137	109/1867(	0.008525	0.017337	0.006903	MAPK3/PT(	4
GO:00109	regulatior	8/137	394/1867(	0.008659	0.017601	0.007008	PDGFRB/B(	8
GO:00060:	glycosamir	4/137	110/1867(	0.008798	0.017874	0.007117	PDGFRB/NI	4
GO:01400:	meiotic nu	5/137	172/1867(	0.008816	0.017901	0.007128	AURKA/TOI	5
GO:00068(	receptor-n	7/137	316/1867(	0.008827	0.017917	0.007134	PIK3CB/VI	7
GO:00327:	positive r	3/137	58/18670	0.008887	0.018012	0.007172	HIF1A/TNI	3
GO:00420(	T-helper c	3/137	58/18670	0.008887	0.018012	0.007172	MTOR/STA'	3
GO:00510	negative r	3/137	58/18670	0.008887	0.018012	0.007172	MET/ITGB:	3
GO:00066	glycerophc	7/137	319/1867(	0.009269	0.018705	0.007448	PLA2G2A/I	7
GO:00072(	positive r	7/137	319/1867(	0.009269	0.018705	0.007448	ESR1/BCL:	7
GO:00328(	regulatior	3/137	59/18670	0.009314	0.018705	0.007448	AKT1/AKT:	3
GO:00421:	negative r	3/137	59/18670	0.009314	0.018705	0.007448	ERBB2/CA:	3
GO:19028	negative r	3/137	59/18670	0.009314	0.018705	0.007448	MET/HIF1/	3
GO:00023:	lymphoid r	2/137	20/18670	0.009314	0.018705	0.007448	KIT/BCL2	2

GO:00070(	mitotic G2	2/137	20/18670	0.009314	0.018705	0.007448	CDK1/ATM	2
GO:00105(	positive r	2/137	20/18670	0.009314	0.018705	0.007448	MTOR/RAC1	2
GO:00230	signal tra	2/137	20/18670	0.009314	0.018705	0.007448	HNF4A/PAI	2
GO:00309(	notochord	2/137	20/18670	0.009314	0.018705	0.007448	EPHA2/GLI	2
GO:00324(	response t	2/137	20/18670	0.009314	0.018705	0.007448	MAPK14/RI	2
GO:00331(	response t	2/137	20/18670	0.009314	0.018705	0.007448	JAK2/DAPI	2
GO:00343(	positive r	2/137	20/18670	0.009314	0.018705	0.007448	PPARG/MAI	2
GO:00435(	tongue dev	2/137	20/18670	0.009314	0.018705	0.007448	KIT/EGFR	2
GO:00456(	regulation	2/137	20/18670	0.009314	0.018705	0.007448	CDK6/JUN	2
GO:00517(	regulation	2/137	20/18670	0.009314	0.018705	0.007448	TNF/SMO	2
GO:00600(	Sertoli ce	2/137	20/18670	0.009314	0.018705	0.007448	ICAM1/NTI	2
GO:00977(	connective	2/137	20/18670	0.009314	0.018705	0.007448	HIF1A/TGI	2
GO:19029(	positive r	2/137	20/18670	0.009314	0.018705	0.007448	TNF/CASP3	2
GO:00708(	divalent m	9/137	483/1867(	0.009356	0.018778	0.007477	VDR/PDGFI	9
GO:00485(	camera-typ	4/137	112/1867(	0.00936	0.018778	0.007477	VEGFA/HII	4
GO:00461(	alcohol bi	5/137	175/1867(	0.009456	0.018963	0.00755	VDR/FGF2/	5
GO:00326(	interferon	4/137	113/1867(	0.009649	0.01933	0.007697	HRAS/TNF/	4
GO:00486	negative r	4/137	113/1867(	0.009649	0.01933	0.007697	PTK2/FGFI	4
GO:00022(	CD4-positi	3/137	60/18670	0.009752	0.019509	0.007768	MTOR/STAT	3
GO:00325	negative r	3/137	60/18670	0.009752	0.019509	0.007768	GSK3B/LGA	3
GO:01201(	tight junc	3/137	60/18670	0.009752	0.019509	0.007768	TGFBR1/EI	3
GO:00067(	coenzyme m	8/137	403/1867(	0.009841	0.019677	0.007835	PTGS2/HII	8
GO:00026(	regulation	4/137	114/1867(	0.009944	0.019865	0.00791	MAPK14/MA	4
GO:00071	meiosis I	4/137	114/1867(	0.009944	0.019865	0.00791	AURKA/TOI	4
GO:00435	protein ac	6/137	248/1867(	0.010035	0.020036	0.007978	MAPK3/GSI	6
GO:00725	divalent i	9/137	489/1867(	0.01009	0.020136	0.008017	VDR/PDGFI	9
GO:00019	heart loop	3/137	61/18670	0.010202	0.020158	0.008026	HIF1A/SM(	3
GO:00022(	alpha-beta	3/137	61/18670	0.010202	0.020158	0.008026	MTOR/STAT	3
GO:00022(	alpha-beta	3/137	61/18670	0.010202	0.020158	0.008026	MTOR/STAT	3
GO:00108(	regulation	3/137	61/18670	0.010202	0.020158	0.008026	TNF/CASP8	3
GO:00305	androgen r	3/137	61/18670	0.010202	0.020158	0.008026	AR/EP300/	3
GO:00341	heterotypi	3/137	61/18670	0.010202	0.020158	0.008026	TNF/ITGB1	3
GO:00424(	mechanorec	3/137	61/18670	0.010202	0.020158	0.008026	FGFR1/NTI	3
GO:00451	cellular e	3/137	61/18670	0.010202	0.020158	0.008026	TNF/ICAM1	3
GO:00513	meiotic ce	6/137	249/1867(	0.010222	0.020158	0.008026	AURKA/TOI	6
GO:00060	aminoglyca	4/137	115/1867(	0.010245	0.020158	0.008026	PDGFRB/NI	4
GO:00060(	glycolytic	4/137	115/1867(	0.010245	0.020158	0.008026	HIF1A/STA	4
GO:00326	interleuki	4/137	115/1867(	0.010245	0.020158	0.008026	CASP8/JAI	4
GO:00032(	atrial sep	2/137	21/18670	0.010246	0.020158	0.008026	MDM2/SMO	2
GO:00108	negative r	2/137	21/18670	0.010246	0.020158	0.008026	TNF/IL1B	2
GO:00108(	positive r	2/137	21/18670	0.010246	0.020158	0.008026	TNF/IL1B	2
GO:00170(	antibiotic	2/137	21/18670	0.010246	0.020158	0.008026	STAT3/CYI	2
GO:00216(	cerebellar	2/137	21/18670	0.010246	0.020158	0.008026	MAP2K1/PT	2
GO:00307	ovulation	2/137	21/18670	0.010246	0.020158	0.008026	PGR/PTGS2	2
GO:00312(	retinal ga	2/137	21/18670	0.010246	0.020158	0.008026	VEGFA/EPI	2
GO:00326(	negative r	2/137	21/18670	0.010246	0.020158	0.008026	EPHA2/GST	2
GO:00328(	negative r	2/137	21/18670	0.010246	0.020158	0.008026	AKT1/AKT2	2

GO:00467	muscle cel	2/137	21/18670	0.010246	0.020158	0.008026	HIF1A/TGI	2
GO:00509	sensory pe	2/137	21/18670	0.010246	0.020158	0.008026	NTRK1/CX(	2
GO:00514	response t	2/137	21/18670	0.010246	0.020158	0.008026	CCND1/NTI	2
GO:00607	labyrinthi	2/137	21/18670	0.010246	0.020158	0.008026	FGFR2/GRI	2
GO:00714	cellular r	2/137	21/18670	0.010246	0.020158	0.008026	SRC/PTGS2	2
GO:00721	cell proli	2/137	21/18670	0.010246	0.020158	0.008026	STAT1/PD(	2
GO:00902	positive r	2/137	21/18670	0.010246	0.020158	0.008026	PDGFRB/L(	2
GO:20002	regulatior	2/137	21/18670	0.010246	0.020158	0.008026	PIK3CA/SI	2
GO:20008	regulatior	2/137	21/18670	0.010246	0.020158	0.008026	EPHA2/TNI	2
GO:00066	protein e	5/137	179/1867(	0.01036	0.020373	0.008112	PTPN11/GS	5
GO:00508	synapse or	8/137	408/1867(	0.010547	0.020732	0.008255	MAPK14/I(	8
GO:00067	ATP genera	4/137	116/1867(	0.010552	0.020732	0.008255	HIF1A/STA	4
GO:00706	regulatior	5/137	180/1867(	0.010595	0.020806	0.008284	SRC/MMP14	5
GO:00725	cellular c	9/137	493/1867(	0.010602	0.020806	0.008284	ESR1/VDR/	9
GO:00450	glyceroli	6/137	251/1867(	0.010605	0.020806	0.008284	PLA2G2A/I	6
GO:00025	somatic di	3/137	62/18670	0.010665	0.020865	0.008308	IL2/ATM/T	3
GO:00027	cytoplasmi	3/137	62/18670	0.010665	0.020865	0.008308	CASP8/XIA	3
GO:00164	somatic ce	3/137	62/18670	0.010665	0.020865	0.008308	IL2/ATM/T	3
GO:00316	killing of	3/137	62/18670	0.010665	0.020865	0.008308	NOS2/GAPI	3
GO:00328	glomerulus	3/137	62/18670	0.010665	0.020865	0.008308	RET/PDGFI	3
GO:00443	disruptior	3/137	62/18670	0.010665	0.020865	0.008308	NOS2/GAPI	3
GO:19003	regulatior	4/137	117/1867(	0.010865	0.021246	0.00846	NOS2/HIF1	4
GO:00512	maintenanc	7/137	330/1867(	0.011029	0.021558	0.008584	PPARG/FGI	7
GO:00300	actin fila	5/137	182/1867(	0.011075	0.021627	0.008611	MTOR/ICAM	5
GO:19033	regulatior	5/137	182/1867(	0.011075	0.021627	0.008611	SRC/MMP14	5
GO:00026	regulatior	3/137	63/18670	0.011139	0.021731	0.008653	TNF/IL2/T	3
GO:00308	regulatior	4/137	118/1867(	0.011183	0.021731	0.008653	NOS2/HIF1	4
GO:00017	organ indu	2/137	22/18670	0.011216	0.021731	0.008653	AR/FGFR1	2
GO:00350	negative r	2/137	22/18670	0.011216	0.021731	0.008653	MET/ITGB1	2
GO:00434	regulatior	2/137	22/18670	0.011216	0.021731	0.008653	BCL2/LGAI	2
GO:00456	positive r	2/137	22/18670	0.011216	0.021731	0.008653	MAPK14/I(	2
GO:00457	positive r	2/137	22/18670	0.011216	0.021731	0.008653	PTGS2/IL1	2
GO:00459	positive r	2/137	22/18670	0.011216	0.021731	0.008653	MTOR/ERBI	2
GO:00466	positive r	2/137	22/18670	0.011216	0.021731	0.008653	PTPN11/SI	2
GO:00483	immunoglol	2/137	22/18670	0.011216	0.021731	0.008653	TNF/IL2	2
GO:00511	negative r	2/137	22/18670	0.011216	0.021731	0.008653	MMP3/STAT	2
GO:00600	uterus dev	2/137	22/18670	0.011216	0.021731	0.008653	ESR1/SRC	2
GO:00605	intestinal	2/137	22/18670	0.011216	0.021731	0.008653	HIF1A/TYM	2
GO:00714	cellular r	2/137	22/18670	0.011216	0.021731	0.008653	PPARG/IT(	2
GO:00903	positive r	2/137	22/18670	0.011216	0.021731	0.008653	VEGFA/TGI	2
GO:19030	negative r	2/137	22/18670	0.011216	0.021731	0.008653	BCL2L1/T(	2
GO:20007	negative r	2/137	22/18670	0.011216	0.021731	0.008653	TERT/CDK6	2
GO:00431	positive r	5/137	183/1867(	0.011321	0.021912	0.008725	TNF/AKT1/	5
GO:00611	regulatior	5/137	183/1867(	0.011321	0.021912	0.008725	GSK3B/AUI	5
GO:00464	glyceroli	8/137	414/1867(	0.011444	0.022141	0.008816	PTPN11/PI	8
GO:00428	pyruvate b	4/137	119/1867(	0.011508	0.022234	0.008853	HIF1A/STA	4
GO:00511	regulatior	4/137	119/1867(	0.011508	0.022234	0.008853	MMP3/HIF1	4

GO:00619(	meiosis I	4/137	119/1867(	0.011508	0.022234	0.008853	AURKA/TOI	4
GO:00636(	transcript	3/137	64/18670	0.011625	0.022439	0.008934	MAPK3/MT(	3
GO:00468:	lipid phos	3/137	64/18670	0.011625	0.022439	0.008934	PIK3CA/PI	3
GO:00269	negative r	4/137	120/1867(	0.011839	0.022841	0.009095	LGALS3/TN	4
GO:00353(	negative r	3/137	65/18670	0.012123	0.023357	0.0093	GSK3B/LGA	3
GO:00457:	positive r	3/137	65/18670	0.012123	0.023357	0.0093	EGFR/PARI	3
GO:00603(	pathway-re	3/137	65/18670	0.012123	0.023357	0.0093	TGFBR1/T(	3
GO:00635	regulation	2/137	23/18670	0.012226	0.023488	0.009352	AR/MTOR	2
GO:00510	regulation	2/137	23/18670	0.012226	0.023488	0.009352	TNF/IL1B	2
GO:00511	positive r	2/137	23/18670	0.012226	0.023488	0.009352	KIT/TGFBI	2
GO:00600	angiogenes	2/137	23/18670	0.012226	0.023488	0.009352	PIK3CB/CX	2
GO:00713(	cellular r	2/137	23/18670	0.012226	0.023488	0.009352	VDR/SFRPI	2
GO:19022	negative r	2/137	23/18670	0.012226	0.023488	0.009352	BCL2/MDM2	2
GO:00431(	proteasome	8/137	419/1867(	0.012233	0.023488	0.009352	GSK3B/CDI	8
GO:00902	regulation	6/137	259/1867(	0.012236	0.023488	0.009352	KIT/MTOR/	6
GO:00062(	regulation	4/137	122/1867(	0.012518	0.024019	0.009564	CHEK1/EGI	4
GO:19030	meiotic ce	5/137	188/1867(	0.012605	0.024175	0.009626	AURKA/TOI	5
GO:00468(	negative r	3/137	66/18670	0.012633	0.024206	0.009638	PTPN11/II	3
GO:19052(	regulation	3/137	66/18670	0.012633	0.024206	0.009638	MTOR/EGFI	3
GO:00008	sister chr	5/137	189/1867(	0.012874	0.024655	0.009817	TOP2A/PLI	5
GO:00031	embryonic	3/137	67/18670	0.013156	0.025138	0.010009	HIF1A/SM(	3
GO:00463(	monosaccha	3/137	67/18670	0.013156	0.025138	0.010009	GAPDH/PKN	3
GO:00486(	negative r	3/137	67/18670	0.013156	0.025138	0.010009	PPARG/IGI	3
GO:00487	muscle fil	3/137	67/18670	0.013156	0.025138	0.010009	BCL2/VEGI	3
GO:00613	determinat	3/137	67/18670	0.013156	0.025138	0.010009	HIF1A/SM(	3
GO:00031(	atrioventr	2/137	24/18670	0.013274	0.025272	0.010062	MDM2/TGFI	2
GO:00032	endocardia	2/137	24/18670	0.013274	0.025272	0.010062	TGFBR1/T(	2
GO:00102(	response t	2/137	24/18670	0.013274	0.025272	0.010062	PTGS2/CDI	2
GO:00443	fibroblast	2/137	24/18670	0.013274	0.025272	0.010062	PIK3CA/SI	2
GO:00509	positive r	2/137	24/18670	0.013274	0.025272	0.010062	AKT2/IL1I	2
GO:00603	trabecula	2/137	24/18670	0.013274	0.025272	0.010062	MMP2/SFRI	2
GO:19000	positive r	2/137	24/18670	0.013274	0.025272	0.010062	PTPN11/SI	2
GO:19043	negative r	2/137	24/18670	0.013274	0.025272	0.010062	BCL2L1/T(	2
GO:00723:	monocarbox	7/137	343/1867(	0.013409	0.025516	0.01016	PTGS2/HII	7
GO:00022	T cell di	3/137	68/18670	0.013691	0.026005	0.010355	MTOR/STA	3
GO:00162:	positive r	3/137	68/18670	0.013691	0.026005	0.010355	MAPK3/KDI	3
GO:00313	positive r	3/137	68/18670	0.013691	0.026005	0.010355	NOS2/GAPI	3
GO:00519(	positive r	3/137	68/18670	0.013691	0.026005	0.010355	NTRK1/NTI	3
GO:00487(	embryonic	4/137	126/1867(	0.013951	0.026476	0.010542	TGFBR1/MN	4
GO:00901(	negative r	4/137	126/1867(	0.013951	0.026476	0.010542	TGFBR1/T(	4
GO:00550(	cardiac mu	3/137	69/18670	0.014238	0.026995	0.010749	TGFBR1/F(	3
GO:20002	positive r	3/137	69/18670	0.014238	0.026995	0.010749	AR/SRC/AI	3
GO:00511(	nuclear ex	5/137	194/1867(	0.014273	0.02705	0.010771	PTPN11/GS	5
GO:00025(	tolerance	2/137	25/18670	0.01436	0.027091	0.010787	TGFB1/TGI	2
GO:00027	negative r	2/137	25/18670	0.01436	0.027091	0.010787	TNF/TGFBI	2
GO:00092	mRNA trans	2/137	25/18670	0.01436	0.027091	0.010787	HIF1A/STA	2
GO:00194:	removal of	2/137	25/18670	0.01436	0.027091	0.010787	TNF/NQO1	2

GO:00324(	positive r	2/137	25/18670	0.01436	0.027091	0.010787	MMP3/MMP1	2
GO:00453:	phospholip	2/137	25/18670	0.01436	0.027091	0.010787	ABCC1/ABC	2
GO:00456(	negative r	2/137	25/18670	0.01436	0.027091	0.010787	TNF/TGFB1	2
GO:00458:	positive r	2/137	25/18670	0.01436	0.027091	0.010787	IL2/TGFB1	2
GO:00509(	leukocyte	2/137	25/18670	0.01436	0.027091	0.010787	TNF/ITGB1	2
GO:00722(	cell diffe	2/137	25/18670	0.01436	0.027091	0.010787	STAT1/SMO	2
GO:00017!	somitogene	3/137	70/18670	0.014797	0.027878	0.0111	ATM/EP300	3
GO:00330'	T cell dif	3/137	70/18670	0.014797	0.027878	0.0111	BCL2/CDK6	3
GO:00507(	positive r	3/137	70/18670	0.014797	0.027878	0.0111	PPARG/TNF	3
GO:00066!	steroid bi	5/137	196/1867(	0.014861	0.027986	0.011143	CYP17A1/V	5
GO:00022:	pattern re	5/137	197/1867(	0.01516	0.028537	0.011363	ESR1/CTSB	5
GO:00015!	oocyte mat	2/137	26/18670	0.015482	0.028925	0.011517	AURKA/CCN	2
GO:00031'	atrioventr	2/137	26/18670	0.015482	0.028925	0.011517	MDM2/TGFB	2
GO:00107!	regulation	2/137	26/18670	0.015482	0.028925	0.011517	MAPK14/MA	2
GO:00303:	melanocyte	2/137	26/18670	0.015482	0.028925	0.011517	KIT/BCL2	2
GO:00319!	positive r	2/137	26/18670	0.015482	0.028925	0.011517	SRC/VEGFA	2
GO:00329(	positive r	2/137	26/18670	0.015482	0.028925	0.011517	PDGFRB/TG	2
GO:00341:	regulation	2/137	26/18670	0.015482	0.028925	0.011517	TNF/IL1B	2
GO:00342(	lipid tran	2/137	26/18670	0.015482	0.028925	0.011517	ABCC1/ABC	2
GO:00435(	regulation	2/137	26/18670	0.015482	0.028925	0.011517	AR/IGFBP3	2
GO:00606(	embryonic	2/137	26/18670	0.015482	0.028925	0.011517	FGFR2/GRH	2
GO:00607(	cell diffe	2/137	26/18670	0.015482	0.028925	0.011517	AKT1/CASP	2
GO:00725:	T-helper 1	2/137	26/18670	0.015482	0.028925	0.011517	STAT3/IL2	2
GO:00970(	response t	2/137	26/18670	0.015482	0.028925	0.011517	CTSB/KIT	2
GO:19035'	negative r	2/137	26/18670	0.015482	0.028925	0.011517	STAT3/PAH	2
GO:19047!	positive r	2/137	26/18670	0.015482	0.028925	0.011517	TERT/MDM2	2
GO:00069!	nucleus or	4/137	130/1867(	0.015484	0.028925	0.011517	CDK1/PRKC	4
GO:00506'	positive r	4/137	130/1867(	0.015484	0.028925	0.011517	BCL2/IL2/	4
GO:00987	cell-cell	6/137	273/1867(	0.015503	0.028948	0.011526	MAPK14/RH	6
GO:00160!	carbohydra	5/137	199/1867(	0.015772	0.029436	0.011721	HIF1A/STA	5
GO:00329	positive r	4/137	131/1867(	0.015883	0.029617	0.011793	BCL2/IL2/	4
GO:19028!	microtubul	4/137	131/1867(	0.015883	0.029617	0.011793	AURKA/PLK	4
GO:00066:	fatty acid	3/137	72/18670	0.015952	0.02972	0.011833	MTOR/AKT1	3
GO:00610:	regulation	3/137	72/18670	0.015952	0.02972	0.011833	TGFBR1/RH	3
GO:00160!	organic ac	6/137	275/1867(	0.016015	0.029797	0.011864	NOS2/MTOR	6
GO:00432'	positive r	6/137	275/1867(	0.016015	0.029797	0.011864	ABCB1/PDC	6
GO:00463!	carboxylic	6/137	275/1867(	0.016015	0.029797	0.011864	NOS2/MTOR	6
GO:00424'	odontogene	4/137	132/1867(	0.016288	0.030293	0.012062	SRC/SMO/F	4
GO:00514(	regulation	7/137	357/1867(	0.016362	0.030415	0.01211	ESR1/BCL2	7
GO:00335!	multicellu	3/137	73/18670	0.016548	0.030676	0.012214	RET/BCL2/	3
GO:00603!	endochondr	3/137	73/18670	0.016548	0.030676	0.012214	MMP14/FGF	3
GO:00099:	epidermal	7/137	358/1867(	0.016589	0.030676	0.012214	VDR/MAP2K	7
GO:00031	outflow tr	2/137	27/18670	0.016641	0.030676	0.012214	FGFR2/TGF	2
GO:00067:	NADH regen	2/137	27/18670	0.016641	0.030676	0.012214	GAPDH/PKM	2
GO:00107:	positive r	2/137	27/18670	0.016641	0.030676	0.012214	PDGFRB/TG	2
GO:00439:	modulation	2/137	27/18670	0.016641	0.030676	0.012214	JUN/EP300	2
GO:00456'	negative r	2/137	27/18670	0.016641	0.030676	0.012214	PIK3R1/SH	2

GO:00487	animal org	2/137	27/18670	0.016641	0.030676	0.012214	RET/FGFR3	2
GO:00524	modulatior	2/137	27/18670	0.016641	0.030676	0.012214	JUN/EP300	2
GO:00602	regulatior	2/137	27/18670	0.016641	0.030676	0.012214	MTOR/STAT	2
GO:00603	innervatic	2/137	27/18670	0.016641	0.030676	0.012214	RET/NTRK1	2
GO:00616	canonical	2/137	27/18670	0.016641	0.030676	0.012214	GAPDH/PKM	2
GO:00617	glucose ca	2/137	27/18670	0.016641	0.030676	0.012214	GAPDH/PKM	2
GO:00714	cellular r	2/137	27/18670	0.016641	0.030676	0.012214	TNF/NQO1	2
GO:00714	cellular r	2/137	27/18670	0.016641	0.030676	0.012214	TNF/NQO1	2
GO:19043	negative r	2/137	27/18670	0.016641	0.030676	0.012214	SRC/PARP1	2
GO:19055	negative r	2/137	27/18670	0.016641	0.030676	0.012214	PPARG/FLT	2
GO:20001	positive r	2/137	27/18670	0.016641	0.030676	0.012214	ESR1/JUN	2
GO:00027	positive r	4/137	133/18670	0.0167	0.030771	0.012252	TNF/IL2/I	4
GO:00072	Rho protei	5/137	203/18670	0.017044	0.031389	0.012498	MET/PDGFR	5
GO:00061	nucleoside	4/137	134/18670	0.017119	0.031514	0.012548	HIF1A/STA	4
GO:00433	CD4-positi	3/137	74/18670	0.017157	0.031556	0.012565	MTOR/STAT	3
GO:00996	regulatior	3/137	74/18670	0.017157	0.031556	0.012565	SRC/DAPK1	3
GO:00091	purine rib	6/137	280/18670	0.017345	0.031874	0.012691	HIF1A/STA	6
GO:00600	heart cont	6/137	280/18670	0.017345	0.031874	0.012691	MTOR/PIK3	6
GO:00300	establishm	4/137	135/18670	0.017544	0.032226	0.012831	GSK3B/HSP	4
GO:00022	somatic di	3/137	75/18670	0.017778	0.032505	0.012943	IL2/ATM/T	3
GO:00015	prostaglar	2/137	28/18670	0.017836	0.032505	0.012943	PTGS2/IL1	2
GO:00017	myeloid de	2/137	28/18670	0.017836	0.032505	0.012943	TGFB1/TGF	2
GO:00019	endochondr	2/137	28/18670	0.017836	0.032505	0.012943	MMP14/FGF	2
GO:00080	motor neur	2/137	28/18670	0.017836	0.032505	0.012943	ERBB2/RAC	2
GO:00082	androgen m	2/137	28/18670	0.017836	0.032505	0.012943	CYP17A1/H	2
GO:00218	cell proli	2/137	28/18670	0.017836	0.032505	0.012943	FGFR1/FGF	2
GO:00346	response t	2/137	28/18670	0.017836	0.032505	0.012943	CCNA2/ICA	2
GO:00360	replacemer	2/137	28/18670	0.017836	0.032505	0.012943	MMP14/FGF	2
GO:00464	prostanoic	2/137	28/18670	0.017836	0.032505	0.012943	PTGS2/IL1	2
GO:00486	regulatior	2/137	28/18670	0.017836	0.032505	0.012943	MAP2K1/NT	2
GO:00616	glycolytic	2/137	28/18670	0.017836	0.032505	0.012943	GAPDH/PKM	2
GO:00616	glycolytic	2/137	28/18670	0.017836	0.032505	0.012943	GAPDH/PKM	2
GO:00903	negative r	2/137	28/18670	0.017836	0.032505	0.012943	TERT/CDK6	2
GO:19005	negative r	2/137	28/18670	0.017836	0.032505	0.012943	STAT3/PAR	2
GO:19020	regulatior	2/137	28/18670	0.017836	0.032505	0.012943	TNF/CASP3	2
GO:19029	positive r	2/137	28/18670	0.017836	0.032505	0.012943	TNF/IL1B	2
GO:19050	regulatior	2/137	28/18670	0.017836	0.032505	0.012943	DNMT1/KIT	2
GO:00469	nucleotide	4/137	136/18670	0.017976	0.032745	0.013038	HIF1A/STA	4
GO:00324	regulatior	6/137	283/18670	0.018178	0.0331	0.013179	PPARG/ABC	6
GO:00196	NAD metabo	3/137	76/18670	0.018411	0.033494	0.013336	PTGS2/GAP	3
GO:00488	artery mor	3/137	76/18670	0.018411	0.033494	0.013336	TGFBR1/PI	3
GO:00322	methylatic	7/137	366/18670	0.018488	0.03362	0.013387	DNMT1/PIH	7
GO:00165	histone de	3/137	77/18670	0.019057	0.034373	0.013686	VEGFA/MAP	3
GO:00350	embryonic	3/137	77/18670	0.019057	0.034373	0.013686	HIF1A/SMC	3
GO:00518	modificati	3/137	77/18670	0.019057	0.034373	0.013686	GAPDH/JUN	3
GO:20007	regulatior	3/137	77/18670	0.019057	0.034373	0.013686	CHEK1/PAR	3
GO:00003	response t	2/137	29/18670	0.019067	0.034373	0.013686	TNF/NQO1	2

GO:00305	positive r	2/137	29/18670	0.019067	0.034373	0.013686	EP300/MEN	2
GO:00311	positive r	2/137	29/18670	0.019067	0.034373	0.013686	MET/RAC1	2
GO:00330	regulation	2/137	29/18670	0.019067	0.034373	0.013686	BCL2/PIK3	2
GO:00350	positive r	2/137	29/18670	0.019067	0.034373	0.013686	PDGFRB/RA	2
GO:00440	regulation	2/137	29/18670	0.019067	0.034373	0.013686	PIK3CA/PA	2
GO:00458	negative r	2/137	29/18670	0.019067	0.034373	0.013686	IL2/JAK2	2
GO:00459	negative r	2/137	29/18670	0.019067	0.034373	0.013686	STAT3/PAF	2
GO:00604	epithelial	2/137	29/18670	0.019067	0.034373	0.013686	TNF/FGFR2	2
GO:00607	regulation	2/137	29/18670	0.019067	0.034373	0.013686	EP300/SFF	2
GO:00702	negative r	2/137	29/18670	0.019067	0.034373	0.013686	HIF1A/IL2	2
GO:00705	protein ki	2/137	29/18670	0.019067	0.034373	0.013686	VEGFA/FLT	2
GO:00725	T-helper 1	2/137	29/18670	0.019067	0.034373	0.013686	STAT3/IL2	2
GO:19000	regulation	2/137	29/18670	0.019067	0.034373	0.013686	HRAS/ICAM	2
GO:19038	positive r	2/137	29/18670	0.019067	0.034373	0.013686	EP300/MEN	2
GO:20007	positive r	2/137	29/18670	0.019067	0.034373	0.013686	MTOR/TGFF	2
GO:00081	actin poly	5/137	209/18670	0.019076	0.034375	0.013687	MTOR/ICAM	5
GO:00028	regulation	4/137	139/18670	0.01931	0.034751	0.013837	MAPK3/STA	4
GO:00313	negative r	4/137	139/18670	0.01931	0.034751	0.013837	SRC/GSK3F	4
GO:00706	positive r	4/137	139/18670	0.01931	0.034751	0.013837	BCL2/IL2/	4
GO:00434	negative r	3/137	78/18670	0.019715	0.035465	0.014121	IL1B/GSTF	3
GO:00026	regulation	8/137	458/18670	0.019827	0.03565	0.014195	MAPK14/MA	8
GO:00507	negative r	4/137	141/18670	0.020232	0.036364	0.014479	PTPN11/TN	4
GO:00003	response t	2/137	30/18670	0.020332	0.03637	0.014482	TNF/NQO1	2
GO:00381	interleuki	2/137	30/18670	0.020332	0.03637	0.014482	STAT3/JAK	2
GO:00427	long-chain	2/137	30/18670	0.020332	0.03637	0.014482	PTGS2/CYF	2
GO:00448	mitotic G2	2/137	30/18670	0.020332	0.03637	0.014482	CDK1/ATM	2
GO:00459	positive r	2/137	30/18670	0.020332	0.03637	0.014482	TNF/IL1B	2
GO:00482	response t	2/137	30/18670	0.020332	0.03637	0.014482	RET/NTRK1	2
GO:00610	myeloid le	2/137	30/18670	0.020332	0.03637	0.014482	KIT/TGFB1	2
GO:00701	negative r	2/137	30/18670	0.020332	0.03637	0.014482	HIF1A/TGF	2
GO:00902	positive r	2/137	30/18670	0.020332	0.03637	0.014482	MMP9/BAD	2
GO:00990	postsynaps	2/137	30/18670	0.020332	0.03637	0.014482	NTRK3/EPH	2
GO:19054	negative r	2/137	30/18670	0.020332	0.03637	0.014482	BCL2L1/TC	2
GO:00064	regulation	3/137	79/18670	0.020386	0.03642	0.014502	MTOR/TNF/	3
GO:00216	nerve deve	3/137	79/18670	0.020386	0.03642	0.014502	RET/NTRK1	3
GO:00435	negative r	3/137	79/18670	0.020386	0.03642	0.014502	PPARG/FGF	3
GO:00063	DNA recomb	6/137	292/18670	0.020843	0.037222	0.014821	CHEK1/PAF	6
GO:00092	ribonucleo	6/137	293/18670	0.021155	0.037729	0.015023	HIF1A/STA	6
GO:00352	regulation	4/137	143/18670	0.021182	0.037729	0.015023	PTGS2/EGF	4
GO:00508	regulation	4/137	143/18670	0.021182	0.037729	0.015023	PTGS2/EGF	4
GO:00977	regulation	4/137	143/18670	0.021182	0.037729	0.015023	PTGS2/EGF	4
GO:01061	cold-induc	4/137	143/18670	0.021182	0.037729	0.015023	IGF1R/VEG	4
GO:01201	regulation	4/137	143/18670	0.021182	0.037729	0.015023	IGF1R/VEG	4
GO:00080	cell recog	5/137	215/18670	0.021263	0.037858	0.015074	LGALS3/CA	5
GO:00020	regulation	2/137	31/18670	0.021631	0.038366	0.015276	CDK1/CCNF	2
GO:00331	response t	2/137	31/18670	0.021631	0.038366	0.015276	PTGS2/IL1	2
GO:00400	positive r	2/137	31/18670	0.021631	0.038366	0.015276	BCL2/SMO	2

GO:00457	positive r	2/137	31/18670	0.021631	0.038366	0.015276	MMP9/AKT	2
GO:00508	negative r	2/137	31/18670	0.021631	0.038366	0.015276	GSK3B/MT	2
GO:00715	protein l	2/137	31/18670	0.021631	0.038366	0.015276	GSK3B/AU	2
GO:19022	negative r	2/137	31/18670	0.021631	0.038366	0.015276	BCL2/BCL	2
GO:19022	regulation	2/137	31/18670	0.021631	0.038366	0.015276	BCL2/MDM	2
GO:20007	positive r	2/137	31/18670	0.021631	0.038366	0.015276	PARP1/MG	2
GO:00351	regulation	4/137	144/1867	0.021666	0.038413	0.015295	PTGS2/EG	4
GO:00305	negative r	3/137	81/18670	0.021764	0.038554	0.015351	TGFBR1/T	3
GO:19035	regulation	3/137	81/18670	0.021764	0.038554	0.015351	HRAS/ERB	3
GO:00028	regulation	4/137	145/1867	0.022158	0.039218	0.015615	TNF/IL2/	4
GO:00076	sensory p	4/137	145/1867	0.022158	0.039218	0.015615	FGFR1/KI	4
GO:00326	interleuki	3/137	82/18670	0.022473	0.039724	0.015817	NOS2/TNF/	3
GO:00517	interactio	3/137	82/18670	0.022473	0.039724	0.015817	GAPDH/JU	3
GO:00604	muscle tis	3/137	82/18670	0.022473	0.039724	0.015817	TGFBR1/F	3
GO:00015	branching	2/137	32/18670	0.022963	0.040403	0.016087	VEGFA/TG	2
GO:00063	regulation	2/137	32/18670	0.022963	0.040403	0.016087	MTOR/ERB	2
GO:00455	regulation	2/137	32/18670	0.022963	0.040403	0.016087	IL2/TGFB	2
GO:00459	positive r	2/137	32/18670	0.022963	0.040403	0.016087	KIT/PTGS	2
GO:00487	skeletal m	2/137	32/18670	0.022963	0.040403	0.016087	BCL2/SMO	2
GO:00603	cardiac ep	2/137	32/18670	0.022963	0.040403	0.016087	TGFBR1/T	2
GO:00705	regulation	2/137	32/18670	0.022963	0.040403	0.016087	MAP2K1/N	2
GO:19007	positive r	2/137	32/18670	0.022963	0.040403	0.016087	VEGFA/IL	2
GO:19019	regulation	2/137	32/18670	0.022963	0.040403	0.016087	CCNB1/CH	2
GO:19055	protein l	2/137	32/18670	0.022963	0.040403	0.016087	GSK3B/AU	2
GO:20007	positive r	2/137	32/18670	0.022963	0.040403	0.016087	TNF/IL1B	2
GO:19038	negative r	3/137	83/18670	0.023193	0.040791	0.016242	TGFBR1/T	3
GO:00463	ribose ph	6/137	300/1867	0.023425	0.041182	0.016397	HIF1A/ST	6
GO:00027	negative r	4/137	148/1867	0.023673	0.0416	0.016564	PTPN11/T	4
GO:00508	endocrine	3/137	84/18670	0.023926	0.042027	0.016734	PTPN11/F	3
GO:00027	regulation	4/137	149/1867	0.024192	0.042476	0.016913	TNF/IL2/	4
GO:00311	positive r	2/137	33/18670	0.024329	0.042555	0.016944	MET/RAC1	2
GO:00330	myeloid c	2/137	33/18670	0.024329	0.042555	0.016944	BCL2/PIK	2
GO:00360	positive r	2/137	33/18670	0.024329	0.042555	0.016944	VEGFA/HI	2
GO:00427	eating be	2/137	33/18670	0.024329	0.042555	0.016944	MTOR/STA	2
GO:00451	regulation	2/137	33/18670	0.024329	0.042555	0.016944	IL2/TGFB	2
GO:00702	regulation	2/137	33/18670	0.024329	0.042555	0.016944	HIF1A/LG	2
GO:19011	positive r	2/137	33/18670	0.024329	0.042555	0.016944	MMP9/AKT	2
GO:19017	negative r	2/137	33/18670	0.024329	0.042555	0.016944	BCL2/MDM	2
GO:20010	negative r	2/137	33/18670	0.024329	0.042555	0.016944	PTPN11/M	2
GO:00104	proteasom	8/137	477/1867	0.024545	0.042916	0.017088	GSK3B/CD	8
GO:00097	axis speci	3/137	85/18670	0.024672	0.0431	0.017161	AURKA/SM	3
GO:00507	positive r	3/137	85/18670	0.024672	0.0431	0.017161	MAP2K1/V	3
GO:00000	mitotic si	4/137	151/1867	0.02525	0.044093	0.017556	PLK1/CCN	4
GO:00327	positive r	3/137	86/18670	0.02543	0.044369	0.017666	PTPN11/J	3
GO:00602	long-term	3/137	86/18670	0.02543	0.044369	0.017666	GSK3B/MA	3
GO:00033	epithelial	2/137	34/18670	0.025727	0.044701	0.017799	AR/MET	2
GO:00311	developme	2/137	34/18670	0.025727	0.044701	0.017799	AR/FGFR1	2

GO:00326(	regulatior	2/137	34/18670	0.025727	0.044701	0.017799	IL2/TGFB1	2
GO:00342(	amyloid-be	2/137	34/18670	0.025727	0.044701	0.017799	TNF/CASP3	2
GO:00435	regulatior	2/137	34/18670	0.025727	0.044701	0.017799	MDM2/ATM	2
GO:00450(	regulatory	2/137	34/18670	0.025727	0.044701	0.017799	IL2/TGFB1	2
GO:00485(	embryonic	2/137	34/18670	0.025727	0.044701	0.017799	TNF/FGFR2	2
GO:00718(	cellular r	2/137	34/18670	0.025727	0.044701	0.017799	MAPK3/MAI	2
GO:00718	cellular r	2/137	34/18670	0.025727	0.044701	0.017799	MAPK3/MAI	2
GO:00970(	energy hon	2/137	34/18670	0.025727	0.044701	0.017799	PIK3CA/ST	2
GO:00326	regulatior	4/137	152/1867(	0.02579	0.044791	0.017835	PTPN11/ST	4
GO:00347(	regulatior	8/137	483/1867(	0.026187	0.045463	0.018102	ABCB1/MMI	8
GO:00458(	positive r	4/137	153/1867(	0.026336	0.045702	0.018197	PPARG/VE(	4
GO:00434	macromolec	6/137	309/1867(	0.026578	0.046103	0.018357	DNMT1/PII	6
GO:00060	pyruvate m	4/137	154/1867(	0.026889	0.046566	0.018541	HIF1A/STA	4
GO:00107	positive r	4/137	154/1867(	0.026889	0.046566	0.018541	MAP2K1/VI	4
GO:00550(	monovalent	4/137	154/1867(	0.026889	0.046566	0.018541	MAPK3/CA7	4
GO:19908	adaptive t	4/137	154/1867(	0.026889	0.046566	0.018541	IGF1R/VE(	4
GO:00486	muscle org	3/137	88/18670	0.026983	0.04665	0.018575	TGFBR1/F(	3
GO:01060	neuron pro	3/137	88/18670	0.026983	0.04665	0.018575	IGF1R/GSI	3
GO:19033	negative r	3/137	88/18670	0.026983	0.04665	0.018575	MTOR/AKT1	3
GO:19035	positive r	3/137	88/18670	0.026983	0.04665	0.018575	PTPN11/JA	3
GO:00027(	negative r	2/137	35/18670	0.027157	0.046836	0.018649	TNF/TGFB1	2
GO:00032	cardiac at	2/137	35/18670	0.027157	0.046836	0.018649	MDM2/SMO	2
GO:00149(	myotube ce	2/137	35/18670	0.027157	0.046836	0.018649	BCL2/SMO	2
GO:00302(	apoptotic	2/137	35/18670	0.027157	0.046836	0.018649	TOP2A/CAS	2
GO:00327	positive r	2/137	35/18670	0.027157	0.046836	0.018649	MAPK14/RI	2
GO:19029	regulatior	2/137	35/18670	0.027157	0.046836	0.018649	TNF/CASP3	2
GO:00109	negative r	4/137	155/1867(	0.027449	0.04732	0.018841	GSK3B/PTI	4
GO:00324(	regulatior	3/137	89/18670	0.027778	0.047847	0.019051	BCL2L1/AI	3
GO:00600	roof of mc	3/137	89/18670	0.027778	0.047847	0.019051	TGFBR1/EI	3
GO:00018	neural tub	3/137	90/18670	0.028586	0.049093	0.019547	CASP3/TGI	3
GO:00099	dorsal/ver	3/137	90/18670	0.028586	0.049093	0.019547	SMO/GLI1/	3
GO:00309(	midbrain c	3/137	90/18670	0.028586	0.049093	0.019547	FGFR1/FGI	3
GO:00326	regulatior	3/137	90/18670	0.028586	0.049093	0.019547	CASP8/JAI	3
GO:00509	pigment ce	2/137	36/18670	0.028619	0.049093	0.019547	KIT/BCL2	2
GO:00604	lung epith	2/137	36/18670	0.028619	0.049093	0.019547	MAP2K1/F(	2
GO:00716	regulatior	2/137	36/18670	0.028619	0.049093	0.019547	PTGS2/HII	2
GO:00972	amyloid-be	2/137	36/18670	0.028619	0.049093	0.019547	IGF1R/TNI	2
GO:20003	regulatior	2/137	36/18670	0.028619	0.049093	0.019547	DAPK1/EPI	2
GO:20011	positive r	2/137	36/18670	0.028619	0.049093	0.019547	HIF1A/STA	2
