# Supplementary material for: The Efficacy and Mechanism of Qinghua Jianpi Recipe in Inhibiting Canceration of Colorectal Adenoma Based on Inflammatory Cancer Transformation
Source: J Immunol Res. 2023 Feb 15;2023:4319551. doi: 10.1155/2023/4319551 (PMC9946765; doi:10.1155/2023/4319551)
Supplement: Supplementary Materials — The analysis data of the network pharmacology. Active ingredients in traditional Chinese medicine (1); 1011 targets in colorectal cancer (2); PPI topological analysis (3); topological analysis of 213 active components in the network diagram (4); MCODE analysis (5); biological processes (BP, GO enrichment analysis) (6); cell components (CC, GO enrichment analysis) (7); molecular function (MF, GO enrichment analysis) (8); KEGG analysis (9). [file 4319551.f1.zip › CC.pdf]

ID	Descriptic	GeneRatic	BgRatio	pvalue	p.adjust	qvalue	geneID	Count
GO:00451	membrane r	18/137	315/19717	7.16E-12	1.04E-09	7.70E-10	TGFBR1/MA	18
GO:00988	membrane m	18/137	316/19717	7.54E-12	1.04E-09	7.70E-10	TGFBR1/MA	18
GO:00985	membrane r	18/137	328/19717	1.40E-11	1.29E-09	9.54E-10	TGFBR1/MA	18
GO:00059	focal adhe	17/137	405/19717	3.28E-09	1.67E-07	1.24E-07	MAP2K1/MA	17
GO:00616	transferas	14/137	259/19717	3.61E-09	1.67E-07	1.24E-07	TGFBR1/TE	14
GO:00059	cell-subst	17/137	408/19717	3.66E-09	1.67E-07	1.24E-07	MAP2K1/MA	17
GO:00300	cell-subst	17/137	412/19717	4.24E-09	1.67E-07	1.24E-07	MAP2K1/MA	17
GO:00003	cyclin-dep	7/137	42/19717	1.48E-08	5.09E-07	3.77E-07	CDK1/CDK6	7
GO:19029	protein ki	9/137	109/19717	6.86E-08	1.93E-06	1.43E-06	TGFBR1/IG	9
GO:01010	ficolin-1-	11/137	185/19717	6.99E-08	1.93E-06	1.43E-06	MAPK14/CT	11
GO:00059	caveola	8/137	80/19717	8.42E-08	2.11E-06	1.56E-06	MAPK3/SRC	8
GO:00986	chromosoma	14/137	349/19717	1.52E-07	3.49E-06	2.58E-06	TERT/DNMT	14
GO:19025	serine/thr	8/137	88/19717	1.78E-07	3.78E-06	2.80E-06	TGFBR1/CD	8
GO:19048	ficolin-1-	9/137	124/19717	2.09E-07	4.12E-06	3.05E-06	MAPK14/CT	9
GO:00448	plasma mem	8/137	109/19717	9.32E-07	1.71E-05	1.27E-05	MAPK3/SRC	8
GO:00007	chromosome	9/137	161/19717	1.89E-06	3.27E-05	2.42E-05	TERT/CHEK	9
GO:00451	pronucleus	4/137	15/19717	2.87E-06	4.66E-05	3.45E-05	AURKA/CCN	4
GO:00057	mitochondr	9/137	178/19717	4.33E-06	6.64E-05	4.91E-05	PGR/BCL2/	9
GO:00989	glutamater	12/137	349/19717	6.00E-06	8.72E-05	6.45E-05	MAPK14/SH	12
GO:00424	melanosome	7/137	106/19717	9.22E-06	0.000121	8.96E-05	CTSB/MMP1	7
GO:00487	pigment gr	7/137	106/19717	9.22E-06	0.000121	8.96E-05	CTSB/MMP1	7
GO:00319	organelle	9/137	201/19717	1.16E-05	0.000145	0.000107	PGR/BCL2/	9
GO:00198	outer memb	9/137	203/19717	1.25E-05	1.50E-04	1.11E-04	PGR/BCL2/	9
GO:00319	vesicle lu	11/137	339/19717	2.54E-05	2.92E-04	2.16E-04	MAPK14/MA	11
GO:00007	condensed	4/137	26/19717	2.96E-05	3.27E-04	2.42E-04	AURKA/PLK	4
GO:00057	early endo	11/137	350/19717	3.40E-05	3.61E-04	2.67E-04	MAP2K1/MA	11
GO:00430	neuronal c	13/137	497/19717	4.41E-05	4.51E-04	3.34E-04	CYP17A1/T	13
GO:00347	secretory	10/137	321/19717	8.41E-05	0.000829	0.000614	MAPK14/MA	10
GO:00059	cell-cell	12/137	459/19717	8.82E-05	0.000839	0.000621	TGFBR1/KI	12
GO:00009	condensed	3/137	14/19717	0.000113	0.001039	0.000769	PLK1/CCNB	3
GO:00602	cytoplasmi	10/137	338/19717	1.29E-04	1.14E-03	8.47E-04	MAPK14/MA	10
GO:00007	condensed	3/137	15/19717	1.40E-04	1.21E-03	8.96E-04	PLK1/CCNB	3
GO:00311	pseudopodi	3/137	17/19717	0.000208	0.001738	0.001286	MAPK3/MAP	3
GO:00056	transcript	10/137	365/19717	0.000239	0.00194	0.001436	VDR/PPARG	10
GO:00432	myelin she	4/137	49/19717	3.71E-04	0.002925	2.16E-03	BCL2/HSP9	4
GO:00057	late endos	8/137	256/19717	4.28E-04	0.003285	0.00243	MAP2K1/MA	8
GO:00325	ruffle mem	5/137	94/19717	5.03E-04	0.003755	0.002779	SRC/EPHA2	5
GO:00620	collagen-c	10/137	406/19717	0.000552	0.004011	0.002968	CTSB/MMP2	10
GO:00007	condensed	5/137	99/19717	0.000638	0.004516	0.003341	CHEK1/AUR	5
GO:00059	phosphatic	3/137	27/19717	8.50E-04	5.86E-03	4.34E-03	PIK3CA/PI	3
GO:00007	condensed	7/137	223/19717	9.56E-04	6.44E-03	4.76E-03	CHEK1/AUR	7
GO:00708	tertiary g	6/137	164/19717	1.01E-03	6.66E-03	4.92E-03	MMP9/LGAL	6
GO:00017	ruffle	6/137	172/19717	0.001294	0.008146	0.006027	SRC/EPHA2	6
GO:00007	nuclear ch	9/137	377/19717	0.001299	0.008146	0.006027	AR/ESR1/S	9
GO:00099	basal plas	3/137	34/19717	0.001677	0.010288	0.007612	MET/EGFR/	3
GO:00007	nuclear ch	5/137	125/19717	1.81E-03	1.09E-02	8.05E-03	TERT/CDK1	5

GO:003125	cell leadi	9/137	403/19717	0.002048	0.012028	0.0089	SRC/PIK3(	9
GO:000581	spindle	8/137	347/19717	2.98E-03	0.017106	0.012657	MAPK14/MA	8
GO:001632	basolatera	6/137	217/19717	0.004129	0.023255	0.017206	CA9/ABCC1	6
GO:004320	lysosomal	4/137	95/19717	0.004359	0.02406	0.017802	CTSB/PDGI	4
GO:004517	basal part	3/137	51/19717	0.005357	0.028993	0.021452	MET/EGFR/	3
GO:009057	RNA polyme	5/137	163/19717	5.65E-03	3.00E-02	2.22E-02	VDR/PPAR(	5
GO:190472	tertiary g	3/137	55/19717	6.61E-03	0.034405	0.025456	MMP9/CTSI	3
GO:003125	leading ec	5/137	170/19717	0.006731	0.034405	0.025456	SRC/EPHA2	5
GO:000577	vacuolar l	5/137	172/19717	0.007064	0.035449	0.026228	CTSB/MAPI	5
GO:003049	midbody	5/137	173/19717	0.007235	0.035656	0.026382	PIK3CB/CI	5
GO:000587	spindle mi	3/137	59/19717	0.008033	0.038899	0.028781	CDK1/AURI	3
GO:000077	condensed	4/137	118/19717	0.0093	0.044257	0.032745	AURKA/PLI	4
GO:003125	cell proje	7/137	345/19717	1.05E-02	0.049073	0.036309	CA9/SRC/I	7
