# Supplementary material for: The Efficacy and Mechanism of Qinghua Jianpi Recipe in Inhibiting Canceration of Colorectal Adenoma Based on Inflammatory Cancer Transformation
Source: J Immunol Res. 2023 Feb 15;2023:4319551. doi: 10.1155/2023/4319551 (PMC9946765; doi:10.1155/2023/4319551)
Supplement: Supplementary Materials — The analysis data of the network pharmacology. Active ingredients in traditional Chinese medicine (1); 1011 targets in colorectal cancer (2); PPI topological analysis (3); topological analysis of 213 active components in the network diagram (4); MCODE analysis (5); biological processes (BP, GO enrichment analysis) (6); cell components (CC, GO enrichment analysis) (7); molecular function (MF, GO enrichment analysis) (8); KEGG analysis (9). [file 4319551.f1.zip › KEGG analysis.pdf]

| ID | Descripti | GeneRatic | BgRatio | pvalue | p.adjust | qvalue |
|---|---|---|---|---|---|---|
| hsa01521 | EGFR tyrc | 34/134 | 79/8087 | 4.58E-41 | 1.14E-38 | 4.24E-39 |
| hsa05215 | Prostate | 35/134 | 97/8087 | 5.43E-39 | 6.76E-37 | 2.52E-37 |
| hsa05205 | Proteogly | 42/134 | 205/8087 | 2.02E-35 | 1.68E-33 | 6.25E-34 |
| hsa05212 | Pancreati | 30/134 | 76/8087 | 8.63E-35 | 5.37E-33 | 2.00E-33 |
| hsa04151 | PI3K-Akt | 50/134 | 354/8087 | 2.88E-34 | 1.44E-32 | 5.34E-33 |
| hsa01522 | Endocrine | 32/134 | 98/8087 | 5.71E-34 | 2.37E-32 | 8.82E-33 |
| hsa05161 | Hepatitis | 37/134 | 162/8087 | 6.24E-33 | 2.22E-31 | 8.26E-32 |
| hsa05230 | Central c | 27/134 | 70/8087 | 5.48E-31 | 1.70E-29 | 6.34E-30 |
| hsa04933 | AGE-RAGE | 30/134 | 100/8087 | 1.45E-30 | 4.01E-29 | 1.49E-29 |
| hsa05167 | Kaposi sa | 36/134 | 193/8087 | 1.14E-28 | 2.83E-27 | 1.05E-27 |
| hsa05210 | Colorecta | 27/134 | 86/8087 | 3.92E-28 | 8.88E-27 | 3.30E-27 |
| hsa04917 | Prolactin | 25/134 | 70/8087 | 1.04E-27 | 2.15E-26 | 7.99E-27 |
| hsa05220 | Chronic m | 25/134 | 76/8087 | 1.16E-26 | 2.22E-25 | 8.28E-26 |
| hsa04010 | MAPK sign | 40/134 | 294/8087 | 2.11E-26 | 3.76E-25 | 1.40E-25 |
| hsa05211 | Renal cel | 24/134 | 69/8087 | 2.70E-26 | 4.48E-25 | 1.67E-25 |
| hsa05163 | Human cyt | 36/134 | 225/8087 | 3.26E-26 | 5.07E-25 | 1.89E-25 |
| hsa05223 | Non-small | 24/134 | 72/8087 | 8.94E-26 | 1.31E-24 | 4.87E-25 |
| hsa04014 | Ras signa | 36/134 | 232/8087 | 9.90E-26 | 1.37E-24 | 5.10E-25 |
| hsa04926 | Relaxin s | 29/134 | 129/8087 | 1.54E-25 | 2.02E-24 | 7.52E-25 |
| hsa05166 | Human T-c | 35/134 | 219/8087 | 1.87E-25 | 2.33E-24 | 8.66E-25 |
| hsa04012 | ErbB sign | 25/134 | 85/8087 | 2.87E-25 | 3.41E-24 | 1.27E-24 |
| hsa05224 | Breast ca | 30/134 | 147/8087 | 4.45E-25 | 5.04E-24 | 1.87E-24 |
| hsa04066 | HIF-1 sig | 27/134 | 109/8087 | 5.06E-25 | 5.48E-24 | 2.04E-24 |
| hsa05235 | PD-L1 exp | 25/134 | 89/8087 | 1.05E-24 | 1.09E-23 | 4.04E-24 |
| hsa05225 | Hepatocel | 31/134 | 168/8087 | 1.69E-24 | 1.68E-23 | 6.26E-24 |
| hsa04510 | Focal adh | 33/134 | 201/8087 | 2.26E-24 | 2.16E-23 | 8.04E-24 |
| hsa04218 | Cellular | 30/134 | 156/8087 | 2.87E-24 | 2.65E-23 | 9.86E-24 |
| hsa05218 | Melanoma | 23/134 | 72/8087 | 3.13E-24 | 2.78E-23 | 1.03E-23 |
| hsa04068 | FoxO sign | 28/134 | 131/8087 | 5.20E-24 | 4.46E-23 | 1.66E-23 |
| hsa05219 | Bladder c | 19/134 | 41/8087 | 7.12E-24 | 5.91E-23 | 2.20E-23 |
| hsa05226 | Gastric c | 29/134 | 149/8087 | 1.28E-23 | 1.02E-22 | 3.81E-23 |
| hsa04210 | Apoptosis | 28/134 | 136/8087 | 1.57E-23 | 1.22E-22 | 4.55E-23 |
| hsa05221 | Acute mye | 22/134 | 67/8087 | 1.65E-23 | 1.24E-22 | 4.62E-23 |
| hsa05165 | Human pap | 39/134 | 331/8087 | 2.40E-23 | 1.76E-22 | 6.54E-23 |
| hsa05170 | Human imm | 32/134 | 212/8087 | 1.87E-22 | 1.33E-21 | 4.94E-22 |
| hsa05214 | Glioma | 22/134 | 75/8087 | 2.82E-22 | 1.95E-21 | 7.24E-22 |
| hsa04370 | VEGF sign | 20/134 | 59/8087 | 9.22E-22 | 6.21E-21 | 2.31E-21 |
| hsa05160 | Hepatitis | 28/134 | 157/8087 | 1.02E-21 | 6.71E-21 | 2.49E-21 |
| hsa04015 | Rap1 sign | 31/134 | 210/8087 | 1.87E-21 | 1.19E-20 | 4.43E-21 |
| hsa04722 | Neurotrop | 25/134 | 119/8087 | 2.78E-21 | 1.73E-20 | 6.44E-21 |
| hsa05206 | MicroRNAs | 35/134 | 310/8087 | 2.80E-20 | 1.70E-19 | 6.33E-20 |
| hsa04625 | C-type le | 23/134 | 104/8087 | 3.60E-20 | 2.13E-19 | 7.93E-20 |
| hsa04935 | Growth hc | 24/134 | 119/8087 | 5.24E-20 | 3.04E-19 | 1.13E-19 |
| hsa05418 | Fluid she | 25/134 | 139/8087 | 1.57E-19 | 8.90E-19 | 3.31E-19 |
| hsa04668 | TNF signa | 23/134 | 112/8087 | 2.18E-19 | 1.20E-18 | 4.48E-19 |
| hsa04914 | Progester | 22/134 | 100/8087 | 2.86E-19 | 1.55E-18 | 5.75E-19 |

| | | | | | | |
|---|---|---|---|---|---|---|
| hsa05213 | Endometri | 18/134 | 58/8087 | 7.10E-19 | 3.76E-18 | 1.40E-18 |
| hsa04919 | Thyroid h | 23/134 | 121/8087 | 1.38E-18 | 7.18E-18 | 2.67E-18 |
| hsa05162 | Measles | 24/134 | 139/8087 | 2.45E-18 | 1.24E-17 | 4.62E-18 |
| hsa05231 | Choline m | 21/134 | 98/8087 | 3.56E-18 | 1.77E-17 | 6.59E-18 |
| hsa05145 | Toxoplasm | 22/134 | 112/8087 | 3.92E-18 | 1.91E-17 | 7.12E-18 |
| hsa04380 | Osteoclas | 23/134 | 128/8087 | 5.23E-18 | 2.51E-17 | 9.32E-18 |
| hsa05142 | Chagas di | 21/134 | 102/8087 | 8.61E-18 | 4.04E-17 | 1.50E-17 |
| hsa04660 | T cell re | 21/134 | 104/8087 | 1.32E-17 | 6.08E-17 | 2.26E-17 |
| hsa04664 | Fc epsilc | 18/134 | 68/8087 | 1.75E-17 | 7.93E-17 | 2.95E-17 |
| hsa05203 | Viral car | 27/134 | 204/8087 | 1.82E-17 | 8.11E-17 | 3.02E-17 |
| hsa05135 | Yersinia | 23/134 | 137/8087 | 2.56E-17 | 1.12E-16 | 4.17E-17 |
| hsa04915 | Estrogen | 23/134 | 138/8087 | 3.04E-17 | 1.30E-16 | 4.85E-17 |
| hsa01524 | Platinum | 18/134 | 73/8087 | 7.06E-17 | 2.98E-16 | 1.11E-16 |
| hsa05169 | Epstein-E | 26/134 | 202/8087 | 1.56E-16 | 6.49E-16 | 2.42E-16 |
| hsa04620 | Toll-like | 20/134 | 104/8087 | 2.28E-16 | 9.32E-16 | 3.47E-16 |
| hsa04071 | Sphingoli | 21/134 | 119/8087 | 2.44E-16 | 9.78E-16 | 3.64E-16 |
| hsa05222 | Small cel | 19/134 | 92/8087 | 3.31E-16 | 1.31E-15 | 4.87E-16 |
| hsa05164 | Influenza | 24/134 | 171/8087 | 3.49E-16 | 1.36E-15 | 5.05E-16 |
| hsa04140 | Autophagy | 22/134 | 137/8087 | 3.60E-16 | 1.38E-15 | 5.14E-16 |
| hsa04550 | Signaling | 22/134 | 143/8087 | 9.24E-16 | 3.48E-15 | 1.30E-15 |
| hsa05152 | Tuberculc | 24/134 | 180/8087 | 1.16E-15 | 4.30E-15 | 1.60E-15 |
| hsa04659 | Th17 cell | 19/134 | 107/8087 | 6.41E-15 | 2.35E-14 | 8.73E-15 |
| hsa04662 | B cell re | 17/134 | 82/8087 | 1.24E-14 | 4.49E-14 | 1.67E-14 |
| hsa04630 | JAK-STAT | 22/134 | 162/8087 | 1.37E-14 | 4.87E-14 | 1.81E-14 |
| hsa05131 | Shigellos | 26/134 | 246/8087 | 2.00E-14 | 7.03E-14 | 2.61E-14 |
| hsa04062 | Chemokine | 23/134 | 192/8087 | 5.16E-14 | 1.78E-13 | 6.64E-14 |
| hsa04657 | IL-17 sig | 17/134 | 94/8087 | 1.37E-13 | 4.67E-13 | 1.74E-13 |
| hsa04115 | p53 signa | 15/134 | 73/8087 | 5.67E-13 | 1.91E-12 | 7.10E-13 |
| hsa05140 | Leishmani | 15/134 | 77/8087 | 1.30E-12 | 4.32E-12 | 1.61E-12 |
| hsa04912 | GnRH sign | 16/134 | 93/8087 | 1.69E-12 | 5.52E-12 | 2.05E-12 |
| hsa05132 | Salmonell | 24/134 | 249/8087 | 1.75E-12 | 5.66E-12 | 2.10E-12 |
| hsa04150 | mTOR sign | 19/134 | 155/8087 | 6.56E-12 | 2.09E-11 | 7.79E-12 |
| hsa04910 | Insulin s | 18/134 | 137/8087 | 7.46E-12 | 2.35E-11 | 8.74E-12 |
| hsa04360 | Axon guid | 20/134 | 182/8087 | 1.36E-11 | 4.23E-11 | 1.57E-11 |
| hsa04929 | GnRH secr | 13/134 | 64/8087 | 2.52E-11 | 7.76E-11 | 2.89E-11 |
| hsa04072 | Phospholi | 18/134 | 148/8087 | 2.81E-11 | 8.55E-11 | 3.18E-11 |
| hsa04650 | Natural k | 17/134 | 131/8087 | 3.64E-11 | 1.09E-10 | 4.07E-11 |
| hsa04810 | Regulatic | 21/134 | 218/8087 | 5.06E-11 | 1.50E-10 | 5.58E-11 |
| hsa04110 | Cell cycl | 16/134 | 124/8087 | 1.56E-10 | 4.58E-10 | 1.70E-10 |
| hsa04931 | Insulin r | 15/134 | 108/8087 | 2.09E-10 | 6.06E-10 | 2.25E-10 |
| hsa05216 | Thyroid c | 10/134 | 37/8087 | 2.65E-10 | 7.59E-10 | 2.82E-10 |
| hsa05171 | Coronavir | 20/134 | 232/8087 | 1.11E-09 | 3.15E-09 | 1.17E-09 |
| hsa05120 | Epithelia | 12/134 | 70/8087 | 1.22E-09 | 3.43E-09 | 1.27E-09 |
| hsa05010 | Alzheimer | 25/134 | 369/8087 | 1.25E-09 | 3.46E-09 | 1.29E-09 |
| hsa04520 | Adherens | 12/134 | 71/8087 | 1.45E-09 | 3.98E-09 | 1.48E-09 |
| hsa05202 | Transcrip | 18/134 | 192/8087 | 2.14E-09 | 5.78E-09 | 2.15E-09 |
| hsa04024 | cAMP sign | 19/134 | 216/8087 | 2.16E-09 | 5.78E-09 | 2.15E-09 |

| | | | | | | |
|---|---|---|---|---|---|---|
| hsa04932 | Non-alcoh | 16/134 | 150/8087 | 2.74E-09 | 7.26E-09 | 2.70E-09 |
| hsa05133 | Pertussis | 12/134 | 76/8087 | 3.28E-09 | 8.60E-09 | 3.20E-09 |
| hsa04621 | NOD-like | 17/134 | 181/8087 | 6.04E-09 | 1.57E-08 | 5.82E-09 |
| hsa04020 | Calcium s | 19/134 | 240/8087 | 1.25E-08 | 3.20E-08 | 1.19E-08 |
| hsa04613 | Neutrophi | 17/134 | 190/8087 | 1.26E-08 | 3.21E-08 | 1.19E-08 |
| hsa04920 | Adipocytc | 11/134 | 69/8087 | 1.38E-08 | 3.46E-08 | 1.29E-08 |
| hsa04540 | Gap junct | 12/134 | 88/8087 | 1.83E-08 | 4.57E-08 | 1.70E-08 |
| hsa04211 | Longevity | 12/134 | 89/8087 | 2.09E-08 | 5.16E-08 | 1.92E-08 |
| hsa04670 | Leukocyte | 13/134 | 114/8087 | 4.12E-08 | 1.01E-07 | 3.74E-08 |
| hsa04930 | Type II d | 9/134 | 46/8087 | 4.71E-08 | 1.14E-07 | 4.24E-08 |
| hsa04666 | Fc gamma | 12/134 | 97/8087 | 5.61E-08 | 1.34E-07 | 5.00E-08 |
| hsa05146 | Amoebiasi | 12/134 | 102/8087 | 9.91E-08 | 2.35E-07 | 8.74E-08 |
| hsa04064 | NF-kappa | 12/134 | 104/8087 | 1.23E-07 | 2.90E-07 | 1.08E-07 |
| hsa05130 | Pathogeni | 16/134 | 197/8087 | 1.36E-07 | 3.17E-07 | 1.18E-07 |
| hsa04114 | Oocyte me | 13/134 | 129/8087 | 1.81E-07 | 4.18E-07 | 1.56E-07 |
| hsa04658 | Th1 and T | 11/134 | 92/8087 | 2.94E-07 | 6.71E-07 | 2.50E-07 |
| hsa04725 | Cholinerg | 12/134 | 113/8087 | 3.11E-07 | 7.03E-07 | 2.62E-07 |
| hsa04371 | Apelin si | 13/134 | 137/8087 | 3.68E-07 | 8.25E-07 | 3.07E-07 |
| hsa04730 | Long-term | 9/134 | 60/8087 | 5.20E-07 | 1.16E-06 | 4.30E-07 |
| hsa01523 | Antifolat | 7/134 | 31/8087 | 5.53E-07 | 1.22E-06 | 4.53E-07 |
| hsa04152 | AMPK sign | 12/134 | 120/8087 | 6.00E-07 | 1.31E-06 | 4.88E-07 |
| hsa04213 | Longevity | 9/134 | 62/8087 | 6.94E-07 | 1.50E-06 | 5.59E-07 |
| hsa05022 | Pathways | 24/134 | 475/8087 | 7.58E-07 | 1.63E-06 | 6.05E-07 |
| hsa05321 | Inflammat | 9/134 | 65/8087 | 1.05E-06 | 2.23E-06 | 8.30E-07 |
| hsa04928 | Parathyrc | 11/134 | 106/8087 | 1.25E-06 | 2.63E-06 | 9.79E-07 |
| hsa04720 | Long-term | 9/134 | 67/8087 | 1.36E-06 | 2.85E-06 | 1.06E-06 |
| hsa04934 | Cushing s | 13/134 | 155/8087 | 1.52E-06 | 3.16E-06 | 1.18E-06 |
| hsa04916 | Melanogen | 10/134 | 101/8087 | 5.95E-06 | 1.22E-05 | 4.56E-06 |
| hsa04921 | Oxytocin | 12/134 | 154/8087 | 8.44E-06 | 1.72E-05 | 6.41E-06 |
| hsa04217 | Necroptos | 12/134 | 159/8087 | 1.17E-05 | 2.36E-05 | 8.79E-06 |
| hsa04215 | Apoptosis | 6/134 | 32/8087 | 1.18E-05 | 2.36E-05 | 8.79E-06 |
| hsa05323 | Rheumatoi | 9/134 | 93/8087 | 2.13E-05 | 4.25E-05 | 1.58E-05 |
| hsa04960 | Aldosterc | 6/134 | 37/8087 | 2.82E-05 | 5.57E-05 | 2.07E-05 |
| hsa04750 | Inflammat | 9/134 | 98/8087 | 3.25E-05 | 6.38E-05 | 2.37E-05 |
| hsa04611 | Platelet | 10/134 | 124/8087 | 3.66E-05 | 7.12E-05 | 2.65E-05 |
| hsa05100 | Bacterial | 8/134 | 77/8087 | 3.71E-05 | 7.16E-05 | 2.66E-05 |
| hsa04726 | Serotoner | 9/134 | 115/8087 | 0.000115 | 0.00022 | 8.17E-05 |
| hsa04137 | Mitophagy | 7/134 | 68/8087 | 0.000123 | 0.000233 | 8.66E-05 |
| hsa04350 | TGF-beta | 8/134 | 94/8087 | 0.000155 | 0.000292 | 0.000109 |
| hsa05168 | Herpes si | 20/134 | 498/8087 | 0.000187 | 0.00035 | 0.00013 |
| hsa04923 | Regulatic | 6/134 | 57/8087 | 0.000336 | 0.000619 | 0.00023 |
| hsa05134 | Legionell | 6/134 | 57/8087 | 0.000336 | 0.000619 | 0.00023 |
| hsa05020 | Prion dis | 13/134 | 273/8087 | 0.00057 | 0.001043 | 0.000388 |
| hsa04973 | Carbohydr | 5/134 | 47/8087 | 0.001017 | 0.001836 | 0.000683 |
| hsa04622 | RIG-I-lik | 6/134 | 70/8087 | 0.001017 | 0.001836 | 0.000683 |
| hsa04310 | Wnt signa | 9/134 | 160/8087 | 0.001313 | 0.002352 | 0.000875 |
| hsa04340 | Hedgehog | 5/134 | 50/8087 | 0.00135 | 0.002385 | 0.000887 |

| | | | | | | |
|---|---|---|---|---|---|---|
| hsa05144 | Malaria | 5/134 | 50/8087 | 0.00135 | 0.002385 | 0.000887 |
| hsa04270 | Vascular | 8/134 | 133/8087 | 0.001592 | 0.002791 | 0.001038 |
| hsa05017 | Spinocere | 8/134 | 143/8087 | 0.002515 | 0.004379 | 0.001629 |
| hsa05416 | Viral myc | 5/134 | 60/8087 | 0.003051 | 0.005276 | 0.001963 |
| hsa05143 | African t | 4/134 | 37/8087 | 0.003116 | 0.005351 | 0.001991 |
| hsa04022 | cGMP-PKG | 8/134 | 167/8087 | 0.006441 | 0.010985 | 0.004087 |
| hsa04530 | Tight jun | 8/134 | 169/8087 | 0.006907 | 0.0117 | 0.004352 |
| hsa04144 | Endocytos | 10/134 | 252/8087 | 0.00892 | 0.015008 | 0.005583 |
| hsa04913 | Ovarian s | 4/134 | 51/8087 | 0.009869 | 0.016492 | 0.006135 |
| hsa04261 | Adrenergi | 7/134 | 150/8087 | 0.012149 | 0.020167 | 0.007502 |
| hsa04728 | Dopaminer | 6/134 | 132/8087 | 0.022168 | 0.036554 | 0.013599 |

| geneID | Count |
|---|---|
| MAP2K1/MAPK3/SRC/KDR/MET/IGF1R/GSK3B/M | 34 |
| AR/MAP2K1/MAPK3/IGF1R/GSK3B/MMP9/MAPK1 | 35 |
| ESR1/MAPK14/MAP2K1/MAPK3/PTPN11/SRC/KD | 42 |
| TGFBR1/MAP2K1/MAPK3/STAT1/MAPK1/MTOR/P | 30 |
| MAP2K1/MAPK3/KDR/MET/IGF1R/GSK3B/MAPK1 | 50 |
| ESR1/ESR2/MAPK14/MAP2K1/MAPK3/SRC/IGF1 | 32 |
| MAPK14/TGFBR1/MAP2K1/MAPK3/SRC/MMP9/ST | 37 |
| MAP2K1/MAPK3/MET/RET/MAPK1/FGFR1/PDGFR | 27 |
| MAPK14/TGFBR1/MAPK3/MMP2/STAT1/MAPK1/B | 30 |
| MAPK14/MAP2K1/MAPK3/SRC/GSK3B/STAT1/MA | 36 |
| TGFBR1/MAP2K1/MAPK3/GSK3B/MAPK1/BCL2/M | 27 |
| CYP17A1/ESR1/ESR2/MAPK14/MAP2K1/MAPK3/ | 25 |
| TGFBR1/MAP2K1/MAPK3/PTPN11/MAPK1/PIK3C | 25 |
| MAPK14/TGFBR1/MAP2K1/MAPK3/KDR/MET/IGF | 40 |
| MAP2K1/MAPK3/PTPN11/MET/MAPK1/PIK3CA/P | 24 |
| MAPK14/MAP2K1/MAPK3/SRC/GSK3B/MAPK1/MT | 36 |
| MAP2K1/MAPK3/MET/RET/MAPK1/PIK3CA/PIK3 | 24 |
| MAP2K1/MAPK3/PTPN11/KDR/PLA2G2A/MET/IG | 36 |
| NOS2/MAPK14/TGFBR1/MAP2K1/MAPK3/SRC/MM | 29 |
| TGFBR1/MAP2K1/TERT/MAPK3/MAPK1/CHEK1/P | 35 |
| MAP2K1/MAPK3/SRC/GSK3B/MAPK1/MTOR/PIK3 | 25 |
| ESR1/ESR2/MAP2K1/PGR/MAPK3/IGF1R/GSK3B | 30 |
| NOS2/MAP2K1/MAPK3/IGF1R/MAPK1/BCL2/MTO | 27 |
| MAPK14/MAP2K1/MAPK3/PTPN11/STAT1/MAPK1 | 25 |
| TGFBR1/MAP2K1/TERT/MAPK3/MET/IGF1R/GSK | 31 |
| MAP2K1/MAPK3/SRC/KDR/MET/IGF1R/GSK3B/M | 33 |
| MAPK14/TGFBR1/MAP2K1/MAPK3/MAPK1/CHEK1 | 30 |
| MAP2K1/MAPK3/MET/IGF1R/MAPK1/FGFR1/PDG | 23 |
| MAPK14/TGFBR1/MAP2K1/MAPK3/IGF1R/MAPK1 | 28 |
| MAP2K1/MAPK3/SRC/MMP2/MMP9/MAPK1/VEGFA | 19 |
| TGFBR1/MAP2K1/TERT/MAPK3/ABCB1/MET/GSK | 29 |
| MAP2K1/CTSB/MAPK3/MAPK1/BCL2/PIK3CA/PI | 28 |
| MAP2K1/MAPK3/MAPK1/KIT/MTOR/PIK3CA/PIK | 22 |
| MAP2K1/TERT/MAPK3/GSK3B/STAT1/MAPK1/PD | 39 |
| MAPK14/MAP2K1/MAPK3/MAPK1/BCL2/CHEK1/M | 32 |
| MAP2K1/MAPK3/IGF1R/MAPK1/PDGFRB/MTOR/P | 22 |
| MAPK14/MAP2K1/MAPK3/SRC/KDR/MAPK1/PIK3 | 20 |
| MAP2K1/MAPK3/GSK3B/STAT1/MAPK1/PIK3CA/ | 28 |
| MAPK14/MAP2K1/MAPK3/SRC/KDR/MET/IGF1R/ | 31 |
| MAPK14/MAP2K1/MAPK3/PTPN11/GSK3B/MAPK1 | 25 |
| MAP2K1/MAPK3/ABCC1/ABCB1/MET/MMP9/DNMT | 35 |
| MAPK14/MAPK3/PTPN11/SRC/STAT1/MAPK1/PI | 23 |
| MAPK14/MAP2K1/MAPK3/GSK3B/STAT1/MAPK1/ | 24 |
| MAPK14/SRC/KDR/MMP2/MMP9/BCL2/PIK3CA/P | 25 |
| MAPK14/MAP2K1/MAPK3/MMP9/MAPK1/MMP14/M | 23 |
| MAPK14/MAP2K1/PGR/MAPK3/IGF1R/MAPK1/PI | 22 |

| | |
|---|---|
| MAP2K1/MAPK3/GSK3B/MAPK1/PIK3CA/PIK3CB | 18 |
| ESR1/MAP2K1/MAPK3/SRC/GSK3B/STAT1/MAPK | 23 |
| GSK3B/STAT1/BCL2/PIK3CA/PIK3CB/BCL2L1/ | 24 |
| MAP2K1/MAPK3/MAPK1/PDGFRB/MTOR/PIK3CA/ | 21 |
| NOS2/MAPK14/MAPK3/STAT1/MAPK1/BCL2/BCL | 22 |
| PPARG/MAPK14/TGFBR1/MAP2K1/MAPK3/STAT1 | 23 |
| NOS2/MAPK14/TGFBR1/MAPK3/MAPK1/PIK3CA/ | 21 |
| MAPK14/MAP2K1/MAPK3/GSK3B/MAPK1/PIK3CA | 21 |
| MAPK14/MAP2K1/MAPK3/MAPK1/PIK3CA/PIK3C | 18 |
| MAPK3/SRC/MAPK1/CHEK1/PIK3CA/PIK3CB/ST | 27 |
| MAPK14/MAP2K1/MAPK3/SRC/GSK3B/MAPK1/PI | 23 |
| ESR1/ESR2/MAP2K1/PGR/MAPK3/SRC/MMP2/MM | 23 |
| MAPK3/MAPK1/BCL2/PIK3CA/PIK3CB/BCL2L1/ | 18 |
| MAPK14/STAT1/BCL2/PIK3CA/PIK3CB/STAT3/ | 26 |
| MAPK14/MAP2K1/MAPK3/STAT1/MAPK1/PIK3CA | 20 |
| MAPK14/MAP2K1/MAPK3/ABCC1/MAPK1/BCL2/P | 21 |
| NOS2/BCL2/PIK3CA/PIK3CB/BCL2L1/PTGS2/C | 19 |
| MAP2K1/MAPK3/STAT1/MAPK1/PIK3CA/PIK3CB | 24 |
| MAP2K1/CTSB/MAPK3/IGF1R/MAPK1/BCL2/MTO | 22 |
| MAPK14/MAP2K1/MAPK3/IGF1R/GSK3B/MAPK1/ | 22 |
| VDR/NOS2/MAPK14/MAPK3/SRC/STAT1/MAPK1/ | 24 |
| MAPK14/TGFBR1/MAPK3/STAT1/MAPK1/MTOR/H | 19 |
| MAP2K1/MAPK3/GSK3B/MAPK1/PIK3CA/PIK3CB | 17 |
| PTPN11/STAT1/PDGFRB/BCL2/MTOR/PIK3CA/P | 22 |
| MAPK14/MAPK3/SRC/GSK3B/MAPK1/BCL2/MTOR | 26 |
| MAP2K1/MAPK3/SRC/GSK3B/STAT1/MAPK1/PIK | 23 |
| MAPK14/MAPK3/GSK3B/MMP9/MAPK1/MMP3/PTG | 17 |
| BCL2/CHEK1/IGFBP3/BCL2L1/CDK1/CDK6/CCN | 15 |
| NOS2/MAPK14/MAPK3/STAT1/MAPK1/PTGS2/TN | 15 |
| MAPK14/MAP2K1/MAPK3/SRC/MMP2/MAPK1/MMP | 16 |
| MAPK14/MAP2K1/MAPK3/MAPK1/BCL2/PIK3CA/ | 24 |
| MAP2K1/MAPK3/IGF1R/GSK3B/MAPK1/MTOR/PI | 19 |
| MAP2K1/MAPK3/GSK3B/MAPK1/MTOR/PIK3CA/P | 18 |
| MAPK3/PTPN11/SRC/MET/GSK3B/MAPK1/PIK3C | 20 |
| ESR2/MAP2K1/MAPK3/MAPK1/PIK3CA/PIK3CB/ | 13 |
| MAP2K1/MAPK3/PTPN11/MAPK1/PDGFRB/KIT/M | 18 |
| MAP2K1/MAPK3/PTPN11/MAPK1/PIK3CA/PIK3C | 17 |
| MAP2K1/MAPK3/SRC/MAPK1/FGFR1/PDGFRB/PI | 21 |
| GSK3B/CHEK1/CDK1/CDK6/PLK1/CCNB1/CCND1 | 16 |
| PTPN11/GSK3B/MTOR/PIK3CA/PIK3CB/STAT3/ | 15 |
| PPARG/MAP2K1/MAPK3/RET/MAPK1/HRAS/CCND | 10 |
| MAPK14/MAPK3/STAT1/MAPK1/MMP3/PIK3CA/P | 20 |
| MAPK14/PTPN11/SRC/MET/EGFR/CASP3/JUN/M | 12 |
| NOS2/MAP2K1/MAPK3/GSK3B/MAPK1/MTOR/PIK | 25 |
| TGFBR1/MAPK3/SRC/MET/IGF1R/MAPK1/FGFR1 | 12 |
| PPARG/MET/IGF1R/MMP9/MMP3/IGFBP3/BCL2L | 18 |
| MAP2K1/MAPK3/MAPK1/PIK3CA/PIK3CB/AKT1/ | 19 |

| | |
|---|---|
| GSK3B/PIK3CA/PIK3CB/TNF/AKT1/CASP3/CAS | 16 |
| NOS2/MAPK14/MAPK3/MAPK1/TNF/CASP3/JUN/ | 12 |
| MAPK14/CTSB/MAPK3/STAT1/MAPK1/BCL2/BCL | 17 |
| NOS2/KDR/MET/RET/FGFR1/PDGFRB/VEGFA/FG | 19 |
| MAPK14/MAP2K1/MAPK3/SRC/MAPK1/MTOR/PIK | 17 |
| PTPN11/MTOR/STAT3/TNF/AKT1/JAK2/MAPK8/ | 11 |
| MAP2K1/MAPK3/SRC/MAPK1/PDGFRB/CDK1/PRK | 12 |
| PPARG/IGF1R/MTOR/PIK3CA/PIK3CB/HRAS/AK | 12 |
| MAPK14/PTPN11/MMP2/MMP9/PIK3CA/PIK3CB/ | 13 |
| MAPK3/MAPK1/MTOR/PIK3CA/PIK3CB/TNF/MAP | 9 |
| MAP2K1/MAPK3/MAPK1/PIK3CA/PIK3CB/PRKCA | 12 |
| NOS2/PIK3CA/PIK3CB/PRKCA/TNF/PTK2/CASP | 12 |
| BCL2/BCL2L1/PTGS2/PARP1/TNF/ICAM1/XIAP | 12 |
| MAPK14/MAPK3/PTPN11/SRC/MAPK1/TNF/GAPD | 16 |
| AR/MAPK14/MAP2K1/PGR/MAPK3/IGF1R/MAPK1 | 13 |
| MAPK14/MAPK3/STAT1/MAPK1/IL2/JUN/JAK2/ | 11 |
| MAP2K1/MAPK3/MAPK1/BCL2/PIK3CA/PIK3CB/ | 12 |
| NOS2/TGFBR1/MAP2K1/MAPK3/MAPK1/MTOR/HR | 13 |
| MAP2K1/MAPK3/IGF1R/MAPK1/PRKCA/HRAS/BR | 9 |
| ABCC1/ABCG2/TNF/NFKB1/TYMS/IL1B/RELA | 7 |
| PPARG/IGF1R/MTOR/PIK3CA/PIK3CB/HNF4A/C | 12 |
| IGF1R/MTOR/PIK3CA/PIK3CB/HRAS/AKT1/RPS | 9 |
| NOS2/MAPK14/MAP2K1/MAPK3/GSK3B/MAPK1/B | 24 |
| STAT1/STAT3/TNF/IL2/JUN/NFKB1/IL1B/REL | 9 |
| VDR/MAP2K1/MAPK3/MAPK1/FGFR1/MMP14/BCL | 11 |
| MAP2K1/MAPK3/MAPK1/PRKCA/HRAS/BRAF/RAF | 9 |
| CYP17A1/MAP2K1/MAPK3/GSK3B/MAPK1/EGFR/ | 13 |
| MAP2K1/MAPK3/GSK3B/MAPK1/KIT/PRKCA/HRA | 10 |
| MAP2K1/MAPK3/SRC/MAPK1/PTGS2/PRKCA/HRA | 12 |
| STAT1/BCL2/HSP90AA1/STAT3/PARP1/TNF/CA | 12 |
| BCL2/BCL2L1/CASP3/CASP8/XIAP/MAPK8 | 6 |
| MMP3/VEGFA/MMP1/TNF/JUN/FLT1/ICAM1/IL1 | 9 |
| MAPK3/MAPK1/PIK3CA/PIK3CB/PRKCA/PIK3R1 | 6 |
| MAPK14/SRC/PIK3CA/PIK3CB/PRKCA/NTRK1/M | 9 |
| MAPK14/MAPK3/SRC/MAPK1/PIK3CA/PIK3CB/A | 10 |
| SRC/MET/PIK3CA/PIK3CB/PTK2/PIK3R1/ITGB | 8 |
| MAP2K1/MAPK3/MAPK1/PTGS2/PRKCA/HRAS/BR | 9 |
| SRC/BCL2L1/HIF1A/HRAS/JUN/MAPK8/RELA | 7 |
| TGFBR1/MAPK3/MAPK1/TNF/RPS6KB1/EP300/T | 8 |
| PTPN11/SRC/STAT1/BCL2/MTOR/PIK3CA/PIK3 | 20 |
| PIK3CA/PIK3CB/PTGS2/AKT1/PIK3R1/AKT2 | 6 |
| TNF/CASP3/CASP8/NFKB1/IL1B/RELA | 6 |
| MAPK14/MAPK3/GSK3B/MAPK1/PIK3CA/PIK3CB | 13 |
| PIK3CA/PIK3CB/AKT1/PIK3R1/AKT2 | 5 |
| MAPK14/TNF/CASP8/MAPK8/NFKB1/RELA | 6 |
| GSK3B/PRKCA/MMP7/CCND1/JUN/MAPK8/EP300 | 9 |
| GSK3B/BCL2/CCND1/SMO/GLI1 | 5 |

| | |
|---|---|
| MET/TNF/ICAM1/IL1B/TGFB1 | 5 |
| MAP2K1/MAPK3/PLA2G2A/MAPK1/PRKCA/BRAF/ | 8 |
| MTOR/PIK3CA/PIK3CB/PRKCA/AKT1/MAPK8/PI | 8 |
| CCND1/CASP3/CASP8/ICAM1/RAC1 | 5 |
| PRKCA/TNF/ICAM1/IL1B | 4 |
| MAP2K1/MAPK3/MAPK1/AKT1/RAF1/MAP2K2/AK | 8 |
| SRC/ERBB2/CCND1/CDK4/JUN/MAPK8/ITGB1/R | 8 |
| TGFBR1/SRC/IGF1R/HRAS/EGFR/MDM2/FGFR2/ | 10 |
| CYP17A1/IGF1R/PTGS2/CYP1A1 | 4 |
| MAPK14/MAPK3/MAPK1/BCL2/PRKCA/AKT1/AKT | 7 |
| MAPK14/GSK3B/PRKCA/AKT1/MAPK8/AKT2 | 6 |
