# Supplementary material for: The Efficacy and Mechanism of Qinghua Jianpi Recipe in Inhibiting Canceration of Colorectal Adenoma Based on Inflammatory Cancer Transformation
Source: J Immunol Res. 2023 Feb 15;2023:4319551. doi: 10.1155/2023/4319551 (PMC9946765; doi:10.1155/2023/4319551)
Supplement: Supplementary Materials — The analysis data of the network pharmacology. Active ingredients in traditional Chinese medicine (1); 1011 targets in colorectal cancer (2); PPI topological analysis (3); topological analysis of 213 active components in the network diagram (4); MCODE analysis (5); biological processes (BP, GO enrichment analysis) (6); cell components (CC, GO enrichment analysis) (7); molecular function (MF, GO enrichment analysis) (8); KEGG analysis (9). [file 4319551.f1.zip › MCODE analysis.pdf]

MCODE App Results

Parameters:
Network Scoring:
Include Loops: false Degree Cutoff: 2
Cluster Finding:
Node Score Cutoff: 0.2 Haircut: true Flu

Cluster	Score (Density*Nodes	Edges	
1	44.069	59	1278
2	8.462	27	110
3	3.833	13	23

ıff: false K-Core: 2 Max. Depth from Seed: 100

Node IDs
VEGFA, CASP3, AKT1, ATM, HRAS, CCNA2, ESR1, IGF1R, TNF, ICAM1, MAPK8, SRC, IL1B, JUN, HIF1A
AURKA, ABCB1, PLK1, EZH2, CDK2, RET, DNMT1, CDK6, CHEK2, RAC1, PIK3CA, CXCR4, ITGB1, PIK3R
PIK3CB, TYMS, SLC2A1, FGFR1, TOP2A, MGMT, PKM, FLT4, GLI1, FGFR2, FGFR3, ABCG2, TOP1

[illegible] BCL2L1, CCNB1, RAF1, RELA, EGFR, ERBB2, STAT3, CDK1, MAPK14, FGF2, KDR, GRB2, EF[illegible]
[illegible]FLT1, CDK4, ALK, PDGFRB, GSK3B, KIT, HNF4A, TERT, MMP14, AKT2, RPS6KB1

[illegible]300, NFKB1, MAPK3, TGFB1, GAPDH, PLAU, MMP2, CCND1, MMP9, IL2, XIAP, MAPK1, JAK2,

MCL1, PARP1, MTOR, PGR, STAT1, PPARG, CASP8, JAK1, PTK2, PTPN11, MET, AR, MMP1, [illegible]

CHEK1, MAP2K1, MMP3, MDM2
