# Supplementary material for: The Efficacy and Mechanism of Qinghua Jianpi Recipe in Inhibiting Canceration of Colorectal Adenoma Based on Inflammatory Cancer Transformation
Source: J Immunol Res. 2023 Feb 15;2023:4319551. doi: 10.1155/2023/4319551 (PMC9946765; doi:10.1155/2023/4319551)
Supplement: Supplementary Materials — The analysis data of the network pharmacology. Active ingredients in traditional Chinese medicine (1); 1011 targets in colorectal cancer (2); PPI topological analysis (3); topological analysis of 213 active components in the network diagram (4); MCODE analysis (5); biological processes (BP, GO enrichment analysis) (6); cell components (CC, GO enrichment analysis) (7); molecular function (MF, GO enrichment analysis) (8); KEGG analysis (9). [file 4319551.f1.zip › MF.pdf]

ID	Descriptio	GeneRatio	BgRatio	pvalue	p.adjust	qvalue	geneID	Count
GO:0004713	protein ty	27/137	134/17697	5.10E-31	2.31E-28	1.48E-28	MAP2K1/SRC	27
GO:0019199	transmembr	22/137	79/17697	8.40E-29	1.90E-26	1.22E-26	TGFBR1/KDR	22
GO:0004714	transmembr	20/137	62/17697	9.92E-28	1.50E-25	9.61E-26	KDR/MET/IG	20
GO:0004674	protein se	31/137	439/17697	2.85E-21	3.22E-19	2.07E-19	MAPK14/TGF	31
GO:0019902	phosphatas	18/137	185/17697	4.59E-15	4.16E-13	2.67E-13	PPARG/MAPK	18
GO:0019903	protein ph	16/137	140/17697	1.29E-14	9.74E-13	6.24E-13	PPARG/MAPK	16
GO:0019838	growth fac	15/137	137/17697	1.72E-13	1.11E-11	7.12E-12	TGFBR1/KDR	15
GO:0043560	insulin re	7/137	11/17697	4.59E-13	2.60E-11	1.67E-11	PTPN11/IGF	7
GO:0035173	histone ki	7/137	17/17697	2.60E-11	1.31E-09	8.40E-10	CHEK1/CDK1	7
GO:0004712	protein se	9/137	43/17697	3.46E-11	1.57E-09	1.00E-09	MAPK14/MAP	9
GO:0005126	cytokine r	16/137	286/17697	7.30E-10	3.01E-08	1.93E-08	TGFBR1/STA	16
GO:0004708	MAP kinase	6/137	16/17697	1.45E-09	5.47E-08	3.51E-08	MAPK14/MAP	6
GO:0044389	ubiquitin-	16/137	308/17697	2.14E-09	7.44E-08	4.77E-08	SRC/GSK3B/	16
GO:0051427	hormone re	12/137	185/17697	2.12E-08	6.88E-07	4.41E-07	ESR1/VDR/P	12
GO:0031625	ubiquitin	14/137	290/17697	5.74E-08	1.73E-06	1.11E-06	SRC/GSK3B/	14
GO:0004879	nuclear re	7/137	47/17697	6.94E-08	1.85E-06	1.19E-06	AR/ESR1/ES	7
GO:0098531	transcript	7/137	47/17697	6.94E-08	1.85E-06	1.19E-06	AR/ESR1/ES	7
GO:0019207	kinase reg	12/137	207/17697	7.37E-08	1.85E-06	1.19E-06	MAP2K1/PIK	12
GO:0003707	steroid ho	7/137	56/17697	2.41E-07	5.76E-06	3.69E-06	AR/ESR1/ES	7
GO:0005003	ephrin rec	5/137	19/17697	2.75E-07	6.24E-06	4.00E-06	EPHA2/NTRK	5
GO:0097110	scaffold p	7/137	59/17697	3.48E-07	7.52E-06	4.82E-06	MAP2K1/MAP	7
GO:0005178	integrin b	9/137	132/17697	8.70E-07	1.79E-05	1.15E-05	SRC/KDR/MM	9
GO:0047485	protein N-	8/137	109/17697	2.07E-06	4.07E-05	2.61E-05	MAP2K1/TER	8
GO:0042910	xenobiotic	4/137	13/17697	2.33E-06	4.30E-05	2.76E-05	ABCC1/ABCG	4
GO:0042826	histone de	8/137	111/17697	2.37E-06	4.30E-05	2.76E-05	HIF1A/HSP9	8
GO:0035257	nuclear ho	9/137	152/17697	2.82E-06	4.92E-05	3.15E-05	ESR1/VDR/P	9
GO:0097472	cyclin-dep	5/137	30/17697	3.15E-06	5.24E-05	3.36E-05	CDK1/CDK6/	5
GO:0004707	MAP kinase	4/137	14/17697	3.24E-06	5.24E-05	3.36E-05	MAPK14/MAP	4
GO:0051219	phosphopro	7/137	83/17697	3.63E-06	5.68E-05	3.64E-05	MAPK3/PTPN	7
GO:0042169	SH2 domain	5/137	36/17697	8.03E-06	0.0001213	7.78E-05	SRC/FGFR1/	5
GO:0070851	growth fac	8/137	134/17697	9.65E-06	0.000141	9.04E-05	SRC/PDGFRB	8
GO:0019887	protein ki	9/137	180/17697	1.12E-05	0.0001584	0.0001016	MAP2K1/PIK	9
GO:0001784	phosphotyr	5/137	40/17697	1.37E-05	0.0001877	0.0001204	MAPK3/PTPN	5
GO:0008022	protein C-	9/137	187/17697	1.52E-05	0.0002023	0.0001297	PPARG/MAP2	9
GO:0005158	insulin re	4/137	22/17697	2.26E-05	0.000284	0.0001821	PTPN11/SRC	4
GO:0001618	virus rece	6/137	74/17697	2.32E-05	0.000284	0.0001821	CDK1/EGFR/	6
GO:0104005	hijacked m	6/137	74/17697	2.32E-05	0.000284	0.0001821	CDK1/EGFR/	6
GO:0001085	RNA polyme	8/137	155/17697	2.78E-05	0.0003315	0.0002126	AR/ESR1/PP	8
GO:0050839	cell adhes	14/137	499/17697	3.33E-05	0.0003864	0.0002478	PTPN11/SRC	14
GO:0016538	cyclin-dep	5/137	49/17697	3.75E-05	0.0004244	0.0002722	CCNB1/CCND	5
GO:0045309	protein ph	5/137	51/17697	4.56E-05	0.0005038	0.0003231	MAPK3/PTPN	5
GO:0005165	neurotroph	3/137	10/17697	5.23E-05	0.0005269	0.0003379	NTRK1/PIK3	3
GO:0016303	1-phosphat	3/137	10/17697	5.23E-05	0.0005269	0.0003379	PIK3CA/PIK	3
GO:0051400	BH domain	3/137	10/17697	5.23E-05	0.0005269	0.0003379	BCL2/BCL2L	3
GO:0070513	death doma	3/137	10/17697	5.23E-05	0.0005269	0.0003379	BCL2/BCL2L	3
GO:0019209	kinase act	6/137	86/17697	5.46E-05	0.0005373	0.0003446	MAP2K1/PIK	6

GO:0046935	cyclin-dep	4/137	29/17697	7.02E-05	0.0006754	0.0004331	CDK1/CDK6/	4
GO:0035004	phosphatic	3/137	11/17697	7.16E-05	0.0006754	0.0004331	PIK3CA/PIK	3
GO:0035258	steroid hc	6/137	92/17697	7.97E-05	0.0007156	0.0004589	ESR1/PPARG	6
GO:0030332	cyclin bir	4/137	30/17697	8.06E-05	0.0007156	0.0004589	CDK1/CDK6/	4
GO:0043548	phosphatic	4/137	30/17697	8.06E-05	0.0007156	0.0004589	IGF1R/PDGF	4
GO:0004175	endopeptic	12/137	427/17697	0.0001224	0.0010659	0.0006836	CTSB/MMP2/	12
GO:0042562	hormone bi	6/137	102/17697	0.0001412	0.0012073	0.0007743	AR/VDR/IGF	6
GO:0004222	metalloenc	6/137	103/17697	0.000149	0.0012503	0.0008019	MMP2/MMP9/	6
GO:0002039	p53 bindir	5/137	66/17697	0.0001581	0.0012813	0.0008218	GSK3B/HIF1	5
GO:0001228	DNA-bindir	12/137	439/17697	0.0001584	0.0012813	0.0008218	AR/ESR1/PG	12
GO:0052742	phosphatic	3/137	16/17697	0.0002361	0.0018762	0.0012033	PIK3CA/PIK	3
GO:0005005	transmembr	3/137	17/17697	0.000285	0.0022263	0.0014278	EPHA2/EPHE	3
GO:0030331	estrogen r	4/137	42/17697	0.0003062	0.0023512	0.0015079	ESR1/PPARG	4
GO:0031435	mitogen-ac	3/137	18/17697	0.0003401	0.0025679	0.0016469	MAPK1/MAP2	3
GO:0030295	protein ki	5/137	80/17697	0.00039	0.0028498	0.0018277	MAP2K1/PIK	5
GO:0046332	SMAD bindi	5/137	80/17697	0.00039	0.0028498	0.0018277	TGFBR1/PAF	5
GO:0004715	non-membra	4/137	46/17697	0.0004359	0.0030853	0.0019787	SRC/PTK2/J	4
GO:0032813	tumor necr	4/137	46/17697	0.0004359	0.0030853	0.0019787	STAT1/TNF/	4
GO:0002020	protease b	6/137	128/17697	0.0004829	0.0033657	0.0021586	GSK3B/KIT/	6
GO:0033613	activating	5/137	85/17697	0.000516	0.0035419	0.0022715	PPARG/HNF4	5
GO:0020037	heme bindi	6/137	135/17697	0.0006401	0.0043276	0.0027755	CYP17A1/NC	6
GO:0001223	transcript	3/137	23/17697	0.0007176	0.0046438	0.0029782	ESR1/TERT/	3
GO:0017134	fibroblast	3/137	23/17697	0.0007176	0.0046438	0.0029782	FGFR1/FGFF	3
GO:0070412	R-SMAD bir	3/137	23/17697	0.0007176	0.0046438	0.0029782	PARP1/JUN/	3
GO:0005496	steroid bi	5/137	95/17697	0.000857	0.0054682	0.003507	AR/ESR1/ES	5
GO:0051287	NAD bindir	4/137	56/17697	0.0009242	0.0057721	0.0037018	PARP1/ALDH	4
GO:0046906	tetrapyrrc	6/137	145/17697	0.0009302	0.0057721	0.0037018	CYP17A1/NC	6
GO:0001968	fibronecti	3/137	27/17697	0.0011587	0.0070929	0.0045489	IGFBP3/VEG	3
GO:0043539	protein se	3/137	29/17697	0.0014312	0.0085306	0.005471	MAP2K1/MAF	3
GO:0051059	NF-kappaB	3/137	29/17697	0.0014312	0.0085306	0.005471	GSK3B/EP30	3
GO:0004252	serine-typ	6/137	160/17697	0.0015435	0.0090805	0.0058237	MMP2/MMP9/	6
GO:0005164	tumor necr	3/137	31/17697	0.0017408	0.0101101	0.006484	STAT1/TNF/	3
GO:0051721	protein pł	3/137	32/17697	0.0019101	0.0109528	0.0070244	STAT1/BCL2	3
GO:0008201	heparin bi	6/137	169/17697	0.0020362	0.011504	0.0073779	FGFR1/VEGF	6
GO:0005539	glycosamir	7/137	229/17697	0.002057	0.011504	0.0073779	FGFR1/VEGF	7
GO:0097718	disorderec	3/137	33/17697	0.0020893	0.011542	0.0074023	HSP90AA1/C	3
GO:0070491	repressing	4/137	71/17697	0.002236	0.0122039	0.0078268	PPARG/STAT	4
GO:0008237	metallopep	6/137	181/17697	0.0028679	0.0154663	0.0099191	MMP2/MMP9/	6
GO:0017147	Wnt-protei	3/137	37/17697	0.0029091	0.0155037	0.0099431	MET/SFRP1/	3
GO:0008236	serine-typ	6/137	182/17697	0.0029472	0.0155242	0.0099563	MMP2/MMP9/	6
GO:0034713	type I tra	2/137	11/17697	0.0031258	0.0161675	0.0103688	TGFB1/TGFF	2
GO:0042056	chemoattra	3/137	38/17697	0.0031407	0.0161675	0.0103688	VEGFA/LGAL	3
GO:0019955	cytokine b	5/137	128/17697	0.0031956	0.0162652	0.0104315	TGFBR1/KIT	5
GO:0017171	serine hyc	6/137	186/17697	0.0032808	0.0165135	0.0105907	MMP2/MMP9/	6
GO:1901681	sulfur con	7/137	250/17697	0.0033535	0.0166939	0.0107064	FGFR1/VEGF	7
GO:0035591	signaling	4/137	80/17697	0.0034503	0.0168063	0.0107785	PTPN11/SRC	4
GO:0051117	ATPase bir	4/137	80/17697	0.0034503	0.0168063	0.0107785	AR/ESR1/PG	4

GO:003199	insulin-li	2/137	12/17697	0.003732	0.0179802	0.0115314	IGF1R/IGFI	2
GO:000801	beta-cater	4/137	82/17697	0.0037707	0.0179802	0.0115314	AR/ESR1/GS	4
GO:005187	Hsp90 prot	3/137	41/17697	0.0039023	0.0184139	0.0118095	KDR/HIF1A/	3
GO:004529	cadherin b	8/137	331/17697	0.0042752	0.0199658	0.0128048	SRC/KDR/ST	8
GO:001665	oxidoreduc	2/137	13/17697	0.0043883	0.0202362	0.0129782	NOS2/NQO1	2
GO:000122	transcript	3/137	43/17697	0.0044672	0.0202362	0.0129782	ESR1/TERT/	3
GO:004802	CCR chemok	3/137	43/17697	0.0044672	0.0202362	0.0129782	STAT1/STAT	3
GO:004815	tau protei	3/137	45/17697	0.0050791	0.0227804	0.01461	GSK3B/HSP9	3
GO:004280	actinin bi	3/137	46/17697	0.005403	0.0239958	0.0153894	PPARG/NFKI	3
GO:000408	carbonate	2/137	15/17697	0.0058478	0.0249909	0.0160276	CA9/CA7	2
GO:000502	transformi	2/137	15/17697	0.0058478	0.0249909	0.0160276	TGFBR1/TGI	2
GO:000516	platelet-c	2/137	15/17697	0.0058478	0.0249909	0.0160276	PDGFRB/VEC	2
GO:009715	cysteine-t	2/137	15/17697	0.0058478	0.0249909	0.0160276	CASP3/CASI	2
GO:199084	promoter-s	3/137	48/17697	0.0060875	0.0257723	0.0165287	DNMT1/STAT	3
GO:000166	G protein-	7/137	280/17697	0.0061876	0.0259536	0.016645	PTPN11/STA	7
GO:001630	phosphatic	2/137	16/17697	0.0066494	0.0273835	0.0175621	PIK3CA/PIK	2
GO:002302	MHC class	2/137	16/17697	0.0066494	0.0273835	0.0175621	HSP90AA1/I	2
GO:000516	transformi	3/137	51/17697	0.0072073	0.0294138	0.0188642	TGFBR1/TGI	3
GO:000512	cytokine a	6/137	220/17697	0.0073597	0.0294485	0.0188864	VEGFA/FGF2	6
GO:000467	transmembr	2/137	17/17697	0.007498	0.0294485	0.0188864	TGFBR1/TGI	2
GO:000512	death rece	2/137	17/17697	0.007498	0.0294485	0.0188864	CASP3/CASI	2
GO:004696	retinoid X	2/137	17/17697	0.007498	0.0294485	0.0188864	VDR/PPARG	2
GO:000507	SH3/SH2 ac	3/137	52/17697	0.0076059	0.0294485	0.0188864	PTPN11/SRC	3
GO:005066	NADP bindi	3/137	52/17697	0.0076059	0.0294485	0.0188864	NOS2/GAPDI	3
GO:000110	RNA polyme	3/137	53/17697	0.0080172	0.0305194	0.0195733	HNF4A/JUN/	3
GO:000508	protein ki	3/137	53/17697	0.0080172	0.0305194	0.0195733	SRC/TOP2A/	3
GO:000835	RNA polyme	2/137	18/17697	0.0083927	0.0316826	0.0203193	MAPK1/CDK1	2
GO:005084	extracellu	3/137	57/17697	0.0097923	0.0366604	0.0235117	VEGFA/LGAI	3
GO:000109	RNA polyme	2/137	20/17697	0.0103176	0.0383106	0.0245701	AR/ESR1	2
GO:003067	protein bi	5/137	170/17697	0.0104233	0.0383882	0.0246198	PTPN11/SRC	5
GO:005142	peptide ho	2/137	21/17697	0.0113463	0.0414508	0.026584	PTPN11/JAK	2
GO:005043	transformi	2/137	22/17697	0.0124182	0.0450037	0.0288626	TGFBR1/TGI	2
GO:000419	cysteine-t	4/137	116/17697	0.0126289	0.0454038	0.0291192	CTSB/CASP3	4
GO:003329	monocarbox	3/137	64/17697	0.0134092	0.0478297	0.030675	VDR/PPARG/	3
GO:003107	heat shock	4/137	119/17697	0.0137608	0.0483226	0.0309912	KDR/HIF1A/	4
GO:003149	chromatin	4/137	119/17697	0.0137608	0.0483226	0.0309912	STAT3/EP30	4
