# Supplementary material for: The Efficacy and Mechanism of Qinghua Jianpi Recipe in Inhibiting Canceration of Colorectal Adenoma Based on Inflammatory Cancer Transformation
Source: J Immunol Res. 2023 Feb 15;2023:4319551. doi: 10.1155/2023/4319551 (PMC9946765; doi:10.1155/2023/4319551)
Supplement: Supplementary Materials — The analysis data of the network pharmacology. Active ingredients in traditional Chinese medicine (1); 1011 targets in colorectal cancer (2); PPI topological analysis (3); topological analysis of 213 active components in the network diagram (4); MCODE analysis (5); biological processes (BP, GO enrichment analysis) (6); cell components (CC, GO enrichment analysis) (7); molecular function (MF, GO enrichment analysis) (8); KEGG analysis (9). [file 4319551.f1.zip › PPI topological analysis.pdf]

SUID	AverageSh	Betweenne	Closeness	Clusterin	Degree	Eccentric	IsSingleN	name
77	1.139706	0.048563	0.877419	0.399941	117	2	FALSE	GAPDH
131	1.191176	0.0341	0.839506	0.424354	110	2	FALSE	HRAS
137	1.191176	0.034873	0.839506	0.412344	110	2	FALSE	AKT1
95	1.198529	0.031158	0.834356	0.41879	109	2	FALSE	EGFR
75	1.205882	0.027231	0.829268	0.436656	108	2	FALSE	CCND1
139	1.205882	0.023945	0.829268	0.444618	108	2	FALSE	VEGFA
103	1.25	0.021247	0.8	0.460881	102	2	FALSE	CASP3
91	1.257353	0.021168	0.795322	0.474257	101	2	FALSE	STAT3
115	1.264706	0.027498	0.790698	0.466465	100	2	FALSE	SRC
113	1.286765	0.019535	0.777143	0.469502	97	2	FALSE	JUN
83	1.294118	0.020906	0.772727	0.470614	96	2	FALSE	MAPK3
129	1.301471	0.02181	0.768362	0.473236	95	2	FALSE	ESR1
199	1.323529	0.019479	0.755556	0.485428	92	2	FALSE	MAPK1
107	1.338235	0.017643	0.747253	0.485144	90	2	FALSE	HSP90AA1
93	1.345588	0.015281	0.743169	0.489786	89	2	FALSE	ERBB2
320	1.375	0.010465	0.727273	0.553762	86	3	FALSE	MTOR
121	1.397059	0.011304	0.715789	0.528154	82	2	FALSE	MAPK8
89	1.426471	0.009909	0.701031	0.532468	78	2	FALSE	FGF2
127	1.433824	0.010354	0.697436	0.561859	77	2	FALSE	TNF
245	1.441176	0.010273	0.693878	0.510595	77	3	FALSE	PIK3CA
101	1.448529	0.007336	0.690355	0.593153	75	2	FALSE	BCL2L1
330	1.448529	0.007991	0.690355	0.561081	75	2	FALSE	MMP9
143	1.455882	0.008633	0.686869	0.557942	74	2	FALSE	MDM2
87	1.485294	0.006046	0.673267	0.580538	70	2	FALSE	KDR
111	1.485294	0.023016	0.673267	0.574741	70	2	FALSE	PTGS2
203	1.5	0.00588	0.666667	0.586918	68	2	FALSE	MMP2
261	1.529412	0.007081	0.653846	0.6	66	3	FALSE	ATM
85	1.522059	0.005762	0.657005	0.584615	65	2	FALSE	EP300
163	1.529412	0.006839	0.653846	0.616567	64	2	FALSE	AR
453	1.544118	0.004197	0.647619	0.637481	63	3	FALSE	JAK2
217	1.544118	0.003926	0.647619	0.639344	62	2	FALSE	MAPK14
366	1.566176	0.005885	0.638498	0.527763	62	3	FALSE	PIK3R1
368	1.544118	0.004467	0.647619	0.613432	62	2	FALSE	HIF1A
255	1.551471	0.003761	0.64455	0.654098	61	2	FALSE	IGF1R
313	1.566176	0.004813	0.638498	0.644068	60	3	FALSE	CASP8
325	1.566176	0.002922	0.638498	0.694915	60	3	FALSE	MCL1
193	1.573529	0.006177	0.635514	0.592636	59	3	FALSE	EZH2
274	1.573529	0.00286	0.635514	0.6955	59	3	FALSE	MAP2K1
342	1.595588	0.004626	0.626728	0.603146	58	3	FALSE	GRB2
209	1.595588	0.002803	0.626728	0.654135	57	3	FALSE	RPS6KB1
317	1.588235	0.002313	0.62963	0.697368	57	3	FALSE	STAT1
352	1.588235	0.00288	0.62963	0.686717	57	3	FALSE	RELA
180	1.595588	0.002571	0.626728	0.692208	56	3	FALSE	CXCR4
232	1.602941	0.003709	0.623853	0.662987	56	3	FALSE	CDK4
73	1.610294	0.002477	0.621005	0.691824	54	3	FALSE	IL2
300	1.625	0.002804	0.615385	0.648041	53	3	FALSE	PTPN11
171	1.632353	0.002024	0.612613	0.692549	51	3	FALSE	MET
228	1.632353	0.002683	0.612613	0.74902	51	3	FALSE	CCNB1
242	1.625	0.003601	0.615385	0.667451	51	2	FALSE	IL1B
258	1.639706	0.003351	0.609865	0.681569	51	3	FALSE	CCNA2
211	1.639706	0.002321	0.609865	0.698776	50	3	FALSE	NFKB1
327	1.639706	0.002217	0.609865	0.706939	50	3	FALSE	XIAP
207	1.654412	0.001891	0.604444	0.713435	49	3	FALSE	TGFB1

165	1.661765	0.002124	0.60177	0.666667	48	3	FALSE	PDGFRB
191	1.647059	0.003552	0.607143	0.681738	48	2	FALSE	PGR
279	1.647059	0.002285	0.607143	0.657801	48	2	FALSE	TERT
292	1.647059	0.004155	0.607143	0.615248	48	2	FALSE	GSK3B
322	1.661765	0.001714	0.60177	0.728723	48	3	FALSE	PARP1
188	1.654412	0.001798	0.604444	0.695652	47	2	FALSE	PPARG
304	1.669118	0.001439	0.599119	0.742831	47	3	FALSE	JAK1
97	1.669118	0.001683	0.599119	0.659903	46	3	FALSE	KIT
253	1.676471	0.00201	0.596491	0.733333	46	3	FALSE	CDK2
151	1.683824	0.002953	0.593886	0.692929	45	3	FALSE	CHEK1
302	1.691176	0.001324	0.591304	0.730303	45	3	FALSE	PTK2
340	1.683824	0.002043	0.593886	0.686869	45	3	FALSE	AKT2
346	1.698529	0.002227	0.588745	0.72833	44	3	FALSE	CDK1
225	1.705882	0.001204	0.586207	0.753045	43	3	FALSE	RAF1
249	1.698529	0.001167	0.588745	0.777409	43	3	FALSE	ICAM1
309	1.705882	0.002704	0.586207	0.624585	43	3	FALSE	RAC1
251	1.713235	0.001467	0.583691	0.753659	41	3	FALSE	CDK6
175	1.713235	0.001242	0.583691	0.707692	40	3	FALSE	ITGB1
267	1.727941	0.001663	0.578723	0.605128	40	3	FALSE	PIK3CB
490	1.720588	0.001093	0.581197	0.730769	40	3	FALSE	FLT1
315	1.713235	0.002017	0.583691	0.65857	39	2	FALSE	DNMT1
119	1.735294	0.001401	0.576271	0.751067	38	3	FALSE	CHEK2
421	1.742647	0.001228	0.57384	0.717718	37	3	FALSE	ALK
79	1.742647	0.006005	0.57384	0.622222	36	3	FALSE	ABCG2
201	1.75	0.001279	0.571429	0.768254	36	3	FALSE	AURKA
332	1.75	6.78E-04	0.571429	0.804762	36	3	FALSE	PLAU
271	1.757353	5.89E-04	0.569038	0.823529	35	3	FALSE	MMP3
282	1.75	0.005626	0.571429	0.719328	35	3	FALSE	HNF4A
290	1.757353	4.10E-04	0.569038	0.860504	35	3	FALSE	MMP1
72	1.75	0.001802	0.571429	0.672014	34	2	FALSE	ABCB1
99	1.757353	0.001508	0.569038	0.634581	34	3	FALSE	MGMT
445	1.764706	0.001225	0.566667	0.7041	34	3	FALSE	RET
155	1.764706	0.001943	0.566667	0.623106	33	3	FALSE	GLI1
277	1.772059	7.29E-04	0.564315	0.763258	33	3	FALSE	MMP14
552	1.779412	0.001833	0.561983	0.577652	33	3	FALSE	PRKCA
105	1.779412	0.00221	0.561983	0.65121	32	3	FALSE	TYMS
135	1.772059	7.18E-04	0.564315	0.764113	32	3	FALSE	SLC2A1
356	1.794118	0.001405	0.557377	0.578629	32	3	FALSE	IDH1
133	1.794118	0.001103	0.557377	0.703226	31	3	FALSE	TOP2A
235	1.786765	2.72E-04	0.559671	0.88046	30	3	FALSE	MMP7
562	1.794118	3.60E-04	0.557377	0.811494	30	3	FALSE	FGFR3
566	1.794118	4.59E-04	0.557377	0.786207	30	3	FALSE	FGFR2
81	1.808824	6.01E-04	0.552846	0.741379	29	3	FALSE	LGALS3
148	1.808824	0.001375	0.552846	0.637931	29	3	FALSE	BCL2
358	1.808824	3.51E-04	0.552846	0.807882	29	3	FALSE	FGFR1
581	1.808824	7.77E-04	0.552846	0.805419	29	3	FALSE	PLK1
1343	1.801471	6.77E-04	0.555102	0.726601	29	3	FALSE	FLT4
109	1.808824	6.02E-04	0.552846	0.78836	28	3	FALSE	TOP1
182	1.801471	9.84E-04	0.555102	0.777778	28	3	FALSE	ESR2
238	1.808824	2.16E-04	0.552846	0.859788	28	3	FALSE	MAP2K2
295	1.808824	4.80E-04	0.552846	0.806878	28	3	FALSE	NOS2
288	1.823529	5.63E-04	0.548387	0.746439	27	3	FALSE	PKM
338	1.823529	6.01E-04	0.548387	0.763533	27	3	FALSE	IGFBP3
123	1.816176	0.005328	0.550607	0.53	25	2	FALSE	GSTP1

307	1.860294	4.98E-04	0.537549	0.72	25	3	FALSE	CTSB
311	1.845588	9.44E-04	0.541833	0.75	25	3	FALSE	EPHA2
451	1.845588	0.001123	0.541833	0.673333	25	3	FALSE	TGFBR1
336	1.838235	2.21E-04	0.544	0.858696	24	3	FALSE	CA9
265	1.860294	4.84E-04	0.537549	0.758893	23	3	FALSE	BRAF
221	1.882353	3.48E-04	0.53125	0.738095	21	3	FALSE	CTSD
230	1.889706	4.28E-04	0.529183	0.647619	21	3	FALSE	VHL
370	1.867647	7.70E-05	0.535433	0.894737	20	3	FALSE	VDR
969	1.882353	8.98E-04	0.53125	0.578947	20	3	FALSE	EPHB2
285	1.875	0.00245	0.533333	0.730994	19	3	FALSE	NQO1
748	1.897059	2.72E-04	0.527132	0.766082	19	3	FALSE	NTRK1
989	1.882353	2.42E-04	0.53125	0.725146	19	3	FALSE	TGFBR2
363	1.904412	1.65E-04	0.525097	0.75817	18	3	FALSE	MAP2K4
298	1.904412	4.37E-04	0.525097	0.551471	17	3	FALSE	MEN1
550	1.889706	3.69E-04	0.529183	0.691176	17	3	FALSE	DAPK1
205	1.897059	2.24E-04	0.527132	0.741667	16	3	FALSE	SFRP1
117	1.926471	0.001856	0.519084	0.580952	15	3	FALSE	CYP1A1
219	1.889706	3.15E-04	0.529183	0.714286	15	2	FALSE	ODC1
344	1.948529	9.13E-05	0.513208	0.838095	15	3	FALSE	BAD
584	2.213235	4.03E-06	0.451827	0.989011	14	3	FALSE	BUB1
125	1.955882	1.45E-04	0.511278	0.690909	11	3	FALSE	ABCC1
269	1.948529	1.61E-05	0.513208	0.909091	11	3	FALSE	MST1R
1822	1.977941	5.81E-06	0.505576	0.945455	11	3	FALSE	NTRK3
508	2.007353	1.25E-04	0.498169	0.535714	8	3	FALSE	CYP17A1
223	2.007353	2.53E-05	0.498169	0.761905	7	3	FALSE	SMO
2134	2.080882	6.85E-06	0.480565	0.866667	6	3	FALSE	PLA2G2A
160	2.507353	5.58E-05	0.398827	0.5	5	3	FALSE	EPHX1
1581	2.073529	5.73E-06	0.48227	0.9	5	3	FALSE	EPHA3
417	2.102941	0	0.475524	1	3	3	FALSE	ALDH2
765	2.477941	0	0.403561	0	1	3	FALSE	CA7

NeighborhNumberOfDNumberOfUPartnerOfRadiality					selected	SelfLoops	shared na	Stress
50.16239	117	0	0	0.953431	FALSE	0	GAPDH	10766
51.66364	110	0	0	0.936275	FALSE	0	HRAS	8754
50.99091	110	0	0	0.936275	FALSE	0	AKT1	8798
51.34862	109	0	0	0.933824	FALSE	0	EGFR	8284
52.42593	108	0	0	0.931373	FALSE	0	CCND1	7910
52.78704	108	0	0	0.931373	FALSE	0	VEGFA	7618
53.76471	102	0	0	0.916667	FALSE	0	CASP3	6874
54.52475	101	0	0	0.914216	FALSE	0	STAT3	6802
53.88	100	0	0	0.911765	FALSE	0	SRC	6906
54.39175	97	0	0	0.904412	FALSE	0	JUN	6314
54.03125	96	0	0	0.901961	FALSE	0	MAPK3	6254
54.61053	95	0	0	0.89951	FALSE	0	ESR1	6120
55.16304	92	0	0	0.892157	FALSE	0	MAPK1	5788
55.07778	90	0	0	0.887255	FALSE	0	HSP90AA1	5186
55.77528	89	0	0	0.884804	FALSE	0	ERBB2	4900
58.87209	86	0	0	0.875	FALSE	0	MTOR	3892
57.2561	82	0	0	0.867647	FALSE	0	MAPK8	3800
56.96154	78	0	0	0.857843	FALSE	0	FGF2	3320
58.85714	77	0	0	0.855392	FALSE	0	TNF	3350
56.25974	77	0	0	0.852941	FALSE	0	PIK3CA	3494
61.2	75	0	0	0.85049	FALSE	0	BCL2L1	2864
58.90667	75	0	0	0.85049	FALSE	0	MMP9	3020
58.98649	74	0	0	0.848039	FALSE	0	MDM2	2860
59.98571	70	0	0	0.838235	FALSE	0	KDR	2298
59.61429	70	0	0	0.838235	FALSE	0	PTGS2	5334
60.04412	68	0	0	0.833333	FALSE	0	MMP2	2268
60.5303	66	0	0	0.823529	FALSE	0	ATM	3178
61.13846	65	0	0	0.82598	FALSE	0	EP300	2134
63.32813	64	0	0	0.823529	FALSE	0	AR	2216
63.52381	63	0	0	0.818627	FALSE	0	JAK2	1620
63.82258	62	0	0	0.818627	FALSE	0	MAPK14	1584
56.43548	62	0	0	0.811275	FALSE	0	PIK3R1	2032
62.87097	62	0	0	0.818627	FALSE	0	HIF1A	1760
65	61	0	0	0.816176	FALSE	0	IGF1R	1482
64.01667	60	0	0	0.811275	FALSE	0	CASP8	1566
66.58333	60	0	0	0.811275	FALSE	0	MCL1	1306
59.86441	59	0	0	0.808824	FALSE	0	EZH2	2714
66.76271	59	0	0	0.808824	FALSE	0	MAP2K1	1246
59.77586	58	0	0	0.801471	FALSE	0	GRB2	1450
65.10526	57	0	0	0.801471	FALSE	0	RPS6KB1	1246
66.80702	57	0	0	0.803922	FALSE	0	STAT1	1048
66.52632	57	0	0	0.803922	FALSE	0	RELA	1154
64.73214	56	0	0	0.801471	FALSE	0	CXCR4	1056
62.89286	56	0	0	0.79902	FALSE	0	CDK4	2234
66.44444	54	0	0	0.796569	FALSE	0	IL2	928
61.81132	53	0	0	0.791667	FALSE	0	PTPN11	1124
67.82353	51	0	0	0.789216	FALSE	0	MET	892
67.43137	51	0	0	0.789216	FALSE	0	CCNB1	1912
63.31373	51	0	0	0.791667	FALSE	0	IL1B	1280
64.88235	51	0	0	0.786765	FALSE	0	CCNA2	2000
67.28	50	0	0	0.786765	FALSE	0	NFKB1	822
67.22	50	0	0	0.786765	FALSE	0	XIAP	954
65.26531	49	0	0	0.781863	FALSE	0	TGFB1	718

66.27083	48	0	0	0.779412	FALSE	0	PDGFRB	830
68.72917	48	0	0	0.784314	FALSE	0	PGR	1126
65.52083	48	0	0	0.784314	FALSE	0	TERT	934
64.0625	48	0	0	0.784314	FALSE	0	GSK3B	1308
67.64583	48	0	0	0.779412	FALSE	0	PARP1	738
66.48936	47	0	0	0.781863	FALSE	0	PPARG	736
68.91489	47	0	0	0.776961	FALSE	0	JAK1	608
67.71739	46	0	0	0.776961	FALSE	0	KIT	784
65.76087	46	0	0	0.77451	FALSE	0	CDK2	1580
61.75556	45	0	0	0.772059	FALSE	0	CHEK1	1486
68.73333	45	0	0	0.769608	FALSE	0	PTK2	562
68.4	45	0	0	0.772059	FALSE	0	AKT2	738
63.63636	44	0	0	0.767157	FALSE	0	CDK1	1426
70.09302	43	0	0	0.764706	FALSE	0	RAF1	504
67.93023	43	0	0	0.767157	FALSE	0	ICAM1	414
61.53488	43	0	0	0.764706	FALSE	0	RAC1	768
66.21951	41	0	0	0.762255	FALSE	0	CDK6	1272
64.4	40	0	0	0.762255	FALSE	0	ITGB1	528
57.425	40	0	0	0.757353	FALSE	0	PIK3CB	670
69.05	40	0	0	0.759804	FALSE	0	FLT1	446
63.51282	39	0	0	0.762255	FALSE	0	DNMT1	630
62.5	38	0	0	0.754902	FALSE	0	CHEK2	1052
70.51351	37	0	0	0.752451	FALSE	0	ALK	422
63.91667	36	0	0	0.752451	FALSE	0	ABCG2	2596
63.02778	36	0	0	0.75	FALSE	0	AURKA	946
69.52778	36	0	0	0.75	FALSE	0	PLAU	304
69.37143	35	0	0	0.747549	FALSE	0	MMP3	248
71.6	35	0	0	0.75	FALSE	0	HNF4A	2704
71.42857	35	0	0	0.747549	FALSE	0	MMP1	202
69.41176	34	0	0	0.75	FALSE	0	ABCB1	514
60.26471	34	0	0	0.747549	FALSE	0	MGMT	514
69.79412	34	0	0	0.745098	FALSE	0	RET	376
65.72727	33	0	0	0.745098	FALSE	0	GLI1	520
65.72727	33	0	0	0.742647	FALSE	0	MMP14	286
60.75758	33	0	0	0.740196	FALSE	0	PRKCA	532
57.59375	32	0	0	0.740196	FALSE	0	TYMS	996
71.34375	32	0	0	0.742647	FALSE	0	SLC2A1	294
58.3125	32	0	0	0.735294	FALSE	0	IDH1	468
57.87097	31	0	0	0.735294	FALSE	0	TOP2A	760
76.33333	30	0	0	0.737745	FALSE	0	MMP7	148
73.7	30	0	0	0.735294	FALSE	0	FGFR3	186
72.2	30	0	0	0.735294	FALSE	0	FGFR2	208
67.48276	29	0	0	0.730392	FALSE	0	LGALS3	248
63.13793	29	0	0	0.730392	FALSE	0	BCL2	388
76.82759	29	0	0	0.730392	FALSE	0	FGFR1	180
60.58621	29	0	0	0.730392	FALSE	0	PLK1	596
64.51724	29	0	0	0.732843	FALSE	0	FLT4	256
65.60714	28	0	0	0.730392	FALSE	0	TOP1	200
78	28	0	0	0.732843	FALSE	0	ESR2	342
74.67857	28	0	0	0.730392	FALSE	0	MAP2K2	118
71.5	28	0	0	0.730392	FALSE	0	NOS2	164
73	27	0	0	0.72549	FALSE	0	PKM	198
70.62963	27	0	0	0.72549	FALSE	0	IGFBP3	182
56.2	25	0	0	0.727941	FALSE	0	GSTP1	1750

64.24	25	0	0	0.713235	FALSE	0	CTSB	170
71.16	25	0	0	0.718137	FALSE	0	EPHA2	276
68.12	25	0	0	0.718137	FALSE	0	TGFBR1	308
75.91667	24	0	0	0.720588	FALSE	0	CA9	92
69.43478	23	0	0	0.713235	FALSE	0	BRAF	162
67.85714	21	0	0	0.705882	FALSE	0	CTSD	122
62.71429	21	0	0	0.703431	FALSE	0	VHL	172
78.15	20	0	0	0.710784	FALSE	0	VDR	50
60.95	20	0	0	0.705882	FALSE	0	EPHB2	272
73.52632	19	0	0	0.708333	FALSE	0	NQO1	1410
76.10526	19	0	0	0.70098	FALSE	0	NTRK1	86
70.78947	19	0	0	0.705882	FALSE	0	TGFBR2	114
72.11111	18	0	0	0.698529	FALSE	0	MAP2K4	74
58.11765	17	0	0	0.698529	FALSE	0	MEN1	136
66.88235	17	0	0	0.703431	FALSE	0	DAPK1	102
67.375	16	0	0	0.70098	FALSE	0	SFRP1	72
49.26667	15	0	0	0.691176	FALSE	0	CYP1A1	686
73.4	15	0	0	0.703431	FALSE	0	ODC1	82
67	15	0	0	0.683824	FALSE	0	BAD	34
44.64286	14	0	0	0.595588	FALSE	0	BUB1	2
58.09091	11	0	0	0.681373	FALSE	0	ABCC1	46
84.45455	11	0	0	0.683824	FALSE	0	MST1R	10
84	11	0	0	0.67402	FALSE	0	NTRK3	6
50	8	0	0	0.664216	FALSE	0	CYP17A1	36
83.14286	7	0	0	0.664216	FALSE	0	SMO	10
72.33333	6	0	0	0.639706	FALSE	0	PLA2G2A	4
26	5	0	0	0.497549	FALSE	0	EPHX1	12
71.2	5	0	0	0.642157	FALSE	0	EPHA3	2
52.33333	3	0	0	0.632353	FALSE	0	ALDH2	0
70	1	0	0	0.507353	FALSE	0	CA7	0

TopologicalCoefficient
0.368841
0.37988
0.374933
0.377563
0.385485
0.38814
0.395329
0.400917
0.396176
0.399939
0.397289
0.401548
0.405611
0.404984
0.410112
0.43609
0.421001
0.418835
0.432773
0.416739
0.45
0.433137
0.433724
0.441071
0.441481
0.441501
0.451719
0.449548
0.465648
0.470547
0.469284
0.424327
0.462287
0.477941
0.474198
0.49321
0.44344
0.494539
0.449443
0.48586
0.494867
0.492788
0.479497
0.46935
0.492181
0.461279
0.502397
0.499492
0.465542
0.484197
0.49837
0.497926
0.487055

0.494558
0.505362
0.481771
0.471048
0.50482
0.488892
0.51429
0.50161
0.490753
0.460862
0.516792
0.510448
0.478469
0.527015
0.506942
0.462668
0.494175
0.477037
0.431767
0.515299
0.467006
0.466418
0.52622
0.473457
0.470357
0.518864
0.517697
0.534115
0.533049
0.510381
0.446405
0.520852
0.486869
0.490502
0.456824
0.429804
0.528472
0.441761
0.43512
0.565432
0.55
0.538806
0.507389
0.474721
0.577651
0.455535
0.481472
0.489606
0.577778
0.557303
0.533582
0.548872
0.53105
0.413235

0.494154
0.539091
0.519695
0.566542
0.526021
0.517993
0.482418
0.583209
0.461742
0.548704
0.580956
0.532252
0.550467
0.440285
0.499122
0.502799
0.376081
0.539706
0.523438
0.480031
0.443442
0.639807
0.65625
0.395833
0.649554
0.607843
0.422951
0.58843
0.439776
0
