# Supplementary material for: The Efficacy and Mechanism of Qinghua Jianpi Recipe in Inhibiting Canceration of Colorectal Adenoma Based on Inflammatory Cancer Transformation
Source: J Immunol Res. 2023 Feb 15;2023:4319551. doi: 10.1155/2023/4319551 (PMC9946765; doi:10.1155/2023/4319551)
Supplement: Supplementary Materials — The analysis data of the network pharmacology. Active ingredients in traditional Chinese medicine (1); 1011 targets in colorectal cancer (2); PPI topological analysis (3); topological analysis of 213 active components in the network diagram (4); MCODE analysis (5); biological processes (BP, GO enrichment analysis) (6); cell components (CC, GO enrichment analysis) (7); molecular function (MF, GO enrichment analysis) (8); KEGG analysis (9). [file 4319551.f1.zip › Topological analysis of 213 active components in the network diagram.pdf]

SUID	AverageShort	BetweennessC	ClosenessCen	Clusterin	Degree	Eccentric	IsSingleN
19953	2.2679558	0.00851156	0.4409257	0	38	4	FALSE
19475	2.22928177	0.00510109	0.44857497	0	37	4	FALSE
19868	2.2679558	0.0062462	0.4409257	0	35	4	FALSE
21000	2.28453039	0.00338916	0.43772672	0	35	4	FALSE
16860	2.29005525	0.00517431	0.43667069	0	34	4	FALSE
17771	2.26243094	0.005234	0.44200244	0	34	4	FALSE
18617	2.29558011	0.00401947	0.43561974	0	34	4	FALSE
19513	2.24585635	0.00584225	0.44526445	0	34	4	FALSE
20758	2.28453039	0.0034079	0.43772672	0	34	4	FALSE
20926	2.29005525	0.00319561	0.43667069	0	34	4	FALSE
21257	2.27348066	0.0032994	0.43985419	0	34	4	FALSE
16589	2.31767956	0.00570832	0.43146603	0	33	4	FALSE
19992	2.27348066	0.0029228	0.43985419	0	33	4	FALSE
20027	2.26243094	0.0053369	0.44200244	0	33	4	FALSE
20794	2.29005525	0.00286212	0.43667069	0	33	4	FALSE
20829	2.28453039	0.00288606	0.43772672	0	33	4	FALSE
21222	2.29558011	0.00321674	0.43561974	0	33	4	FALSE
21293	2.29558011	0.00306214	0.43561974	0	33	4	FALSE
21394	2.28453039	0.0032493	0.43772672	0	33	4	FALSE
17301	2.29558011	0.00380471	0.43561974	0	32	4	FALSE
17656	2.48342541	0.00469916	0.40266963	0	32	4	FALSE
19163	2.30110497	0.00437672	0.43457383	0	32	4	FALSE
19243	2.30110497	0.00484743	0.43457383	0	32	4	FALSE
20239	2.30662983	0.00443258	0.43353293	0	32	4	FALSE
21468	2.29558011	0.00302092	0.43561974	0	32	4	FALSE
18432	2.32320442	0.0042257	0.43043995	0	31	4	FALSE
18500	2.27900552	0.00729104	0.43878788	0	31	4	FALSE
18561	2.35082873	0.00398202	0.4253819	0	31	4	FALSE
19413	2.27900552	0.00316779	0.43878788	0	31	4	FALSE
19572	2.29558011	0.00362284	0.43561974	0	31	4	FALSE
20529	2.45027624	0.00436212	0.40811725	0	31	4	FALSE
20726	2.30110497	0.00303184	0.43457383	0	31	4	FALSE
21328	2.29558011	0.0028836	0.43561974	0	31	4	FALSE
21361	2.30110497	0.00253976	0.43457383	0	31	4	FALSE
19062	2.28453039	0.0036761	0.43772672	0	30	4	FALSE
19638	2.49447514	0.00332463	0.40088594	0	30	4	FALSE
19692	2.28453039	0.00584403	0.43772672	0	30	4	FALSE
20962	2.30110497	0.00244366	0.43457383	0	30	4	FALSE
21191	2.35082873	0.00369367	0.4253819	0	30	4	FALSE
21428	2.30662983	0.00247327	0.43353293	0	30	4	FALSE
17944	2.33425414	0.00630246	0.42840237	0	29	4	FALSE
18372	2.31767956	0.0038876	0.43146603	0	29	4	FALSE
18680	2.29558011	0.00562445	0.43561974	0	29	4	FALSE
18946	2.34530387	0.00426933	0.42638398	0	29	4	FALSE
19382	2.29005525	0.00306995	0.43667069	0	29	4	FALSE
19604	2.49447514	0.00311999	0.40088594	0	29	4	FALSE

20354	2.29558011	0.00449062	0.43561974	0	29	4	FALSE
20864	2.36187845	0.00386088	0.42339181	0	29	4	FALSE
18403	2.30662983	0.00211717	0.43353293	0	28	4	FALSE
18843	2.32872928	0.00373702	0.42941874	0	28	4	FALSE
19093	2.34530387	0.00339427	0.42638398	0	28	4	FALSE
19445	2.33977901	0.00253914	0.42739079	0	28	4	FALSE
20059	2.34530387	0.00396688	0.42638398	0	28	4	FALSE
17531	2.32872928	0.00379744	0.42941874	0	27	4	FALSE
18268	2.32320442	0.00380153	0.43043995	0	27	4	FALSE
19029	2.32320442	0.00307222	0.43043995	0	27	4	FALSE
19723	2.33425414	0.00331201	0.42840237	0	27	4	FALSE
20895	2.36187845	0.00277047	0.42339181	0	27	4	FALSE
21948	2.36740331	0.00483089	0.42240373	0	27	4	FALSE
22105	2.41712707	0.00533787	0.41371429	0	27	4	FALSE
17197	2.4281768	0.00424017	0.41183163	0	26	4	FALSE
17570	2.59392265	0.00350454	0.38551651	0	26	4	FALSE
17616	2.43922652	0.0038839	0.40996602	0	26	4	FALSE
18532	2.37845304	0.0028564	0.42044135	0	26	4	FALSE
18652	2.35635359	0.00271301	0.42438453	0	26	4	FALSE
18816	2.33425414	0.00291018	0.42840237	0	26	4	FALSE
21161	2.34530387	0.00343115	0.42638398	0	26	4	FALSE
21819	2.51104972	0.00446804	0.39823982	0	26	4	FALSE
16632	2.34530387	0.00440244	0.42638398	0	25	4	FALSE
16788	2.3839779	0.00346661	0.41946698	0	25	4	FALSE
18222	2.33977901	0.0026578	0.42739079	0	25	4	FALSE
18742	2.35635359	0.00245736	0.42438453	0	25	4	FALSE
19546	2.33977901	0.00358937	0.42739079	0	25	4	FALSE
20275	2.37292818	0.00304444	0.42142026	0	25	4	FALSE
16923	2.36740331	0.00267434	0.42240373	0	24	4	FALSE
17225	2.31767956	0.00436884	0.43146603	0	24	4	FALSE
18314	2.33425414	0.00333264	0.42840237	0	24	4	FALSE
20119	2.33425414	0.00278624	0.42840237	0	24	4	FALSE
20700	2.35635359	0.00236922	0.42438453	0	24	4	FALSE
19286	2.36187845	0.00240823	0.42339181	0	23	4	FALSE
21094	2.40607735	0.00222755	0.41561424	0	23	4	FALSE
19122	2.48895028	0.0020219	0.4017758	0	22	4	FALSE
20676	2.36740331	0.00255031	0.42240373	0	22	4	FALSE
21764	2.50552486	0.00286561	0.39911797	0	22	4	FALSE
16838	2.47237569	0.00379195	0.40446927	0	21	4	FALSE
18984	2.36740331	0.00237876	0.42240373	0	21	4	FALSE
19006	2.37292818	0.00210003	0.42142026	0	21	4	FALSE
19670	2.38950276	0.00211627	0.41849711	0	21	4	FALSE
21685	2.58839779	0.00229379	0.38633938	0	21	4	FALSE
21742	2.62154696	0.00250726	0.38145416	0	21	5	FALSE
17896	2.43922652	0.00285939	0.40996602	0	20	4	FALSE
18464	2.41712707	0.00195488	0.41371429	0	20	4	FALSE
19345	2.3839779	0.00227968	0.41946698	0	20	4	FALSE

20090	2.3839779	0.00168558	0.41946698	0	20	4	FALSE
21122	2.42265193	0.00138718	0.41277081	0	20	4	FALSE
22219	2.36740331	0.00327185	0.42240373	0	20	4	FALSE
19202	2.43370166	0.00150335	0.41089671	0	19	4	FALSE
19325	2.37845304	0.00193607	0.42044135	0	19	4	FALSE
19751	2.40607735	0.00242169	0.41561424	0	19	4	FALSE
19772	2.43922652	0.00120171	0.40996602	0	19	4	FALSE
17717	2.62707182	0.00124784	0.38065195	0	18	4	FALSE
17736	2.62707182	0.00124784	0.38065195	0	18	4	FALSE
18598	2.38950276	0.00217376	0.41849711	0	18	4	FALSE
18769	2.41712707	0.00209264	0.41371429	0	18	4	FALSE
16959	2.5441989	0.00281154	0.39305103	0	17	4	FALSE
17598	2.6878453	0.00190341	0.37204522	0	17	5	FALSE
17859	2.48342541	0.00154638	0.40266963	0	17	4	FALSE
17878	2.48342541	0.00154638	0.40266963	0	17	4	FALSE
17917	2.51657459	0.00151319	0.39736553	0	17	4	FALSE
18051	2.51104972	0.00154839	0.39823982	0	17	4	FALSE
18296	2.40607735	0.00138434	0.41561424	0	17	4	FALSE
18928	2.37845304	0.00227297	0.42044135	0	17	4	FALSE
19145	2.47237569	0.00111189	0.40446927	0	17	4	FALSE
21143	2.43922652	0.00109504	0.40996602	0	17	4	FALSE
17513	2.55524862	0.00133479	0.39135135	0	16	4	FALSE
17976	2.53867403	0.00130316	0.39390642	0	16	4	FALSE
18355	2.4558011	0.00107979	0.4071991	0	16	4	FALSE
22034	2.46132597	0.00182376	0.40628507	0	16	4	FALSE
17260	2.55524862	0.00180763	0.39135135	0	15	4	FALSE
17693	2.52762431	0.00241289	0.39562842	0	15	4	FALSE
18069	2.54972376	0.00107504	0.39219935	0	15	4	FALSE
18339	2.45027624	9.73E-04	0.40811725	0	15	4	FALSE
18800	2.43922652	0.00105778	0.40996602	0	15	4	FALSE
20571	2.69337017	8.61E-04	0.37128205	0	15	4	FALSE
21078	2.57734807	0.00177678	0.38799571	0	15	4	FALSE
17037	2.48895028	0.00159288	0.4017758	0	14	4	FALSE
17345	2.5441989	0.00126352	0.39305103	0	14	4	FALSE
18253	2.62707182	7.67E-04	0.38065195	0	14	5	FALSE
18898	2.48895028	9.32E-04	0.4017758	0	14	4	FALSE
18913	2.41712707	0.00117474	0.41371429	0	14	4	FALSE
19310	2.48895028	9.32E-04	0.4017758	0	14	4	FALSE
19367	2.49447514	4.62E-04	0.40088594	0	14	4	FALSE
19931	2.4558011	0.00118321	0.4071991	0	14	4	FALSE
16663	2.42265193	0.00321957	0.41277081	0	13	4	FALSE
16977	2.69337017	0.00181548	0.37128205	0	13	4	FALSE
17063	2.50552486	0.00240505	0.39911797	0	13	4	FALSE
17375	2.58839779	8.99E-04	0.38633938	0	13	4	FALSE
18486	2.4558011	0.0011497	0.4071991	0	13	4	FALSE
21728	2.56629834	8.19E-04	0.38966631	0	13	4	FALSE
22271	2.60497238	0.00115831	0.38388123	0	13	4	FALSE

16908	2.48342541	0.0016344	0.40266963	0	12	4	FALSE
17050	2.54972376	7.84E-04	0.39219935	0	12	4	FALSE
17288	2.48895028	0.00121977	0.4017758	0	12	4	FALSE
18014	2.60497238	0.00138725	0.38388123	0	12	4	FALSE
18027	2.49447514	0.00103162	0.40088594	0	12	4	FALSE
18788	2.48342541	0.00117597	0.40266963	0	12	4	FALSE
18873	2.4281768	0.00109913	0.41183163	0	12	4	FALSE
18886	2.4558011	0.00111218	0.4071991	0	12	4	FALSE
19796	2.4281768	0.00109913	0.41183163	0	12	4	FALSE
19823	2.62707182	6.79E-04	0.38065195	0	12	5	FALSE
19845	2.5	8.90E-04	0.4	0	12	4	FALSE
16991	2.77624309	0.00136677	0.360199	0	11	5	FALSE
17003	2.77624309	0.00136677	0.360199	0	11	5	FALSE
17185	2.57734807	8.71E-04	0.38799571	0	11	4	FALSE
16948	2.62707182	7.36E-04	0.38065195	0	10	4	FALSE
17335	2.67679558	9.37E-04	0.37358101	0	10	4	FALSE
17645	2.77624309	4.94E-04	0.360199	0	10	4	FALSE
18040	2.61049724	6.86E-04	0.38306878	0	10	4	FALSE
19858	2.54972376	8.98E-04	0.39219935	0	10	4	FALSE
22207	2.67679558	8.80E-04	0.37358101	0	10	4	FALSE
16485	2.59944751	0.00276141	0.38469713	0	9	4	FALSE
16504	2.66022099	0.00216761	0.37590862	0	9	4	FALSE
17397	2.64364641	5.24E-04	0.37826541	0	9	4	FALSE
19222	2.50552486	9.89E-04	0.39911797	0	9	4	FALSE
21635	2.62154696	4.45E-04	0.38145416	0	9	4	FALSE
22065	2.63812155	9.53E-04	0.37905759	0	9	4	FALSE
16495	2.70441989	0.00169559	0.36976507	0	8	4	FALSE
16829	2.74309392	7.13E-04	0.36455186	0	8	4	FALSE
17407	2.61049724	4.85E-04	0.38306878	0	8	4	FALSE
17935	2.84254144	2.76E-04	0.35179786	0	8	4	FALSE
18006	2.63259669	8.14E-04	0.3798531	0	8	4	FALSE
18733	2.52762431	2.82E-04	0.39562842	0	8	4	FALSE
19809	2.74309392	2.61E-04	0.36455186	0	8	4	FALSE
19836	2.5718232	2.28E-04	0.38882922	0	8	4	FALSE
21708	2.60497238	3.85E-04	0.38388123	0	8	4	FALSE
16624	2.65469613	6.04E-04	0.37669095	0	7	4	FALSE
16894	2.75966851	4.93E-04	0.36236236	0	7	5	FALSE
17176	2.64364641	3.74E-04	0.37826541	0	7	4	FALSE
17280	2.70441989	6.78E-04	0.36976507	0	7	4	FALSE
17755	2.80939227	2.29E-04	0.35594887	0	7	4	FALSE
18213	2.7320442	5.79E-04	0.36602629	0	7	4	FALSE
19278	2.54972376	1.78E-04	0.39219935	0	7	4	FALSE
20305	2.82596685	6.16E-04	0.35386119	0	7	4	FALSE
22249	2.83149171	4.33E-04	0.35317073	0	7	4	FALSE
16815	2.89779006	2.57E-04	0.34509056	0	6	5	FALSE
16822	2.89779006	2.57E-04	0.34509056	0	6	5	FALSE
16901	2.89779006	2.57E-04	0.34509056	0	6	5	FALSE

16917	2.66022099	4.53E-04	0.37590862	0	6	4	FALSE
17015	2.89779006	2.57E-04	0.34509056	0	6	5	FALSE
17022	2.89779006	2.57E-04	0.34509056	0	6	5	FALSE
17999	2.95856354	2.70E-04	0.33800187	0	6	4	FALSE
18977	2.81491713	1.64E-04	0.35525025	0	6	5	FALSE
19946	2.58839779	2.68E-04	0.38633938	0	6	4	FALSE
20313	2.82596685	4.73E-04	0.35386119	0	6	4	FALSE
20993	2.89226519	1.18E-04	0.34574976	0	6	4	FALSE
16657	2.79281768	3.53E-04	0.35806133	0	5	4	FALSE
17249	2.85359116	3.13E-04	0.35043562	0	5	4	FALSE
17993	2.72651934	1.85E-04	0.36676798	0	5	4	FALSE
19237	2.87569061	2.06E-04	0.34774256	0	5	5	FALSE
17255	3.15745856	1.40E-04	0.31671041	0	4	5	FALSE
17276	2.93646409	1.82E-04	0.34054563	0	4	4	FALSE
17371	3.45027624	1.72E-04	0.28983187	0	4	5	FALSE
19057	2.76519337	5.68E-05	0.36163836	0	4	5	FALSE
19232	2.98618785	7.19E-05	0.33487512	0	4	5	FALSE
20331	3.33425414	1.42E-04	0.29991715	0	4	5	FALSE
21118	3.24585635	1.34E-04	0.30808511	0	4	5	FALSE
19792	2.78176796	3.17E-05	0.35948361	0	3	5	FALSE
20567	3.43370166	9.79E-05	0.29123089	0	3	5	FALSE
28231	3.4558011	1.17E-04	0.28936851	0	3	5	FALSE
18250	3.00276243	6.50E-06	0.33302668	0	2	5	FALSE
19820	3.03038674	2.62E-05	0.32999088	0	2	5	FALSE
20146	3.00276243	6.50E-06	0.33302668	0	2	5	FALSE
20588	3.48895028	2.75E-05	0.28661916	0	2	5	FALSE

name	NeighborhoodCo	NumberOfD	NumberOfU	PartnerOfI	Radiality	selected	SelfLoops
M1	42.73684211	38	0	0	0.74640884	TRUE	0
M2	58.51351351	37	0	0	0.75414365	TRUE	0
M4	47.62857143	35	0	0	0.74640884	TRUE	0
M3	57.77142857	35	0	0	0.74309392	TRUE	0
M6	55.88235294	34	0	0	0.74198895	TRUE	0
M7	55.47058824	34	0	0	0.74751381	TRUE	0
M10	56.26470588	34	0	0	0.74088398	TRUE	0
M8	56.14705882	34	0	0	0.75082873	TRUE	0
M11	54.26470588	34	0	0	0.74309392	TRUE	0
M9	57.08823529	34	0	0	0.74198895	TRUE	0
M5	56.23529412	34	0	0	0.74530387	TRUE	0
M16	50.63636364	33	0	0	0.73646409	TRUE	0
M14	58.6969697	33	0	0	0.74530387	TRUE	0
M18	53.78787879	33	0	0	0.74751381	TRUE	0
M17	52.48484848	33	0	0	0.74198895	TRUE	0
M12	58.60606061	33	0	0	0.74309392	TRUE	0
M13	59.96969697	33	0	0	0.74088398	TRUE	0
M15	59.18181818	33	0	0	0.74088398	TRUE	0
M19	53	33	0	0	0.74309392	TRUE	0
M21	58.5625	32	0	0	0.74088398	TRUE	0
M22	38.1875	32	0	0	0.70331492	TRUE	0
M20	42.40625	32	0	0	0.73977901	TRUE	0
M23	47.4375	32	0	0	0.73977901	TRUE	0
M25	52.78125	32	0	0	0.73867403	TRUE	0
M24	55.9375	32	0	0	0.74088398	TRUE	0
M31	50.93548387	31	0	0	0.73535912	TRUE	0
M33	38.61290323	31	0	0	0.7441989	TRUE	0
M27	46.96774194	31	0	0	0.72983425	TRUE	0
M34	56.38709677	31	0	0	0.7441989	TRUE	0
M29	51.61290323	31	0	0	0.74088398	TRUE	0
M32	42.41935484	31	0	0	0.70994475	TRUE	0
M30	54.87096774	31	0	0	0.73977901	TRUE	0
M26	57.25806452	31	0	0	0.74088398	TRUE	0
M28	58.06451613	31	0	0	0.73977901	TRUE	0
M39	54.73333333	30	0	0	0.74309392	TRUE	0
M38	45	30	0	0	0.70110497	TRUE	0
M36	53.1	30	0	0	0.74309392	TRUE	0
M35	57.63333333	30	0	0	0.73977901	TRUE	0
M37	48.16666667	30	0	0	0.72983425	TRUE	0
M40	63.43333333	30	0	0	0.73867403	TRUE	0
M43	47.13793103	29	0	0	0.73314917	TRUE	0
M45	48.31034483	29	0	0	0.73646409	TRUE	0
M44	45.93103448	29	0	0	0.74088398	TRUE	0
M41	52.44827586	29	0	0	0.73093923	TRUE	0
M47	57.13793103	29	0	0	0.74198895	TRUE	0
M46	40.03448276	29	0	0	0.70110497	TRUE	0

M42	49.68965517	29	0	0	0.74088398	TRUE	0
M48	52.37931034	29	0	0	0.72762431	TRUE	0
M51	52.96428571	28	0	0	0.73867403	TRUE	0
M50	52.60714286	28	0	0	0.73425414	TRUE	0
M52	53.82142857	28	0	0	0.73093923	TRUE	0
M53	57.85714286	28	0	0	0.7320442	TRUE	0
M49	48.5	28	0	0	0.73093923	TRUE	0
M59	48.11111111	27	0	0	0.73425414	TRUE	0
M54	51.37037037	27	0	0	0.73535912	TRUE	0
M57	54.2962963	27	0	0	0.73535912	TRUE	0
M55	45.7037037	27	0	0	0.73314917	TRUE	0
M60	54	27	0	0	0.72762431	TRUE	0
M58	42.66666667	27	0	0	0.72651934	TRUE	0
M56	35.92592593	27	0	0	0.71657459	TRUE	0
M64	39.15384615	26	0	0	0.71436464	TRUE	0
M65	39.15384615	26	0	0	0.68121547	TRUE	0
M63	39.15384615	26	0	0	0.7121547	TRUE	0
M66	53.03846154	26	0	0	0.72430939	TRUE	0
M68	60.23076923	26	0	0	0.72872928	TRUE	0
M67	56.88461538	26	0	0	0.73314917	TRUE	0
M62	53.65384615	26	0	0	0.73093923	TRUE	0
M61	30.65384615	26	0	0	0.69779006	TRUE	0
M70	50.24	25	0	0	0.73093923	TRUE	0
M72	50.88	25	0	0	0.72320442	TRUE	0
M74	59	25	0	0	0.7320442	TRUE	0
M69	59.68	25	0	0	0.72872928	TRUE	0
M71	44.72	25	0	0	0.7320442	TRUE	0
M73	41.64	25	0	0	0.72541436	TRUE	0
M76	52.875	24	0	0	0.72651934	TRUE	0
M75	49.25	24	0	0	0.73646409	TRUE	0
M78	58.70833333	24	0	0	0.73314917	TRUE	0
M79	51.58333333	24	0	0	0.73314917	TRUE	0
M77	50.875	24	0	0	0.72872928	TRUE	0
M81	53.65217391	23	0	0	0.72762431	TRUE	0
M80	53.2173913	23	0	0	0.71878453	TRUE	0
M83	37.95454545	22	0	0	0.70220994	TRUE	0
M84	57.04545455	22	0	0	0.72651934	TRUE	0
M82	42.13636364	22	0	0	0.69889503	TRUE	0
M90	37	21	0	0	0.70552486	TRUE	0
M87	53.66666667	21	0	0	0.72651934	TRUE	0
M85	52.57142857	21	0	0	0.72541436	TRUE	0
M88	44.9047619	21	0	0	0.72209945	TRUE	0
M89	35.38095238	21	0	0	0.68232044	TRUE	0
M86	28.23809524	21	0	0	0.67569061	TRUE	0
M92	49.6	20	0	0	0.7121547	TRUE	0
M93	50.95	20	0	0	0.71657459	TRUE	0
M95	50.1	20	0	0	0.72320442	TRUE	0

M91	55.95	20	0	0	0.72320442	TRUE	0
M96	59.7	20	0	0	0.71546961	TRUE	0
M94	46.15	20	0	0	0.72651934	TRUE	0
M98	49	19	0	0	0.71325967	TRUE	0
M100	49.26315789	19	0	0	0.72430939	TRUE	0
M99	44.68421053	19	0	0	0.71878453	TRUE	0
M97	45.57894737	19	0	0	0.7121547	TRUE	0
M102	39.5	18	0	0	0.67458564	TRUE	0
M103	39.5	18	0	0	0.67458564	TRUE	0
M104	54.44444444	18	0	0	0.72209945	TRUE	0
M101	55	18	0	0	0.71657459	TRUE	0
M114	31.94117647	17	0	0	0.69116022	TRUE	0
M105	31.82352941	17	0	0	0.66243094	TRUE	0
M111	54.94117647	17	0	0	0.70331492	TRUE	0
M106	54.94117647	17	0	0	0.70331492	TRUE	0
M109	49.88235294	17	0	0	0.69668508	TRUE	0
M110	50.23529412	17	0	0	0.69779006	TRUE	0
M113	51.05882353	17	0	0	0.71878453	TRUE	0
M107	54.64705882	17	0	0	0.72430939	TRUE	0
M108	53.88235294	17	0	0	0.70552486	TRUE	0
M112	59.35294118	17	0	0	0.7121547	TRUE	0
M115	35.6875	16	0	0	0.68895028	TRUE	0
M116	49.8125	16	0	0	0.69226519	TRUE	0
M118	58.8125	16	0	0	0.70883978	TRUE	0
M117	54.5	16	0	0	0.70773481	TRUE	0
M121	38.86666667	15	0	0	0.68895028	TRUE	0
M122	39.13333333	15	0	0	0.69447514	TRUE	0
M125	53.66666667	15	0	0	0.69005525	TRUE	0
M119	57.66666667	15	0	0	0.70994475	TRUE	0
M124	54.73333333	15	0	0	0.7121547	TRUE	0
M123	50.6	15	0	0	0.66132597	TRUE	0
M120	30.6	15	0	0	0.68453039	TRUE	0
M129	52.28571429	14	0	0	0.70220994	TRUE	0
M131	53.07142857	14	0	0	0.69116022	TRUE	0
M132	55.35714286	14	0	0	0.67458564	TRUE	0
M133	61.21428571	14	0	0	0.70220994	TRUE	0
M128	68.14285714	14	0	0	0.71657459	TRUE	0
M126	61.21428571	14	0	0	0.70220994	TRUE	0
M127	64.57142857	14	0	0	0.70110497	TRUE	0
M130	62.21428571	14	0	0	0.70883978	TRUE	0
M136	54.07692308	13	0	0	0.71546961	TRUE	0
M140	26.07692308	13	0	0	0.66132597	TRUE	0
M137	49.15384615	13	0	0	0.69889503	TRUE	0
M138	55.69230769	13	0	0	0.68232044	TRUE	0
M134	56.92307692	13	0	0	0.70883978	TRUE	0
M135	55.69230769	13	0	0	0.68674033	TRUE	0
M139	54.84615385	13	0	0	0.67900552	TRUE	0

M141	52.25	12	0	0	0.70331492	TRUE	0
M149	57.83333333	12	0	0	0.69005525	TRUE	0
M150	59.83333333	12	0	0	0.70220994	TRUE	0
M145	42.75	12	0	0	0.67900552	TRUE	0
M143	53.16666667	12	0	0	0.70110497	TRUE	0
M142	64.33333333	12	0	0	0.70331492	TRUE	0
M148	56.16666667	12	0	0	0.71436464	TRUE	0
M146	66.58333333	12	0	0	0.70883978	TRUE	0
M144	56.16666667	12	0	0	0.71436464	TRUE	0
M147	52.33333333	12	0	0	0.67458564	TRUE	0
M151	60.25	12	0	0	0.7	TRUE	0
M154	32.81818182	11	0	0	0.64475138	TRUE	0
M153	32.81818182	11	0	0	0.64475138	TRUE	0
M152	43.63636364	11	0	0	0.68453039	TRUE	0
M157	46.3	10	0	0	0.67458564	TRUE	0
M155	26	10	0	0	0.66464088	TRUE	0
M160	42.9	10	0	0	0.64475138	TRUE	0
M158	53.9	10	0	0	0.67790055	TRUE	0
M159	48.6	10	0	0	0.69005525	TRUE	0
M156	39.7	10	0	0	0.66464088	TRUE	0
M161	61.33333333	9	0	0	0.6801105	TRUE	0
M164	59.77777778	9	0	0	0.6679558	TRUE	0
M163	58.22222222	9	0	0	0.67127072	TRUE	0
M162	55.55555556	9	0	0	0.69889503	TRUE	0
M165	61.55555556	9	0	0	0.67569061	TRUE	0
M166	60.66666667	9	0	0	0.67237569	TRUE	0
M170	33.875	8	0	0	0.65911602	TRUE	0
M171	28.375	8	0	0	0.65138122	TRUE	0
M175	64.125	8	0	0	0.67790055	TRUE	0
M173	42.875	8	0	0	0.63149171	TRUE	0
M169	57.5	8	0	0	0.67348066	TRUE	0
M172	57.875	8	0	0	0.69447514	TRUE	0
M174	44	8	0	0	0.65138122	TRUE	0
M168	75.625	8	0	0	0.68563536	TRUE	0
M167	68.625	8	0	0	0.67900552	TRUE	0
M181	74.85714286	7	0	0	0.66906077	TRUE	0
M179	36.28571429	7	0	0	0.6480663	TRUE	0
M184	56.85714286	7	0	0	0.67127072	TRUE	0
M176	57.42857143	7	0	0	0.65911602	TRUE	0
M178	45.42857143	7	0	0	0.63812155	TRUE	0
M180	36.57142857	7	0	0	0.65359116	TRUE	0
M177	90.85714286	7	0	0	0.69005525	TRUE	0
M182	38.71428571	7	0	0	0.63480663	TRUE	0
M183	45.42857143	7	0	0	0.63370166	TRUE	0
M191	32.16666667	6	0	0	0.62044199	TRUE	0
M185	32.16666667	6	0	0	0.62044199	TRUE	0
M190	32.16666667	6	0	0	0.62044199	TRUE	0

M194	67.33333333	6	0	0	0.6679558	TRUE	0
M188	32.16666667	6	0	0	0.62044199	TRUE	0
M195	32.16666667	6	0	0	0.62044199	TRUE	0
M192	26.66666667	6	0	0	0.60828729	TRUE	0
M189	50.16666667	6	0	0	0.63701657	TRUE	0
M186	78.66666667	6	0	0	0.68232044	TRUE	0
M187	44.83333333	6	0	0	0.63480663	TRUE	0
M193	46.33333333	6	0	0	0.62154696	TRUE	0
M196	51.2	5	0	0	0.64143646	TRUE	0
M197	38	5	0	0	0.62928177	TRUE	0
M199	77.8	5	0	0	0.65469613	TRUE	0
M198	45.8	5	0	0	0.62486188	TRUE	0
M203	31	4	0	0	0.56850829	TRUE	0
M206	38.25	4	0	0	0.61270718	TRUE	0
M200	7.75	4	0	0	0.50994475	TRUE	0
M201	64	4	0	0	0.64696133	TRUE	0
M202	41.75	4	0	0	0.60276243	TRUE	0
M204	14.5	4	0	0	0.53314917	TRUE	0
M205	18.75	4	0	0	0.55082873	TRUE	0
M208	73.33333333	3	0	0	0.64364641	TRUE	0
M207	12.33333333	3	0	0	0.51325967	TRUE	0
M209	9.66666667	1	2	0	0.50883978	TRUE	0
M210	68	2	0	0	0.59944751	TRUE	0
M213	54.5	2	0	0	0.59392265	TRUE	0
M212	68	2	0	0	0.59944751	TRUE	0
M211	12	2	0	0	0.50220994	TRUE	0

shared name	Stress	TopologicalC	Type
Phaseolinisoflav	194054	0.20764598	mol
Jaranol	252468	0.27518428	mol
Medicarpin	184948	0.22857143	mol
acacetin	193004	0.28244492	mol
baicalein	235816	0.27304653	mol
wogonin	231420	0.26442033	mol
6-prenylated eri	200746	0.27632353	mol
kaempferol	231486	0.26386153	mol
5,2'-Dihydroxy-6	167540	0.26368666	mol
5,7,4'-Trihydrox	178644	0.27904595	mol
NEOBAICALEIN	170248	0.27076125	mol
5,7-dihydroxy-2-	185372	0.25196124	mol
Quercetin der.	159472	0.28144863	mol
quercetin	202566	0.25501391	mol
5,7,2,5-tetrahyd	144286	0.25487549	mol
5,7,2',6'-Tetrah	169064	0.2837737	mol
Moslosooflavone	186358	0.29338158	mol
Norwogonin	184302	0.28946178	mol
rivularin	153462	0.25615764	mol
luteolin	170192	0.28496287	mol
ledebouriellol	105288	0.22135417	mol
Glyasperins M	137380	0.20600124	mol
Glypallichalcone	160738	0.23103234	mol
(6aR,11aR)-9,10-	185238	0.25890625	mol
Skullcapflavone	155714	0.27196782	mol
2-[(3R)-8,8-dime	149934	0.25219941	mol
3,22-Dihydroxy-1	159866	0.18258691	mol
3'-Methoxyglabri	125450	0.23817483	mol
isorhamnetin	146494	0.2688694	mol
kanzonols W	145726	0.24932465	mol
(3R)-3-(2-hydrox	115136	0.23668203	mol
5,2',6'-Trihydro	148392	0.26668796	mol
oroxylin a	161426	0.27713332	mol
Panicolin	151156	0.2824976	mol
Glepidotin A	152430	0.26084142	mol
Licoagroisoflavo	95288	0.26190476	mol
Licochalcone B	160428	0.25291262	mol
5,8,2'-Trihydrox	142328	0.27898194	mol
Eriodyctiol (fla	117096	0.24312715	mol
Salvigenin	167428	0.30907591	mol
Cerevisterol	183576	0.23301985	mol
1-Methoxyphaseol	141066	0.23537485	mol
7-Acetoxy-2-meth	139220	0.21917578	mol
Gancaonin G	142374	0.2624912	mol
Isolicoflavonol	140332	0.27251423	mol
Licoagrocarpin	77156	0.23097327	mol

isoflavanone	158678	0.23751051	mol
5,7,4'-trihydrox	122314	0.26621404	mol
2-(3,4-dihydroxy	103180	0.25472689	mol
euchrenone	128444	0.25803571	mol
glyasperin B	136970	0.26812908	mol
Isotrifoliol	112852	0.28715729	mol
shinpterocarpin	126432	0.24111675	mol
anomalin	118488	0.23438364	mol
(2S)-7-hydroxy-2	121910	0.24935827	mol
Glabridin	129920	0.26384305	mol
licochalcone G	117722	0.22351852	mol
5,7,4'-trihydrox	110882	0.27179487	mol
beta-ecdysterone	131788	0.21477663	mol
Methyl arachidon	93580	0.18878879	mol
7-(beta-Xylosyl)	90882	0.20735786	mol
Decursin	65780	0.24775225	mol
divaricatol	83266	0.20963652	mol
3'-Hydroxy-4'-O-	108616	0.26962933	mol
7,2',4'-trihydro	116250	0.3006638	mol
DFV	108116	0.27803291	mol
DIHYDROOROXYLIN	111098	0.26459219	mol
palmatine	64718	0.17546655	mol
naringenin	118696	0.2462	mol
(2R,3R)-4-methox	106158	0.2584456	mol
(2R)-7-hydroxy-2	106030	0.28855721	mol
8-prenylated eri	116054	0.29636364	mol
Kanzonol F	93852	0.21751244	mol
3,9-di-O-methyln	86926	0.20841026	mol
ellagic acid	102272	0.26332487	mol
7-Methoxy-2-meth	131422	0.2342233	mol
(E)-3-[3,4-dihyd	137526	0.2842775	mol
Vestitol	101688	0.24917898	mol
2,6,2',4'-tetrah	97370	0.25062814	mol
HMO	93228	0.26458379	mol
Carthamidin	83770	0.27338948	mol
Glyasperin C	49950	0.20878274	mol
(2R)-7-hydroxy-5	88066	0.28163545	mol
Inophyllum E	60588	0.23641588	mol
Albiflorin	71980	0.19889503	mol
Glabranin	86118	0.26333333	mol
Glabrene	80910	0.25915291	mol
licochalcone a	69082	0.22400389	mol
berberine	48184	0.21488095	mol
epiberberine	38630	0.17572965	mol
(2R)-2-[(5R,10S,	93038	0.25851064	mol
3-(2,4-dihydroxy	70670	0.26015625	mol
Inflacoumarin A	73108	0.2479798	mol

Sigmoidin-B	71560	0.27752525	mol
Dihydrobaicalin_	66000	0.30732984	mol
2-Monoolein	101638	0.22462687	mol
Glycyrin	56574	0.25263158	mol
Inermine	65034	0.24131579	mol
Licocoumarone	65768	0.22402159	mol
licoisoflavanone	46484	0.23586745	mol
Phellopterin	36738	0.24679487	mol
phelloptorin	36738	0.24679487	mol
5,7-dihydroxy-3-	64796	0.26856505	mol
18alpha-hydroxyg	69066	0.27835052	mol
isobenzoylpaeoni	45012	0.17989056	mol
divaricatacid	29422	0.20968387	mol
(2R)-2-[(3S,5R,1	75484	0.29476053	mol
(2R)-2-[(3S,5R,1	75484	0.29476053	mol
3beta-Hydroxy-24	57636	0.27617149	mol
Poricoic acid C	58386	0.27660278	mol
(E)-1-(2,4-dihyd	53718	0.2541057	mol
Gancaonin B	65442	0.2655795	mol
glyasperin F	46850	0.28585056	mol
dihydrooroxylin	51828	0.30551278	mol
Ammidin	36080	0.20285088	mol
dehydroeburicoic	49224	0.28053161	mol
1,3-dihydroxy-9-	48536	0.30588624	mol
(2R)-5,7-dihydro	52214	0.28457447	mol
Daturilin	39400	0.22015504	mol
Mandenol	54766	0.21544256	mol
trametenolic aci	47546	0.3044316	mol
1,3-dihydroxy-8,	43782	0.29668412	mol
dehydroglyasperi	45374	0.27841105	mol
7-O-methylisomuc	29184	0.33741497	mol
bis[(2S)-2-ethyl	29284	0.17619048	mol
Spinasterol	48568	0.27722008	mol
poriferasta-7,22	37516	0.29755102	mol
(2S)-6-(2,4-dihy	25786	0.33762201	mol
gadelaidic acid	40982	0.32548263	mol
Gancaonin A	55284	0.33910534	mol
icos-5-enoic aci	40982	0.32548263	mol
Isoglycyrol	32938	0.34549689	mol
Odoratin	44068	0.32049364	mol
sitosterol	83266	0.26806527	mol
Lactiflorin	20860	0.1683015	mol
Stigmasterol	66218	0.26313577	mol
stigmast-7-enol	27626	0.32554945	mol
3-(3,4-dihydroxy	38440	0.29126603	mol
delta 7-stigmast	26846	0.31797853	mol
Sitosterol alpha	34482	0.32634033	mol

beta-sitosterol	50932	0.27260638	mol
stigmast-7-en-3-	28258	0.32291667	mol
glycitein	36260	0.31461676	mol
pachymic acid	31062	0.25150602	mol
Poricoic acid A	43102	0.28046595	mol
Calycosin	39212	0.33687943	mol
Eurycarpin A	36712	0.27861953	mol
formononetin	42716	0.33981002	mol
Licoisoflavone	36712	0.27861953	mol
Licoricone	25630	0.31492843	mol
Lupiwighteone	31040	0.32027027	mol
paeoniflorgenone	18504	0.2322495	mol
Paeoniflorigenon	18504	0.2322495	mol
5alpha-Stigmasta	23870	0.24788584	mol
Ethyl oleate (NF	21250	0.27621951	mol
methyl icosa-11,	13342	0.16129032	mol
isoimperatorin	11602	0.30583942	mol
Poricoic acid B	26368	0.31676647	mol
Mairin	24868	0.26741573	mol
[(2R)-2,3-dihydr	22944	0.24967742	mol
(3S,8S,9S,10R,13	64762	0.35490196	mol
alpha-Amyrin	53986	0.36967156	mol
Taraxerol	15020	0.35322359	mol
Glycyrol	20024	0.29174094	mol
28-norolean-17-e	14966	0.3647925	mol
CLR	28342	0.36605317	mol
8beta-ethoxy atr	34448	0.21628289	mol
8-debenzoylpaeon	11840	0.1887931	mol
ZINC03978781	16140	0.37352071	mol
7,9(11)-dehydrop	10734	0.32972441	mol
hederagenin	25796	0.34242424	mol
8-(6-hydroxy-2-b	14110	0.30910326	mol
licopyranocoumar	8102	0.29655172	mol
liquiritin	14742	0.42400568	mol
beta-daucosterol	14994	0.39779412	mol
Citromitin	18006	0.45590829	mol
Baicalin	11426	0.24503968	mol
(8S,9S,10R,13R,1	11070	0.34059233	mol
Frutinone A	11530	0.36881419	mol
Prangenidin	7482	0.3315565	mol
(-)-Medicocarpin	6264	0.24034749	mol
Glyzaglabrin	14674	0.4964483	mol
9,10-dimethoxypt	8642	0.28789531	mol
Coixenolide	12118	0.34175824	mol
4-ethyl-paeonifl	4870	0.25972222	mol
4-o-methyl-paeon	4870	0.25972222	mol
benzoyl paeonifl	4870	0.25972222	mol

campest-5-en-3be	15552	0.40946502	mol
paeoniflorin	4870	0.25972222	mol
paeoniflorin_qt	4870	0.25972222	mol
Ergosterol perox	6386	0.23765432	mol
Gancaonin H	5810	0.36419753	mol
Phaseol	11080	0.44380952	mol
Bifendate	7834	0.33207071	mol
11,13-Eicosadien	4816	0.37777778	mol
nobiletin	9008	0.36115108	mol
11-Hydroxyrankin	5120	0.2890625	mol
ergosta-7,22E-di	12098	0.50860927	mol
Glycyrrhiza flav	3698	0.35555556	mol
Chrysanthemaxant	2884	0.39473684	mol
Diop	4170	0.32675439	mol
Spinoside A(MOL0	728	0.28125	mol
Glabrone	3138	0.43150685	mol
glycyroside	1512	0.38443396	mol
FA	1364	0.3	mol
coptisine	1730	0.29583333	mol
Licoisoflavone B	2124	0.50231481	mol
5'-hydroxyiso-mu	696	0.37777778	mol
Spinoside A(MOL0	538	0.36111111	mol
(2S)-2-[4-hydrox	358	0.63207547	mol
licorice glycosi	622	0.535	mol
Xambioona	358	0.63207547	mol
isomucronulatol-	214	0.52380952	mol
